# A Systematic Review to Compare Chemical Hazard Predictions of the Zebrafish Embryotoxicity Test With Mammalian Prenatal Developmental Toxicity

**DOI:** 10.1093/toxsci/kfab072

**Published:** 2021-06-09

**Authors:** Sebastian Hoffmann, Bianca Marigliani, Sevcan Gül Akgün-Ölmez, Danielle Ireland, Rebecca Cruz, Francois Busquet, Burkhard Flick, Manoj Lalu, Elizabeth C Ghandakly, Rob B M de Vries, Hilda Witters, Robert A Wright, Metin Ölmez, Catherine Willett, Thomas Hartung, Martin L Stephens, Katya Tsaioun

**Affiliations:** 1 Evidence-Based Toxicology Collaboration (EBTC), Johns Hopkins Bloomberg School of Public Health, Baltimore, Maryland 21205, USA; 2 seh consulting + services, 33106 Paderborn, Germany; 3 Department of Science and Technology, Federal University of São Paulo (UNIFESP), São José dos Campos, 12231-280 São Paulo, Brazil; 4 Department of Pharmaceutical Toxicology, Faculty of Pharmacy, Marmara University, Istanbul, 34722, Turkey; 5 Department of Biology, Swarthmore College, Swarthmore, Pennsylvania 19081, USA; 6 Laboratory of Dental Clinical Research, Universidade Federal Fluminense, Niterói, 20520-040 Rio de Janeiro, Brazil; 7 Altertox, 1050 Brussels, Belgium; 8 Experimental Toxicology and Ecology, BASF SE, 67063 Ludwigshafen am Rhein, Germany; 9 Department of Anesthesiology and Pain Medicine, Ottawa Hospital Research Institute, Ottawa, K1H 8L6 Ontario, Canada; 10 Berman Institute of Bioethics, Johns Hopkins University, Baltimore, Maryland 21205, USA; 11 Systematic Review Centre for Laboratory Experimentation (SYRCLE), Department for Health Evidence, Radboud Institute for Health Sciences, Radboudumc, 6500HB Nijmegen, The Netherlands; 12 VITO NV, 2400 Mol, Belgium; 13 William H. Welch Medical Library, Johns Hopkins University, Baltimore, Maryland 21205, USA; 14 Umraniye Family Health Center (No. 44), Turkish Ministry of Health, 34760 Istanbul, Turkey; 15 Humane Society International, Washington, 20037 District of Columbia, USA; 16 Center for Alternatives to Animal Testing (CAAT), Johns Hopkins Bloomberg School of Public Health, Baltimore, Maryland 21205, USA

**Keywords:** systematic review, zebrafish embryotoxicity test, prenatal developmental toxicity, test method comparison

## Abstract

Originally developed to inform the acute toxicity of chemicals on fish, the zebrafish embryotoxicity test (ZET) has also been proposed for assessing the prenatal developmental toxicity of chemicals, potentially replacing mammalian studies. Although extensively evaluated in primary studies, a comprehensive review summarizing the available evidence for the ZET’s capacity is lacking. Therefore, we conducted a systematic review of how well the presence or absence of exposure-related findings in the ZET predicts prenatal development toxicity in studies with rats and rabbits. A two-tiered systematic review of the developmental toxicity literature was performed, a review of the ZET literature was followed by one of the mammalian literature. Data were extracted using DistillerSR, and study validity was assessed with an amended SYRCLE's risk-of-bias tool. Extracted data were analyzed for each species and substance, which provided the basis for comparing the 2 test methods. Although limited by the number of 24 included chemicals, our results suggest that the ZET has potential to identify chemicals that are mammalian prenatal developmental toxicants, with a tendency for overprediction. Furthermore, our analysis confirmed the need for further standardization of the ZET. In addition, we identified contextual and methodological challenges in the application of systematic review approaches to toxicological questions. One key to overcoming these challenges is a transition to more comprehensive and transparent planning, conduct and reporting of toxicological studies. The first step toward bringing about this change is to create broad awareness in the toxicological community of the need for and benefits of more evidence-based approaches.

Prenatal developmental toxicity is a pivotal concern in chemical hazard and risk assessment. Therefore, it is an integral part of many regulatory frameworks around the globe, which usually require mammalian toxicity data according to the Test Guideline 414 of the Organisation for Economic Co-operation and Development (OECD TG 414). Some regulatory frameworks require studies in 2 mammalian species, such as the European chemical regulation REACH (Registration, Evaluation and Authorization of Chemicals) for high-volume substances. In such studies, a test substance is administered to pregnant animals (most often orally to rats and rabbits) and maternal toxicity as well as fetal structural abnormalities, altered growth, and death are measured (OECD, 2018). However, the OECD TG 414 is laborious, costly, and time consuming. Also, it requires a substantial number of animals and thereby raises ethical concerns. Because of these issues, there is momentum to develop and alternative methods for prenatal developmental safety assessments. For example, the International Council for Harmonization (ICH) guideline on the detection of reproductive toxicity for human pharmaceuticals encourages the use of *in vitro* assays to support the identification of potential hazards to embryo-fetal development (ICH, 2020).

A promising approach to study prenatal developmental effects is the zebrafish embryotoxicity test (ZET). This test is 1 product arising from the increased use of the zebrafish (*Danio rerio*) as a model organism for studying the effects of chemicals and pharmaceuticals. Simple literature searches demonstrate the exponential growth of these uses of zebrafish since the late 1990s (see, eg, for environmental health, Bambino and Chu [2017] and Cassar *et al.* [2020]). The increased popularity of the zebrafish model for chemical testing has been mainly driven by the zebrafish’s breadth of applications, relevance to human health, and compatibility with high-throughput screening (Bambino and Chu, 2017; Cassar *et al.*, 2020; Garcia *et al.*, 2016; Horzmann and Freeman, 2018). In addition, the translucency of the oviparously developing zebrafish embryo, which allows direct microscopic observation throughout the entire developmental process, is an advantage for studying developmental effects.

The ZET has been developed to identify teratogenic and embryotoxic chemicals (Brannen *et al.*, 2010; He *et al.*, 2014; Selderslaghs *et al.*, 2009; Ton *et al.*, 2006; Yang *et al.*, 2009). It focuses on the first days post-fertilization, starting chemical exposure as early as during cleavage (0.7–2.2 hours post fertilization [hpf]) and ending exposure and observations at the early larval period (approximately 72–120 hpf), when morphogenesis is mostly completed (Kimmel *et al.*, 1995). The ZET focuses on toxic effects of test substances related to mortality and general and specific embryotoxicity (Beekhuijzen *et al.*, 2015). The utility of the ZET for the detection of prenatal developmental effects has been evaluated for specific classes of chemicals (Beker van Woudenberg *et al.*, 2013; Hermsen *et al.*, 2011), and the use of the ZET in combination with other test methods has been suggested and explored (Augustine-Rauch *et al.*, 2016; Kroese *et al.*, 2015; Piersma *et al.*, 2013).

However, broader application of the ZET—when either used alone or in combination with other evidence, for example, from new approach methodologies—has been impeded by substantial differences in published protocols, especially regarding exposure (duration and concentrations); outcomes to be observed; outcome interpretation; and chorionation status (Beekhuijzen *et al.*, 2015; Hamm *et al.*, 2019). Such differences can lead to discrepancies among tests assessing the same substance; thus method harmonization and standardization has been called for (Nishimura *et al.*, 2016). First steps toward the harmonization of the ZET include a promising effort led by the pharmaceutical industry toward standardization and validation (Ball *et al.*, 2014; Gustafson *et al.*, 2012) and the proposal of optimal test conditions (Beekhuijzen *et al.*, 2015). More recently, the U.S. National Toxicology Program contributed to these efforts through the Systematic Evaluation of the Application of Zebrafish in Toxicology program that identified sources of variability in ZET assays (Hamm *et al.*, 2019).

Although the ZET offers a number of compelling advantages as compared with traditional mammalian methods, a systematic assessment of its value for the evaluation of prenatal developmental effects of chemicals is lacking. An obvious choice for moving forward would be a formal validation study conducted according to internationally agreed-upon principles (OECD, 2005). This approach could build on the results obtained by Gustafson *et al.* (2012) and Ball *et al.* (2014). However, such a prospective approach entails practical and methodological challenges, such as the requirement for substantial resources and a standardized ZET protocol. To avoid the practical challenges of a prospective approach, retrospective validation has been proposed for test methods, such as the ZET, for which a substantial amount of data is already available (Balls *et al.*, 2006). Balls *et al.* (2006) also proposed that systematic review methods could be applied to collect and assess existing evidence in this context. Furthermore, one would have to consider the fact that the ZET could be used in combination with other evidence as part of a testing strategy. The construction and assessment of testing strategies entails the integration of various test methods and other information sources, typically combining testing and modelling approaches addressing distinct and complementary mechanisms. Due in no small part to the daunting methodological challenges, assessment approaches for such strategies are still being discussed (Burgdorf *et al.*, 2019; Hartung *et al.*, 2013; Piersma *et al.*, 2018).

Systematic review techniques have recently attracted substantial attention in the field of chemical risk assessment (Hoffmann *et al.*, 2017; Whaley *et al.*, 2016). Inspired by systematic reviews assessing diagnostic test accuracy (see https://methods.cochrane.org/sdt/handbook-dta-reviews; last accessed on June 15, 2021), we applied systematic review methods to retrospectively assess a specific toxicological test method. In the process, we addressed two main objectives: (1) to determine to what extent ZET and mammalian test results agree and (2) to explore the challenges of applying systematic review methodology to toxicological test method assessment. We chose the ZET primarily because we wanted to provide a comprehensive, systematic, and objective evaluation of its potential to inform the assessment of the prenatal developmental toxicity hazard of chemicals. We also expected that sufficient studies would be available to allow for a systematic review. Our systematic review of the ZET and mammalian literatures was guided by the following question: “How well does the presence or absence of treatment-related findings in the ZET predict the presence or absence of prenatal development toxicity in rat and rabbit studies (OECD TG 414 and equivalents)?” A preparatory study addressing this question and documenting initial lessons learned in the application of systematic review methods has been summarized by Stephens *et al.* (2019). Here, we present and discuss the results of the fully realized systematic review documented in our PROSPERO-registered protocol, with some modifications (Tsaioun *et al.*, 2018).

## MATERIALS AND METHODS

###  

####  

Adaptations of systematic review methods to the assessment of toxicological test method performance were explored in a preparatory study (Stephens *et al.*, 2019). Based on the findings of this study, a final review protocol was registered, to which we refer for details not reported here (Tsaioun *et al.*, 2018). The protocol was based on the template for systematic reviews of animal intervention studies proposed by de Vries *et al.* (2015). We briefly describe the protocol here, highlighting and justifying any subsequent amendments.

##### Search strategy

Literature searches were performed using PubMed, Embase (Embase.com), BIOSIS Previews (Clarivate Analytics), and TOXLINE (National Library of Medicine). (TOXNET, which included TOXLINE, was retired on December 16, 2019 [https://www.nlm.nih.gov/pubs/techbull/nd19/nd19_toxnet_new_locations.html; last accessed on June 15, 2021]. Much of TOXLINE’s content has been migrated to PubMed, with archival content available via download [https://www.nlm.nih.gov/toxnet/toxline-help.html; last accessed on June 15, 2021].) There were no language or other limitations, except for a date limitation indicated below for the mammalian searches. The search strings included a combination of keywords and terms from controlled vocabularies (ie, MeSH and Emtree) and were constructed to achieve a balance of precision and recall in the results. Search strings were designed for each of the 4 databases to identify ZET and mammalian developmental toxicity studies. These search strings were developed and run in a particular sequence, with the goal of identifying 2 sets of studies—1 for ZET and 1 for mammalian tests—examining the same chemicals.

The zebrafish searches were first run in the 4 databases on June 23, 2016. These searches included concepts for species, developmental stage, and toxicity. The results of these searches were screened for eligibility and the chemicals examined in the included studies were extracted. The mammalian searches, focused on the chemicals identified by the zebrafish searches, were then run in the 4 databases. Searches in the databases were run on July 13, July 14, and July 15, 2018. These searches covered the earliest dates in each database up to 2016, in order to match the time frame of the zebrafish searches, and included concepts for species, developmental stage, toxicity, and chemicals. For reasons outlined below, only terms for 75 of the 1436 chemicals identified by the zebrafish searches were included in the mammalian searches. Search terms for these 75 chemicals and their synonyms were derived from MeSH, Emtree, and PubChem. These chemical terms are not part of the mammalian searches that are listed in the published protocol (Tsaioun *et al.*, 2018). The final zebrafish searches and the final mammalian searches (with chemical terms) are provided here as Supplementary Material (Supplementary Material 1 [zebrafish] and Supplementary Material 2 [mammalian]). No additional sources, such as references of eligible studies, were considered.

##### Screening

Eligibility criteria for ZET studies were identical to those reported in the preparatory study (Stephens *et al.*, 2019), with the exception of studies exposing zebrafish embryos 144 hpf, in which only the observations until 120 hpf were considered eligible. Outcome measures were assigned to 3 types: mortality, general embryotoxicity, or specific embryotoxicity (Table 1). Note that we excluded behavior-related outcomes, which are frequently addressed in ZET studies (Dach *et al.*, 2019), because functional deficits are usually not investigated in mammalian prenatal developmental toxicity studies (OECD, 2018). Rather than defining eligibility by specific outcomes, ZET studies were included if outcome measures of all 3 types were observed.

**Table 1.** Summary of ZET Outcome Measures by Outcome Group and Type

Outcome Type	Outcome Group	Outcome Measure
Mortality	—	Heartbeat severely reduced, coagulation
General embryotoxicity	Hatching	Unhatched, partially hatched
	Cell viability	Overall degeneration, coagulation (local)
	Body shape (general)	Arrest, retardation
	Edema	Cranial, pericardium, or yolk edema
	Cardiovascular system	Heartbeat or blood flow decreased
	Yolk	Yolk sac or yolk sac extension still present
Specific embryotoxicity	Body shape (specific)	Curved, short, or kinked tail; short body
	Fins	Dorsal, ventral, pectoral, or caudal fin alterations
	Skin	Pigmentation alterations
	Cardiovascular system	Heart, aorta, vein, or vessel alterations
	CNS and sensory organs	Brain or nasal cavity impaired; eye or otic vesicle alterations
	Head	Mouth opening or jaw impaired
	Digestive system	Anterior, mid, posterior intestine, or anus alterations
	Trunk	Somites, spinal cord, or notochord impaired

The eligibility criteria for mammalian studies have been amended from those reported previously (Stephens *et al.*, 2019). The time frame for eligible exposures, which were defined based on most frequently used exposure windows (rat: gestation days [GDs] 5–15; rabbit: GDs 6–18), was expanded to the entire gestational period, as this was imposing an unnecessary restriction. Mammalian outcomes were grouped under 4 types: growth retardation, external abnormalities, soft tissue abnormalities, and skeletal abnormalities. Prenatal mortality was not considered, as the cause can often not be determined unambiguously (OECD, 2008).

The title and abstract screening and full-text screening of the zebrafish and mammalian studies were each carried out by 2 reviewers, who resolved conflicts through discussion or, if needed, by involving a third reviewer. In addition, title and abstract screening was aided by automated machine-learning tools: zebrafish studies were excluded when 1 reviewer confirmed exclusion suggested by the automatic exclusion functionality of SWIFT-Active Screener (Sciome LLC, https://www.sciome.com/swift-activescreener/; last accessed on June 15, 2021), and mammalian studies were included or excluded when 1 reviewer confirmed the respective suggestion obtained by applying the automated reviewer functionality of DistillerSR’s AI toolkit (Evidence Partners Inc., https://www.evidencepartners.com; last accessed on June 15, 2021).

##### Selection of chemicals

A total of 1436 chemicals were tested in the included ZET studies, with a majority of these chemicals (1060) tested using a high-throughput system (Truong *et al.*, 2014). This large number of chemicals presented challenges for developing the mammalian searches. As each chemical has multiple synonyms, even with the use of a URL-based API (Application Programming Interface) for searching PubChem, the search and data clean-up for generating the synonyms for 1436 chemicals would have been very labor- and time-intensive. A related challenge would have been the length of the resulting search strings. Very long search strings can present problems for databases, resulting in the need to split searches into multiple parts. This can lead to more than usual duplication in search results, which then needs to be removed at a later step. Furthermore, had these searching-based hurdles been overcome, it was likely that the resulting set of mammalian studies requiring screening would have been unmanageable, based on project resources.

In light of these challenges, we reduced the number of chemicals from 1436 to 75. Although possibly introducing bias, an informed, nonrepresentative selection of chemicals was preferred over a random selection, primarily because it would likely result in a set of chemicals better balancing mammalian prenatal developmental toxicants and nontoxicants. The 75 chemicals were chosen because they are represented in at least one of the following sources identified by the review team as relevant: 2 lists of reference substances (Brown, 2002; Daston *et al.*, 2014), an assessment of a human embryonic stem cell-based assay for developmental toxicity screening (Palmer *et al.*, 2013), the EPA ToxRefDB database (available at https://www.epa.gov/chemical-research/exploring-toxcast-data-downloadable-data; last accessed on June 15, 2021), and in other relevant resources (eg, Kleinstreuer *et al.*, 2011; Malir *et al.*, 2013; Palmer *et al.*, 2017). The list of 75 chemicals and the resources are provided as Supplementary Material 4.

##### Data extraction

Specific data extraction forms addressing both study characteristics and outcome data focused on outcome types were devised for ZET and mammalian studies in DistillerSR. Note that from studies exposing zebrafish embryos 144 hpf only eligible observations, that is, until 120 hpf were extracted. For ZET studies testing more than one chemical and for mammalian studies that tested a chemical on both rats and rabbits, data were extracted separately for each chemical and each species (using the clone functionality of DistillerSR). In order to address the fact that more than one set of data may be extracted from a study, we refer to datasets (rather than studies) from here onwards. Data were extracted by one reviewer, and quality control was ensured by a second reviewer by checking all extracted data. Conflicts were resolved by the 2 reviewers through discussion.

##### Critical appraisal

We critically appraised the included studies regarding their reporting completeness, their risk of bias (RoB), that is, systematic errors in study design or conduct that may lead to either an overestimation or an underestimation of the true effect (Higgins *et al.*, 2021). Because, to our knowledge, a specific tool for potential biases in toxicological studies that is based on empirical evidence is not available, we applied the RoB tool developed by the SYstematic Review Center for Laboratory animal Experimentation (SYRCLE) (Hooijmans *et al.*, 2014). Based on the Cochrane RoB tool (Higgins *et al.*, 2011), the SYRCLE tool has been developed for application to preclinical animal studies and addresses the classical biases related to selection, performance, detection, attrition, and reporting, to both mammalian and ZET studies with some modifications. We omitted the criterion addressing selective outcome reporting due to the multitude of potential outcomes and the “catch-all” criterion on biases not covered by the other domains in the tool. When applying the tool to ZET datasets, we replaced the criterion on randomized housing, which cannot be applied to zebrafish embryos, with a criterion on homogeneity of test conditions.

In addition, and deviating from the protocol, we included 3 criteria addressing reporting completeness and a set of “other” appraisal criteria not related to RoB, but considered important for data analysis, for example, dose-response and concentration-response plausibility, and issues with negative control data, such as high mortality. Plausibility of the dose-/concentration-response was determined by evaluating the change in response over time (ZET datasets) and over increasing concentrations (ZET and mammalian datasets) for each outcome, flagging nonmonotonous patterns. The “other” criteria relate to the concept of study sensitivity, that is, the ability to detect a true effect, described by Cooper *et al.* (2016).

For studies with more than one dataset, reporting and RoB criteria were assessed for the study as a whole, but the “other” criteria were applied to each dataset. All studies and datasets were appraised by one reviewer, and quality control was ensured by a second reviewer by checking all appraisals. Conflicts were resolved by the 2 reviewers through discussion.

An overview of all criteria including supportive instruction for reviewers is included in Supplementary Material 4.

##### Data analysis

Data analysis was conducted in a 3-step process as outlined in detail in the published protocol (Tsaioun *et al.*, 2018).

First, we concluded for each dataset whether the results were positive (effect(s) present), negative (no effect(s) present), or inconclusive. In brief, a ZET dataset was considered positive for embryotoxicity if any outcome of general or specific embryotoxicity was observed at any concentration and any time point. ZET datasets not meeting these criteria were considered negative or, in specific cases, for example, when the maximum test concentration was considered too low (ie, did not induce mortality or was below 1000 µM), inconclusive. A mammalian dataset was considered positive if (1) an increased number of malformations or a significant increase in variations (compared with control) were observed for at least 1 outcome and (2) these malformations or variations occurred at a dose equal to or lower than the dose causing maternal toxicity. Mammalian datasets not meeting these criteria were considered negative or, in specific cases, for example, when the maximum dose was considered too low, inconclusive.

Second, we identified the chemicals with discordant results across ZET studies or across mammalian datasets (ie, negative in some ZET/mammalian studies and positive in other ZET/mammalian studies). The respective datasets were examined to identify potential experimental reasons for the differences.

Third, the results from ZET studies were compared with the results from mammalian studies across all chemicals using contingency tables.

## RESULTS

###  

#### Summary of Searching and Screening Steps

**Figure 1.**
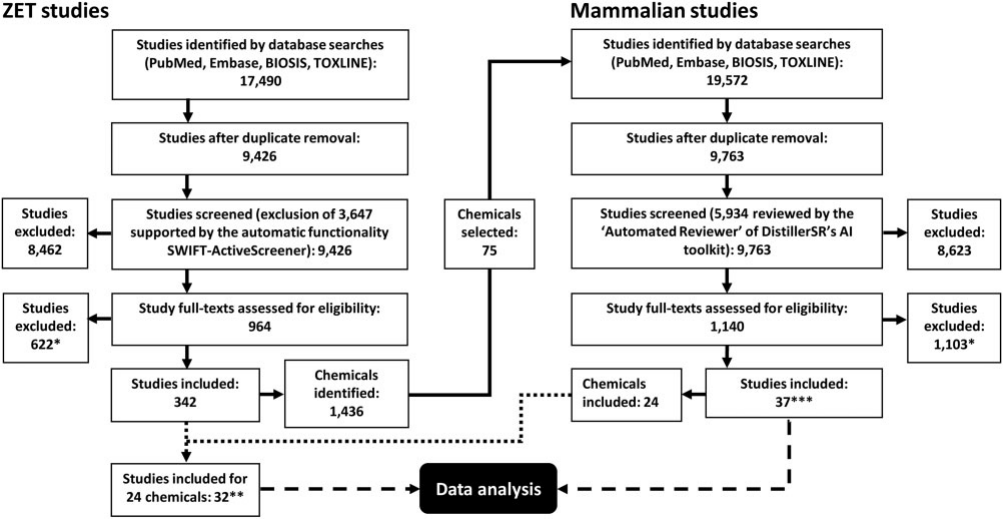
Modified PRISMA diagram (*see Table 2 for reasons for exclusion; **74 datasets; ***40 datasets).

The ZET searches generated a total of 17 490 publications. Duplicate removal reduced these to 9426 results, from which 1654 out-of-scope references (books, book chapters, meeting abstracts, non-English, patents, and research proposals) were excluded by sorting and searching reference type fields in EndNote. The remaining 7772 references were further reduced to 964 after title and abstract screening. Full-text screening for eligibility yielded 342 included studies. At this stage, studies were excluded primarily because no original data were reported (26.1%), the exposure was not started within 0–6 hpf (18.5%), less than 3 concentrations were used (17.0%), or no developmental toxicity outcomes were investigated (12.7%). A complete overview of reasons for exclusion is presented in Table 2. The 342 included ZET studies tested a total of 1436 chemicals (Figure 1). More than 1000 of these chemicals were tested in a single high-throughput study, most of them exclusively (Truong *et al.*, 2014). The majority of studies (193/342 = 56%) investigated 1 substance, whereas 15 studies (4.4%) tested more than 10 substances.

**Table 2.** Frequencies of Exclusion for Different Criteria During Full-Text Screening of Zebrafish and Mammalian Studies

Zebrafish Studies			Mammalian Studies		
Exclusion Criterion	No.	%	Exclusion Criterion	No.	%
Population: modified zebrafish	42	6.8	Population: modified rat or rabbit	20	1.8
Exposure: not single chemical exposure	33	5.3	Exposure: not oral route or not single chemical exposure	150	13.6
Outcomes: no developmental toxicity	79	12.7	Outcomes: no developmental toxicity	48	4.4
Language: not English	12	1.9	Language: not English	38	3.4
No original data reported	162	26.1	No original data reported	542	49.1
Less than 10 eggs per concentration	18	2.9	Less than 16 animals per group	149	13.5
Less than 3 concentrations	106	17.0	Less than 3 doses	86	7.8
Exposure not within 0–6 hpf	115	18.5	Other (eg, nonincluded chemical)	70	6.3
Time point of outcome assessment	30	4.8			
Other (eg, duplicates, full text unavailable, etc.)	25	4.0			
Total	622	100		1103	100

The mammalian searches generated a total of 19 572 publications. Duplicate removal reduced these to 9763 results, from which 983 out-of-scope references (non-English and research proposals) were excluded by sorting and searching reference type fields in EndNote. The remaining 8780 references were further reduced to 1140 in the title and abstract screening. Full-text screening for eligibility yielded 37 included studies (Figure 1). During full-text screening, almost half of the studies (49.1%) were excluded because no original data were reported, especially in conference abstracts (Table 2). Exclusion also occurred for the following main reasons: exposures were not eligible (including nonoral administration routes) (13.6%), group sizes were smaller than 16 (13.5%), and less than 3 doses were tested (7.8%). Because 3 of the 37 eligible studies tested a chemical in both rats and rabbits, 40 mammalian datasets were included. Twenty-four unique chemicals were represented in these 40 datasets.

In a final step, we determined which of the 342 included ZET studies tested at least 1 of the 24 chemicals from the 37 included mammalian studies. This resulted in a final included set of 32 ZET studies with 74 datasets.

The entire evidence retrieval process is summarized in Figure 1 as a PRISMA flow diagram by Moher *et al.* (2009) adapted to our review approach.

#### Characterization of the Included Studies

The 32 included ZET studies were published between 1993 and 2016. Twenty-five studies had 1 eligible dataset (ie, for 1 chemical), 5 studies had 3–6, 1 study had 9, and one study had 21 eligible datasets. Of the 24 included chemicals, 10 chemicals had 1 dataset (ie, tested in one ZET study), 7 chemicals had 2 or 3, and the remaining 7 chemicals had 5–8 datasets. The summary of the extracted data presented in Supplementary Table 1 shows heterogeneity in the experimental design and the reporting of results. For example, the number of test concentrations ranged from 3 to 10, exposure ended between 48 and 144 hpf, and the way the results were presented ranged from detailed information (ie, each outcome at each timepoint) to summary measures integrating the data across timepoints and outcomes. In addition, information relevant for the data extraction, for example, the zebrafish strain and the dechorionation status, was not reported in some cases. However, the test concentration ranges of datasets for the same substance usually overlapped. Four studies did not observe or report results for all outcome types, but were considered eligible based on embryotoxicity observed in either general or specific outcomes.

The 37 included mammalian studies were published between 1978 and 2015. Three studies tested chemicals in both rats and rabbits: Infurna *et al.* (1988) (atrazine), SDS-Biotech (1997) (cyproconazole), and Kennedy and Kaplan (1984) (hexazinone). Six studies were submitted to the Office of Toxic Substances of the U.S. Environmental Protection Agency between 1990 and 1992. Fifteen of the 24 included chemicals were tested in rats only, 2 in rabbits only, and 7n in both species (Supplementary Table 2). Eight chemicals had more than one dataset. The following were tested multiple times in rats: caffeine (3×), camphor (2×) cyproconazole (2×), ethylene glycol (3×), lovastatin (2×), 2-phenylphenol (2×), and valproic acid (2×). Atrazine was tested twice in rabbits.

Most rat studies (19/31) exposed the pregnant females from GD 6 to GD 15, which is the duration recommended in the OECD TG 414. One study had a shorter exposure duration, and 10 studies had longer exposure durations. Most rabbit studies administered tested chemicals for 13 or 14 days, starting on GD 6 or GD 7. The one exception administered thalidomide for 4 days, from GD 8 to GD 11 (Sterz *et al.*, 1987).

#### Results of the Critical Appraisal

Using 14 criteria, the reporting completeness and RoB of the included studies were critically appraised along with specific aspects important for data analysis. Details for all included studies (ZET and mammalian) are provided in Supplementary Material 5.

Reporting in the 32 included ZET studies was very poor. Twenty-seven studies failed all 3 reporting criteria and 30 studies reported insufficient information to evaluate the RoB of 6 criteria, that is, allocation sequence, allocation concealment, blinding of investigators, random outcome assessment, blinded outcome assessment, and completeness of reported outcomes. For the baseline similarity criterion, 16 studies had low RoB, 1 had a high RoB and for 15 insufficient reporting resulted in unclear RoB. The criterion addressing homogeneity of test conditions could not be assessed for 6 studies. For the remaining 26 studies (81%) a low RoB was concluded. In summary, on average the RoB of 6.5 (of 8) criteria could not be appraised due to poor reporting. Therefore, we considered all ZET studies to be at high RoB.

Information to enable assessments of whether exposures were sufficiently high or concentration-responses were plausible was usually reported in the included ZET studies. Control data issues could not be assessed due to insufficient reporting for 43% of the datasets, the majority of which were from 4 studies (Gustafson *et al.*, 2012; Hermsen *et al.*, 2011; Piersma *et al.*, 2013; Selderslaghs *et al.*, 2012). Control data issues were identified for 9 datasets from the only included high-throughput study (Truong *et al.*, 2014). This same study had issues for 6 datasets regarding the highest test concentration and for 9 datasets regarding the plausibility of the concentration-response. The concentration-response was also found to be not plausible for 5 datasets from other studies. The impact of these issues on the data analysis is discussed below.

Reporting in the 37 mammalian studies was better than for the ZET studies: randomization was mentioned in 62% of the studies and blinding in 27% of the studies, but power calculation was not mentioned in any of the studies. However, reporting across all studies was such that the RoB could be assessed for only 24% of all criteria. Reporting was particularly poor regarding the criteria addressing allocation sequence, allocation concealment, random housing, blinding of investigators, and random outcome assessment. Reporting was sufficiently detailed to conclude low RoB for 21 studies for “baseline similarity” (57%), for 10 studies for “blinded outcome assessment” and for 25 studies for “complete outcomes” (68%). A high RoB was primarily identified for “completeness of reported outcomes” (9 studies). This resulted in an average of 1.8 criteria with a low RoB per study, so that all studies were considered to be at high RoB.

The information needed to assess the other criteria informing the data analysis was usually reported in mammalian studies. There were no issues identified for 24 of the 40 datasets, 1 dataset had 3 potential issues and 15 datasets had 1 potential issue. The impact of these issues on the data analysis is discussed below.

#### Data Analysis

##### Analysis of individual datasets

The first step in the data analysis was to conclude for each dataset if the tested chemical was positive, negative, or inconclusive based on the extracted data and the procedures specified in the protocol. This step took into account issues identified by the “other” criteria, where applicable. ZET results are presented in Table 3 and mammalian results in Table 4. Both tables are sorted by chemical name and briefly summarize the experimental findings driving the results.

**Table 3.** ZET Outcomes and Results

Chemical Name (Reference)	Lowest Test Conc. (in µM) With Observed Outcomes (earliest timepoint, hpf)			Result	Justification
	Mortality	General Embryotoxicity	Specific Embryotoxicity		
Acetaminophen (Selderslaghs *et al.*, 2012)	10 120 (24)	NO	6230 (72)	+	Skeletal deformities and lack of pigmentation at 6230 µM
Acetaminophen (David and Pancharatna, 2009)	33.1 (n.r.)	33.1 (n.r.)	66.2 (n.r.)	+	Reduced hatching, body mass, and body length at 33.1 µM; tail deformities and lack of pigmentation at 66.2 µM
Acetaminophen (Truong *et al.*, 2014)	NO	NO	NO	Inc.	No effects observed up to the maximum test concentration of 64 µM
All-trans-retinoic acid (Truong *et al.*, 2014)	6.4 (120)	0.064 (120)	0.0064 (120)	+	Caudal fin effects and reduced trunk length at 0.0064 µM
All-trans-retinoic acid (Selderslaghs *et al.*, 2009)	0.0266 (48)	0.0266 (48)	0.000213 (72)	+	Kinked tail at 0.000213 µM and other effects at higher concentrations
All-trans-retinoic acid (Wang *et al.*, 2014)	0.01 (120)	0.004 (120)	0.008 (120)	+	Several general and specific effects at 0.004 and 0.008 µM
All-trans-retinoic acid (Vandersea *et al.*, 1998)	n.r.	n.r.	0.5 (72)	+	Pectoral fin effects at 0.5 µM
All-trans-retinoic acid (Teixido *et al.*, 2013)	YES*	YES*	0.005 (52)	+	Short tails at 0.005 µM
All-trans-retinoic acid (Selderslaghs *et al.*, 2012)	1.47 (72)	YES*	0.00491 (72)	+	A wide spectrum of general and specific embryotoxicity outcomes at 0.00491 µM
All-trans-retinoic acid (Piersma *et al.*, 2013)	YES*	YES*	YES*	+	A teratogenicity index (BMC 20) that included general and specific embryotoxic effects was 0.063 µM
Atrazine (Wiegand *et al.*, 2001)	46.25 (24)	92.5 (24)	46.25 (36)	+	Effects on pigmentation and otoliths at 46.25 µM
Atrazine (Weber *et al.*, 2013)	NO	NO	0.0014 (n.r.)	+	Increased head length at 0.0014 µM
Atrazine (Ton *et al.*, 2006)	500 (48)	200 (96)	200 (96)	+	Heart and trunk edema, and underdeveloped jaw at 200 µM
Atrazine (Perez *et al.*, 2013)	NO	NO	NO	Inc.	No effects observed up to the maximum test concentration of 185.5 µM
Atrazine (Truong *et al.*, 2014)	NO	NO	NO	Inc.	No effects observed up to the maximum test concentration of 64 µM
Butylparaben (Truong *et al.*, 2014)	64 (120)	64 (120)	0.64 (120)	+	Jaw effects at 0.64 and 64 µM (but not at 6.4 µM)
Caffeine (Zhu *et al.*, 2015)	5150 (54)	YES*	YES*	+	A summary index of general and specific embryotoxicity indicated embryotoxicity at sublethal concentrations
Caffeine (Chakraborty *et al.*, 2011)	NO	YES*	n.r.	+	Decreased hatching rate at 500 µM, but likely also lower concentrations (reporting is unclear)
Caffeine (Selderslaghs *et al.*, 2009)	3220 (24)	1610 (48)	390 (72)	+	Kinked tail at 390 µM and other effects at higher concentrations
Caffeine (Teixido *et al.*, 2013)	YES*	YES*	500 (52)	+	Short tails at 500 µM
Caffeine (Selderslaghs *et al.*, 2012)	3480 (72)	YES*	390 (72)	+	Skeletal deformities observed at 390 µM
Caffeine (Truong *et al.*, 2014)	0.064 (120)	6.4 (120)	6.4 (120)	+	Mortality at 0.064 and 64 µM; yolk sac and pericardial edema, as well as pectoral fin effects, at 6.4 µM
Camphor (Yim *et al.*, 2014)	395 (48)	395 (72)	395 (96)	+	Bent spine, yolk sac edema, and decreased hatching rate at 395 µM
Camphor (Selderslaghs *et al.*, 2012)	4000 (72)	NO	1230 (72)	+	Otolith abnormalities observed at 1230 µM
Clopyralid (Truong *et al.*, 2014)	0.64 (120)	NO	NO	Inc.	No embryotoxic effects up to the maximum test concentration of 64 µM
Cyproconazole (Truong *et al.*, 2014)	NO	64 (120)	64 (120)	+	A wide spectrum of embryotoxic effects at 64 µM
Cyproconazole (Hermsen *et al.*, 2011)	YES (n.r.)	31.6 (72)	31.6 (72)	+	Score used, but teratogenic effects observed at 31.6 µM
Dimethyl phthalate (Truong *et al.*, 2014)	NO	NO	NO	Inc.	No effects observed up to the maximum test concentration of 64 µM
Ethylene glycol (Truong *et al.*, 2014)	0.064 (120)	NO	NO	−	No embryotoxic effects observed up to the maximum test concentration of 64 µM, but mortality
Fluazinam (Truong *et al.*, 2014)	0.64 (120)	NO	NO	−	No embryotoxic effects up to the maximum test concentration of 64 µM, but mortality
Genistein (Ren *et al.*, 2012)	20 (120)	20 (120)	20 (120)	+	Increased malformation rate, for example, ocular effects, and high mortality observed at 20 µM
Genistein (Truong *et al.*, 2014)	0.64 (120)	64 (120)	64 (120)	+	Yolk sac and pericardial edema, jaw effects, and high mortality observed at 64 µM
Hexazinone (Truong *et al.*, 2014)	6.4 (120)	NO	NO	−	No embryotoxic effects observed up to the maximum test concentration of 64 µM, but mortality
Lovastatin (Gustafson *et al.*, 2012)″	0.1 (120)	0.1 (120)	0.1 (120)	+	NOAEL for malformations lower than LC25 (mortality)
Lovastatin (Gustafson *et al.*, 2012)″	0.1 (120)	0.1 (120)	0.1 (120)	+	NOAEL for malformations lower than LC25 (mortality)
Lovastatin (Gustafson *et al.*, 2012)″	0.1 (120)	0.1 (120)	0.1 (120)	+	NOAEL for malformations lower than LC25 (mortality)
Lovastatin (Gustafson *et al.*, 2012)″	0.03 (120)	0.001 (120)	0.001 (120)	+	NOAEL for malformations lower than LC25 (mortality)
Lovastatin (Truong *et al.*, 2014)	64 (120)	0.64 (120)	0.64 (120)	+	Wide spectrum of general and specific embryotoxicity outcomes at 0.64 µM
Methoxyacetic acid (Teixido *et al.*, 2013)	YES*	YES*	2000 (52)	+	Short tails at 2000 µM.
Methoxyacetic acid (Hermsen *et al.*, 2011)	YES (n.r.)	3160 (72)	1000 (72)	+	Score used, but teratogenic effects observed at 1000 µM
Methoxyacetic acid (*Hermsen et al.*, 2011)	NO	YES*	YES*	+	A teratogenicity index (BMC 20) that included general and specific embryotoxic effects was 6310 µM
*n*-Methylpyrrolidone (Zhang *et al.*, 2013)	8800 (72)	2640 (72)	2640 (72)	+	Small heads and eyes, retarded growth, and pericardial edema at 2640 µM
2-Phenylphenol (Truong *et al.*, 2014)	64 (120)	NO	NO	Inc.	No embryotoxic effects observed up to the maximum test concentration of 64 µM, but mortality at 64 µM
Rotenone (Pinho *et al.*, 2013)	0.316 (32)	0.316 (56)	0.0316 (32)	+	Abnormal embryos at 0.316 µM
Rotenone (Melo *et al.*, 2015)	0.025 (48)	0.025 (72)	0.018 (48)	+	Lack of pigmentation at 0.018 µM
Rotenone (Truong *et al.*, 2014)	0.64 (120)	NO	NO	−	No embryotoxic effects observed up to the maximum test concentration of 64 µM, but mortality at 0.64 µM
Tetrabromobisphenol A (Yang *et al.*, 2015)	0.92 (96)	0.92 (48)	0.92 (96)	+	Malformations at 0.92 µM
Tetrabromobisphenol A (Song *et al.*, 2014)	2.76 (48)	0.92 (72)	NO	+	Reduced hatching rate at 0.92 µM
Tetrabromobisphenol A (Noyes *et al.*, 2015)	6.4 (120)	6.4 (120)	0.64 (120)	+	Jaw malformations at 0.64 µM
Tetrabromobisphenol A (McCormick *et al.*, 2010)	3 (48)	0.75 (48)	0.75 (48)	+	Edema, hemorrhage, and tail malformations at 0.75 µM
Tetrabromobisphenol A (Hu *et al.*, 2009)	2.76 (60)	1.38 (36)	1.38 (36)	+	Malformations at 1.38 µM
Tetrabromobisphenol A (Carlsson and Norrgren, 2014)	1.84 (24)	1.84 (48)	n.r.	+	Edema and mortality at 1.84 µM
Tetrabromobisphenol A (Baumann *et al.*, 2016)	n.r.	n.r.	0.5 (120)	+	Decrease in size and pigmentation of retinal cells at 0.5 µM
Tetrabromobisphenol A (Truong *et al.*, 2014)	6.1 (120)	NO	NO	−	No embryotoxic effects observed up to the maximum test concentration of 64 µM, but mortality at 6.1 µM
Thalidomide (Gao *et al.*, 2014)	27 (96)	13.8 (n.r.)	2.76 (n.r.)	+	Pectoral fins missing at 2.76 µM
Thalidomide (Selderslaghs *et al.*, 2012)	NO	NO	NO	−	No effects observed up to the maximum soluble concentration of 150 µM
Thalidomide (Truong *et al.*, 2014)	6.4 (120)	NO	NO	−	No embryotoxic effects observed up to the maximum test concentration of 64 µM, but mortality
Thalidomide (Gustafson *et al.*, 2012)″	10 (120)	0.01 (120)	0.01 (120)	+	NOAEL for malformations lower than LC25 (mortality)
Thalidomide (Gustafson *et al.*, 2012)″	NO	NO	NO	−	No effects observed up to the maximum test concentration of 1000 µM
Thalidomide (Gustafson *et al.*, 2012)″	1000 (120)	1 (120)	1 (120)	+	NOAEL for malformations lower than LC25 (mortality)
Thalidomide (Gustafson *et al.*, 2012)″	1000 (120)	100 (120)	100 (120)	+	NOAEL for malformations lower than LC25 (mortality)
Thalidomide (Gustafson *et al.*, 2012)″	100 (120)	1 (120)	1 (120)	+	NOAEL for malformations lower than LC25 (mortality)
Triadimefon (Truong *et al.*, 2014)	NO	64 (120)	64 (120)	+	Several general and specific effects at 64 µM
Triadimefon (Hermsen *et al.*, 2011)	YES*	31.6 (72)	10 (72)	+	Score used, but embryotoxic effects at the sublethal concentration of 10 µM
Triclopyr (Truong *et al.*, 2014)	NO	NO	NO	Inc.	No effects observed up to the maximum test concentration of 64 µM
Triethylene glycol (Truong *et al.*, 2014)	NO	NO	NO	Inc.	No effects observed up to the maximum test concentration of 64 µM
Valproic acid (Herrmann, 1993)	3000 (24)	100 (20)	30 (24)	+	Short or bent tail at 30 µM
Valproic acid (Beker van Woudenberg *et al.*, 2014)	730 (n.r.)	150 (72)	150 (n.r.)	+	Brain and eye effects, as well as pericardial edema, at or approximately at 150 µM
Valproic acid (Selderslaghs *et al.*, 2009)	1500 (24)	750 (48)	1500 (48)	+	Blood circulation effects observed at 750 µM
Valproic acid (Teixido *et al.*, 2013)	YES*	50 (n.r.)	300 (n.r.)	+	Increased head-trunk angle at 50 µM (sublethal) and short tails at 300 µM (sublethal)
Valproic acid (Selderslaghs *et al.*, 2012)	1570 (72)	YES*	550 (72)	+	A wide spectrum of general and specific embryotoxicity outcomes observed
Valproic acid (Truong *et al.*, 2014)	NO	NO	NO	Inc.	No effects observed up to the maximum test concentration of 64 µM
Valproic acid # (Piersma *et al.*, 2013)	NO	YES*	YES*	+	A teratogenicity index (BMC 20) that included general and specific embryotoxic effects was 1585 µM
Valproic acid # (Lee *et al.*, 2013)	6.25 (72)	NO	6.25 (72)	+	Growth retardation at the sublethal concentration of 6.25 µM

hpf, hours post fertilization; n.r., not reported; *, data reported differently, not allowing to determine the lowest test concentration for respective outcomes; +, positive; −, negative; Inc., inconclusive; #, sodium salt; NOAEL, no observed adverse effect level; LC25, lethal concentration inducing 25% mortality; ″, same order as in Supplementary Table 1.

**Table 4.** Mammalian Outcomes and Results

Chemical Name (Reference)	Species	Retarded Growth *	Variations or Malformations* (at lowest dose)			Maternal Toxicity*	Result	Justification
			External	Visceral	Skeletal			
Acetaminophen (Burdan *et al.*, 2001)	Rat	350	—	—	—	—	+	Retarded growth at nonmaternally toxic dose (350 mg/kg bw)
All-trans-retinoic acid (Seegmiller *et al.*, 1997)	Rat	—	10	—	5	—	+	Significant increase in incomplete ossification and supernumerary ribs at nonmaternally toxic dose (5 mg/kg bw) and in cleft palate at nonmaternally toxic dose (10 mg/kg bw)
Atrazine (Infurna *et al.*, 1988)	Rat	700	—	—	70	70	+	Significant increase in incomplete ossification at lowest maternally toxic dose (70 mg/kg bw)
Atrazine (Infurna *et al.*, 1988)	Rabbit	75	—	—	75	75	+	Significant increase in nonossification at lowest maternally toxic maternal dose (75 mg/kg bw)
Butylparaben (Daston, 2004)	Rat	—	—	—	—	1000	−	No developmental effects observed at maternally toxic dose (1000 mg/kg bw)
Caffeine (Collins *et al.*, 1981)	Rat	40	80	—	40	12	−	No developmental effects observed at maternally toxic dose (12 mg/kg bw)
Caffeine (Collins *et al.*, 1983)	Rat	100.8	100.8	144	50.8	100.8	+	Significant increase in skeletal variation at nonmaternally toxic dose (50.8 mg/kg bw)
Caffeine (Collins *et al.*, 1987)	Rat	—	—	n.a.	48.8	48.8	+	Significant increase in sternebral variation at lowest maternally toxic dose (48.8 mg/kg bw)
Camphor (Navarro *et al.*, 1992a)	Rat	—	—	—	—	400	−	No developmental effects observed at maternally toxic dose (400 mg/kg bw)
Camphor (Navarro *et al.*, 1992b)	Rabbit	—	—	–	—	400	−	No developmental effects observed at maternally toxic dose (400 mg/kg bw)
Camphor (Leuschner, 1997)	Rat	—	—	—	—	1000	−	No developmental effects observed at maternally toxic dose (1000 mg/kg bw)
Clopyralid (Hayes *et al.*, 1984)	Rat	—	—	—	—	250	−	No developmental effects observed at maternally toxic dose (250 mg/kg bw)
Cyproconazole (Machera, 1995)	Rat	20	—	20	50	—	+	Retarded growth and visceral malformations at nonmaternally toxic dose (20 mg/kg bw)
Cyproconazole (SDS-Biotech, 1997)	Rat	24	48	n.a.	24	24	+	Retarded growth and skeletal malformations at lowest maternally toxic dose (24 mg/kg bw)
Cyproconazole (SDS-Biotech, 1997)	Rabbit	n.a.	—	—	—	50	−	No developmental effects observed at maternally toxic dose (50 mg/kg bw)
Dimethyl phthalate (Field *et al.*, 1993)	Rat	—	—	—	—	200	−	No developmental effects observed at maternally toxic dose (200 mg/kg bw)
Ethylene glycol (Maronpot *et al.*, 1983)	Rat	—	—	—	1000	—	+	Significant increase in poorly ossified and nonossified vertebral centra at nonmaternally toxic dose (1000 mg/kg bw)
Ethylene glycol (Neeper-Bradley, 1990)	Rat	1000	2500	2500	1000	2500	+	Retarded growth and skeletal malformations at nonmaternally toxic dose (1000 mg/kg bw)
Ethylene glycol (Price *et al.*, 1992)	Rat	2500	5000	1250	2500	1250	+	Increase in (litters with) visceral malformations at lowest maternally toxic dose (1250 mg/kg bw)
Ethylene glycol (Tyl *et al.*, 1991)	Rabbit	—	—	—	–	2000	−	No developmental effects observed at maternally toxic dose (2000 mg/kg bw)
Fluazinam (Tesh *et al.*, 1992)	Rat	50	250	250	50	50	+	Increase in skeletal malformations and retarded growth at lowest maternally toxic dose (50 mg/kg bw)
Genistein (McClain *et al.*, 2007)	Rat	—	—	—	—	500	−	No developmental effects observed at maternally toxic dose (500 mg/kg bw)
Hexazinone (Kennedy and Kaplan, 1984)	Rat	—	—	—	—	22.5	−	No developmental effects observed at maternally toxic dose (22.5 mg/kg bw)
Hexazinone (Kennedy and Kaplan, 1984)	Rabbit	—	—	—	—	125	−	No developmental effects observed at maternally toxic dose (125 mg/kg bw)
Lovastatin (Lankas *et al.*, 2004)	Rat	100	800	—	400	200	+	Retarded growth at nonmaternally toxic dose (100 mg/kg bw)
Lovastatin (Minsker *et al.*, 1983)	Rat	800	800	—	800	800	+	Retarded growth, external malformations, and skeletal malformations at lowest maternally toxic dose (800 mg/kg bw)
Methoxyacetic acid (Carney *et al.*, 2003)	Rabbit	7.5	7.5	7.5	7.5	15	+	Retarded growth, various malformations, and increased variations at nonmaternally toxic dose (7.5 mg/kg bw)
*n*-Methylpyrrolidone (Saillenfait *et al.*, 2002)	Rat	250	500	500	500	500	+	Retarded growth at nonmaternally toxic dose (250 mg/kg bw)
2-Phenylphenol (Anonymous, 1991)	Rat	—	—	—	700	700	+	Significantly delayed sternebrae ossification and skull foramen at the lowest maternally toxic dose (700 mg/kg bw)
2-Phenylphenol (Anonymous, 1992)	Rabbit	—	—	—	—	250	−	No developmental effects observed at maternally toxic dose (250 mg/kg bw)
2-Phenylphenol (Kaneda *et al.*, 1978)	Rat	600	—	300	—	300	+	Increase in visceral malformations at lowest maternally toxic dose (300 mg/kg bw)
Rotenone (Khera *et al.*, 1982)	Rat	—	—	—	5	5	+	Significant increase in extra ribs, delayed ossification, and missing sternebrae at lowest maternally toxic dose (5 mg/kg bw)
Tetrabromobisphenol A (Cope *et al.*, 2015)	Rat	—	—	—	—	—	−	No developmental effects observed at highest and nonmaternally toxic dose (1000 mg/kg bw)
Thalidomide (Sterz *et al.*, 1987)	Rabbit	n.a.	50	50	50	—	+	External, visceral, and skeletal malformations at nonmaternally toxic dose (50 mg/kg bw)
Triadimefon (Machener, 1992)	Rat	—	75	—	—	30	−	No developmental effects observed at maternally toxic dose (30 mg/kg bw)
Triclopyr (Hanley *et al.*, 1984)	Rat	—	200	—	200	200	+	External and skeletal malformations (two fetuses) at lowest maternally toxic dose (200 mg/kg bw)
Triethylene glycol (Ballantyne and Snellings, 2005)	Rat	11 260	—	—	11 260	11 260	+	Significant increase in thoracic centrum skeletal variation and retarded growth at lowest maternally toxic dose (11 200 mg/kg bw)
Valproic acid (Ong *et al.*, 1983)	Rat	600		—	150	600	+	Significant increase in rib variation at nonmaternally toxic dose (150 mg/kg bw)
Valproic acid (Petrere *et al.*, 1986)	Rabbit	—	—	—	350	—	+	Skeletal malformations at nonmaternally toxic dose (350 mg/kg bw)
Valproic acid (Vorhees, 1987)	Rat	300	200	200	300	400	+	External and visceral malformations at nonmaternally toxic dose (200 mg/kg bw)

*, unit is mg/kg bw/day; n.a., not available (not observed or not reported); +, positive; −, negative.

Of the 74 ZET datasets, 57 were positive, 8 were negative, and 9 inconclusive. All inconclusive datasets did not observe general or specific embryotoxicity, but also did not test sufficiently high doses, all being below 1000 µM. Eight of these datasets were from the only high-throughput study (Truong *et al.*, 2014), which used a default test concentration range with 64 µM being the highest test concentration. Inconclusive datasets were excluded from further analysis, reducing the number of chemicals with at least 1 conclusive ZET dataset to 19 (see Table 5). Of these 19 chemicals, 5 had 1 conclusive dataset, 8 had 2 or 3 conclusive datasets, and 6 had 5–8 conclusive datasets.

**Table 5.** Summary of All Results by Chemical

Chemical Name	ZET Studies		Mammalian Studies	
	Individual Datasets	Overall	Rat	Rabbit
Acetaminophen	+/+^1^	+	+	nd
All-trans-retinoic acid	+/+/+/+/+/+/+	+	+	nd
Atrazine	+/+/+^2^	+	+	+
Butylparaben	+	+	−	nd
Caffeine	+/+/+/+/+/+	+	−/+/+*	nd
Camphor	+/+	+	−/−	−
Cyproconazole	+/+	+	+/+	−
Ethylene glycol	−	−	+/+/+	−
Fluazinam	−	−	+	nd
Genistein	+/+	+	−	nd
Hexazinone	−	−	−	−
Lovastatin	+/+/+/+/+	+	+/+	nd
Methoxyacetic acid	+/+/+	+	nd	+
*n*-Methylpyrrolidone	+	+	+	nd
Rotenone	+/+/−	+	+	nd
Tetrabromobisphenol A	+/+/+/+/+/+/+/−	+	−	nd
Thalidomide	+/+/+/+/+/−/−/−	+	nd	+
Triadimefon	+/+	+	−	nd
Valproic acid	+/+/+/+/+/+/+^1^	+	+/+	+
Clopyralid	^1^	Inconclusive	−	nd
Dimethyl phthalate	^1^	Inconclusive	−	nd
2-Phenylphenol	^1^	Inconclusive	+/+	−
Triclopyr	^1^	Inconclusive	+	nd
Triethylene glycol	^1^	Inconclusive	+	nd

+, positive; −, negative; *, considered positive overall; nd, no data; superscript numbers (1 and 2) indicate amount of inconclusive ZET studies.

All mammalian datasets were conclusive. Of the 25 positive datasets, 21 were conducted with rats and 4 with rabbits. Of the 15 negative datasets, 10 were conducted with rats and 5 with rabbits. Two rat datasets did not report visceral outcomes but were considered eligible based on the effects for other outcomes: Collins *et al.* (1987) focused in this follow-up study of Collins *et al.* (1983) on the most sensitive outcome and confirmed skeletal effects observed earlier, and SDS-Biotech (1997) tested rabbits in parallel, for which visceral outcomes were reported, so that we assumed that no visceral effects were observed. This protocol deviation did not introduce bias as both chemicals tested in the datasets showed skeletal effects and were therefore considered positive. Two rabbit datasets did not report growth outcomes but were considered eligible based on other outcomes and information: Sterz *et al.* (1987) observed all types of malformations at the lowest dose tested, and SDS-Biotech (1997) tested rabbits in parallel, for which growth outcomes were reported, so that we assumed that no growth effects were observed. This protocol deviation did not introduce potential bias for Sterz *et al.* (1987), whereas for SDS-Biotech (1997) the test chemical may have been positive instead of negative, which would have had only a marginal effect on the data analysis.

##### Evaluation of inconsistent results

Inconsistent results (in terms of negative/positive) were evaluated in detail for the respective chemicals. For the ZET datasets inconsistent results were present for rotenone, tetrabromobisphenol A, and thalidomide. Although Truong *et al.* (2014) observed no effects other than mortality for rotenone concentrations of 0.64 µM and higher after 120 hpf, 2 studies observed effects on pigmentations at concentrations below 0.64 µM up to the last observation time points, that is, 80 and 96 hpf (Melo *et al.*, 2015; Pinho *et al.*, 2013). Similarly, although Truong *et al.* (2014) observed no effects other than mortality for tetrabromobisphenol A at concentrations of 6.1 and 61 µM, 7 studies observed embryotoxic effects at concentrations between 0.5 and 2 µM (see Table 4). The negative results for rotenone and tetrabromobisphenol A obtained by Truong *et al.* (2014) may be explained by the experimental conditions used, in particular the use of the tropical 5D zebrafish strain and the use of only one early, here not eligible and one late, here eligible assessment time point (120 hpf). Thalidomide produced the most heterogeneous results. It was positive at low concentrations in the Gao *et al.* (2014) study, where absent pectoral fins were observed at 2.76 µM. It was also positive in 4 datasets from an interlaboratory study (Gustafson *et al.*, 2012), which measured embryotoxic concentrations ranging from 0.1 to 1000 µM. However, thalidomide was also found to be negative for 1 dataset in the Gustafson *et al.* (2012) study, in the Selderslaghs *et al.* (2012) study, which tested up to 150 µM due to solubility, and the Truong *et al.* (2014) study, which was difficult to interpret due to a high negative control mortality and an unclear concentration-related mortality. Although there was no obvious explanation for these heterogeneous results, we judged thalidomide to be positive overall. In doing so, we deviated slightly from the procedure specified in the protocol, according to which a bootstrap resampling procedure should have been applied in case inexplicable discordant results were obtained for more than 5% of the chemicals included in the comparative data analysis. As such results were observed for 1 (thalidomide) of 19 included chemicals as listed in Table 5, that is, 5.3%, this procedure would have been triggered. We considered this a minor deviation from the protocol, even though it biased the overall results toward a slightly increased concordance between the ZET and the mammalian studies.

Regarding the mammalian datasets, caffeine was the only chemical showing discordant results within species, with 2 positive rat studies and 1 negative rat study. As this difference can be explained by different methods of administration (intubation vs. drinking water) (Collins *et al.*, 1983), caffeine was overall considered positive. Cyproconazole, ethylene glycol, and 2-phenylphenol showed discordant results between mammalian species, all being positive in the rat and negative in the rabbit (Table 5). These results may be due to species differences in maternal and prenatal-developmental toxicity or due to experimental differences, for example, in the determination of the dosing regimen or the choice of vehicle (Theunissen *et al.*, 2016).

Chemicals with consistent datasets results were not analyzed further in this regard, because the type of outcome is of less relevance for our hazard-focused review question.

##### Concordance of ZET and mammalian results

Deriving overall dichotomized results for all chemicals and species allowed us to conduct the planned concordance analysis, which is presented in Table 6. The total number of chemicals that could be compared was low. Because only 8 chemicals were available for a comparison of ZET studies with prenatal developmental toxicity studies in rabbits (Table 6b), these results were not considered further. Seventeen chemicals, that is, 24% of the 75 chemicals initially selected, qualified for a comparison of ZET studies with prenatal developmental toxicity studies in rats (Table 6a). The ZET studies tended to overpredict rat negative results as positive (5 out of 6 chemicals). In addition, 2 out of 3 chemicals that were negative in the ZET (ethylene glycol and fluazinam) were positive in the rat. Consequently, concordant results were obtained for 10 of the 17 chemicals (56%). When combining rat and rabbit studies in a conservative way, that is, both have to be negative for an overall negative result, while at least one has to be positive for an overall positive result, 15 chemicals (20%) qualified for the concordance analysis (Table 6c). Of the 13 chemicals that were positive in at least 1 mammalian species, 11 were also positive in the ZET. In addition, one chemical (hexazinone) was negative in all species. In summary, concordant results were obtained for 12 of the 15 chemicals (80%). Statistical significance was not calculated due to the small sample size of included chemicals.

**Table 6.** 2 × 2 Contingency Tables Comparing the ZET Results With (a) the Rat Results, (b) the Rabbit Results, and c) the Combined Rat and Rabbit Results (“and”: Negative Results for Both Species; “or”: a Positive Result for at Least One Species)

(a)		Rat		∑	(b)	Rabbit		∑	(c)	Rat and/or rabbit		∑
		−	+			−	+			− (and)	+ (or)	
ZET	−	1	2	3	2	0	2	1	2	3
	+	5	9	14	2	4	6	1	11	12
∑		6	11	17	4	4	8	2	13	15

##### Confidence in results

The two factors impacting on confidence of the entire evidence base, that is, across all chemicals, systematically analyzed were the RoB and the plausibility of concentration-/dose-response. Due to poor reporting, the evidence has high RoB, reducing our general confidence in the evidence used for the determination of concordance. The concentration-/dose-responses, as assessed under the “other” critical appraisal criteria, were considered plausible, with exception of the jaw effects observed by Truong *et al.* (2014) for butylparaben, which lacked a concentration-response, with effects at 0.64 µM and the lethal concentration of 64 µM, but not at 6.4 µM. This general plausibility increased the confidence in the overall evidence base.

However, on a chemical level, other factors impacting on the confidence were explored. For example, clearly increased severe developmental effects in the absence of general toxicity increased the confidence, for example, as observed for most all-trans-retinoic acid ZET datasets, and in Seegmiller *et al.* (1997) and Machera (1995).

In other cases, issues identified in the critical appraisal, especially by the “other” criteria that were specifically designed to highlight factors impacting the data analysis reduced the confidence. Among the ZET datasets, we identified, in addition to the above-mentioned butylparaben dataset, 2 datasets with positive results that had such issues. First, Truong *et al.* (2014) observed three significant outcomes for genistein at 64 µM, which also induced a very high mortality. Second, the decreased hatching rates observed by Chakraborty *et al.* (2011) with increasing caffeine concentrations could have been due to the difference in embryo ages at baseline. Among the mammalian studies, one dataset had unclear reporting, which reduced the confidence in its negative result. SDS-Biotech (1997) reported no growth outcomes for cyproconazole in the rabbit. Based on other details of the study, we decided that the lack of reporting was due to an absence of effects, although this was not explicitly reported.

Also the above analysis of inconsistent results informed the confidence assessment on the level of the individual chemicals. The frequency of inconsistent results was relatively low (3 out of 14 chemicals for the ZET, 1 of 7 chemicals for rat studies), and, except for the ZET results for thalidomide, potential reasons for the inconsistency of results were identified. Therefore, we considered the overall evidence base as consistent and not as a confidence-reducing factor.

Although we have not planned to integrate those 4 factors, we are confident in that the evidence base allows to draw moderately sound conclusions.

Furthermore, due to the relatively low incidence of chemicals with confidence-reducing issues and due to small sample size, we refrained from a chemical-specific data analysis approach accounting for confidence and weighted all chemicals equally in the concordance analysis.

## DISCUSSION

###  

The capacity of the ZET and the mammalian prenatal developmental toxicity test to predict prenatal developmental toxicity hazard of chemicals were systematically reviewed. The potential of the ZET to provide relevant evidence for the assessment of the prenatal developmental toxicity of chemicals has been explored extensively in primary studies. This popularity is evident from our literature search targeted to result in a homogeneous subset of ZET studies, in which we identified 1436 chemicals tested in 342 ZET studies. Informed by an initial scoping exercise (Stephens *et al.*, 2019), we decided to focus on 75 chemicals to stay within feasible dimensions of our review. The search of the mammalian literature identified 37 eligible prenatal developmental toxicity studies for 24 of the 75 chemicals. After we derived conclusions as either positive or negative for each dataset and summarized conclusions for chemicals with more than one dataset, a total of 19 chemicals were available to compare the ZET with the prenatal developmental mammalian test using 2 × 2 contingency tables.

Although the confidence in the evidence was moderate, the confidence in the results of the test method comparison was weakened by the small number of chemicals and also by a higher number of positive results on both sides. However, our review results suggest that the ZET has some potential to identify chemicals that are prenatal developmental toxicants in rats and/or rabbits. Furthermore, our analysis indicated that the ZET is overpredicting chemicals as positive that are negative in the individual mammalian species, and confirmed the need for further standardization of the ZET. To elucidate why the confidence in the test method comparison results remained weak, we discuss potential reasons that limited the evidence and reconsider decisions made when defining the systematic review protocol.

#### Selection Challenges

The systematic review was designed in such a way that the confidence in its conclusions would be driven to a major extent by the number of chemicals included. By selecting these substances in a nonrandom manner possibly introducing a bias (of unknown direction), we expected that selecting substances well-studied for prenatal developmental toxicity would result in a high number of chemicals for the concordance analysis. This assumption did not hold true, as we found eligible studies for only 24 of the 75 chemicals.

One factor contributing to the low chemical coverage could have been the stringency of our eligibility criteria, which may have excluded studies relating to any of the other 51 chemicals. However, more relaxed eligibility criteria could have led to other complications. For instance, the criteria addressing group size and number of doses could potentially have been less stringent for mammalian studies, but only for positive chemicals. For negative chemicals, a group size of at least 16 and 3 doses seems to be conventionally required to have sufficient confidence in a negative result. Such a results-based approach would have substantially increased the risk of selection bias because the eligibility of studies could then only have been determined after data analysis. There would also have been complications if the route of exposure criterion had been less stringent. The inclusion of mammalian studies with nonoral administration routes would have further increased the complexity and decreased the interpretability of the data due to route-specific absorption and metabolism.

Another factor contributing to the low number of included chemicals could have been the exclusion of regulatory databases from our set of information sources. However, although regulatory databases are likely to report findings in mammals based on OECD TG 414 and similar tests, these databases may not be publicly available, may not report original data and may not offer comprehensive search options.

Consequently, selection of more than 75 of the 1436 chemicals would have been the most promising way to increase the number of chemicals for the test method comparison. However, a selection process of such dimensions would have required more efficient approaches, for example, aided by artificial intelligence tools that are still being developed and optimized for mining existing evidence for selection purposes.

An increase in the number of included chemicals would also be the only viable approach to obtain a substantial number of chemicals that are negative in the ZET and the mammalian test. The extent of ZET development and standardization is likely an important factor contributing to a high proportion of positive results. Once the general experimental setup of a test method like the ZET has been defined, researchers usually start exploring its application by making sure that reference chemicals with well-known and clear effects are identified. This likely explains, for example, why several ZET datasets for the well-known prenatal developmental toxicants all-trans retinoic acid, thalidomide, and valproic acid were included. In a next step, the interpretation of experimental data is standardized based on the results obtained. With a strong focus on the correct identification of harmful substances, that is, a test methods’ sensitivity, exposure conditions and interpretation procedures are often tuned to be sensitive. For example, the effects of embryo dechorionization on ZET outcomes and conclusions have been discussed by Hamm *et al.* (2019). The risk of such tuning is that a test method will become overly sensitive, indicating harmful effects for most substances tested. This will inevitably lead to a reduced ability to correctly identify nonharmful substances. Our focus on well-known prenatal developmental toxicants and our requirement for a 1000 µM test concentration for negative conclusions for soluble chemicals likely resulted in the observation that the ZET was positive for 16 of the 19 chemicals with conclusive ZET data. Although we anticipated this lack of balance and attempted to account for it in the selection process for the 75 chemicals, we did not succeed in avoiding the imbalance, and this reduced the comprehensiveness of our test method comparison. This is an important lesson for researchers planning future systematic reviews comparing toxicological test methods, particularly if the 2 test methods substantially differ in their levels of development and standardization.

#### Data Extraction and Analysis Challenges

Standardization issues also impacted the data extraction step of our review. Studies of mammalian prenatal developmental toxicity have well-established guidelines for which outcomes should be measured and how outcomes should be measured and assessed, both individually and in combination, particularly fetal and maternal effects induced by the same dose (Chahoud *et al.*, 1999; Danielsson, 2013). In contrast, ZET studies differ substantially in outcomes observed and in how effects are summarized and interpreted (Beekhuijzen *et al.*, 2015). This is reflected, for example, in our data extraction for cases in which we could determine that effects were observed, but not at which concentration and at which timepoint (see Supplementary Table 1). This lack of ZET standardization led to discrepancies between the results of studies, for example, when different outcomes are observed, different concentrations are tested, and different outcome assessment timepoints are used.

Data analysis challenges relate to the discrimination of positive and negative results. This process leads to cases that are clearly positive or negative, but also to borderline cases, which are usually associated with a higher level of uncertainty (Gabbert *et al.*, 2020). Indeed, our conservative interpretation of ZET data led to positive results of such borderline cases. A good example is the positive result determined for the only ZET study with the highly water-soluble chemical *n*-methylpyrrolidone (Zhang *et al.*, 2013), which clearly induced embryotoxic effects at nonlethal, but very high concentrations, that is, ≥ 2640 µM. Had *n*-methylpyrrolidone been tested only up to 1000 µM, Zhang *et al.* (2013) data suggest that no effects would have been observed, which would have led to a negative result according to our data analysis criteria. A similar example is the positive result for the only mammalian study testing triclopyr, which showed a low incidence of malformation at the maternally toxic dose of 200 mg/kg bw (Hanley *et al.*, 1984). Although such malformations were not observed in the other dose groups and the control, a historical database of negative control data may have shown a similarly low background incidence of such malformations, which may have resulted in a negative result.

#### Detailed Discussion of Two Example Chemicals

Accounting for additional relevant evidence, we evaluated in further detail 2 chemicals, camphor and fluazinam, to better understand the results obtained and potentially decrease uncertainty associated with them.

Camphor was the only chemical without any prenatal developmental effects in both mammalian species (Navarro *et al.*, 1992a,b; Leuschner, 1997). These results are strengthened by another negative rabbit study included in Leuschner (1997) that was considered not eligible in our review because of group sizes smaller than 16. Based on the same studies, the European Food Safety Agency (EFSA) also concluded that camphor is not a prenatal developmental toxicant in mammals (EFSA, 2008). In addition, camphor is easily absorbed in the gastrointestinal tract and is metabolized initially by oxidation, which is possibly species specific. Some human evidence exists that suggests that camphor does not induce prenatal developmental toxicity in humans (Heinonen *et al.*, 1977). In contrast, our review of the 2 included ZET camphor datasets concluded a positive result for both.

Yim *et al.* (2014) observed coagulation and general embryotoxic effects (yolk sac edema, pericardial edema, and delayed hatching) in a concentration- and time-dependent manner. At 790 µM, both edema types were found in approximately 25% of the embryos and coagulation was found in 20% of the embryos. Two specific embryotoxic effects were also observed. Bent spine was primarily induced by the lowest test concentration of 395 µM and to a minor extent at higher concentrations. Ocular defects were observed at 790 and 1580 µM, the latter concentration leading to 60%–70% coagulation. The interpretation of the data was impaired by the fact that negative control data were reported for coagulation and hatching only. The second ZET camphor study, Selderslaghs *et al.* (2012), observed only 1 embryotoxic effect. At 72 hpf, abnormal otoliths were found in 50% of the embryos treated with 1230 µM, a concentration 3–4-fold lower than the concentration that induced 50% lethality. As effects were reported in terms of LC50 and EC50 only, it cannot be determined at which concentration abnormal otoliths started to occur and if other embryotoxic effects were present in less than 50% of the embryos.

The results of the 2 ZET studies, which tested similar concentrations, but different camphor forms using different vehicles, are difficult to compare, mainly because outcome results were reported differently. However, even though the concentrations inducing about 50% lethality differed in the 2 studies by approximately a factor of 2, no contradictory results were obtained. When the ZET and mammalian results are compared, it is not clear why they are discordant. Assuming that camphor was bioavailable, species-specific metabolism may have caused differences in internal exposure and thus in results, which is supported by a review that identified different metabolites formed by mammalian species (EFSA, 2008). In addition, given that the general biotransformation capacity of zebrafish embryos is still a matter of debate (de Souza Anselmo *et al.*, 2018; Saad *et al.*, 2017), the zebrafish embryo, in contrast to mammals, may not be able to metabolize camphor at all.

The second chemical, fluazinam, was positive in a rat prenatal developmental toxicity study due to an increased number of skeletal malformations and retarded growth at the lowest maternally toxic dose (Tesh *et al.*, 1992). This positive result was confirmed by several unpublished rat and rabbit prenatal developmental toxicity studies, which are summarized in a classification and labeling proposal under the REACH regulation (Anonymous, 2011). In the only ZET study, fluazinam significantly induced mortality at 0.64 and 64 µM (but not at 6.4 µM), but did not induce any other effects in a statistically significant or clearly concentration-dependent manner (Truong *et al.*, 2014). Therefore, it was considered negative. A more recent ZET study showed that fluazinam started to be lethal at 0.3 µM at 96 hpf, killing all embryos at 0.7 µM, and to induce deformities in the same concentration range (Wang *et al.*, 2018). Despite several differences between the 2 ZET studies, such as the zebrafish strain used, the concentration used and the exposure duration, it seems that fluazinam acts through a general (systemic) mechanism and is not specifically embryotoxic, but may induce embryotoxic effects secondary to general effects. Prenatal developmental effects and systemic effects, as determined by maternal toxicity, are also induced by similar doses in rats and rabbits. The level of standardization of mammalian effect interpretation and the nature and severity of effects observed with fluazinam, led to an interpretation as positive for mammalian tests. The case of fluazinam shows that although differences in the interpretation of effects may explain discordant results between ZET and mammalian tests, there may be other explanations, such as species differences in transformations (hydrolysis and metabolism) or toxicological mechanisms.

Both examples demonstrate that even when data complexity is reduced to dichotomous results through an unambiguous and transparent interpretation, reasons for discordance of results can be manifold. This applies to the concordance of ZET and mammalian results, as well as for the concordance of ZET results from different studies.

In this context, it is important to recall that we are ultimately interested in the potential of a chemical to induce prenatal developmental effects in humans that both the ZET and the mammalian tests attempt to predict. We did not include human evidence in our review, however, primarily because we expected that conclusive human evidence would be available for only a limited number of substances (Clements *et al.*, 2020). Indeed, the lack of reliable human data and the largely unknown relevance of animal prenatal developmental toxicity data for humans are major obstacles to the assessment of the value of new approaches to measuring prenatal developmental toxicity, such as the ZET. This issue is not unique to developmental toxicity. It applies to many, if not all toxicological human health effects, and has been discussed in the broader context of references for the comparison of test methods and strategies (Hoffmann *et al.*, 2008). Strategies for shifting toxicology from a strong reliance on animal data to a more human-relevant and mechanism-based discipline are being proposed and discussed, but require time, resources, and some points of reference to establish confidence (Scialli *et al.*, 2018).

Regarding the methodological challenges of applying systematic review methods to toxicological test method assessment, the conclusions and recommendations of the preparatory study have been confirmed in this full systematic review. Stephens *et al.* (2019) concluded that the application of systematic review methods to toxicological test method assessment is in principle feasible. However, numerous challenges need to be considered in planning and conducting such a review. In retrospect, the most fundamental are the following.

##### Scoping

The importance of an interdisciplinary review team that covers all needed expertise, especially when adapting systematic review methods to new toxicological or other environmental health applications is stressed. Given that the application of systematic review methods to toxicology is relatively new, the review team should dedicate the necessary time for toxicology domain experts to educate systematic review experts and vice versa. This should take place in the project planning phase in order to optimally scope and frame the review and to understand the requirements and implications of each step in the review. Although we engaged in this process, we nevertheless encountered some challenges, in particular total amount of potentially relevant evidence and its heterogeneity.

##### Efficiency

In the future, broad review questions, which are required when comprehensive test method comparisons are undertaken, can be expected to be addressed more efficiently with the help of artificial intelligence tools. Although we applied such tools to aid title and abstract screening, tools supporting the review steps of full-text screening, data extraction, and critical appraisal would be of great help. To maximize the potential of artificial intelligence approaches for systematic reviews in toxicology and environmental health, a fundamental change in the reporting of research is needed. Common ontologies, annotations, and other approaches should be employed to improve the ability of computers to read and process the research literature (Whaley *et al.*, 2020). A key to that change is to increase researchers’ awareness of the importance of reporting completeness, which has also been called for in the context of improving reproducibility (Percie du Sert *et al.*, 2020). This change can only be brought about through a combination of efforts, including the appropriate education and training of researchers and the creation of incentives by scientific journals and research funders.

#### Critical Appraisal

Improved reporting would also facilitate the critical appraisal of studies. In our review, the RoB of approximately 75% of all studies could not be assessed due to inadequate reporting. Although better reporting would help to assess RoB, reducing such bias in future studies will demand more focused efforts. Assuming that poor reporting originates from a fundamental lack of awareness of biases that have the potential to lead to overestimation of effects, education and training of researchers could gradually lead to well-planned, conducted, and reported experimental studies that reduce or eliminate sources of bias.

The concept of study sensitivity, defined as a measure of the ability of a study to detect a true effect or hazard (Cooper *et al.*, 2016), to address important study aspects that would not be identified by a RoB assessment was particularly helpful. A more systematic and empirical exploration of this concept focused on comprehensiveness, applicability, and operationalization has the potential to facilitate and optimize systematic review approaches in environmental health and toxicology.

The application of systematic review approaches to the comparison of 2 toxicological test methods addressing the prenatal developmental effects of chemicals led us to identify contextual and methodological challenges in a transparent and objective manner. One key to overcoming these challenges is a fundamental change in how toxicological studies are planned, conducted, and reported. The first step toward bringing about this change is to create a broad awareness in the toxicological community of the urgent need for and benefits of more evidence-based approaches. This will provide the basis for creating a momentum in the community—from scientists to regulatory agencies and policymakers—to invest in the efforts needed.

We are confident that systematic review methodology will help advance the assessment of toxicological test methods, elucidating their strength and weaknesses in an evidence-based manner. It offers the flexibility to focus on various aspects of test method assessment, such as mechanistic relevance, reproducibility, predictivity, and aspects of applicability. However, advances in adjusting the review methodology for this purpose are required.

## SUPPLEMENTARY DATA

Supplementary data are available at *Toxicological Sciences* online.

## Supplementary Material

kfab072_Supplementary_Data
