# Supplementary material for: A Systematic Review to Compare Chemical Hazard Predictions of the Zebrafish Embryotoxicity Test With Mammalian Prenatal Developmental Toxicity
Source: Toxicol Sci. 2021 Jun 9;183(1):14–35. doi: 10.1093/toxsci/kfab072 (PMC8404989; doi:10.1093/toxsci/kfab072)
Supplement: kfab072_Supplementary_Data [file kfab072_supplementary_data.zip › toxsci-21-0054-File003.docx]

**Supplemental Material 1: Zebrafish Toxicity Searches**

**A systematic review to compare chemical hazard predictions of the zebrafish embryotoxicity test with mammalian prenatal developmental toxicity**

Sebastian Hoffmann^1,2*^, Bianca Marigliani^3^, Sevcan Gül Akgün-Ölmez^4^, Danielle Ireland^5^, Rebecca Cruz^6^, Francois Busquet^7^, Burkhard Flick^8^, Manoj Lalu^9^, Elizabeth C. Ghandakly^10^, Rob B.M. de Vries^1,11^, Hilda Witters^12^, Robert A. Wright^13^, Metin Ölmez^14^, Catherine Willett^15^, Thomas Hartung^16^, Martin L. Stephens^1^, Katya Tsaioun^1^

^1^ Evidence-Based Toxicology Collaboration (EBTC), Johns Hopkins Bloomberg School of Public Health, Baltimore, Maryland, 21205 (https://orcid.org/0000-0002-3214-7678)

^2^ seh consulting + services, 33106 Paderborn, Germany

^3^ Department of Science and Technology, Federal University of São Paulo (UNIFESP), São José dos Campos, São Paulo, Brazil (https://orcid.org/0000-0002-0498-4284)

^4^ Department of Pharmaceutical Toxicology, Faculty of Pharmacy, Marmara University, 34722 Istanbul, Turkey

^5^ Swarthmore College, Swarthmore, Pennsylvania 19081 (https://orcid.org/0000-0002-9827-1604)

^6^ Laboratory of Dental Clinical Research, Universidade Federal Fluminense, Niterói, RJ, Brazil

^7^ Altertox, Brussels, Belgium

^8^ Experimental Toxicology and Ecology, BASF SE, 67063 Ludwigshafen am Rhein, Germany

^9^ Department of Anesthesiology and Pain Medicine, Ottawa Hospital Research Institute,

Ottawa, Canada

^10^ Berman Institute of Bioethics, Johns Hopkins University, Baltimore, Maryland, 21205

^11^ Systematic Review Centre for Laboratory Experimentation (SYRCLE), Department for Health Evidence, Radboud Institute for Health Sciences, Radboudumc, Nijmegen, The Netherlands

^12^ VITO NV, 2400 Mol, Belgium (https://orcid.org/0000-0002-2026-3962)

^13^ William H. Welch Medical Library, Johns Hopkins University, Baltimore, Maryland 21205

^14^ Umraniye Family Health Center (No. 44), Turkish Ministry of Health, Istanbul, Turkey

^15^ Humane Society International, Washington, DC

^16^ Center for Alternatives to Animal Testing (CAAT), Johns Hopkins Bloomberg School of Public Health, Baltimore, Maryland 21205

*Corresponding author: [sebastian.hoffmann@seh-cs.com](mailto:sebastian.hoffmann@seh-cs.com)

In addition, the reference databases of the various selection steps will be provided openly accessible on zenodo.org.

**Zebrafish Toxicity Searches for All Databases (for the Definitive Study) – Ran 6/23/16**

**BIOSIS**

TS=(zebrafish* OR "zebra fish" OR "zebra fishes" OR "Danio rerio" OR "zebra danio" OR "Brachydanio rerio")

**AND**

TS=(embryo* OR hpf OR h.p.f. OR "hours post fertilization" OR larva* OR egg* OR zygot* OR "maternal exposure" OR "maternal exposures")

**AND**

TS=(ZET OR ZFET OR toxi* OR neurotoxi* OR cardiotoxi* OR embryotoxi* OR "embryo test" OR "embryo tests" OR "embryo testing" OR "embryonic test" OR "embryonic tests" OR "embryonic testing" OR terato* OR "drug induced" OR malform*) OR (TS=(drug* OR chemical* OR pharmaceuti* OR contaminant* OR pollutant*) AND TS=(bioassay* OR assay* OR "high-throughput" OR screen* OR librar* OR automat* OR "well plate" OR "well plates" OR array* OR test* OR expos*))

**4,856 results, ran 6/23/16**

**Embase**

'zebra fish'/exp OR ((zebrafish* OR 'zebra fish' OR 'zebra fishes' OR 'Danio rerio' OR 'zebra danio' OR 'Brachydanio rerio'):ab,ti,de,lnk)

**AND**

'embryo'/exp OR 'embryo development'/exp OR 'embryo (anatomy)'/exp OR 'embryo death'/exp OR 'embryo growth'/exp OR 'embryo mortality'/exp OR 'embryo research'/exp OR 'embryology'/exp OR 'larva'/exp OR 'egg'/exp OR 'zygote'/exp OR 'animal embryo'/de OR ((embryo* OR hpf OR 'h.p.f.' OR 'hours post fertilization' OR larva* OR egg* OR zygot* OR 'maternal exposure' OR 'maternal exposures'):ab,ti,de,lnk)

**AND**

'toxicity testing'/exp OR 'toxicological parameters'/exp OR 'developmental toxicity'/exp OR 'toxicity'/exp OR 'teratogenic agent'/exp OR 'teratogen testing'/exp OR 'teratogenesis'/exp OR 'congenital malformation'/exp OR ((ZET OR ZFET OR toxi* OR neurotoxi* OR cardiotoxi* OR embryotoxi* OR 'embryo test' OR 'embryo tests' OR 'embryo testing' OR 'embryonic test' OR 'embryonic tests' OR 'embryonic testing' OR terato* OR 'drug induced' OR malform*):ab,ti,de,lnk) OR (((drug* OR chemical* OR pharmaceuti* OR contaminant* OR pollutant*):ab,ti,de,lnk) AND ('bioassay'/exp OR 'high throughput screening'/exp OR 'molecular library'/exp OR ((bioassay* OR assay* OR 'high-throughput' OR screen* OR librar* OR automat* OR 'well plate' OR 'well plates' OR array* OR test OR tests OR tested OR testing OR expos*):ab,ti,de,lnk)))

**5,840 results, ran 6/23/16**

**PubMed**

"Zebrafish"[Mesh] OR zebrafish*[tw] OR zebra fish*[tw] OR Danio rerio[tw] OR zebra danio[tw] OR Brachydanio rerio[tw]

**AND**

"Embryo, Nonmammalian"[Mesh] OR "embryology"[Subheading] OR "Larva"[Mesh] OR "Eggs"[Mesh] OR "Zygote"[Mesh] OR "Maternal Exposure"[Mesh] OR embryo*[tw] OR hpf[tw] OR h.p.f.[tw] OR hours post fertilization[tw] OR larva*[tw] OR egg*[tw] OR zygot*[tw] OR maternal exposure*[tw]

**AND**

"Toxicity Tests"[Mesh] OR "toxicity"[Subheading] OR "Teratogens"[Mesh] OR "Teratogenesis"[Mesh] OR "Abnormalities, Drug-Induced"[Mesh] OR ZET[tw] OR ZFET[tw] OR toxic*[tw] OR toxin*[tw] OR neurotoxi*[tw] OR cardiotoxi*[tw] OR embryotoxi*[tw] OR embryo test*[tw] OR embryonic test*[tw] OR terato*[tw] OR drug induc*[tw] OR malform*[tw] OR ((drug*[tw] OR chemical*[tw] OR pharmaceuti*[tw] OR contaminant*[tw] OR pollutant*[tw]) AND ("Biological Assay"[Mesh] OR "High-Throughput Screening Assays"[Mesh] OR "Small Molecule Libraries"[Mesh] OR bioassay*[tw] OR assay*[tw] OR "high-throughput"[tw] OR screen*[tw] OR librar*[tw] OR automat*[tw] OR "well plate"[tw] OR "well plates"[tw] OR array*[tw] OR test[tw] OR tests[tw] OR tested[tw] OR testing*[tw] OR expos*[tw]))

**3,743 results, ran 6/23/16**

**TOXLINE**

Zebrafish OR "zebra fish" OR "Danio rerio" OR "zebra danio" OR "Brachydanio rerio"

**AND**

Embryo OR embryoes OR embryonic OR embryology OR hpf OR h.p.f. OR "hours post fertilization" OR larva* OR egg OR zygot* OR "maternal exposure" OR "maternal exposures"

**AND**

"Toxicity test" OR ZET OR ZFET OR toxicity OR toxic OR toxicant OR toxicology OR toxicological* OR toxin OR neurotoxicology OR neurotoxicological* OR neurotoxicity OR neurotoxic

**OR**

neurotoxin OR cardiotoxi* OR embryotoxi* OR "embryo test" OR "embryo testing" OR "embryonic test" OR "embryonic testing" OR teratogen OR teratogenesis OR teratogeny

**OR**

teratogenic* OR teratogenicity OR teratological* OR teratology OR teratopharmacological* OR "drug induced" OR malform*

**OR**

((drug OR chemical* OR pharmaceuti* OR contaminant* OR pollutant*) AND ("biological assay" OR bioassay* OR assay* OR "high-throughput" OR screen OR screened OR screening OR librar* OR automat* OR "well plate" OR array* OR test OR tested OR testing OR expose* OR exposing OR exposure))

**3,051 results, ran 6/23/16**
