# Supplementary material for: A Systematic Review to Compare Chemical Hazard Predictions of the Zebrafish Embryotoxicity Test With Mammalian Prenatal Developmental Toxicity
Source: Toxicol Sci. 2021 Jun 9;183(1):14–35. doi: 10.1093/toxsci/kfab072 (PMC8404989; doi:10.1093/toxsci/kfab072)
Supplement: kfab072_Supplementary_Data [file kfab072_supplementary_data.zip › toxsci-21-0054-File004.docx]

**Supplemental Material 2: Final Mammalian BIOSIS Search Strategy**

**A systematic review to compare chemical hazard predictions of the zebrafish embryotoxicity test with mammalian prenatal developmental toxicity**

Sebastian Hoffmann^1,2*^, Bianca Marigliani^3^, Sevcan Gül Akgün-Ölmez^4^, Danielle Ireland^5^, Rebecca Cruz^6^, Francois Busquet^7^, Burkhard Flick^8^, Manoj Lalu^9^, Elizabeth C. Ghandakly^10^, Rob B.M. de Vries^1,11^, Hilda Witters^12^, Robert A. Wright^13^, Metin Ölmez^14^, Catherine Willett^15^, Thomas Hartung^16^, Martin L. Stephens^1^, Katya Tsaioun^1^

^1^ Evidence-Based Toxicology Collaboration (EBTC), Johns Hopkins Bloomberg School of Public Health, Baltimore, Maryland, 21205 (https://orcid.org/0000-0002-3214-7678)

^2^ seh consulting + services, 33106 Paderborn, Germany

^3^ Department of Science and Technology, Federal University of São Paulo (UNIFESP), São José dos Campos, São Paulo, Brazil (https://orcid.org/0000-0002-0498-4284)

^4^ Department of Pharmaceutical Toxicology, Faculty of Pharmacy, Marmara University, 34722 Istanbul, Turkey

^5^ Swarthmore College, Swarthmore, Pennsylvania 19081 (https://orcid.org/0000-0002-9827-1604)

^6^ Laboratory of Dental Clinical Research, Universidade Federal Fluminense, Niterói, RJ, Brazil

^7^ Altertox, Brussels, Belgium

^8^ Experimental Toxicology and Ecology, BASF SE, 67063 Ludwigshafen am Rhein, Germany

^9^ Department of Anesthesiology and Pain Medicine, Ottawa Hospital Research Institute,

Ottawa, Canada

^10^ Berman Institute of Bioethics, Johns Hopkins University, Baltimore, Maryland, 21205

^11^ Systematic Review Centre for Laboratory Experimentation (SYRCLE), Department for Health Evidence, Radboud Institute for Health Sciences, Radboudumc, Nijmegen, The Netherlands

^12^ VITO NV, 2400 Mol, Belgium (https://orcid.org/0000-0002-2026-3962)

^13^ William H. Welch Medical Library, Johns Hopkins University, Baltimore, Maryland 21205

^14^ Umraniye Family Health Center (No. 44), Turkish Ministry of Health, Istanbul, Turkey

^15^ Humane Society International, Washington, DC

^16^ Center for Alternatives to Animal Testing (CAAT), Johns Hopkins Bloomberg School of Public Health, Baltimore, Maryland 21205

*Corresponding author: [sebastian.hoffmann@seh-cs.com](mailto:sebastian.hoffmann@seh-cs.com)

In addition, the reference databases of the various selection steps will be provided openly accessible on zenodo.org.

**Final Mammalian BIOSIS Search Strategy**

TS=(Rattus OR "Sprague-Dawley" OR Wistar OR "Long-Evans" OR rat OR rats OR Oryctolagus OR rabbit OR rabbits)

**AND**

TS=(embryo* OR fetus* OR foetus* OR fetal* OR foetal* OR "ductus arteriosus" OR "endocardial cushion*" OR "atrioventricular canal cushion*" OR "truncus arteriosus" OR organogenesis OR mother* OR dam OR dams OR maternal* OR pregnancy OR pregnancies OR pregnant OR congenital* OR prenatal* OR "pre-natal" OR "pre-natally" OR intrauterine OR "intra-uterine" OR antenatal* OR gestation* OR "transplacental exposure*" OR (("organ development" OR "tooth development" OR "tooth formation" OR "tooth growth" OR "tooth calcification" OR "tooth mineralization" OR "dental development" OR "dental formation" OR cementogenesis OR cementification OR "cementum formation" OR dentinogenesis OR dentinogeneses OR dentification OR "dentin formation" OR dentogenesis OR odontogenesis OR odontogeneses OR amelogenesis OR amelogeneses OR "enamel formation" OR lymphangiogenesis OR lymphangiogeneses OR "musculoskeletal development" OR "musculoskeletal system development" OR "limb development" OR "bone development" OR "bone growth" OR "physiologic calcification" OR "physiological calcification" OR "bone mineralization" OR "maxillofacial development" OR "craniofacial development" OR "face development" OR "facial development" OR "skull development" OR "cranial development" OR "skull growth" OR osteogenesis OR osteogeneses OR "bone formation" OR ossification OR osteoclastogenesis OR osteoclastogeneses OR chondrogenesis OR chondrogeneses OR "muscle development" OR "muscular development" OR myogenesis OR myogeneses OR myofibrillogenesis OR myofibrillogeneses OR neurogenesis OR neurogeneses OR "nervous system development" OR "neurologic development" OR "brain development" OR "brain cortex development" OR "cerebral development" OR "brain maturation" OR "brain maturity" OR "eye development" OR "retina development" OR "sex differentiation" OR "sexual differentiation" OR "gonad development" OR "gonadal development" OR "gonad differentiation" OR "gonadal differentiation" OR "sex gland development" OR "sexual gland development" OR "ovary development" OR "ovarian development" OR "ovary maturation" OR "ovarian maturation" OR "follicle development" OR folliculogenesis OR "ovary follicle formation" OR "ovarian follicle formation" OR "follicle maturation" OR "testis development" OR "testicle development" OR "testicular development" OR "testis descent" OR "testicle descent" OR "testicular descent" OR "descensus testiculorum" OR "descensus testis" OR "testis descensus" OR "heart development" OR "cardiac development" OR "heart growth" OR "kidney development" OR "kidney growth" OR "renal development" OR "renal growth" OR "liver development" OR "hepatic development" OR "lung development" OR "pulmonary development") AND (embryo* OR fetus* OR foetus* OR fetal* OR foetal* OR congenital* OR prenatal* OR "pre-natal" OR "pre-natally" OR intrauterine OR "intra-uterine" OR antenatal* OR gestation*)))

**AND**

TS=(toxic* OR neurotoxi* OR hormesis OR hormeses OR hormetic OR "lethal dos*" OR "fatal dos*" OR LD10 OR LD100 OR LD50 OR L.D.50 OR "LD 50" OR LD90 OR LD95 OR LD99 OR "sublethal dos*" OR "inhibitory concentration 50" OR IC50 OR "IC-50" OR "50% inhibitory concentration" OR "inhibitory concentration 50%" OR "half maximal inhibitory concentration" OR "half maximum inhibitory concentration" OR "median inhibitory concentration" OR "maximum tolerated dos*" OR "maximally tolerated dos*" OR "maximal tolerated dos*" OR "maximum tolerable dos*" OR "maximal tolerable dos*" OR "maximally tolerable dos*" OR "maximum permissible dos*" OR "maximal permissible dos*" OR "maximum permissible exposure level*" OR "permissible level*" OR "permissible limit*" OR "maximum dos*" OR "maximal dos*" OR "no-observed-adverse-effect level*" OR "no-observed-adverse-effects level*" OR NOAEL OR NOAELs OR "no-observed-effect level*" OR "no-observed-effects level*" OR "no observable effect level*" OR "no observable effects level*" OR "no effect dose level*" OR "non-observed effect dose level*" OR "non-observed-effect level*" OR "no-observed-adverse-event level*" OR "no-observed-adverse-events level" OR "no-observed-adverse-events levels" OR "no-observable-adverse-event level" OR "no-observable-adverse-event levels" OR "no-observable-adverse-events level" OR "no-observable-adverse-events levels" OR "protective index*" OR "protective indices" OR "therapeutic index*" OR "therapeutic indices" OR "safety window*" OR "therapeutic ratio" OR "therapeutic ratios" OR "therapeutic window*" OR "therapeutic drug index*" OR "therapeutic drug indices" OR "therapeutic drug window*" OR "toxic dos*" OR TD50 OR "body burden*" OR "drug residue*" OR "pesticide residue*" OR poison* OR teratogen* OR teratolog* OR teratomorph* OR teratotoxic* OR embryotoxi* OR fetotoxi* OR dysmorpholog* OR "drug-induced" OR malform* OR (("OECD guideline*" OR "OECD testing guideline*" OR "test guideline*") AND 414) OR "guideline 414" OR "guideline no. 414" OR "guideline number 414" OR "OECD no. 414" OR "OECD number 414" OR "OECD 414" OR "TG 414")

**AND**

**Part 1**

TS=(**"alitretinoin"** OR "1UA8E65KDZ" OR "5300-03-8" OR "(9cis)-retinoic acid" OR "[3H]9-cis-retinoic acid" OR "9(Z)-Retinoic acid" OR "9-(Z)-retinoic Acid" OR "9-cis-RA" OR "9-cis-Retinoate" OR "9-cis-Retinoic acid" OR "9-cis-Tretinoin" OR "9CRA" OR "9C-RA" OR "9-CRA" OR "9cRA compound" OR "9-Retinoate" OR "9-Retinoic acid" OR "agn 192013" OR "agn192013" OR "ALRT 1057" OR "ALRT1057" OR "BAL4079" OR "BAL-4079" OR "DB00523" OR "LG100057" OR "LG-100057" OR "LGD 100057" OR "LGD 1057" OR "lgd100057" OR "LGD1057" OR "nsc 659772" OR "nsc659772" OR "Panretin" OR "Panretyn" OR "Panrexin" OR "Ro-04-4079" OR "Toctino") OR TS=(**"Fluorouracil"** OR "U3P01618RT" OR "51-21-8" OR "2, 4 dioxo 5 fluoropyrimidine" OR "5 fluoro 2, 4 pyrimidinedione" OR "5 fluoropyrimidine 2, 4 dione" OR "5 fu" OR "5-Faracil" OR "5-florouracil" OR "5-Fluoracil" OR "5-Fluoracyl" OR "5-fluorouacil" OR "5-fluorourasil" OR "5-Fluracil" OR "5-Ftouracyl" OR "5FU" OR "5F-uracil" OR "5-HU Hexal" OR "accusite" OR "actino-hermal" OR "Adrucil" OR "agicil" OR "Arumel" OR "Carac" OR "Carzonal" OR "cinkef-u" OR "Effluderm" OR "Efudex" OR "Efudix" OR "Efurix" OR "eurofluor" OR "F 6627" OR "f6627" OR "fivoflu" OR "Fluoro Uracil" OR "Fluoroblastin" OR "Fluoroplex" OR "Fluorouracile" OR "Fluoro-uracile" OR "Fluorouracilo Ferrer Far" OR "Fluoruracil" OR "Fluouracil" OR "fluoxan" OR "flurablastin" OR "Fluracedyl" OR "Fluracil" OR "fluracilium" OR "Fluracilum" OR "Fluri" OR "Fluril" OR "Fluro Uracil" OR "Fluroblastin" OR "fluroblastine" OR "Flurodex" OR "Ftoruracil" OR "Haemato-FU" OR "ifacil" OR "Kecimeton" OR "Neofluor" OR "nsc 18913" OR "nsc18913" OR "NSC19893" OR "NSC-19893" OR "oncofu" OR "Onkofluor" OR "Phthoruracil" OR "Phtoruracil" OR "Queroplex" OR "Ribofluor" OR "ro2 9757" OR "Ro-29757" OR "Ro-2-9757" OR "Timazin" OR "Tolak" OR "uflahex" OR "utoral") OR TS=(**"Lovastatin"** OR "9LHU78OQFD" OR "75330-75-5" OR "6alpha-Methylcompactin" OR "6-alpha-Methylcompactin" OR "6-Methylcompactin" OR "Advicor" OR "Altocor" OR "Altoprev" OR "Artein" OR "Belvas" OR "birotin" OR "Cholestra" OR "cid_53232" OR "Closterol" OR "Colevix" OR "cysin" OR "DB00227" OR "ellanco" OR "elstatin" OR "Hipolip" OR "Hipovastin" OR "l 654969" OR "L-154803" OR "Lestatin" OR "Lipdip" OR "Lipivas" OR "Lipofren" OR "Liposcler" OR "lofacol" OR "lomar" OR "lostatin" OR "lovacel" OR "lovacol" OR "lovahexal" OR "Lovalip" OR "Lovalord" OR "lovastan" OR "Lovasterol" OR "Lovastin" OR "lovatadin" OR "lowachol" OR "Lozutin" OR "medostatin" OR "Mevacor" OR "meverstin" OR "Mevinacor" OR "Mevinolin" OR "Mevlor" OR "mk 0803" OR "mk0803" OR "MK803" OR "MK-803" OR "Monacolin K" OR "Monakolin K" OR "msd 803" OR "neolipid" OR "Nergadan" OR "ovasta" OR "Paschol" OR "Rextat" OR "Rodatin" OR "Rovacor" OR "Sivlor" OR "Statosan" OR "Taucor" OR "Tecnolip" OR "Teroltrat") OR TS=(**"mono-(2-ethylhexyl)phthalate"** OR "4376-20-9" OR "FU2EWB60RT" OR "(2 ethylhexyl) phthalate" OR "(2-Ethylhexyl) hydrogen phthalate" OR "2 ethylhexyl phthalate" OR "2 ethylhexylphthalate" OR "2-Ethylhexyl hydrogen phthalate" OR "MEHP" OR "mono (2 ethylhexyl) phthalate" OR "mono 2 ethylhexyl phthalate" OR "mono-(2-ethyl)hexyl phthalate" OR "Mono(2-ethylhexyl) phthalate" OR "Mono-(2-ethylhexyl) phthalate" OR "Mono(2-ethylhexyl)phthalate" OR "mono(ethylhexyl) phthalate" OR "mono-ethylhexyl" OR "Monoethylhexyl phthalate" OR "Monoethylhexyl phthalic acid" OR "monoethylhexylphthalate" OR "mono-ethylhexylphthalate" OR "phthalic acid 2 ethylhexyl ester" OR "phthalic acid 2 ethylhexyl monoester" OR "phthalic acid mono (2 ethylhexyl) ester" OR "Phthalic Acid Mono(2-ethylhexyl) Ester" OR "PHTHALIC ACID MONO-2-ETHYLHEXYL ESTER" OR "Phthalic acid mono-2-ethylhexylester" OR "Phthalic acid, mono-(2-ethylhexyl) ester" OR "Phthalic acid, mono-2-ethylhexyl ester" OR "Phthalic Acid-d4 Mono(2-ethylhexyl) Ester") OR TS=(**"Caffeine"** OR "3G6A5W338E" OR "58-08-2" OR "95789-13-2" OR "1, 3, 7 trimethyl 2, 6 dioxopurine" OR "1,3,7-Trimethyl-2,6-dioxopurine" OR "1,3,7-Trimethylpurine-2,6-dione" OR "1,3,7-trimethylxanthine" OR "1,7-Trimethyl-2,6-dioxopurine" OR "1-methyltheobromine" OR "1-methyl-Theobromine" OR "7-methyl Theophylline" OR "7-Methyltheophylline" OR "Alert-pep" OR "animine" OR "cafalgine" OR "Cafamil" OR "Cafecon" OR "Cafeina" OR "cafeine" OR "Cafergot" OR "Caffedrine" OR "Caffein" OR "Caffeina" OR "Caffeinum" OR "Caffine" OR "Cafipel" OR "coffein" OR "Coffeine" OR "Coffeinum" OR "Darvon compound-65" OR "Dasin" OR "Dexitac" OR "DHCplus" OR "Durvitan" OR "Eldiatric C" OR "Enerjets" OR "Ercatab" OR "Guaranine" OR "guarin" OR "Hycomine" OR "Kofein" OR "Koffein" OR "Lanorinal" OR "Mateina" OR "Methyltheobromide" OR "Methyltheobromine" OR "Methylxanthine theophylline" OR "Miudol" OR "Nix Nap" OR "no doz" OR "Nodaca" OR "nodoz" OR "nymusa" OR "Organex" OR "P-A-C Analgesic Tablets" OR "pac compound" OR "Pep-Back" OR "Percoffedrinol N" OR "Percutafeine" OR "peyona" OR "Phensal" OR "Propoxyphene Compound 65" OR "Quick Pep" OR "QuickPep" OR "Respia" OR "SK-65 Compound" OR "teina" OR "Theine" OR "Tirend" OR "trimethylxanthine" OR "Vivarin" OR "Wigraine") OR TS=(**"Busulfan"** OR "G1LN9045DK" OR "55-98-1" OR "1, 4 bis (methanesulfonyloxy) butane" OR "1, 4 butanediol dimethanesulfonate" OR "1, 4 dimethanesulfonyloxybutane" OR "1, 4 dimethylsulfonyloxybutane" OR "1,4-Bis(methanesulfonoxy)butane" OR "1,4-Bis(methanesulfonyloxy)butane" OR "1,4-Butanedi yl dimethanesulfonate" OR "1,4-BUTANEDIOL DIMETHANESULFONATE" OR "1,4-Butanediol dimethanesulphonate" OR "1,4-Butanediol dimethylsulfonate" OR "1,4-butanedioldimethanesulfonate" OR "1,4-Butanediyl dimethanesulfonate" OR "1,4-Di(methylsulfonoxy)butane" OR "1,4-Dimesyloxybutane" OR "1,4-Dimethane sulfonyl oxybutane" OR "1,4-Dimethanesulfonoxybutane" OR "1,4-Dimethanesulfonoxylbutane" OR "1,4-Dimethanesulfonyloxybutane" OR "1,4-Dimethanesulphonyloxybutane" OR "1,4-Dimethylsulfonoxybutane" OR "1,4-Dimethylsulfonyloxybutane" OR "Bisulfex" OR "Busilvex" OR "Busulfano" OR "Busulfanum" OR "busulfex" OR "busulphan" OR "Busulphane" OR "butane-1,4-diyl dimethanesulfonate" OR "Butanedioldimethanesulfonate" OR "Buzulfan" OR "citosulfan" OR "cytoleukon" OR "glyzophrol" OR "krn 246" OR "krn246" OR "Leucosulfan" OR "mablin" OR "Mielevcin" OR "Mielosan" OR "mielucin" OR "Milecitan" OR "Mileran" OR "misulban" OR "mitistan" OR "mitosan" OR "mitostan" OR "muleran" OR "myelenkon" OR "myeleran" OR "Myeleukon" OR "myeloleukon" OR "Myelosan" OR "Myelosanum" OR "myeloxan" OR "myelucin" OR "mylecitan" OR "Mylecytan" OR "Myleran" OR "Mylerlan" OR "n-Butane-1,3-di(methylsulfonate)" OR "nsc 750" OR "NSC750" OR "Sulfabutin" OR "Sulphabutin" OR "tetramethylene dimesylate") OR TS=(**"Warfarin"** OR "129-06-6" OR "5Q7ZVV76EI" OR "81-81-2" OR "1 (4' hydroxy 3' coumarinyl) 1 phenyl 3 butanone" OR "3 (alpha acetonylbenzyl) 4 hydroxycoumarin" OR "3 acetonylbenzonyl 4 hydroxy coumarinedimethylaminoethanol" OR "3 alpha phenyl beta acetylethyl 4 hydroxycoumarin" OR "3-(.alpha.-Acetonylbenzyl)-4-hydroxycoumarin" OR "3-(.alpha.-Phenyl-.beta.-acetylaethyl)-4-hydroxycumarin" OR "3-(.alpha.-Phenyl-.beta.-acetylethyl)-4-hydroxycoumarin" OR "3-(1'-Phenyl-2'-acetylethyl)-4-hydroxycoumarin" OR "3-(a-acetonylbenzyl)-4-hydroxycoumarin" OR "3-(Acetonylbenzyl)-4-hydroxycoumarin" OR "3-(alpha-Acetonylbenzyl)-4-hydroxycoumarin" OR "3-(alpha-Phenyl-beta-acetylaethyl)-4-hydroxycumarin" OR "3-(alpha-Phenyl-beta-acetylethyl)-4-hydroxycoumarin" OR "4-hydroxy-3-(3-oxo-1-phenylbutyl)-1-benzopyran-2-one" OR "4-Hydroxy-3-(3-oxo-1-phenylbutyl)-2H-1-benzopyran-2-one" OR "acetonylbenzylhydroxycoumarin" OR "adoisine" OR "Aldocumar" OR "alpha acetonylbenzyl 4 hydroxycoumarin dimethylaminoethanol" OR "antrombin k" OR "Athrombin" OR "athrombine k" OR "athrombinek" OR "befarin" OR "Brumolin" OR "carfin" OR "circuvit" OR "CO-Rax" OR "coumadan" OR "coumadin" OR "coumadine" OR "Coumafen" OR "coumafene" OR "Coumaphen" OR "coumaphene" OR "Coumefene" OR "Cov-R-Tox" OR "dagonal" OR "DB00682" OR "delta-con" OR "Dethmor" OR "Dethnel" OR "Dicusat E" OR "farin" OR "Frass-Ratron" OR "jantoven" OR "Kumader" OR "Kumadu" OR "kumatox" OR "Kypfarin" OR "maforan" OR "marevan" OR "Mar-Frin" OR "Maveran" OR "orfarin" OR "panwarfarin" OR "panwarfin" OR "Prothromadin" OR "Ratorex" OR "Ratox" OR "Ratoxin" OR "Ratron" OR "Rattunal" OR "Rodafarin" OR "Rosex" OR "Sewarin" OR "simarc-2" OR "Sofarin" OR "Solfarin" OR "Sorexa plus" OR "Tedicumar" OR "Temus W" OR "tintorane" OR "uniwarfin" OR "Vampirinip II" OR "Vampirinip iii" OR "wafarin" OR "waran" OR "Warf 10" OR "Warf 42" OR "Warfant" OR "warfar" OR "Warfarat" OR "Warfarina" OR "warfarine" OR "Warfarinum" OR "Warficide" OR "warfil 5" OR "warfilone" OR "warnerin" OR "Zoocoumarin") OR TS=(**"Ketoconazole"** OR "R9400W927I" OR "142128-59-4" OR "65277-42-1" OR "1 [4 [4 [ [2 (2, 4 dichlorophenyl) 2 (1h imidazol 1 ylmethyl) 1, 3 dioxolan 4 yl] methoxy] phenyl] 1 piperazinyl] ethanone" OR "1 [4 [4 [ [2 (2, 4 dichlorophenyl) 2 [ (1h imidazol 1 yl) methyl] 1, 3 dioxolan 4 yl] methoxy] phenyl] piperazin 1 yl] ethan 1 one" OR "1 acetyl 4 [4 [ [2 (2, 4 dichlorophenyl) 2 (1h imidazol 1 ylmethyl) 1, 3 dioxolan 4 yl] methoxy] phenyl] piperazine" OR "4 (4 acetylpiperazin 1 yl) alpha [2 (2, 4 dichlorophenyl) 2 imidazol 1 ylmethyl 1, 3 dioxolan 4 yl] anisole" OR "akorazol" OR "anfuhex" OR "antanazol" OR "beatoconazole" OR "bigazol" OR "cetonax" OR "comozol" OR "conazol" OR "cremosan" OR "daktagold" OR "dezoral" OR "dio 902" OR "dio902" OR "extina" OR "formyco" OR "fugen" OR "funazole tabs" OR "funet" OR "fungarest" OR "fungaway" OR "fungazol tabs" OR "fungiderm-k" OR "funginoc" OR "funginox tabs" OR "fungoral" OR "kenazol" OR "kenazole" OR "kesnazol" OR "ketazol" OR "ketocanazole" OR "keto-comp" OR "ketoconazol" OR "Ketoconazolum" OR "keto-crema" OR "ketoderm" OR "ketoisdin" OR "ketomed" OR "ketomicin" OR "ketomicol" OR "ketona" OR "keto-shampoo" OR "ketozal" OR "ketozol" OR "ketozole" OR "kezon" OR "konaturil" OR "Kuric" OR "kw 1414" OR "lusanoc" OR "micoral" OR "mizole" OR "mizoron" OR "mycofebrin" OR "nastil" OR "nazole" OR "neutrogena t/sal" OR "nisoral" OR "niz creme" OR "niz shampoo" OR "nizoral" OR "oxocanazole" OR "oxoconazole" OR "oxonazol" OR "panfungol" OR "pasalen" OR "picamic" OR "prenalon" OR "pristinex" OR "profungal" OR "r 41, 400" OR "R 41,400" OR "r 41400" OR "R41,400" OR "R41400" OR "sebizole" OR "sporium" OR "sporoxyl" OR "sporozol" OR "termizol" OR "terzolin" OR "triatop lotion" OR "Xolegel" OR "zoralin tabs" OR "zorinax") OR TS=(**"Hydroxyurea"** OR "X6Q56QN5QC" OR "127-07-1" OR "(HYDROXYCARBAMOYL)AMINYL" OR "1-oxidanylurea" OR "aminohydroxamic acid" OR "biosupressin" OR "carbamic acid oxime" OR "carbamide oxide" OR "Carbamohydroxamic acid" OR "Carbamohydroximic acid" OR "Carbamohydroxyamic acid" OR "Carbamoyl oxime" OR "Carbamyl hydroxamate" OR "Carbomohydroxamic acid" OR "Carrbamoyl Oxime" OR "Cytodrox" OR "droxia" OR "Hidrix" OR "Hidroxicarbamida" OR "hydab" OR "hydrea" OR "Hydreia" OR "hydrine" OR "Hydroxicarbamidum" OR "hydroxy carbamide" OR "hydroxy urea" OR "hydroxyaminomethanamide" OR "Hydroxycarbamid" OR "hydroxycarbamide" OR "Hydroxycarbamidum" OR "Hydroxycarbamine" OR "hydroxyl urea" OR "Hydroxylurea" OR "Hydura" OR "Hydurea" OR "Idrossicarbamide" OR "Litaler" OR "litalir" OR "mylocel" OR "N-(Aminocarbonyl) Hydroxyamine" OR "N-(Aminocarbonyl)hydroxylamine" OR "N-Carbamoylhydroxylamine" OR "neodrea" OR "nsc 32065" OR "NSC32065" OR "oncocarbide" OR "onco-carbide" OR "oxycarbamide" OR "oxyrea" OR "oxyurea" OR "siklos") OR TS=(**"Valproic Acid"** OR "614OI1Z5WI" OR "99-66-1" OR "2 propylpentanoate" OR "2 propylpentanoic acid" OR "2 propylvalerate sodium" OR "2 propylvaleric acid" OR "2, 2 dipropyl acetic acid" OR "2-propyl-Pentanoate" OR "2-Propylpentanoic Acid" OR "2-PROPYL-PENTANOIC ACID" OR "2-PropylpentanoicAcid" OR "2-Propylvaleric acid" OR "4-Heptanecarboxylic acid" OR "absenor" OR "Acidum valproicum" OR "alpha propylvalerate" OR "alpha propylvaleric acid" OR "apilepsin" OR "atemperator" OR "Avugane" OR "Baceca" OR "convulex" OR "Convulsofin" OR "delepsine" OR "depacon" OR "depakene" OR "depakin" OR "depakine" OR "Depakote" OR "depalept" OR "deprakine" OR "Deproic" OR "di n propylacetate" OR "di n propylacetic acid" OR "di-n-propyl acetic acid" OR "diplexil" OR "Dipropyl Acetate" OR "dipropyl acetic acid" OR "dipropylacetate" OR "dipropylacetatic acid" OR "dipropylacetic acid" OR "diprosin" OR "Divalproex" OR "Encorate" OR "Epical" OR "epilam" OR "epilex" OR "epilim" OR "episenta" OR "Epival" OR "ergenyl" OR "espa valept" OR "Eurekene" OR "everiden" OR "goilim" OR "hexaquin" OR "kw 6066 n" OR "labazene" OR "leptilan" OR "leptilanil" OR "micropakine" OR "mylproin" OR "myproic acid" OR "n dipropylacetic acid" OR "orfil" OR "orfiril" OR "orlept" OR "petilin" OR "Propylisopropylacetic Acid" OR "Propylvaleric acid" OR "propymal" OR "Savicol" OR "sodium 2 propylpentanoate" OR "sodium 2 propylvalerate" OR "sodium di n propyl acetate" OR "sodium di n propylacetate" OR "sodium dipropyl acetate" OR "sodium dipropylacetate" OR "sodium n dipropylacetate" OR "stavzor" OR "valberg pr" OR "valcote" OR "Valdisoval" OR "valepil" OR "valeptol" OR "valerin" OR "valhel pr" OR "valoin" OR "valpakine" OR "valparin" OR "valporal" OR "valprax" OR "valpro" OR "valproate" OR "valprodura" OR "valprosid" OR "valprotek" OR "valsup" OR "Vupral") OR TS=(**"Tretinoin"** OR "5688UTC01R" OR "302-79-4" OR "1 (8 carboxy 3, 7 dimethyl 1, 3, 5, 7 octatetraen 1 yl) 2, 6, 6 trimethyl 1 cyclohexene" OR "3, 7 dimethyl 9 (2, 6, 6 trimethyl 1 cyclohexen 1 yl) 2, 4, 6, 8 nonatetraenoic acid" OR "3, 7 dimethyl 9 (2, 6, 6 trimethyl 1 cyclohexen 1 yl) nona 2, 4, 6, 8 tetraen 1 oic acid" OR "3,7-Dimethyl-9-(2,6,6-trimethyl-1-cyclohexen-1-yl)-2,4,6,8-nonatetraenoic acid" OR "3,7-Dimethyl-9-(2,6,6-trimethyl-1-cyclohexene-1-yl)-2,4,6,8-nonatetraenoic acid" OR "3,7-dimethyl-9-(2,6,6-trimethyl-1-cyclohexenyl)nona-2,4,6,8-tetraenoic acid" OR "3,7-Dimethyl-9-(2,6,6-trimethylcyclohex-1-enyl)nona-2,4,6,8-all-trans-tetraenoic acid" OR "9-cis-RA" OR "Aberel" OR "Aberela" OR "acid a vit" OR "Acnavit" OR "Airol" OR "Aknefug" OR "Aknoten" OR "all-trans-Vitamin A1 acid" OR "alquingel" OR "alten" OR "altinac" OR "anhydroretinoic acid" OR "ar 623" OR "ar623" OR "atra" OR "atragen" OR "atralin" OR "avita" OR "avitcid" OR "Avitoin" OR "betarretin" OR "dermairol" OR "dermik a" OR "effederm" OR "epi aberel" OR "epiaberel" OR "eudyna" OR "facenol" OR "ilotycin-a" OR "locacid" OR "Nexret" OR "nsc 122758" OR "nsc122758" OR "prosome a cream" OR "reacel-a" OR "Refissa" OR "Renova" OR "Retacnyl" OR "retavit" OR "retiderma" OR "Retin A" OR "Retinoate" OR "Retinoic acid" OR "Retinova" OR "Retionic acid" OR "Retisol-A" OR "retrieve cream" OR "ro 01 5488" OR "ro 1 5488" OR "ro 15488" OR "ro015488" OR "ro15488" OR "stieva a" OR "stievaa" OR "tracne" OR "Trans-Retinoicacid" OR "trentin" OR "Tretin M" OR "Tretinoinum" OR "TRETINON" OR "Vesanoid" OR "Vitamin A acid" OR "vitinoin") OR TS=(**"Vitamin A"** OR "11103-57-4" OR "68-26-8" OR "3, 7 dimethyl 9 (2, 6, 6 trimethyl 1 cyclohexen 1 yl) 2, 4, 6, 8 nonatetraen 1 ol" OR "3, 7 dimethyl 9 (2, 6, 6 trimethyl 1 cyclohexenyl) 2, 4, 6, 8 nonatetraen 1 ol" OR "3,7-Dimethyl-9-(2,6,6-trimethyl-1-cyclchexen-1-yl)-2,4,6,8-nonatetraen-1-ol" OR "3,7-Dimethyl-9-(2,6,6-trimethyl-1-cyclohexen-1-yl)-2,4,6,8-nonate-traen-1-ol" OR "3,7-Dimethyl-9-(2,6,6-trimethyl-1-cyclohexen-1-yl)-2,4,6,8-nonatetraen-1-ol, (all-E)-" OR "3,7-Dimethyl-9-(2,6,6-trimethyl-1-cyclohexen-1-yl)-2,4,6,8-nonatetraen-1-ol, all (E)-" OR "3,7-Dimethyl-9-(2,6,6-trimethyl-1-cyclohexenyl)-2,4,6,8-nonatetraen-1-ol" OR "3,7-dimethyl-9-(2,6,6-trimethyl-1-cyclohexenyl)-nona-2,4,6,8-tetraen-1-ol" OR "a 313" OR "a mulsal" OR "a mulsin" OR "a mulsine" OR "a sol" OR "a vi pel" OR "a vitadit" OR "a vitan" OR "a313" OR "acrisina" OR "acrisine" OR "actifral a" OR "adatone" OR "Afaxin" OR "afaxine" OR "afilina" OR "afiline" OR "agiolan" OR "Agoncal" OR "alcovit a" OR "alfa monovite" OR "alfaergin" OR "alfaergine" OR "alfamin" OR "alfamine" OR "alfamonovit" OR "alfasir" OR "alfasole" OR "alfasterolo" OR "alfatar" OR "alfavena" OR "alfavene" OR "alfavitina" OR "alfavitine" OR "alfene" OR "alin" OR "all-trans-Retinyl alcohol" OR "Alphalin" OR "alphaline" OR "alphasterol" OR "amulsal" OR "A-Mulsal" OR "amulsin" OR "amulsine" OR "amulvit" OR "Anatola" OR "anavit" OR "Anti-infective vitamin" OR "Antixerophthalmic vitamin" OR "Aoral" OR "apexol" OR "Apostavit" OR "Aquasol A" OR "Aquasola" OR "Aquasynth" OR "arcavit A" OR "asol" OR "A-Sol" OR "asteril" OR "Atars" OR "aterapion" OR "Avibon" OR "avimin" OR "avimine" OR "avipel" OR "A-Vi-Pel" OR "avipur" OR "avitabiol" OR "avitadit" OR "avital" OR "avitaminum kolin" OR "avitan" OR "A-Vitan" OR "avitana" OR "avitane" OR "avite" OR "avitil" OR "avitina" OR "Avitol" OR "avogina" OR "avogine" OR "avoleum" OR "axerodina" OR "axerodine" OR "axerol" OR "axerophthol" OR "Axerophtholum" OR "axerophthylium" OR "bentavit a" OR "bentavite a" OR "biosterol" OR "biotan" OR "chivibit a" OR "Chocola A" OR "Cylasphere" OR "cytobiase" OR "dagravit a" OR "davitamon a" OR "difvitamin a" OR "Disatabs Tabs" OR "Dofsol" OR "Dohyfral A" OR "elageno a" OR "endo a" OR "envit a" OR "Epiteliol" OR "fletase" OR "gadeol" OR "gadol" OR "halivitan" OR "halivitane" OR "homagenets aoral" OR "Homagenets aorl" OR "hydrosol" OR "Hydrovit A" OR "ido a" OR "idratene" OR "inovitan a" OR "Lard Factor" OR "meditalfa" OR "mulsal a" OR "multamine" OR "oleovit a" OR "Oleovitamin A" OR "ophthalamin" OR "panvita" OR "Plivit A" OR "Prepalin" OR "prepaline" OR "preparato a" OR "primavit" OR "quotivit" OR "Retin-11,12-t2-ol (9CI)" OR "retinol" OR "Retinolo" OR "Retinolum" OR "Retinyl A" OR "retinyl alcohol" OR "Retrovitamin A" OR "ro a vit" OR "Rovimix A 500" OR "Sehkraft A" OR "Tegosphere VitA" OR "Testavol" OR "Thalasphere" OR "ucemine a" OR "vaconex" OR "Vaflol" OR "Vafol" OR "Veroftal" OR "viadenin" OR "vialpha" OR "Vi-Alpha" OR "viatate" OR "vidoma" OR "vitadone" OR "vitadral" OR "vitalen a" OR "vitalfa" OR "vitama" OR "Vitamin A1" OR "Vitamine A" OR "Vitaminum A" OR "vitaplex a" OR "vitapur a" OR "vitasan a" OR "Vitavel A" OR "vitpex" OR "Vogan" OR "wandervit a" OR "xerophthol" OR "Zinosan N") OR TS=(**"Aminopterin"** OR "JYB41CTM2Q" OR "54-62-6" OR "4 amino 4 deoxyfolic acid" OR "4 amino 4 desoxyfolic acid" OR "4 amino 9 methylpteroylglutamic acid" OR "4 aminofolic acid" OR "4 aminomethylpteroylglutamic acid" OR "4 aminopteroylglutamic acid" OR "4-Amino-4-deoxypteroylglutamate" OR "4-Aminofolate" OR "4-Aminopteroyl- glutamic acid" OR "4-Aminopteroyl-<R>glutamic acid" OR "4-Aminopteroylglutamate" OR "4-Aminopteroylglutamic acid" OR "4-Aminopteroyl-glutamic acid" OR "Aminopterine" OR "Aminopterinum" OR "Aminotrexate" OR "n [para [ (2, 4 diaminopterid 6 ylmethyl) amino] benzoyl] glutamic acid" OR "nsc 739" OR "NSC739" OR "Pteramina") OR TS=(**"Methotrexate"** OR "YL5FZ2Y5U1" OR "59-05-2" OR "4 amino 10 methylfolic acid" OR "4 amino 10 methylpteroylglutamic acid" OR "4 amino n10 methylpteroylglutamic acid" OR "4-Aminomethylpteroylglutamic acid" OR "4-amino-N(10)-methylpteroylglutamic acid" OR "4-Amino-N(sup 10)-methylpteroylglutamic acid" OR "4-Amino-N10-methylpteroyl-L-glutamic acid" OR "a methopterine" OR "Abitrexate" OR "amethopterin" OR "A-Methopterin" OR "amethopterine" OR "A-Methpterin" OR "ametopterine" OR "Antifolan" OR "Arbitrexate" OR "biotrexate" OR "Brimexate" OR "canceren" OR "CL 14377" OR "cl14377" OR "Emtexate" OR "emthexat" OR "emthexate" OR "emtrexate" OR "enthexate" OR "farmitrexat" OR "farmitrexate" OR "farmotrex" OR "Fauldexato" OR "Folex" OR "ifamet" OR "intradose MTX" OR "jylamvo" OR "Lantarel" OR "ledertrexate" OR "Lumexon" OR "maxtrex" OR "Medsatrexate" OR "Metatrexan" OR "metex" OR "methoblastin" OR "methohexate" OR "Methotextrate" OR "methotrate" OR "Methotrexat" OR "methotrexato" OR "Methotrexatum" OR "methoxtrexate" OR "methrotrexate" OR "Methylaminopterin" OR "methylaminopterine" OR "Methylaminopterinum" OR "meticil" OR "metoject" OR "Metolate" OR "metothrexate" OR "Metotressato" OR "metotrexat" OR "metotrexate" OR "metotrexin" OR "metrex" OR "Metrotex" OR "Mexate" OR "mpi 5004" OR "mpi5004" OR "MTX hydrate" OR "n [4 [ (2, 4 diamino 6 pteridylmethyl) methylamino] benzoyl] glutamic acid" OR "N-(4-(((2,4-DIAMINO-6-PTERIDINYL)METHYL)METHYLAMINO)BENZOYL)L-GLUTAMIC ACID" OR "N-(4-(((2,4-Diamino-6-pteridinyl)methyl)methylamino)benzoyl)-L-glutamicacid" OR "N-[4-[[(2,4-Diamino-6-pteridinyl)methyl] methylamino]benzoyl]-L-glutamic acid" OR "N-[4-[[(2,4-diamino-6-pteridinyl)methyl]methylamino]benzoyl]-L-glutamic acid" OR "N-Bismethylpteroylglutamic acid" OR "neotrexate" OR "nordimet" OR "novatrex" OR "nsc 740" OR "NSC740" OR "Otrexup" OR "rasuvo" OR "reumatrex" OR "Rheumatrex" OR "Texate" OR "texorate" OR "Tremetex" OR "trexall" OR "Trexeron" OR "Trixilem" OR "xaken" OR "Xatmep" OR "zexate") OR TS=(**"Phenytoin"** OR "6158TKW0C5" OR "57-41-0" OR "630-93-3" OR "5, 5 diphenyl 2, 4 imidazolidinedione" OR "5, 5 diphenylglycolylurea" OR "5, 5 diphenylhydantoin" OR "5, 5' diphenylhydantoin" OR "5, 5 diphenylimidazoline 2, 4 dione" OR "5,5-di(phenyl)imidazolidine-2,4-dione" OR "5,5-diphenyl hydantoin" OR "5,5-Diphenyl-2,4-imidazolidinedione" OR "5,5-Diphenylhydantoin" OR "5,5-Diphenylimidazolidin-2,4-dione" OR "5,5-diphenylimidazolidine-2,4-dione" OR "5,5-Diphenyl-imidazolidine-2,4-dione" OR "alepsin" OR "aleviatin" OR "antilepsin" OR "Antisacer" OR "Auranile" OR "cansoin" OR "Causoin" OR "Citrullamon" OR "Citrulliamon" OR "Comital" OR "Comitoina" OR "Convul" OR "cumatil" OR "Danten" OR "Dantinal" OR "dantoin" OR "Dantoinal" OR "Dantoine" OR "denyl" OR "Difenin" OR "difetoin" OR "differenin" OR "difhydan" OR "Dihycon" OR "dihydan" OR "di-hydan" OR "Dihydantoin" OR "Dilabid" OR "Dilantin" OR "Dilantine" OR "Dillantin" OR "dintoin" OR "dintoina" OR "Diphantoin" OR "diphantoine" OR "Diphedal" OR "diphedan" OR "Diphenat" OR "Diphenin" OR "Diphenine" OR "Diphentoin" OR "Diphentyn" OR "diphenyl hydantoin" OR "Diphenylan" OR "diphenyldantoin" OR "Diphenylhydantoin" OR "Diphenylhydatanoin" OR "diphenytoin" OR "Di-Phetine" OR "ditoin" OR "Ditoinate" OR "ditomed" OR "Elepsindon" OR "Enkelfel" OR "Epamin" OR "Epanutin" OR "Epdantoin" OR "Epdantoine simple" OR "Epelin" OR "Epifenyl" OR "Epihydan" OR "Epilan D" OR "Epilantin" OR "epileptin" OR "Epinat" OR "Episar" OR "Epised" OR "Epsolin" OR "Eptal" OR "Eptoin" OR "felantin" OR "fenantoin" OR "Fenidantoin s" OR "Fenigramon" OR "Fenitoin" OR "Fentoin" OR "Fenylepsin" OR "fenytoin" OR "Fenytoine" OR "Hidan" OR "hidanil" OR "Hidantal" OR "Hidantilo" OR "Hidantina" OR "Hidantomin" OR "Hindatal" OR "Hydantal" OR "Hydantin" OR "hydantinal" OR "Hydantoinal" OR "Hydantol" OR "Ictalis simple" OR "Idantoil" OR "Idantoin" OR "Iphenylhydantoin" OR "Kessodanten" OR "Labopal" OR "Lehydan" OR "lepitoin" OR "Lepsin" OR "Minetoin" OR "Neos-Hidantoina" OR "Neosidantoina" OR "neosidantoina" OR "Novantoina" OR "Novophenytoin" OR "Om hidantoina simple" OR "Om-Hydantoine" OR "Oxylan" OR "Phanantin" OR "Phanatine" OR "Phenatine" OR "Phenatoine" OR "Phenhydan" OR "phenhydane" OR "Phenhydanin" OR "phenilep" OR "Phenitoin" OR "Phentoin" OR "Phentytoin" OR "phenybin" OR "phenydan" OR "phenydantin" OR "phenytek" OR "phenytex" OR "Phenytoinum" OR "phenytonium" OR "pyoredol" OR "Ritmenal" OR "Saceril" OR "sanepil" OR "Silantin" OR "Sinergina" OR "Sodanthon" OR "Sodantoin" OR "sodanton" OR "Sodium Diphenylhydantoinate" OR "Solantin" OR "Solantoin" OR "solantyl" OR "Sylantoic" OR "Tacosal" OR "Thilophenyl" OR "Toin unicelles" OR "vasilcon" OR "Zentronal" OR "Zentropil") OR TS=(**"Ethylene Glycol"** OR "FC72KVT52F" OR "107-21-1" OR "1, 2 ethanediol" OR "1,2-dihydroxy ethane" OR "1,2-Dihydroxyethane" OR "1,2-Ethandiol" OR "1,2-ethane diol" OR "1,2-ethanediol" OR "1,2-ethyleneglycol" OR "2-hydroxyethanol" OR "ethan-1,2-diol" OR "Ethane-1,2-diol" OR "ethane-1.2-diol" OR "ethanediol" OR "ethyl glycol" OR "ethylen glycol" OR "Ethylene alcohol" OR "Ethylene dihydrate" OR "ethyleneglycol" OR "Ethylenglycol" OR "Etilenglicol" OR "etylene glycol" OR "Glycol alcohol" OR "glycol ethylene" OR "Hypodicarbonous acid" OR "Mono Ethylene Glycol" OR "monoethylene glycol") OR TS=(**"Thiotepa"** OR "905Z5W3GKH" OR "52-24-4" OR "AI3-24916" OR "AI324916" OR "Girostan" OR "Ledertepa" OR "methylenethiophosphoramide" OR "n, n', n'' triethylenethiophosphoramide" OR "NSC 6396" OR "nsc 6996" OR "NSC6396" OR "Oncotepa" OR "Oncothio-tepa" OR "oncotiotepa" OR "Phosphoric tri(ethyleneamide)" OR "Phosphorothioic acid triethylenetriamide" OR "PHOSPHOROTHIOIC TRI(ETHYLENEAMIDE)" OR "Stepa" OR "tepadina" OR "tespa" OR "Tespamin" OR "Tespamine" OR "thio tepa" OR "Thiofozil" OR "Thiophosphamide" OR "Thiophosphamidum" OR "Thioplex" OR "Thiotef" OR "Thio-Tep" OR "Thiotepum" OR "thiotriethylenephosphoramide" OR "Tifosyl" OR "tio tef" OR "Tiofosfamid" OR "Tiofosyl" OR "Tiofozil" OR "trethylenethiophosphoramide" OR "Tri(1-aziridinyl)phosphine sulfide" OR "Tri(aziridin-1-yl)phosphine sulfide" OR "Tri(ethyleneimino)thiophosphoramide" OR "Tri-1-aziridinylphosphine sulfide" OR "Triaziridinylphosphine sulfide" OR "triethylene thio phosphoramide" OR "triethylene thiophosphamide" OR "triethylene thiophosphoramide" OR "triethylenethiophosphamide" OR "Triethylenethiophosphoramide" OR "Triethylenethiophosphorotriamide" OR "triethylenethiophosphortriamide" OR "tris (1 azaridinyl) phosphine sulfide" OR "tris (1 aziridinyl) phosphine sulfide" OR "tris (1 aziridinyl) phosphine sulphide" OR "tris (1 aziridinyl) phosphinsulfide" OR "tris (1 aziridinyl) phosphinsulphide" OR "tris (ethylenimino) thiophosphate" OR "Tris(1-aziridinyl)phosphine sulfide" OR "Tris(1-aziridinyl)phosphine sulphide" OR "tris(1-aziridinyl)-sulfanylidenephosphorane" OR "tris(aziridin-1-yl)-sulfanylidenephosphorane" OR "Tris(aziridinyl)phosphine sulfide" OR "Tris(aziridinyl)-phosphine sulfide" OR "Tris(ethylenimino)thiophosphate") OR TS=(**"Atrazine"** OR "QJA9M5H4IM" OR "1912-24-9" OR "2 chloro 4 ethylamino 6 isopropylamino 1, 3, 5 triazine" OR "2 chloro 4 ethylamino 6 isopropylamino s triazine" OR "2-Chloro-4-(ethylamino)-6-(isopropylamino)-1,3,5-triazine" OR "2-Chloro-4-(ethylamino)-6-(isopropylamino)-s-triazine" OR "2-Chloro-4-(ethylamino)-6-(isopropylamino)triazine" OR "2-Chloro-4-(ethylamino)-6-[(prop-2-yl)amino]-1,3,5-triazine" OR "2-Chloro-4-ethylamineisopropylamine-s-triazine" OR "2-Chloro-4-ethylamino-6-isopropylamino-1,3,5-triazine" OR "2-Chloro-4-ethylamino-6-isopropylamino-s-triazine" OR "2-chloro-4-ethyl-amino-6-isopropylamino-s-triazine" OR "2-CHLORO-4-ETHYLAMINO-6-ISOPROPYLAMINO-sym-TRIAZINE" OR "6 chloro n2 ethyl n4 isopropyl 1, 3, 5 triazine 2, 4 diamine" OR "6-Chloro-N2-ethyl-N4-isopropyl-1,3,5-triazine-2,4-diamine" OR "Aatram" OR "Aatrex" OR "Actinite PK" OR "Akticon" OR "Aktikon" OR "Aktinit A" OR "Aktinit PK" OR "Aneldazin" OR "Argezin" OR "Atazinax" OR "Atraflow" OR "Atranex" OR "Atrasine" OR "Atrataf" OR "Atratol" OR "Atrazin" OR "atrazinus" OR "Atred" OR "Atrex" OR "Attrex" OR "Azinotox 500" OR "Azoprim" OR "Candex" OR "Ceasin 50" OR "Cekuzina-T" OR "Chromozin" OR "Crisamina" OR "Crisatrina" OR "Crisazina" OR "Crisazine" OR "Cyazin" OR "Cyazine" OR "Farmozine" OR "Fenamin" OR "Fenamine" OR "Fenatrol" OR "Fogard" OR "Gesamprim" OR "Gesaprim" OR "Gesaprin" OR "Gesoprim" OR "Griffex" OR "Herbatoxol" OR "Hungazin" OR "Inakor" OR "Laddock" OR "Maizina" OR "Mebazine" OR "Oleogesaprim" OR "Pitezin" OR "Primatol" OR "Primaze" OR "Primitol A" OR "Primoleo" OR "Radazin" OR "Radizin" OR "Radizine" OR "Strazine" OR "Triazine A 1294" OR "Vectal" OR "Weedex A" OR "Wonuk" OR "Zeapho" OR "Zeaphos" OR "Zeapos" OR "Zeazin" OR "Zeazine" OR "zeazint" OR "Zeopos") OR TS=(**"dinoseb"** OR "88-85-7" OR "2 (1 methylpropyl) 4, 6 dinitrophenol" OR "2 sec butyl 4, 6 dinitrophenol" OR "2-(1-Methylpropyl)-4,6-dinitrophenol" OR "2-(1-Methylpropyl)-4,6-dinitro-Phenol" OR "2-(sec-Butyl)-4,6-dinitrophenol" OR "2, 4 dinitro 6 sec butylphenol" OR "2,4-Dinitro-6-sec-butylphenol" OR "2,4-dinitro-6-sec-butyl-phenol" OR "2-[1-methylpropyl]-4,6-dinitrophenol" OR "2-sec-Butyl-4,6-dinitrophenol" OR "2-Sec-butyl-4,6-dinitro-Phenol" OR "4, 6 dinitro 2 (1 methylpropyl) phenol" OR "4, 6 dinitro 2 sec butylphenol" OR "4,6-Dinitro-2-(1-methyl-n-propyl)phenol" OR "4,6-Dinitro-2-(1-methyl-propyl)phenol" OR "4,6-Dinitro-2-sec-butylphenol" OR "4,6-Dinitro-o-sec-butylphenol" OR "6 sec butyl 2, 4 dinitrophenol" OR "6-sec-Butyl-2,4-dinitrophenol" OR "Aatox" OR "Aretit" OR "Basanite" OR "Blaartox" OR "Butaphene" OR "Caldon" OR "Chemsect" OR "Desicoil" OR "Dibutox" OR "Dinitrall" OR "Dinitrax" OR "dinitrobutylphenol" OR "Dinitro-ortho-sec-butyl phenol" OR "Dinitro-o-sec-butylphenol" OR "Dynanap" OR "Dytop" OR "Elgetol 318" OR "Gebutox" OR "Hivertox" OR "Ivosit" OR "Kiloseb" OR "Ladob" OR "Laseb" OR "Nitropone C" OR "Phenotan" OR "Premerg" OR "Premerge" OR "Sinox general" OR "Sparic" OR "Spurge" OR "Subitex" OR "Tubotox") OR TS=(**"fluazinam"** OR "0P91PCK33Q" OR "79622-59-6" OR "3-Chloro-N-(3-chloro-2,6-dinitro-4-(trifluoromethyl)phenyl)-5-(trifluoromethyl)-2-pyridinamine" OR "3-Chloro-N-(3-chloro-2,6-dinitro-4-(trifluoromethyl)phenyl)-5-(trifluoromethyl)pyridin-2-amine" OR "3-chloro-N-(3-chloro-2,6-dinitro-4-trifluoromethylphenyl)-5-trifluoromethyl-2-pyridinamine" OR "3-Chloro-N-(3-chloro-2,6-dinitro-4-trifluoromethylphenyl)-5-trifluoromethyl-2-pyridylamine" OR "3-chloro-N-[3-chloro-2,6-dinitro-4-(trifluoromethyl)phenyl]-5-(trifluoromethyl)-2-pyridinamine" OR "3-chloro-N-[3-chloro-2,6-dinitro-4-(trifluoromethyl)phenyl]-5-(trifluoromethyl)pyridin-2-amine" OR "Altima" OR "Fluaziname" OR "Mapro" OR "Sekoya" OR "Shirlan") OR TS=(**"systhane"** OR "B6T1JTM6KZ" OR "88671-89-0" OR "(R)-2-p-chlorophenyl-2-(1H-1,2,4-triazol-1-ylmethyl)hexanenitrile" OR ".alpha.-Butyl-.alpha.-(4-chlorophenyl)-1H-1,2,4-triazole-1-propanenitrile" OR "2 (4 chlorophenyl) 2 (1h 1, 2, 4 triazol 1 ylmethyl) hexanenitrile" OR "2-(4-chlorophenyl)-2-(1,2,4-triazol-1-ylmethyl)hexanenitrile" OR "2-(4-Chlorophenyl)-2-(1H-1,2,4-triazol-1-ylmethyl)hexanenitrile" OR "2-p-Chlorophenyl-2-(1H-1,2,4-triazol-1-ylmethyl)hexanenitrile" OR "alpha butyl alpha (4 chlorophenyl) 1h 1, 2, 4 triazole 1 propanenitrile" OR "alpha-Butyl-alpha-(4-chlorophenyl)-1H-1,2,4-triazole-1-propanenitrile" OR "alpha-n-butyl-alpha(4-chlorophenyl)-1H-1,2,4-triazole-1-propanenitrile" OR "alpha-n-butyl-alpha-(4-chlorophenyl)-1H-1,2,4-triazole-1-propanenitrile" OR "myclobutanil" OR "Synthane 12E") OR TS=(**"ochratoxin A"** OR "1779SX6LUY" OR "303-47-9" OR "ochratoxine a") OR TS=(**"spiroxamine"** OR "OUT5YHB7BO" OR "118134-30-8" OR "(8-tert-Butyl-1,4-dioxa-spiro[4.5]dec-2-ylmethyl)-ethyl-propyl-amine" OR "8-tert-butyl-1,4-dioxaspiro(4.5)decan-2-ylmethyl(ethyl)(propyl)amine") OR TS=(**"thiacloprid"** OR "DSV3A944A4" OR "111988-49-9" OR "(3-((6-Chloro-3-pyridinyl)methyl)-2-thiazolidinylidene)cyanamide" OR "[3 (6 chloro 3 pyridinylmethyl) 2 thiazolidinylidene] cyanamide" OR "[3 [ (6 chloro 3 pyridinyl) methyl] 2 thiazolidinylidene] cyanamide" OR "[3 [ (6 chloropyridin 3 yl) methyl] 1, 3 thiazolidin 2 ylidene] cyanamide" OR "[3-[(6-chloranylpyridin-3-yl)methyl]-1,3-thiazolidin-2-ylidene]cyanamide" OR "[3-[(6-chloro-3-pyridinyl)methyl]-2-thiazolidinylidene]cyanamide" OR "[3-[(6-chloro-3-pyridyl)methyl]thiazolidin-2-ylidene]cyanamide" OR "[3-[(6-chloropyridin-3-yl)methyl]-1,3-thiazolidin-2-ylidene]cyanamide" OR "{3-[(6-chloropyridin-3-yl)methyl]-1,3-thiazolidin-2-ylidene}cyanamide" OR "3-((6-chloro-3-pyridinyl)methyl)-2-thiazolidinylidene cyanamide" OR "thiaclopride") OR TS=(**"Thiram"** OR "0D771IS0FH" OR "137-26-8" OR "16c tetramethylthiuram disulfide" OR "Aapirol" OR "Aatiram" OR "Accel TMT" OR "Aceto TETD" OR "Akrochem TMTD" OR "Anles" OR "Arasan" OR "Atiram" OR "Aules" OR "Basultra" OR "Betoxin" OR "bis (dimethyl thiocarbamoyl) disulfide" OR "bis (dimethylthiocarbamoyl) disulfide" OR "Bis(dimethyl thiocarbamoyl)disulfide" OR "Bis(dimethylthiocarbamoyl) disulfide" OR "Bis(dimethylthiocarbamoyl) disulphide" OR "Bis(dimethylthiocarbamyl) disulfide" OR "bis[Dimethylthiocarbamyl] disulfide" OR "Cunitex" OR "Cyuram DS" OR "Delsan" OR "Ekagom TB" OR "Falitiram" OR "Fermide" OR "Fernacol" OR "Fernasan" OR "Fernide" OR "Formalsol" OR "Granuflo" OR "Hermal" OR "Hermat TMT" OR "Heryl" OR "Hexathir" OR "Kregasan" OR "Mercuram" OR "Methyl thiuramdisulfide" OR "Methyl tuads" OR "Methylthiuram disulfide" OR "Metiur" OR "Metiurac" OR "Nobecutan" OR "Nocceler TT" OR "Nomersan" OR "Normersan" OR "NSC 1771" OR "NSC1771" OR "Panoram 75" OR "Polyram ultra" OR "Pomarsol" OR "Pomasol" OR "puralin" OR "Radothiram" OR "Rezifilm" OR "rhenogran" OR "Robac TMT" OR "Sadoplon" OR "Spotrete" OR "Sranan-sf-X" OR "Teramethylthiuram disulfide" OR "Tersan" OR "Tersantetramethyldiurane sulfide" OR "tetra methyl thiuram disulfide" OR "tetramethyl tetramethylthiuram disulfide" OR "tetramethyl thiuram disulfide" OR "Tetramethyl thiuramdisulfide" OR "Tetramethyl thiurane disulfide" OR "Tetramethyl thiurane disulphide" OR "Tetramethyldiurane sulphite" OR "Tetramethylenethiuram disulfide" OR "Tetramethylenethiuram disulphide" OR "Tetramethylthiocarbamoyldisulphide" OR "Tetramethylthioperoxydicarbonic diamide" OR "Tetramethylthioramdisulfide" OR "Tetramethylthiouram disulfide" OR "Tetramethylthiuram" OR "tetramethylthiuramdisulfide" OR "tetramethylthiuramidisulfide" OR "Tetramethylthiuran disulphide" OR "Tetramethylthiurane disulfide" OR "Tetramethylthiurum disulfide" OR "Tetramethylthiurum disulphide" OR "Tetrapom" OR "Tetrasipton" OR "tetrathion" OR "tetrathione" OR "tetrathionine" OR "Tetrathiuram disulfide" OR "Tetrathiuram disulphide" OR "Thianosan" OR "Thillate" OR "Thimar" OR "Thimer" OR "thiosan" OR "Thioscabin" OR "Thiotex" OR "Thiotox" OR "Thiramad" OR "Thirampa" OR "Thiramum" OR "Thirasan" OR "Thiulin" OR "Thiulix" OR "Thiurad" OR "Thiuram" OR "Thiuramin" OR "thiuramyl" OR "Thylate" OR "Tiradin" OR "tiram" OR "tiramo" OR "Tirampa" OR "tiuramyl" OR "TMT Disulfide" OR "TMTD" OR "TMTDS" OR "Trametan" OR "Tridipam" OR "Tripomol" OR "tuad" OR "TUEX" OR "Tulisan" OR "Tutan" OR "Tyradin" OR "Vancide TM" OR "Vulcafor TMT" OR "Vulkacit TH" OR "Vulkazam S") OR TS=(**"triadimefon"** OR "43121-43-3" OR "1 (4 chlorophenoxy) 3, 3 dimethyl 1 (1, 2, 4 triazol 1 yl) 2 butanone" OR "1-(1,2,4-triazolyl)-1-(4-chlorophenoxy)-3,3-dimethylbutan-2-one" OR "1-(1,2,4-Triazoyl-1)-1-(4-chloro-phenoxy)-3,3-dimethylbutanone" OR "1-(4-chlorophenoxy)-3,3-dimethyl-1-(1,2,4-triazol-1-yl)butan-2-one" OR "1-(4-Chlorophenoxy)-3,3-dimethyl-1-(1,2,4-triazol-1-yl)-butan-2-one" OR "1-(4-Chlorophenoxy)-3,3-dimethyl-1-(1,2,4-triazol-1-yl)butanone" OR "1-(4-chlorophenoxy)-3,3-dimethyl-1-(1H-1,2,4-triazol-1-yl) butan-2-one" OR "1-(4-Chlorophenoxy)-3,3-dimethyl-1-(1H-1,2,4-triazol-1-yl)-2-butanone" OR "1-(4-chlorophenoxy)-3,3-dimethyl-1-(1H-1,2,4-triazol-1-yl)butan-2-one" OR "1-(4-Chlorophenoxy)-3,3-dimethyl-1-(1H-1,2,4-triazole -1-yl)-2-butanone" OR "1-(4-Chloro-phenoxy)-3,3-dimethyl-1-[1,2,4]triazol-1-yl-butan-2-one" OR "Acizol" OR "Adifon" OR "Amiral" OR "Azocene" OR "Bayleton" OR "Diametom B" OR "Fenxiunin" OR "Haleton" OR "Miltek" OR "Nurex" OR "Otria 25" OR "Rofon" OR "Tidifon" OR "Triadimefone" OR "Triadimeform" OR "triadimenol" OR "Tripinacloraz") OR TS=(**"flusilazole"** OR "F3WG2VVD87" OR "85509-19-9" OR "Benocarp" OR "bis (4 fluorophenyl) methyl (1, 2, 4 triazol 1 yl) silane" OR "bis(4-fluorophenyl)(methyl)(1H-1,2,4-triazol-1-ylmethyl)silane" OR "bis(4-fluorophenyl)-methyl-(1,2,4-triazol-1-ylmethyl)silane" OR "Bis(4-fluorophenyl)methyl(1H-1,2,4-triazol-1-ylmethyl)silane" OR "DPX 6573" OR "dpx h6573" OR "DPX-H 6573" OR "dpxh6573" OR "DPX-N 6573" OR "DPX-N6573" OR "Flusilazol" OR "Fluzilazol" OR "Nustar" OR "Olymp" OR "PPX-H6573") OR TS=(**"hexaconazole"** OR "SX9R3X1FQV" OR "79983-71-4" OR "2 (2, 4 dichlorophenyl) 1 (1h 1, 2, 4 triazol 1 yl) 2 hexanol" OR "2-(2,4-dichlorophenyl)-1-(1,2,4-triazol-1-yl)-2-hexanol" OR "2-(2,4-dichlorophenyl)-1-(1,2,4-triazol-1-yl)hexan-2-ol" OR "2-(2,4-dichlorophenyl)-1-(1H-1,2,4-triazol-1-yl)hexan-2-ol" OR "alpha butyl alpha (2, 4 dichlorophenyl) 1h 1, 2, 4 triazole 1 ethanol" OR "Canvil" OR "Chlortriafol" OR "Clortriafol" OR "Contaf" OR "Flowmax 5SC" OR "Hexaconazol" OR "Ranvil") OR TS=(**"propiconazole"** OR "142KW8TBSR" OR "60207-90-1" OR "1 [2 (2, 4 dichlorophenyl) 4 propyl 1, 3 dioxolan 2 ylmethyl] 1h 1, 2, 4 triazole" OR "1-((2-(2,4-Dichlorophenyl)-4-propyl-1,3-dioxolan-2-yl)methyl)-1H-1,2,4-triazole" OR "1-(2-(2,4-Dichlorophenyl)-4-propyl-1,3-dioxolan-2-yl)methyl-1H-1,2,4-triazole" OR "1-(2-(2,4-Dichlorophenyl)-4-propyl-1,3-dioxolan-2-ylmethyl)-1H-1,2,4-triazole" OR "1-[[2-(2,4-dichlorophenyl)-4-propyl-1,3-dioxolan-2-yl]methyl]-1,2,4-triazole" OR "1-[[2-(2,4-Dichlorophenyl)-4-propyl-1,3-dioxolan-2-yl]methyl]-1H-1,2,4-triazole" OR "1-[2-(2,4-Dichloro-phenyl)-4-propyl-[1,3]dioxolan-2-ylmethyl]-1H-[1,2,4]triazole" OR "1-[2-(2,4-dichlorophenyl)-4-propyl-1,3-dioxolan-2-ylmethyl]-1H-1,2,4-triazole" OR "1-{[2-(2,4-dichlorophenyl)-4-propyl-1,3-dioxolan-2-yl]methyl}-1H-1,2,4-triazole" OR "Bamper" OR "cga 64250" OR "cga64250" OR "Desmel" OR "Proconazole" OR "Propiconazol" OR "Propyconazol" OR "Wocosen" OR "Wocosin 50TK") OR TS=(**"Endosulfan"** OR "OKA6A6ZD4K" OR "115-29-7" OR "5, 6 bis (hydroxymethyl) 1, 2, 3, 4, 7, 7 hexachloronorbornene sulfite" OR "5, 6 bis (hydroxymethyl) 1, 2, 3, 4, 7, 7 hexachloronorbornenesulfite" OR "5, 6 bis (hydroxymethyl) hexachlorobicyclo [2.2.1] hept 2 ene sulfite" OR "5, 6 bis (hydroxymethyl) hexachloronorcamphene sulfite" OR "alpha endosulfane" OR "benzoepin" OR "beosit" OR "beta endosulfane" OR "chlorothiepine" OR "chlorthiapinum" OR "chlorthiepin" OR "Chlortiepin" OR "Crisulfan" OR "cyclodan" OR "devisulfan" OR "Devisulphan" OR "Endocel" OR "endogan" OR "Endosol" OR "Endosulphan" OR "Endotaf" OR "ensawan" OR "FMC 5462" OR "fmc5462" OR "hexachlorohexahydro 6, 9 methano 2, 4, 3 benzodioxathiepine 3 oxide" OR "Hexachlorohexahydromethano 2,4,3-benzodioxathiepin-3-oxide" OR "hexachloronorbornene 5, 6 bis (oxymethylene) sulfite" OR "Hildan" OR "Insectophene" OR "malix" OR "Rasayansulfan" OR "Sialan" OR "thifor" OR "thimul" OR "Thiodan" OR "Thiodon" OR "thiofor" OR "thiomul" OR "Thionate" OR "thionex" OR "Thiosulfan" OR "Thiotox" OR "thyodan" OR "thyonex" OR "tiodan" OR "tionel" OR "Tionex" OR "Tiovel") OR TS=(**"pyridaben"** OR "2E4JBA5272" OR "96489-71-3" OR "2-tert-Butyl-5-(4-tert-Butylbenzylthio)-4-chloropyridazin-3(2H)-one" OR "2-tert-Butyl-5-(4-tert-butyl-benzylthio)-4-chloropyridazin-3(2H)-one" OR "2-tert-butyl-5-[(4-tert-butylbenzyl)thio]-4-chloropyridazin-3(2H)-one" OR "2-tert-butyl-5-[(4-tert-butylbenzyl)thio]-4-chloro-pyridazin-3-one" OR "Damanlin" OR "Nexter" OR "Pyramite" OR "Sanmite") OR TS=(**"Methanol"** OR "67-56-1" OR "Y4S76JWI15" OR "carbinol" OR "Carbonal" OR "hydroxymethan" OR "Hydroxymethane" OR "MeOH" OR "Methoxy Group" OR "methyl alcohol" OR "Methyl hydrate" OR "Methyl hydroxide" OR "Methylalcohol" OR "Methylic alcohol" OR "Methylol" OR "MetOH" OR "Monohydroxymethane" OR "monomethylol" OR "wood alcohol" OR "Wood naphtha" OR "wood spirit") OR TS=(**"cyproconazole"** OR "94361-06-5" OR "622B9C3E6T" OR "Atemi" OR "Cyproconazol") OR TS=(**"triticonazole"** OR "131983-72-7" OR "(1RS)-(E)-5-((4-chlorophenyl)methylene)-2,2-dimethyl-1-(1H-1,2,4-triazol-1-ylmethyl)cyclopentan-1-ol") OR TS=(**"diniconazole"** OR "X82HVO1N83" OR "76714-16-4" OR "83657-24-3" OR "1-(2,4-dichlorophenyl)-4,4-dimethyl-2-(1,2,4-triazol-1-yl)-1-penten-3-ol" OR "1-(2,4-Dichlorophenyl)-4,4-dimethyl-2-(1H-1,2,4-triazol-1-yl)pent-1-en-3-ol" OR "Diclopentezol" OR "S 3308" OR "S-3308L") OR TS=(**"Zidovudine"** OR "4B9XT59T7S" OR "30516-87-1" OR "3' azido 2', 3' dideoxyribosylthymine" OR "3' azido 2', 3' dideoxythymidine" OR "3' Azido 2',3' Dideoxythymidine" OR "3' Azido 3' deoxythymidine" OR "3' azido 3' deoxythymidine" OR "3' azidothymidine" OR "3'azido-3'deoxythymidine" OR "3'-azido3'-deoxythymidine" OR "3-Azido-3-deoxythymidine" OR "3'-Azido-3'deoxythymidine" OR "adovi" OR "avirzid" OR "azidodeoxythymidine" OR "azidomine" OR "Azidothymidine" OR "Azitidin" OR "AZT" OR "bio zt" OR "bw a 509 u" OR "BW A509U" OR "BW-A 509U" OR "BWA509U" OR "BWA-509U" OR "Dendrigen" OR "pranadox" OR "retrocar" OR "Retrovir" OR "Retrovis" OR "ZDV" OR "zidis" OR "zidovir" OR "zidovudin" OR "Zidovudinum" OR "zudovidine" OR "zydowin") OR TS=(**"Metoclopramide"** OR "L4YEB44I46" OR "364-62-5" OR "4 amino 5 chloro n (2 diethylaminoethyl) 2 methoxybenzamide" OR "4 amino 5 chloro n (2 diethylaminoethyl) o anisamide" OR "4 amino 5 chloro n (2 diethylaminoethyl) ortho anisamide" OR "4 Amino-5-chloro-N-(2-(diethylamino)ethyl)-2-methoxybenzamide" OR "4-Amino-5-chloro-N-(2-(diethylamino)ethyl)-o-anisamide" OR "4-Amino-5-chloro-N-(2-diethylamino-ethyl)-2-methoxy-benzamide" OR "4-amino-5-chloro-N-[2-(diethylamino)ethyl]-2-methoxybenzamide" OR "4-amino-5-chloro-N-[2-(diethylamino)ethyl]-2-methoxy-benzamide" OR "4-amino-5-chloro-N-[2-(diethylamino)-ethyl]-2-methoxybenzamide" OR "5 chloro 2 methoxyprocainamide" OR "ahr 3070 c" OR "ahr 3070c" OR "ahr3070c" OR "ametic" OR "anausin" OR "apo-metoclop" OR "aputern" OR "betaclopramide" OR "carnotprim primperan" OR "Cerucal" OR "clodilion" OR "clopamon" OR "clopan" OR "Clopra" OR "clopram" OR "Clopromate" OR "Degan" OR "del 1267" OR "del1267" OR "dibertil" OR "Duraclamid" OR "Elieten" OR "emenil" OR "emetal" OR "emetard" OR "Emetid" OR "Emitasol" OR "emperal" OR "encil" OR "enzimar" OR "Eucil" OR "gastro timelets" OR "gastrobi" OR "Gastrobid" OR "Gastromax" OR "Gastronerton" OR "gastrosil" OR "Gastrotablinen" OR "gastrotem" OR "gastrotimelets" OR "gavistal" OR "gensil" OR "Gimoli" OR "hemesis" OR "hyrin" OR "imperan" OR "m 813" OR "m813" OR "maril" OR "Maxeran" OR "maxeron" OR "maxolan" OR "Maxolon" OR "mcp-beta tropfen" OR "meclomid" OR "meclopamide" OR "meclopramide" OR "Meclopran" OR "Megaldrate" OR "meramide" OR "Metaclopramide" OR "Metadrate" OR "metagliz" OR "metamide" OR "Methochlopramide" OR "methoclopramide" OR "methoclopramine" OR "Methoxychlorprocainamide" OR "metlazel" OR "Metochlopramide" OR "Metochloropramide" OR "Metoclol" OR "metoclopamide" OR "metoclopramid" OR "Metoclopramidum" OR "metoclopramine" OR "metoclopranide hydrochloride" OR "metoclor" OR "metoclorpramide" OR "metocobil" OR "metocyl" OR "metodopramide" OR "metolon" OR "metopram" OR "metox" OR "metozolv" OR "metpamid" OR "metram" OR "Metramid" OR "Moriperan" OR "mygdalon" OR "nausil" OR "neopramiel" OR "netaf" OR "nilatika" OR "normastin" OR "Octamide" OR "opram" OR "Parmid" OR "Paspertin" OR "Peraprin" OR "perinorm" OR "pharmyork" OR "Plasil" OR "pramidin" OR "Pramiel" OR "pramin" OR "pramotel" OR "Primperan" OR "primperil" OR "prinparl" OR "prokinyl lp" OR "prowel" OR "pulin" OR "Pylomid" OR "Reclomide" OR "Regla" OR "Reglan" OR "Reliveran" OR "rimetin" OR "sensamide" OR "sotatic-10" OR "Terperan" OR "tomid" OR "vertivom" OR "vomitrol" OR "zumatrol")

OR

**Part 2**

TS=(**"Acebutolol"** OR "67P356D8GH" OR "37517-30-9" OR "1 (2 acetyl 4 butyramidophenoxy) 2 hydroxy 3 isopropylaminopropane" OR "1-(2-Acetyl-4-n-butyramidophenoxy)-2-hydroxy-3-isopropylaminopropane" OR "Acebrutololum" OR "Acebutololo" OR "Acebutololum" OR "acecor" OR "ApoAcebutolol" OR "bay c 7705" OR "bay c7705" OR "diasectral" OR "espesil" OR "flebutol" OR "grifobutol" OR "il 17803a" OR "il17803a" OR "M & B 17803A" OR "m and b 17803 a" OR "M and B 17803A" OR "M and B17803 A" OR "M&B-17803 A" OR "Monitan" OR "n [3 acetyl 4 [2 hydroxy 3 [ (methylethyl) amino] propoxy] phenyl] butanamide" OR "N-[3-acetyl-4-[2-hydroxy-3-[(1-methylethyl)amino]propoxy]phenyl]butanamide" OR "Neptal" OR "neptall" OR "NovoAcebutolol" OR "Prent" OR "Rhotral" OR "Sectral" OR "Wesfalin") OR TS=(**"Isoniazid"** OR "V83O1VOZ8L" OR "54-85-3" OR "4 pyridinecarbohydrazide" OR "4 pyridinecarboxylic acid hydrazide" OR "Abdizide" OR "Andrazide" OR "Anidrasona" OR "antimic" OR "Antimicina" OR "antimicine" OR "Antituberkulosum" OR "apacizina (aminosalicylate)" OR "Armacide" OR "Armazid" OR "Armazide" OR "atcotibin" OR "Atcotibine" OR "Azuren" OR "bacillen" OR "Bacillin" OR "Cedin" OR "Cemidon" OR "Chemiazid" OR "Chemidon" OR "Continazine" OR "Cortinazine" OR "Cotinazin" OR "cotinazine" OR "Cotinizin" OR "cotinizine" OR "curazid forte" OR "Defonin" OR "dianicotyl" OR "diazid" OR "Dibutin" OR "Diforin" OR "Dinacrin" OR "Ditubin" OR "Ebidene" OR "Eralon" OR "eralone" OR "Ertuban" OR "Eutizon" OR "eutizone" OR "Evalon" OR "Fetefu" OR "Fimalene" OR "fimaline" OR "FRS-3" OR "fsr3" OR "Ftivazide" OR "GINK" OR "hain" OR "hiconyl" OR "Hid rasonil" OR "hidraciber" OR "Hidranizil" OR "Hidrasonil" OR "hidrazida" OR "Hidrulta" OR "hidrulte" OR "Hidrun" OR "Hycozid" OR "hycozide" OR "hydrasonil" OR "Hydrazid" OR "Hydrazide" OR "hydrazin" OR "Hyozid" OR "Hyzyd" OR "ido tebin" OR "Idrazil" OR "Inah" OR "inh burgthal" OR "INHd20" OR "Inizid" OR "inizide" OR "Iscotin" OR "iscotine" OR "Isidrina" OR "isidrine" OR "Ismazide" OR "Isobicina" OR "isobicine" OR "Isocid" OR "Isocidene" OR "isoco tin" OR "Isocotin" OR "isocotine" OR "Isohydrazide" OR "Isokin" OR "Isolyn" OR "isolyne" OR "isomazide" OR "isomerina" OR "Isonerit" OR "Isonex" OR "isoniac" OR "Isoniacid" OR "Isoniazide" OR "isoniazidine" OR "Isoniazidum" OR "isoniazone" OR "isonicazid" OR "Isonicazide" OR "Isonicid" OR "isonicide" OR "Isonico" OR "Isonicotan" OR "isonicotane" OR "isonicotic acid hydrazide" OR "Isonicotil" OR "isonicotinate hydrazide" OR "Isonicotinhydrazid" OR "isonicotinhydrazide" OR "Isonicotinic acid hydrazide" OR "isonicotinic acid hydrazine" OR "isonicotinic acid hydrazone" OR "Isonicotinic Acid Vanillylidenehydrazide" OR "Isonicotinic hydrazide" OR "isonicotinicacid hydrazide" OR "Isonicotinohydrazide" OR "Isonicotinoyl hydrazide" OR "isonicotinoylhydrazide" OR "Isonicotinoylhydrazine" OR "Isonicotinyl hydrazide" OR "Isonicotinyl hydrazine" OR "Isonicotinylhydrazide" OR "Isonicotinylhydrazine" OR "Isonide" OR "Isonidrin" OR "isonidrine" OR "Isonikazid" OR "isonikazide" OR "Isonilex" OR "isonilyd" OR "Isonin" OR "Isonindon" OR "isonindone" OR "isonine" OR "Isonirit" OR "isonisin" OR "isonisine" OR "Isoniton" OR "isonitone" OR "isonivit" OR "Isonizida" OR "Isonizide" OR "isopharmide" OR "Isotamine" OR "Isotebe" OR "Isotebezid" OR "isotebezide" OR "isothiavit" OR "Isotinyl" OR "Isozid" OR "Isozide" OR "isozin" OR "isozine" OR "isozone" OR "Isozyd" OR "isozyde" OR "izoniazid" OR "L 1945" OR "Laniazid" OR "Laniozid" OR "LANIZID" OR "Mayambutol" OR "micosan" OR "micosane" OR "milazide" OR "Mybasan" OR "mybasane" OR "Neoteben" OR "neotebene" OR "Neo-Tizide" OR "Neoxin" OR "neoxine" OR "neoxon" OR "neoxone" OR "Neumandin" OR "Nevin" OR "Niadrin" OR "nicatibine" OR "nicazid" OR "Nicazide" OR "Nicetal" OR "Nicizina" OR "nicodrin" OR "Niconyl" OR "nicosciorin" OR "nicotibin" OR "Nicotibina" OR "Nicotibine" OR "Nicotisan" OR "nicotubin" OR "nicotubine" OR "nicozid" OR "Nicozide" OR "nicozyd" OR "Nidaton" OR "Nidrazid" OR "nidrazide" OR "Nikozid" OR "nikozide" OR "niosciorine" OR "Niplen" OR "Nitadon" OR "Niteban" OR "Nitebannsc 9659" OR "nortibina" OR "nortibine" OR "Nydrazid" OR "nydrazide" OR "Nyscozid" OR "nyscozide" OR "Pelazid" OR "pelazide" OR "Percin" OR "Phthisen" OR "Phthivazid" OR "Phthivazide" OR "puran" OR "Pycazide" OR "Pyreazid" OR "pyreazide" OR "Pyricidin" OR "pyricidine" OR "Pyridicin" OR "pyridine 4 carbohydrazide" OR "pyridine 4 carbonic acid hydrazide" OR "pyridine 4 carboxyhydrazide" OR "pyridine-4-carboxylic acid hydrazide" OR "Pyrizidin" OR "pyrizidine" OR "ramnanon" OR "Raumanon" OR "Razide" OR "Retozide" OR "rhymicid" OR "Rifater" OR "Rimicid" OR "rimicide" OR "Rimifon" OR "Rimiphone" OR "Rimitsid" OR "Robiselin" OR "Robisellin" OR "robiselline" OR "Roxifen" OR "roxyfen miquel" OR "RP 5015" OR "Sanohidrazina" OR "sanohydrazina" OR "sanohydrazine" OR "santerazid" OR "santerazide" OR "Sauterazid" OR "Sauterzid" OR "Stanozide" OR "supercidin" OR "tb phlogin" OR "Tebecid" OR "tebecide" OR "tebecin" OR "tebecine" OR "tebemid" OR "Tebenic" OR "tebesium" OR "tebetracin" OR "tebetracine" OR "Tebexin" OR "tebexine" OR "Tebilon" OR "tebilone" OR "Tebos" OR "Teebaconin" OR "teebaconine" OR "Tekazin" OR "tekazine" OR "thiocevit" OR "Tibazide" OR "Tibemid" OR "tibemide" OR "Tibiazide" OR "Tibinide" OR "Tibison" OR "tibisone" OR "tibitan" OR "tibitane" OR "Tibivis" OR "Tibizide" OR "Tibusan" OR "tibusane" OR "Tisin" OR "Tisiodrazida" OR "tisiodrazide" OR "tisiotrazida" OR "Tizide" OR "Tubazid" OR "Tubazide" OR "Tubeco" OR "Tubecotubercid" OR "tubercid" OR "Tuberian" OR "tuberiane" OR "Tubicon" OR "tubicone" OR "Tubilysin" OR "Tubizid" OR "Tubomel" OR "tubonil" OR "tubylisin" OR "tubylisine" OR "tyrid" OR "Tyvid" OR "tyvide" OR "Unicocyde" OR "Unicozyde" OR "valifol" OR "Vazadrine" OR "Vederon" OR "vederone" OR "vitazide" OR "yuhan-zid" OR "Zidafimia" OR "Zinadon" OR "zinadone" OR "Zonazide") OR TS=(**"Saccharin"** OR "FST467XS7D" OR "81-07-2" OR "128-44-9" OR "1, 2 benzisothiazol 3 (2h) one 1, 1 dioxide" OR "1, 2 benzisothiazol 3 one 1, 1 dioxide" OR "1, 2 dihydro 2 ketobenzisosulfonazole" OR "1, 2-Benzisothiazol-3(2H)-one, 1,1-dioxide" OR "1,2-Benzisothiazol-3(2H)-one 1,1-dioxide" OR "1,2-Benzisothiazol-3(2H)-one, 1,1-dioxide" OR "1,2-Dihydro-2-ketobenzisosulfonazole" OR "1,2-Dihydro-2-ketobenzisosulphonazole" OR "2, 3 dihydro 3 oxobenzisosulfonazole" OR "2,3-Dihydro-3-oxobenzisosulfonazole" OR "2,3-Dihydro-3-oxo-Benzisosulfonazole" OR "2,3-Dihydro-3-oxobenzisosulphonazole" OR "2-Sulfobenzoic acid imide" OR "2-Sulfobenzoic imide" OR "2-Sulfobenzoicimide" OR "2-Sulphobenzoic imide" OR "Anhydro-o-sulfaminebenzoic acid" OR "Benzo-2-sulfiide" OR "Benzo-2-sulphimide" OR "Benzoic acid sulfimide" OR "Benzoic sulfimide" OR "Benzoic sulphimide" OR "Benzosulfimide" OR "Benzosulfinide" OR "Benzosulphimide" OR "Benzo-sulphinide" OR "Benzoylsulfonic Imide" OR "Cristallose" OR "Crystallose" OR "Garantose" OR "Glucid" OR "glucide" OR "Gluside" OR "Glycophenol" OR "Hermesetas" OR "Kandiset" OR "Kristallose" OR "Madhurin" OR "Natreen" OR "Neosaccharin" OR "o-Benzoic acid sulfimide" OR "o-Benzoic sulfimide" OR "o-Benzoic sulphimide" OR "o-Benzosulfimide" OR "o-Benzosulphimide" OR "o-Benzoyl sulfimide" OR "o-Benzoyl sulphimide" OR "O-Benzoylsulfimide" OR "ortho sulfobenzimide" OR "ortho sulfobenzoic acid imide" OR "Ortho sulphobenzamide" OR "o-Sulfobenzimide" OR "o-Sulfobenzoic acid imide" OR "O-Sulfobenzoic imide" OR "o-Sulfonbenzoic acid imide sodium salt" OR "Sacarina" OR "Saccharimide" OR "Saccharina" OR "Saccharine" OR "Saccharinol" OR "Saccharinose" OR "saccharoid" OR "Saccharol" OR "Saxin" OR "Sodium o-benzosulfimide" OR "Sodium saccharide" OR "Sucrette" OR "Sucromat" OR "sweet n low" OR "Sweeta" OR "sweetex" OR "sweetnin" OR "Sykose" OR "Syncal" OR "Willosetten" OR "Zaharina") OR TS=(**"Penicillin G"** OR "YS5LY7JF4N" OR "69-57-8" OR "1406-05-9" OR "6 (phenylacetamido) penicillanate sodium" OR "6 phenylacetamidopenicillanate sodium" OR "American penicillin" OR "Benpen" OR "Benzylpenicillin sodium" OR "benzylpenicillinate sodium" OR "Benzylpenicillinic acid sodium salt" OR "bupenna sodium" OR "Coliriocilina" OR "Crystapen" OR "Kesso-Pen" OR "Mycofarm" OR "Novocillin" OR "Parcillin" OR "Pekamin" OR "pen a brasive" OR "Pengesod" OR "Penibiot" OR "Penicilina G Llorente" OR "Penicillin Grünenthal" OR "penicillin ii sodium" OR "penicillin sodium" OR "penicilline g sodium" OR "PenicillinGsodiumsalt" OR "Penilaryn" OR "Penilevel" OR "Peniroger" OR "Pfizerpen" OR "Sodiopen" OR "Sodipen" OR "Sodium 6-(phenylacetamido)penicillanate" OR "sodium benzyl penicillin" OR "Sodium benzylpenicillin" OR "Sodium benzylpenicillinate" OR "Sodium penicillin" OR "sodium penicilline g" OR "Sugracillin sodium salt" OR "Unicilina" OR "Ursopen" OR "Veticillin") OR TS=(**"Thalidomide"** OR "4Z8R6ORS6L" OR "50-35-1" OR ".alpha.-(N-Phthalimido)glutarimide" OR ".alpha.-N-Phthalylglutaramide" OR ".alpha.-Phthalimidoglutarimide" OR "3-Phthalimidoglutarimide" OR "Algosediv" OR "alpha (n phthalimido) glutarimide" OR "alpha-(N-Phthalimido)glutarimide" OR "alpha-N-Phthalylglutaramide" OR "alpha-Phthalimidoglutarimide" OR "Asidon 3" OR "Asmadion" OR "Asmaval" OR "Bonbrain" OR "Calmore" OR "Calmorex" OR "Celgene" OR "Contergan" OR "Corronarobetin" OR "Distaval" OR "Distaxal" OR "Distoval" OR "Ectiluran" OR "Enterosediv" OR "Gastrinide" OR "Glupan" OR "Glutanon" OR "Grippex" OR "Hippuzon" OR "Imidene" OR "Isomin" OR "Kedavon" OR "Kevadon" OR "n (2, 6 dioxopiperid 3 yl) phthalimide" OR "N-(2,6-dioxo-3-piperidinyl)phthalimide" OR "N-(2,6-Dioxo-3-piperidyl)phthalimide" OR "n-(2,6-dioxopiperidin-3-yl)phthalimide" OR "Neaufatin" OR "Neosedyn" OR "Neosydyn" OR "Nerosedyn" OR "Neufatin" OR "Neurodyn" OR "Neurosedin" OR "Neurosedym" OR "Neurosedyn" OR "neurosedyne" OR "Nevrodyn" OR "Nibrol" OR "Noctosediv" OR "Noxodyn" OR "N-Phthalimidoglutamic acid imide" OR "N-Phthaloylglutamimide" OR "N-Phthalylglutamic acid imide" OR "nsc 66847" OR "NSC66847" OR "Pangul" OR "Pantosediv" OR "Pharmion" OR "Polygripan" OR "Pro-Bam M" OR "Pro-ban M" OR "Profarmil" OR "Quetimid" OR "Quietoplex" OR "Sandormin" OR "Sedalis" OR "Sedimide" OR "Sedin" OR "Sedisperil" OR "Sedoval" OR "shin naito" OR "Shinnibrol" OR "Sleepan" OR "Slipro" OR "Softenil" OR "Softenon" OR "Synovir" OR "Talargan" OR "Talidomide" OR "Talimol" OR "Talinol" OR "Talismol" OR "Talizer" OR "Telagan" OR "Telargan" OR "Telargean" OR "Tensival" OR "thado" OR "Thaled" OR "thalidomid" OR "Thalidomidum" OR "Thalin" OR "Thalinette" OR "thalix" OR "Thalomid" OR "Thalomide" OR "Theophilcholine" OR "Valgis" OR "Valgraine" OR "Yodomin") OR TS=(**"Doxylamine"** OR "95QB77JKPL" OR "469-21-6" OR "562-10-7" OR ".alpha.-Dimethylaminoethoxyphenylmethyl-2-picoline" OR "2-Dimethylaminoethoxyphenylmethyl-2-picoline" OR "alsadorm" OR "decapryn" OR "deoxylamine succinate" OR "Diclectin" OR "Dolased" OR "donormyl" OR "dormidina" OR "Dossilamina" OR "doxilamina" OR "doxylaminesuccinate" OR "doxylaminium succinate" OR "doxylaminosuccinate" OR "Doxylaminum" OR "doxy-sleep-aid" OR "Dozile" OR "Evanorm" OR "gittalun" OR "histadoxylamine" OR "hoggar" OR "mereprine" OR "Mersyndol" OR "Restavit" OR "sedaplus" OR "Somnil" OR "Syndol" OR "Unisom" OR "vicks nyquil") OR TS=(**"Diphenhydramine"** OR "8GTS82S83M" OR "58-73-1" OR "147-24-0" OR ".beta.-(Dimethylamino)ethyl benzhydryl ether" OR ".beta.-Dimethylamino-aethyl-benzhydryl-aether" OR "2 (benzhydroloxy) n, n dimethylethylamine" OR "2 (diphenylmethoxy) n, n dimethyl ethylamine" OR "2 (diphenylmethoxy) n, n dimethylethylamine" OR "2 benzhydryloxy n, n dimethylethylamine" OR "2 diphenylmethoxy n, n dimethylethylamine" OR "2-(Benzhydryloxy)-N,N-dimethylethanamine" OR "2-(Benzhydryloxy)-N,N-dimethylethylamine" OR "2-(Benzohydryloxy)-N,N-dimethylethylamine" OR "2-(diphenylmethoxy)-N,N-dimethylethanamine" OR "2-(Diphenylmethoxy)-N,N-dimethylethylamine" OR "2-(diphenylmethyl)oxy-N,N-dimethylethanamine" OR "2-(diphenylmethyl)oxy-N,N-dimethyl-ethanamine" OR "2-[(diphenylmethyl)oxy]-N,N-dimethylethanamine" OR "2-[di(phenyl)methoxy]-N,N-dimethylethanamine" OR "2-benzhydryloxy-N,N-dimethylethanamine" OR "2-benzhydryloxy-N,N-dimethyl-ethanamine" OR "2-diphenylmethoxy-N,N-demthylethanamine" OR "2-Diphenylmethoxy-N,N-dimethylethylamine" OR "Aleryl" OR "Alledryl" OR "Allerdryl" OR "Allergan" OR "Allergeval" OR "Allergical" OR "Allergina" OR "Allergival" OR "Amidryl" OR "Antistominum" OR "Antomin" OR "Automin" OR "Bagodryl" OR "banaril" OR "Banophen" OR "Baramine" OR "Beldin" OR "Belix" OR "Benachlor" OR "benadril" OR "Benadrin" OR "Benadryl" OR "benadyl" OR "Ben-allergin" OR "Benapon" OR "Benhydramin" OR "benocten" OR "Benodin" OR "Benodine" OR "Benylan" OR "Benylin" OR "Benzantine" OR "Benzhydramine" OR "Benzhydraminum" OR "Benzhydroamina" OR "beta dimethylaminoethyl benzhydryl ether" OR "beta-Dimethylaminoethanol diphenylmethyl ether" OR "beta-Dimethylaminoethylbenzhydrylether" OR "Betramin" OR "caladryl" OR "carphenamine" OR "carphenex" OR "cathejell" OR "Compoz" OR "Dabylen" OR "Debendrin" OR "Dermistina" OR "Dermodrin" OR "Desentol" OR "Diabenyl" OR "Diabylen" OR "dibadorm n" OR "Dibendrin" OR "Dibenil" OR "Dibondrin" OR "dibrondrin" OR "Difedryl" OR "Difenhydramin" OR "Difenhydramine" OR "Dihidral" OR "Dimedrol" OR "Dimedryl" OR "Dimehydrinate" OR "Dimethylamine benzhydryl ester" OR "dimidril" OR "dimiril" OR "Diphantine" OR "diphedryl" OR "Diphen" OR "diphenacen" OR "diphendramine" OR "Diphenhist" OR "diphenhydramide" OR "diphenhydramin" OR "Diphenhydraminum" OR "diphenydramine" OR "Diphenylhydramin" OR "Diphenylhydramine" OR "Dobacen" OR "Dormarex 2" OR "Dormin" OR "dryhistan" OR "Dryistan" OR "Drylistan" OR "Dylamon" OR "dytan" OR "emesan" OR "Etanautine" OR "Genahist" OR "Histacyl" OR "Histaxin" OR "histergan" OR "Hyadrine" OR "Hydramine" OR "Hyrexin" OR "Ibiodral" OR "Medidryl" OR "Mephadryl" OR "n, n dimethyl 2 (diphenylmethoxy) ethylamine" OR "N,N-Dimethyl-2-(diphenylmethoxy)-ethylamine hydrochloride" OR "N,N-Dimethyl-2-diphenylmethyloxyethylamine" OR "Nausen" OR "neosynodorm" OR "Novamina" OR "nytol" OR "o benzhydryldimethylaminoethanol" OR "O-Benzhydryl(dimethylamino)ethanol" OR "Probedryl" OR "reisegold" OR "resmin" OR "restamin" OR "Rigidil" OR "Rigidyl" OR "sediat" OR "sedryl" OR "Siladryl" OR "Silphen" OR "sleepeze" OR "Sleep-Eze D" OR "sominex" OR "Syntedril" OR "Syntodril" OR "trux-adryl" OR "tzoali" OR "unisom sleepgels" OR "valdrene" OR "venasmin" OR "vertirosan" OR "vicks formula 44" OR "vilbin" OR "wehdryl" OR "ziradryl") OR TS=(**"clopyralid"** OR "10G14M0WDH" OR "1702-17-6" OR "3,6-Dichloropicolinic acid" OR "Benzalox" OR "Cirtoxin" OR "Cliophar" OR "Clopiralid" OR "Clopyralide" OR "Cyronal" OR "dichloropyridine acid" OR "Dowco 290" OR "Huiloralid" OR "Loncid" OR "Lontrel" OR "Matrigon" OR "Transline" OR "Versatill") OR TS=(**"Camphor"** OR "76-22-2" OR "21368-68-3" OR "1, 7, 7 trimethylbicyclo [2.2.1] heptan 2 one" OR "1,7,7-Trimethyl-bicyclo(2,2,1)Heptan-2-one" OR "1,7,7-Trimethylbicyclo(2.2.1)-2-heptanone" OR "1,7,7-Trimethylbicyclo(2.2.1)heptan-2-one" OR "1,7,7-Trimethylbicyclo[2.2.1]-2-heptanone" OR "1,7,7-Trimethylbicyclo[2.2.1]heptan-2-one" OR "1,7,7-Trimethyl-bicyclo[2.2.1]heptan-2-one" OR "1,7,7-trimethyl-bicyclo[2.2.1]heptane-2-one" OR "2 bornanone" OR "2 camphanone" OR "2 keto 1, 7, 7 trimethylnorcamphane" OR "2 oxo 1, 7, 7 trimethylbicyclo (2.2.1) heptane" OR "2 oxobornane" OR "2-Camphanone" OR "2-Camphonone" OR "2-Keto-1,7,7-trimethylnorcamphane" OR "Alcanfor" OR "Alphanon" OR "Bornan-2-one" OR "camphora" OR "d-2-Bornanone" OR "d-2-Camphanone" OR "korodin" OR "pi hydroxycamphor" OR "Root bark oil" OR "Root bark spirit" OR "sarna") OR TS=(**"fipronil"** OR "QGH063955F" OR "120068-37-3" OR "5 amino 1 (2, 6 dichloro alpha, alpha, alpha trifluoro para tolyl) 4 trifluoromethylsulfinylpyrazole 3 carbonitrile" OR "5 amino 1 [2, 6 dichloro 4 (trifluoromethyl) phenyl] 4 [ (trifluoromethyl) sulfinyl] 1h pyrazole 3 carbonitrile" OR "5-amino-1 -(2,6-dichloro-4-trifluoromethylphenyl)-4-trifluoromethylsulfinyl-1 H-pyrazole-3-carbonitrile" OR "5-amino-1-(2,6-dichloro-4-(trifluoromethyl) phenyl)-4-((trifluoromethyl) sulfinyl)-1H-pyrazol-3-carbonitrile" OR "5-amino-1-(2,6-dichloro-4-(trifluoromethyl)phenyl)-4-((trifluoromethyl)sulfinyl)-1h-pyrazole-3-carbonitrile" OR "5-amino-1-(2,6-dichloro-alpha,alpha,alpha-trifluoro-p-tolyl)-4-trifluoromethylsulfinylpyrazole-3-carbonitile" OR "5-Amino-1-[2,6-dichloro-4-(trifluoromethyl)phenyl]-4-(trifluoromethyl)sulfinylpyrazole-3-carbonitrile" OR "5-amino-1-[2,6-dichloro-4-(trifluoromethyl)phenyl]-4-(trifluoromethylsulfinyl)pyrazole-3-carbonitrile" OR "5-amino-1-[2,6-dichloro-4-(trifluoromethyl)phenyl]-4-[(trifluoromethyl)sulfinyl]-1H-pyrazole-3-carbonitrile" OR "5-amino-1-[2,6-dichloro-4-(trifluoromethyl)phenyl]-4-trifluoromethanesulfinyl-1H-pyrazole-3-carbonitrile" OR "fiprex" OR "Fluocyanobenpyrazole" OR "Frontline Spot-on" OR "Frontline Spray" OR "Frontline Top Spot" OR "Goliath gel" OR "Granedo MC" OR "Maxforce FC" OR "Termidor") OR TS=(**"Glycerol"** OR "PDC6A3C0OX" OR "56-81-5" OR "107283-02-3" OR "144086-02-2" OR "144086-03-3" OR "25618-55-7" OR "8013-25-0" OR "1, 2, 3 propanetriol" OR "1, 2, 3 trihydroxypropane" OR "1,2,3-Propanetriol" OR "1,2,3-triglycerol" OR "1,2,3-TRIHYDROXYPROPAN-2-YL" OR "1,2,3-trihydroxypropane" OR "1,2,3-trihydroxypropanol" OR "1,3-Propanetriol" OR "1,3-Trihydroxypropane" OR "alditol" OR "Artificial tears" OR "Bulbold" OR "Citifluor AF 2" OR "Cristal" OR "Dagralax" OR "Glicerina" OR "Glyceol" OR "glycerin" OR "Glycerine" OR "Glycerinum" OR "Glyceritol" OR "Glycerolum" OR "Glycyl alcohol" OR "Glyrol" OR "Glysanin" OR "Glyzerin" OR "Grocolene" OR "microglycerin" OR "Monoctanoin Component D" OR "Neutracett" OR "Oelsuess" OR "Olsuss" OR "Ophthalgan" OR "Osmoglyn" OR "Polyglycerin" OR "Polyglycerine" OR "Polyglycerol" OR "Pricerine 9091" OR "PROPANE-1,2,3-TRIOL" OR "Propanetriol" OR "Tegin M" OR "Trihydroxypropane" OR "Tryhydroxypropane" OR "vilardell" OR "Vitrosupos") OR TS=(**"hexazinone"** OR "51235-04-2" OR "Y51727MR1Y" OR "3 cyclohexyl 6 (dimethylamino) 1 methyl 1, 3, 5 triazine 2, 4 (1h, 3h) dione" OR "3-Cyclohexy-6-(dimethylamino)-1-methyl-1,3,5-triazine-2,4(1H,3H)-dione" OR "3-Cyclohexyl-6-(dimethylamino)-1-methyl-1,3,5-triazine-2,4(1H,3H)-dione" OR "3-cyclohexyl-6-(dimethylamino)-1-methyl-1,3,5-triazine-2,4-dione" OR "Gridball" OR "Hexazinoe" OR "Hexazinon" OR "Velpar") OR TS=(**"imazamox"** OR "UG6793ON5F" OR "114311-32-9") OR TS=(**"imazapyr"** OR "81334-34-1" OR "2-(4-Isopropyl-4-methyl-5-oxo-2-imidazolin-2-yl)nicotinic acid") OR TS=(**"Loratadine"** OR "7AJO3BO7QN" OR "79794-75-5" OR "4 (8 chloro 5, 6 dihydro 11h benzo [5, 6] cyclohepta [1, 2 b] pyridin 11 ylidene) 1 piperidinecarboxylic acid ethyl ester" OR "4-(8-Chloro-5,6-dihydro-11H-benzo(5,6)cyclohepta(1,2-b)pyridin-11-ylidene)-1-piperidinecarboxylic Acid Ethyl Ester" OR "4-(8-CHLORO-5,6-DIHYDRO-11H-BENZO[5,6]CYCLOHEPTA[1,2-B]PYRIDIN-11-YLIDENE)-1-PIPERIDINECARBOXYLATE" OR "4-(8-chloro-5,6-dihydro-11H-benzo[5,6]cyclohepta[1,2-b]pyridin-11-ylidene)-1-piperidinecarboxylic acid ethyl ester" OR "4-(8-Chloro-5,6-dihydro-11H-benzo[5,6]cyclohepta[1,2-b]pyridin-11-ylidene-1-piperidinecarboxylic acid ethyl ester" OR "8 chloro 11 (1 ethoxycarbonyl 4 piperidylidene) 6, 11 dihydro 5h benzo [5, 6] cyclohepta [1, 2 b] pyridine" OR "8 chloro 6, 11 dihydro 11 (1 carboethoxy 4 piperidylidene) 5h benzo [5, 6] cyclohepta [1, 2 b] pyridine" OR "8-chloro-11-(1-ethoxycarbonyl-4-piperidylidene)-6,11-dihydro-5H-benzo[5,6]cyclohepta[1,2-b]pyridine" OR "8-chloro-6,11-dihydro-11-(1- ethoxycarbonyl-4-piperidylidene)-5H-benzo[5,6]cyclohepta[1,2-b]pyridine" OR "Aerotina" OR "Alarin" OR "Alavert" OR "alerfast" OR "alernitis" OR "Alerpriv" OR "alertadin" OR "alertrin" OR "allerta" OR "Allertidin" OR "allertyn" OR "allohex" OR "ambrace" OR "analergal" OR "Anhissen" OR "anlos" OR "ardin" OR "Bactimicina allergy" OR "Bedix Loratadina" OR "Biloina" OR "Bonalerg" OR "caradine" OR "carin" OR "Civeran" OR "clalodine" OR "Claratyne" OR "clarid" OR "Clarinase" OR "Claritin" OR "Claritine" OR "Clarityn" OR "Clarityne" OR "Clarium" OR "cronitin" OR "Cronopen" OR "curyken" OR "demazin anti-allergy" OR "ethyl 4 (8 chloro 5, 6 dihydro 11h benzo [5, 6] cyclohepta [1, 2 b] pyridin 11 ylidene) 1 piperidinecarboxylate" OR "Ethyl 4-(8-chloro-5,6-dihydro-11H-benzo(5,6)cyclohepta(1,2-b)pyridin-11-ylidene)-1-piperidinecarboxylate" OR "Ethyl 4-(8-chloro-5,6-dihydro-11H-benzo[5,6]cyclohepta[1,2-b]pyridin-11-ylidene)-1-piperidinecarboxylate" OR "ethyl 4-(8-chloro-5,6-dihydro-11H-benzo[5,6]cyclohepta[1,2-b]pyridin-11-ylidene)piperidine-1-carboxylate" OR "Ethyl-4-(8-chloro-5,6-dihydro-11H-benzo [5,6] cyclohepta [1,2-b]pyridin-11-ylidene)-1-piperidinecarboxylate" OR "ezasmin" OR "ezede" OR "finska" OR "Flonidan" OR "frenaler" OR "Fristamin" OR "genadine" OR "halodin" OR "hislorex" OR "histalor" OR "Histaloran" OR "j-tadine" OR "klarihist" OR "Klaritin" OR "klinset" OR "laredine" OR "lergia" OR "Lergy" OR "Lertamine" OR "Lesidas" OR "lindine" OR "Lisino" OR "lisono" OR "lobeta" OR "lodain" OR "Lomilan" OR "lorabasics" OR "Loracert" OR "loraclar" OR "loraderm" OR "Loradex" OR "Loradif" OR "loradin" OR "lorahist" OR "loralerg" OR "lora-lich" OR "lorano" OR "Loranox" OR "Lorantis" OR "lorapaed" OR "Lorastine" OR "lora-tabs" OR "Loratadinum" OR "loratadura" OR "loratan" OR "loratazine" OR "loratidin" OR "Loratidine" OR "loraton" OR "loratrim" OR "Loratyne" OR "Loraver" OR "loraxin" OR "loreen" OR "Lorfast" OR "lorihis" OR "lorita" OR "Loritine" OR "lotadine" OR "lotarin" OR "Lowadina" OR "mosedin" OR "noratin" OR "notamin" OR "Nularef" OR "onemin" OR "Optimin" OR "Polaratyne" OR "proactin" OR "Restamine" OR "Rhinase" OR "ridamin" OR "rihest" OR "rinityn" OR "Rinolan" OR "Rinomex" OR "rityne" OR "Roletra" OR "Sanelor" OR "Sch 29851" OR "Sch29851" OR "Sensibit" OR "Sinhistan Dy" OR "Sohotin" OR "Symphoral" OR "Tadine" OR "Talorat Dy" OR "tidilor" OR "tirlor" OR "toradine" OR "Velodan" OR "Versal" OR "voratadine" OR "Zeos") OR TS=(**"novaluron"** OR "Z8H1B3CW0B" OR "116714-46-6" OR "1-(3-chloro-4-(1,1,2-trifluoro-2-trifluoromethoxyethoxy)phenyl)-3-(2,6-difluorobenzoyl)urea" OR "1-[3-Chloro-4-(1,1,2-trifluoro-2-trifluoromethoxyethoxy)phenyl]-3-(2,6-difluorobenzoyl)urea" OR "1-{3-CHLORO-4-[1,1,2-TRIFLUORO-2-(TRIFLUOROMETHOXY)ETHOXY]PHENYL}-3-(2,6-DIFLUOROBENZOYL)UREA" OR "Rimon EC-10") OR TS=(**"2-phenylphenol"** OR "90-43-7" OR "61788-42-9" OR "D343Z75HT8" OR "1-Hydroxy-2-phenylbenzene" OR "2 biphenylol" OR "2 hydroxydiphenyl" OR "2 phenylphenol" OR "2-Biphenylol" OR "2-hydroxy biphenyl" OR "2-Hydroxy-1,1'-biphenyl" OR "2-Hydroxybiphenyl" OR "2-Hydroxydiphenyl" OR "2-Phenyl phenol" OR "Amocid" OR "Anthrapole 73" OR "Biphenyl-2-ol" OR "Biphenylol" OR "Dowicide" OR "Hydroxy-2-phenylbenzene" OR "Hydroxybiphenyl" OR "Invalon OP" OR "Lyorthol" OR "Nectryl" OR "Nipacide OPP" OR "o-Biphenylol" OR "o-Diphenylol" OR "o-Hydroxybiphenyl" OR "o-Hydroxydiphenyl" OR "o-Phenyl phenol" OR "o-phenylphenate" OR "o-phenylphenol" OR "ortho hydroxybiphenyl" OR "ortho hydroxydiphenyl" OR "ortho phenylphenol" OR "Orthohydroxydiphenyl" OR "Orthophenyl phenol" OR "ortho-phenylphenate" OR "Orthophenylphenol" OR "Orthoxenol" OR "o-Xenol" OR "o-Xonal" OR "Preventol 3041" OR "Preventol O extra" OR "Remol TRF" OR "Rotoline" OR "sodium o-phenylphenoate" OR "Stellisept" OR "Tetrosin oe" OR "Torsite" OR "Xenol") OR TS=(**"Propylene Glycol"** OR "6DC9Q167V3" OR "57-55-6" OR "123120-98-9" OR "63625-56-9" OR "1, 2 dihydroxypropane" OR "1, 2 propandiol" OR "1, 2 propanediol" OR "1, 2 propylenglycol" OR "1, 2-propanediol" OR "1,2 Propanediol" OR "1,2-(RS)-Propanediol" OR "1,2-dihydroxypropan-2-yl" OR "1,2-dihydroxypropane" OR "1,2-Dihydroxypropanl" OR "1,2-propandiol" OR "1,2-propane diol" OR "1.2-propanediol" OR "2,3-Propanediol" OR "2-Hydroxypropanol" OR "Aliphatic alcohol" OR "apopropanediol" OR "Ilexan P" OR "Isopropylene glycol" OR "methyl ethyl glycol" OR "methyl glycol" OR "Methylethyl glycol" OR "Methylethylene glycol" OR "methylglycol" OR "Monopropylene glycol" OR "Prolugen" OR "propan 1, 2 diol" OR "Propan-1,2-Diol" OR "propane 1, 2 diol" OR "propane-1,2-diol" OR "propanediol" OR "propyleneglycol" OR "propylenglycol" OR "Sirlene" OR "Trimethyl glycol") OR TS=(**"triclopyr"** OR "MV06PHJ6I0" OR "55335-06-3" OR "3,5,6-TPA" OR "((3,5,6-trichloro-2-pyridinyl)oxy)-acetic acid" OR "[(3,5,6-trichloro-2-pyridinyl)oxy]-acetic acid" OR "3,5,6-Trichloro-2-pyridinyloxyacetic Acid" OR "((3,5,6-trichloro-2-pyridyl)oxy)-acetic acid" OR "3,5,6-Trichloro-2-pyridyloxyacetic acid" OR "Garlon" OR "Grazon ET" OR "Trichlopyr" OR "Turflon") OR TS=(**"triethylene glycol"** OR "112-27-6" OR "103734-98-1" OR "122784-99-0" OR "137800-98-7" OR "145112-98-7" OR "3P5SU53360" OR "2, 2' ethylenedioxybis (ethanol)" OR "2,2'-(Ethylenedioxy)diethanol" OR "2,2-(Ethylenedioxy)diethanol" OR "2,2'-Ethylenedioxybis(ethanol)" OR "2,2'-Ethylenedioxydiethanol" OR "3, 6 dioxaoctane 1, 8 diol" OR "3,6-Dioxa-1,8-octanediol" OR "3,6-Dioxaoctane-1,8-diol" OR "Bis(2-hydroxyethoxyethane)" OR "Di-.beta.-hydroxyethoxyethane" OR "Di-beta-hydroxyethoxyethane" OR "Ethylene glycol dihydroxydiethyl ether" OR "Ethylene glycol-bis-(2-hydroxyethyl ether)" OR "Glycol bis(hydroxyethyl) ether" OR "Tri-ethylene glycol" OR "Triethyleneglycol" OR "triethylenglycol" OR "Trigenos" OR "Triglycol" OR "Trigol") OR TS=(**"zoxamide"** OR "156052-68-5" OR "RH 7281" OR "RH7281" OR "Zoxamid" OR "Zoxium") OR TS=(**"pyriproxyfen"** OR "3Q9VOR705O" OR "95737-68-1" OR "126040-81-1" OR "2 [1 methyl 2 (4 phenoxyphenoxy) ethoxy] pyridine" OR "2-(1-Methyl-2-(4-phenoxyphenoxy)ethoxy)pyridine" OR "2-[ 1-methyl-2-(4-phenoxyphenoxy)ethoxy]pyridine" OR "2-[1-methyl-2-(4-phenoxyphenoxy)ethoxy] pyridine" OR "2-[1-Methyl-2-(4-phenoxyphenoxy)ethoxy]pyridine" OR "4-Phenoxyphenyl (RS)-2-(2-pyridyloxy)propyl ether" OR "Archer IGR" OR "Cyclio" OR "juvinal" OR "NyGuard IGR" OR "Nylar" OR "Pyriproxifen" OR "S 31183" OR "S31183" OR "Sumilarv") OR TS=(**"methyl cellosolve"** OR "109-86-4" OR "9004-74-4" OR "95507-80-5" OR "EK1L6XWI56" OR "1-Hydroxy-2-methoxyethane" OR "2-(methoxy)ethanol" OR "2-(methyloxy)ethanol" OR "2-HYDROXY-1-METHOXYETHYL" OR "2-Hydroxyethyl methyl ether" OR "2-methoxy ethanol" OR "2-Methoxy-1-ethanol" OR "2-Methoxyethan-1-Ol" OR "2methoxyethanol" OR "2-methoxyethanol" OR "2-Methoxyethyl alcohol" OR "2-methoxylethanol" OR "3-Oxa-1-butanol" OR "alpha-hydro-omega-methoxypoly(oxyethylene)" OR "beta-Methoxyethanol" OR "dimethyleneglycol monomethylether" OR "Dowanol 7" OR "Dowanol EM" OR "Ektasolve EM" OR "Ethylene glycol methyl ether" OR "Ethylene glycol monomethyl ether" OR "ethylene glycol monomethylether" OR "Ethyleneglycol monomethyl ether" OR "ethyleneglycol monomethylether" OR "ethylglycol monomethyl ether" OR "Glycol ether EM" OR "Glycol monomethyl ether" OR "Glycolmethyl ether" OR "Jeffersol EM" OR "Karl Fischer Reagent" OR "Methoxyhydroxyethane" OR "Methyl ethoxol" OR "Methyl icinol" OR "Methyl oxitol" OR "methylcellosolve" OR "methylcello-solve" OR "Monoethylene glycol methyl ether" OR "Monomethyl ether of ethylene glycol" OR "Monomethyl ethylene glycol ether" OR "Monomethyl glycol" OR "O-Methyl Glycol") OR TS=(**"Acetaminophen"** OR "362O9ITL9D" OR "103-90-2" OR "4 hydroxyacetanilide" OR "4' hydroxyacetanilide" OR "4-(Acetylamino)phenol" OR "4-(N-Acetylamino)phenol" OR "4-acetamido phenol" OR "4-ACETAMIDOPHENYLOXIDANYL" OR "A.F. Anacin" OR "Abenol" OR "Abensanil" OR "Abrol" OR "Abrolet" OR "Acamol" OR "Acenol" OR "Acephen" OR "Acertol" OR "Acetaco" OR "Acetagesic" OR "Acetalgin" OR "Acetamidophenol" OR "acetamino phenol" OR "Acetaminofen" OR "acetaminophene" OR "acetaminophenol" OR "Acetamol" OR "Acetavance" OR "Acetofen" OR "acetomenophen" OR "Acetominophen" OR "acetominophene" OR "acetylaminophenol" OR "acetyl-p-aminophenol" OR "Actamin" OR "Actimol" OR "adorem" OR "Afebrin" OR "Afebryl" OR "Aferadol" OR "Algesidal" OR "algiafin" OR "Algina" OR "algocit" OR "Algomol" OR "Algotropyl" OR "alphagesic" OR "Alpiny" OR "Alpinyl" OR "Alvedon" OR "Amadil" OR "Aminofen" OR "Anacin 3" OR "Anacin3" OR "anadin" OR "Anaflon" OR "analgiser" OR "Analter" OR "Anapap" OR "Andox" OR "Anelix" OR "Anexsia" OR "Anhiba" OR "Antidol" OR "Anuphen" OR "Apacet" OR "Apadon" OR "Apamid" OR "Apamide" OR "APAP" OR "apirex" OR "Apitrelal" OR "apotel" OR "Arfen" OR "Arthralgen" OR "Asetam" OR "Asomal" OR "Aspac" OR "Asplin" OR "atamel" OR "Atasol" OR "Atralidon" OR "Babikan" OR "Bacetamol" OR "Banesin" OR "Benmyo" OR "benuron" OR "Ben-u-ron" OR "Biocetamol" OR "biogesic" OR "bodrex" OR "Bucet" OR "Butapap" OR "Cadafen" OR "Calapol" OR "Calmanticold" OR "calodol" OR "Calonal" OR "Calpol" OR "Capital with Codeine" OR "Captin" OR "Causalon" OR "Cefalex" OR "cemol" OR "Cetadol" OR "christamol" OR "Citramon P" OR "claradol" OR "Claratal" OR "Clixodyne" OR "clocephen" OR "Codabrol" OR "Codalgin" OR "Codapane" OR "Codicet" OR "Codisal" OR "Codoliprane" OR "Cofamol" OR "Co-Gesic" OR "Conacetol" OR "Cosutone" OR "cp 500" OR "cp500" OR "Cuponol" OR "Curadon" OR "Curpol" OR "Dafalgan" OR "Daphalgan" OR "Darocet" OR "Darvocet" OR "Datril" OR "Demilets" OR "Deminofen" OR "Democyl" OR "Demogripal" OR "depon" OR "depyretin" OR "Desfebre" OR "Dhamol" OR "Dimindol" OR "Dirox" OR "dismifen" OR "Disprol" OR "dolal" OR "Dolcor" OR "Dolefin" OR "dolex" OR "Dolgesic" OR "Doliprane" OR "dolitabs" OR "Dolko" OR "dolofen" OR "Dolofugin" OR "dolomol" OR "Doloreduct" OR "dolorol" OR "Dolotec" OR "dolotemp" OR "Dolprone" OR "doltem" OR "Dorocoff" OR "Dresan" OR "drilan" OR "dristan af" OR "Duaneo" OR "Dularin" OR "Duorol" OR "Duracetamol" OR "Durapan" OR "Dymadon" OR "Ecosetol" OR "efferalgan" OR "efferalganodis" OR "efferelgan" OR "Elixodyne" OR "Empracet" OR "Endecon" OR "Enelfa" OR "Eneril" OR "eraldor" OR "eu med" OR "Excipain" OR "exopon" OR "expandol" OR "Fanalgic" OR "Farmadol" OR "Febranine" OR "Febrectal" OR "Febrectol" OR "Febrex" OR "Febricet" OR "Febridol" OR "Febrilix" OR "Febrin" OR "Febrinol" OR "Febrolin" OR "Fendon" OR "Fensum" OR "Fepanil" OR "fervex" OR "fibrinol" OR "Finimal" OR "Fluparmol" OR "fortolin" OR "Gelocatil" OR "Geluprane" OR "Genapap" OR "Genebs" OR "Grippostad" OR "Gynospasmine" OR "Hedex" OR "helporal" OR "Homoolan" OR "Hydroxyacetanilide" OR "Hy-Phen" OR "Ildamol" OR "Inalgex" OR "infants' feverall" OR "Injectapap" OR "Intensin" OR "Janupap" OR "kamolas" OR "Kataprin" OR "Korum" OR "kyofen" OR "Labamol" OR "Lekadol" OR "Lemgrip" OR "Lemsip" OR "Lestemp" OR "letamol" OR "Liqiprine" OR "liquiprin" OR "Lonarid" OR "lotemp" OR "Lupocet" OR "Lyteca" OR "Magnidol" OR "Malgis" OR "Malidens" OR "Maxadol" OR "medamol" OR "Medocodene" OR "meforagesic" OR "Melabon Infantil" OR "metagesic" OR "metalid" OR "Mexalen" OR "Midol" OR "Minafen" OR "minopan" OR "Minoset" OR "Miralgin" OR "Mono Praecimed" OR "n acetyl 4 aminophenol" OR "n acetyl para aminophenol" OR "N-(4-Hydroxyphenyl)acetamide" OR "N-(4-hydroxyphenyl)-acetamide" OR "N-(4-Hydroxyphenyl)acetanilide" OR "N-(4-hydroxyphenyl)ethanamide" OR "n-acetyl-4-hydroxyaniline" OR "N-Acetyl-p-aminophenol" OR "Naldegesic" OR "nalgesik" OR "Napafen" OR "napamol" OR "NAPAP" OR "naprex" OR "Naprinol" OR "Nealgyl" OR "NeoCitran" OR "neodalmin" OR "Neodol" OR "Neodolito" OR "Neo-Fepramol" OR "Neopap" OR "Neuridon" OR "nevral" OR "nilapur" OR "Nobedon" OR "Nodolex" OR "Noral" OR "Norco" OR "nysacetol" OR "Ofirmev" OR "Oltyl" OR "Oralgan" OR "Oraphen-PD" OR "Ortensan" OR "Oxycocet" OR "p-(Acetylamino)phenol" OR "Paceco" OR "Pacemo" OR "Pacemol" OR "Pacet" OR "p-Acetamidophenol" OR "p-Acetaminophenol" OR "p-Acetoaminophen" OR "p-Acetylaminophenol" OR "Pacimol" OR "Paedialgon" OR "Paedol" OR "Painex" OR "Paldesic" OR "Pamol" OR "Panacete" OR "Panadeine" OR "Panadiene" OR "Panado-Co" OR "Panadol" OR "Panaleve" OR "Panamax" OR "Panasorb" OR "Panasorbe" OR "Panex" OR "Panodil" OR "Panofen" OR "Pantalgin" OR "para acetamidophenol" OR "para acetylaminophenol" OR "para hydroxyacetanilide" OR "para suppo" OR "Paracemol" OR "Paracenol" OR "Paracet" OR "paracetaminophenol" OR "Paracetamol" OR "Paracetamole" OR "Paracetamolum" OR "Paracetanol" OR "Paracetol" OR "Paracin" OR "Paracod" OR "Paracodol" OR "Parador" OR "parageniol" OR "paragin" OR "Parakapton" OR "Parake" OR "Paralen" OR "Paralief" OR "Paralink" OR "Paralyoc" OR "paramax" OR "paramidol" OR "Paramol" OR "Paramolan" OR "Paranox" OR "parapaed" OR "Parapan" OR "Parasedol" OR "Parasin" OR "Paraspen" OR "paratabs" OR "Para-Tabs" OR "Parcetol" OR "Parelan" OR "Parmol" OR "Parogal" OR "Paroma" OR "parvid" OR "Pasolind" OR "paximol" OR "Pediapirin" OR "Pediatrix" OR "pedipan" OR "Pedric" OR "Perdolan Mono" OR "Perfalgan" OR "Phenaphen" OR "Phendon" OR "Phenipirin" OR "Phogoglandin" OR "Phrenilin" OR "p-hydroxy-acetanilid" OR "p-Hydroxyacetanilide" OR "p-hydroxyacetoanilide" OR "p-Hydroxyphenolacetamide" OR "Pinex" OR "Piramin" OR "Pirinasol" OR "Plicet" OR "polarfen" OR "Polmofen" OR "Predimol" OR "Prodol" OR "Prontina" OR "Propacet" OR "Puernol" OR "Pulmofen" OR "Pyrigesic" OR "Pyrinazine" OR "Pyromed" OR "Quiet World" OR "raperon" OR "rapidol" OR "Redutemp" OR "relaphen" OR "Reliv" OR "Remedol" OR "Resfenol" OR "Resprin" OR "revanin" OR "rhodapap" OR "Rivalgyl" OR "Robigesic" OR "Rounox" OR "Rubophen" OR "Rupemol" OR "Salzone" OR "Sanicet" OR "Sanicopyrine" OR "Scanol" OR "Sedalito" OR "Sedapap" OR "sedes a" OR "Semolacin" OR "serimol" OR "Servigesic" OR "Seskamol" OR "Setakop" OR "Setamol" OR "Setol" OR "Sifenol" OR "Sinaspril" OR "Sine-Aid" OR "sinebriv" OR "Sinedol" OR "Sine-Off" OR "Sinmol" OR "sinpro" OR "Sinubid" OR "Snaplets-FR" OR "St Joseph Aspirin-Free" OR "Stanback" OR "Stopain" OR "Sunetheton" OR "Supadol mono" OR "Supofen" OR "Suppap" OR "Supramol-M" OR "Tabalgin" OR "tachipirin" OR "tachipirina" OR "taganopain" OR "Talacen" OR "Tapanol" OR "Tapar" OR "Tazamol" OR "Tempanal" OR "Tempra" OR "tempte" OR "Tencon" OR "Termacet" OR "Termalgin" OR "Termalgine" OR "Termofren" OR "Theraflu" OR "Tiffy" OR "Titralgan" OR "Toximer P" OR "Tralgon" OR "tramil" OR "Treupel N" OR "Treuphadol" OR "Triaprin" OR "Tricoton" OR "turpan" OR "Tussapap" OR "Tycolet" OR "Tylenol" OR "Tylex" OR "Tylol" OR "Tylox" OR "Tymol" OR "Upsanol" OR "Utragin" OR "Valadol" OR "Valgesic" OR "Valorin" OR "Veralgina" OR "Vermidon" OR "Verpol" OR "Vicodin" OR "Vivimed" OR "Volpan" OR "wegmal" OR "winadol" OR "winasorb" OR "Wygesic" OR "xebramol" OR "Zatinol" OR "Zolben" OR "zydinol" OR "Zydone") OR TS=(**"Ascorbic Acid"** OR "PQ6CK8PD0R" OR "50-81-7" OR "53262-66-1" OR "1-Xyloascorbic Acid" OR "Acidum ascorbicum" OR "acidylina" OR "adenex" OR "agrumina" OR "allercorb" OR "allescorb" OR "Antiscorbic vitamin" OR "Antiscorbutic factor" OR "antiscorbutic vitamin" OR "arcavit c" OR "arcavite c" OR "arkovital c" OR "ascelat" OR "ascofar" OR "Ascoltin" OR "ascomed" OR "asconvita" OR "ascor" OR "ascorbate" OR "ascorbicap" OR "ascorbicin" OR "ascorbico" OR "ascorbin" OR "ascorbina" OR "ascorbinic acid" OR "Ascorbinsaure" OR "ascorbit" OR "ascorbite" OR "ascorbitol" OR "ascorbivit" OR "ascorbivite" OR "ascorbone" OR "ascorbutina" OR "ascorbyl" OR "ascorbyn" OR "ascorgil" OR "ascorin" OR "ascormin" OR "ascorteal" OR "ascorval" OR "ascorvel" OR "ascorvit" OR "ascorvite" OR "ascorvitina" OR "askorbin" OR "austrovit c" OR "austrovite c" OR "bentavit c" OR "bentavite c" OR "c tamin" OR "c vimin" OR "c vit" OR "c vita" OR "cantan" OR "cantaxin" OR "catavin c" OR "ce vi sol" OR "cebetate" OR "cebicure" OR "Cebid" OR "cebion" OR "cebione" OR "cecap" OR "cecon" OR "cecone" OR "cecorbin" OR "cecorbine" OR "cecorbyl" OR "cecorbyle" OR "cecrisina" OR "cedon" OR "cedone" OR "cedoxon" OR "cedoxone" OR "ceevifil" OR "cegiolan" OR "Ceklin" OR "celaskon" OR "celaskone" OR "celin" OR "Cemagyl" OR "Cemill" OR "cenetone" OR "cenol" OR "cenolate" OR "cequinyl" OR "cereon" OR "cergona" OR "cescorbat" OR "cetamican" OR "cetamid" OR "cetamine" OR "cetebe" OR "Cetemican" OR "ceterapion" OR "ceterapione" OR "cetrinets" OR "cevalin" OR "cevaline" OR "cevatine" OR "cevex" OR "cevibid" OR "Cevi-Bid" OR "cevibram" OR "cevigal" OR "cevigen" OR "cevigol" OR "cevilat" OR "cevimin" OR "cevimine" OR "cevisol" OR "ce-vi-sol" OR "cevit" OR "cevita" OR "Cevital" OR "Cevitamate" OR "cevitamic acid" OR "cevitamin" OR "cevitaminic acid" OR "cevitan" OR "cevite" OR "cevitex" OR "cevitil" OR "cevitol" OR "cewin" OR "chewcee" OR "chivibit c" OR "Chromagen" OR "ciamin" OR "ciergin" OR "cifilina" OR "cipca" OR "cisir" OR "citamino" OR "citoascorbina" OR "citoxyl" OR "citran" OR "citravite" OR "Citriscorb" OR "citritabs" OR "Citrovit" OR "citrovitamina" OR "civigor" OR "civitin" OR "civitine" OR "Colascor" OR "concemin" OR "cortalex" OR "c-vimin" OR "dagrascorbin" OR "dagravit c" OR "dancimin c" OR "davitamon c" OR "dayvital" OR "difvitamin c" OR "dumovit c" OR "dumovite c" OR "Duoscorb" OR "erftamin c" OR "erftamine c" OR "esuron" OR "esurvit" OR "esurvite" OR "Ferancee" OR "godabion c" OR "gregovite c" OR "hicee" OR "hybrin" OR "ido c" OR "inovitan c" OR "irocevit" OR "irocevite" OR "jarexin" OR "jarexine" OR "Juvamine" OR "l 3 keto hexuronic acid lactone" OR "L-3-ketothreohexuronic acid" OR "lacivit" OR "lacivite" OR "laroscorbine" OR "L-AscorbicAcid" OR "leder c" OR "lemascorb" OR "limcee" OR "L-lyxoascorbate" OR "L-Lyxoascorbic acid" OR "L-Threoascorbic acid" OR "L-threo-Ascorbic acid" OR "L-xyloascorbate" OR "Magnorbin" OR "myascorbin" OR "natrascorb" OR "novo ascorbic" OR "nybadol" OR "Parentrovite" OR "parkovit c" OR "pharmascorbine" OR "pharmatovit c" OR "pharmatovite c" OR "planavit c" OR "planavite c" OR "plivit c" OR "plivite c" OR "proscorbin" OR "proscorbine" OR "redoxon" OR "ribena" OR "Rovimix C" OR "scorbacid" OR "scorbacide" OR "scorbex" OR "scorbin c" OR "scorbitol" OR "scorbumine" OR "scottavit c" OR "scottavite c" OR "secorbate" OR "Semidehydroascorbate" OR "sevalin" OR "sigmavit c" OR "sigmavite c" OR "sodascorbate" OR "Sunkist" OR "synum c" OR "tanvimil-c" OR "testascorbic" OR "Tolfrinic" OR "ucemine c" OR "upsa-c" OR "vicef" OR "vicelat" OR "vicetrin" OR "viciman" OR "vicin" OR "vicitina" OR "vicon" OR "viforcit" OR "viforcite" OR "viscorin" OR "viscorine" OR "vitace" OR "vitacee" OR "vitacimin" OR "vitacimine" OR "vitacin" OR "vitacine" OR "vitamin C" OR "Vitamisin" OR "vitaplex c" OR "vitapric" OR "vitapur c" OR "vitasan c" OR "vitascorbin" OR "vitascorbine" OR "vitascorbol" OR "vitelix c" OR "wandervit c" OR "wandervite c" OR "witamina c" OR "xitix" OR "xyloascorbic acid") OR TS=(**"butylparaben"** OR "3QPI1U3FV8" OR "94-26-8" OR "4 hydroxybenzoic acid butyl ester" OR "4-(Butoxycarbonyl)phenol" OR "4-Hydroxybenzoic acid-n-butyl ester" OR "Aseptoform butyl" OR "butoben" OR "butyl 4 hydroxybenzoate" OR "butyl butex" OR "butyl hydroxybenzoic acid" OR "butyl para hydroxybenzoate" OR "Butyl paraben" OR "butyl parabenbutyl parahydroxybenzoatebutyl p-hydroxybenzoate" OR "Butyl parahydroxybenzoate" OR "Butyl p-hydroxybenzoate" OR "butylhydroxybenzoate" OR "n-Butyl hydroxybenzoate" OR "Nipabutyl" OR "para hydroxybenzoic acid butyl ester" OR "p-Hydroxy butyl benzoate" OR "p-Hydroxybenzoic acid butyl ester" OR "p-Hydroxybenzoic acid n-butyl ester" OR "p-Hydroxybenzoic butyl ester") OR TS=(**"Carbamazepine"** OR "33CM23913M" OR "298-46-4" OR "(5h) dibenz (b, f) azepine 5 carboxamide" OR "5 carbamoyl 5h dibenz [b, f] azepine" OR "5-Carbamoyl-5H-dibenz(b,f)azepine" OR "5-Carbamoyl-5H-dibenz[b,f]azepine" OR "5-Carbamoyl-5H-dibenzo(b,f)azepine" OR "5h dibenz [b, f] azepine 5 carboxamide" OR "5H-Dibenz(b,f)azepine-5-carboxamide" OR "5H-Dibenz[ b, f]azepine-5-carboxamide" OR "5H-Dibenz[b,f]azepine-5-carboxamide" OR "5H-Dibenzo[b,f]azepine-5-carboxamide" OR "amizepin" OR "amizepine" OR "apo-carbamazepine" OR "atretol" OR "Bipotrol" OR "biston" OR "calepsin" OR "camapine" OR "carbadac" OR "Carbamazepen" OR "carbamazepin" OR "Carbamazepinum" OR "Carbamezepine" OR "carbategral" OR "carbatol" OR "carbatrol" OR "carbazene" OR "carbazep" OR "Carbazepin" OR "Carbazepine" OR "carbazina" OR "Carbelan" OR "carmaz" OR "carnexiv" OR "carpaz" OR "carzepin" OR "carzepine" OR "clostedal" OR "convuline" OR "epileptol" OR "epimax" OR "epitol" OR "equetro" OR "espa-lepsin" OR "finlepsin" OR "foxalepsin" OR "g 32883" OR "g32883" OR "hermolepsin" OR "Iminostilbene-N-carboxamide" OR "karbamazepin" OR "kodapan" OR "lexin" OR "mazepine" OR "mazetol" OR "neugeron" OR "neurotol" OR "neurotop" OR "nordotol" OR "Novo-Carbamaz" OR "panitol" OR "servimazepin" OR "sirtal" OR "spd 417" OR "spd417" OR "Stazepin" OR "Stazepine" OR "tardotol" OR "taver" OR "tegol" OR "tegral" OR "tegretal" OR "tegretol" OR "tegrital" OR "telesmin" OR "temporol" OR "teril" OR "timonil" OR "Trimonil") OR TS=(**"dimethyl phthalate"** OR "08X7F5UDJM" OR "131-11-3" OR "avolin" OR "citrola" OR "Dimethyl benzeneorthodicarboxylate" OR "Dimethyl o-phthalate" OR "Dimethyl orthophthalate" OR "dimethylphthalate" OR "dmp 30" OR "dmp30" OR "fermine" OR "Kemester DMP" OR "Kodaflex DMP" OR "methyl phthalate" OR "mipax" OR "mugia" OR "palatinol m" OR "Phthalic acid dimethyl ester" OR "Repeftal" OR "sketofax" OR "Solvanom" OR "Solvarone" OR "Unimoll DM" OR "Uniplex 110") OR TS=(**"dimethylamine"** OR "124-40-3" OR "6912-12-5" OR "ARQ8157E0Q" OR "dimethlamine" OR "dimethyamine" OR "dimethyl amine" OR "di-methylamine" OR "dimethylammonia" OR "dimethylammonium chloride" OR "dimethylammonium formate" OR "dimethylarnine" OR "dirnethylamine") OR TS=(**"Penicillamine"** OR "GNN1DV99GX" OR "52-67-5" OR "3, 3 dimethylcysteine" OR "3,3-Dimethyl-D(-)-cysteine" OR "3,3-Dimethyl-D-cysteine" OR "3-Mercapto-D-valine" OR "3-sulfanyl-D-valine" OR "adaleen" OR "alpha amino beta methyl beta mercaptobutyric acid" OR "artamin" OR "Artamine" OR "atamir" OR "beta, beta dimethylcysteamine" OR "beta-Thiovaline" OR "byanodine" OR "Copper penicillaminate" OR "cuprenil" OR "cuprim" OR "cuprimin" OR "cuprimine" OR "cuprimune" OR "cupripen" OR "Depamine" OR "depen" OR "dextropenicillamine" OR "dimethyl cysteine" OR "Dimethylcysteine" OR "distamine" OR "d-penamine" OR "D-Penicilamine" OR "D-Penicyllamine" OR "d-penil" OR "gerodyl" OR "kelatin" OR "kelatine" OR "Kuprenil" OR "Mercaptovaline" OR "mercaptyl" OR "metalcaptase" OR "pemine" OR "pendramine" OR "penicillame" OR "penicillamin" OR "Penicillamina" OR "Penicillaminum" OR "penicillinamine" OR "Perdolat" OR "Sufirtan" OR "Sufortan" OR "sufortanon" OR "trolovol") OR TS=(**"Folic Acid"** OR "935E97BOY8" OR "59-30-3" OR "32108-06-8" OR "acfol" OR "Acidum folicum" OR "Acifolic" OR "Aspol" OR "Cytofol" OR "Dosfolat B activ" OR "Facid" OR "filicine" OR "Folacid" OR "folacin" OR "Folan" OR "folart" OR "Folasic" OR "folate" OR "Folbal" OR "Folcidin" OR "Folcysteine" OR "foldine" OR "Folettes" OR "foliamin" OR "Folic" OR "folicet" OR "folicid" OR "folinsyre" OR "Folipac" OR "folitab" OR "folium acid" OR "folivit" OR "Folovit" OR "Folsaeure" OR "folsan" OR "Folsaure" OR "Folsav" OR "folverlan" OR "folvite" OR "Folvron" OR "gravi-fol" OR "Incafolic" OR "ingafol" OR "lactobacillus casei factor" OR "lafol" OR "lexpec" OR "megafol" OR "Millafol" OR "Mittafol" OR "n [para [ (2 amino 4 hydroxy 6 pteridylmethyl) amino] benzoyl] glutamic acid" OR "N-(p-(((2-Amino-4-hydroxy-6-pteridinyl)methyl)amino)benzoyl)-L-glutamic acid" OR "N-{p-[(2-amino-4-hydroxypteridin-6-yl)methylamino]benzoyl}glutamic acid" OR "neocepri" OR "Novofolacid" OR "nsc 3073" OR "PteGlu" OR "Pteroyglutamic acid" OR "pteroyl glutamate" OR "pteroyl l glutamic acid" OR "pteroyl monoglutamate" OR "pteroylglutamate" OR "pteroylglutamic acid" OR "Pteroyl-L-glutamate" OR "Pteroyl-L-monoglutamate" OR "Pteroyl-L-monoglutamic acid" OR "pteroylmonoglutamate" OR "pteroylmonoglutamic acid" OR "rubiefol" OR "vifolin" OR "Vitamin B11" OR "Vitamin B9" OR "vitamin bc" OR "vitamin m") OR TS=(**"Genistein"** OR "DH2M523P0H" OR "446-72-0" OR "690224-00-1" OR "4', 5, 7 trihydroxyisoflavone" OR "4',5, 7-Trihydroxyisoflavone" OR "4,5,7-Trihydroxy Iso-Flavone" OR "4',5,7-Trihydroxy isoflavone" OR "4,5,7-Trihydroxyisoflavone" OR "4',5,7-Trihydroxyisoflavone" OR "Bonistein" OR "differenol a" OR "Genestein" OR "genisteine" OR "Genisteol" OR "Genisterin" OR "prunetol" OR "Sophoricol") OR TS=(**"methoxyacetic acid"** OR "625-45-6" OR "F11T1H7Q7W" OR "(methyloxy)acetic acid" OR "methoxy acetic acid" OR "methoxyacetate" OR "methoxyessigs" OR "Methoxyethanoic acid" OR "methyloxyacetic acid") OR TS=(**"N-methylpyrrolidone"** OR "JR9CE63FPM" OR "872-50-4" OR "30207-69-3" OR "51013-18-4" OR "1 methyl 2 pyrrolidene" OR "1-methyl pyrrolidinone" OR "1-methyl-2-pyrolidinone" OR "1-Methylazacyclopentan-2-one" OR "1-Methylazacyclopentane-2-one" OR "1-methylpyrrolid-2-one" OR "1-methyl-pyrrolidin-2-one" OR "1-methylpyrrolidine-2-one" OR "1-N-methyl-2-pyrrolidinone" OR "Agsolex 1" OR "methyl pyrrolidone" OR "Methyl-2-pyrrolidinone" OR "methyl-2-pyrrolidone" OR "methylpyrrolidin-2-one" OR "Methylpyrrolidinone" OR "Methylpyrrolidone" OR "M-Pyrol" OR "N-methyl 2-pyrolidone" OR "N-methyl pirrolidone" OR "N-methyl pyrollidone" OR "N-methyl pyrrolidinone" OR "n-methyl pyrrolidon" OR "N-Methyl-.alpha.-pyrrolidinone" OR "N-Methyl-.alpha.-pyrrolidone" OR "N-Methyl-.gamma.-butyrolactam" OR "N-methyl-2-pyrolidinone" OR "N-methyl-2-pyrolidone" OR "N-methyl-2-pyrrolidinon" OR "N-Methyl-2-pyrrolidon" OR "N-Methyl-alpha-pyrrolidinone" OR "N-Methyl-alpha-pyrrolidone" OR "n-methylbutyrolactam" OR "N-Methyl-gamma-butyrolactam" OR "N-methylpyrolidin-2-one" OR "N-Methylpyrrolid-2-one" OR "N-methyl-pyrrolid-2-one" OR "N-methyl-pyrrolidin-2-one" OR "N-methylpyrrolidin-2-one" OR "N-methylpyrrolidine-2-one" OR "N-Methylpyrrolidinon" OR "N-Methylpyrrolidinone" OR "N-Methylpyrrolidon" OR "N-methyl-pyrrolidon" OR "pharmasolve" OR "Pyrol M") OR TS=(**"o,p'-DDT"** OR "D4K93Z1TBH" OR "789-02-6" OR "1 (2 chlorophenyl) 1 (4 chlorophenyl) 2, 2, 2 trichloroethane" OR "1 (ortho chlorophenyl) 1 (para chlorophenyl) 2, 2, 2 trichloroethane" OR "1, 1, 1 trichloro 2 (ortho chlorophenyl) 2 (para chlorophenyl) ethane" OR "1,1,1-trichloro-2-(2-chlorophenyl)-2-(4-chlorophenyl)ethane" OR "1,1,1-Trichloro-2-(o-chlorophenyl)-2-(p-chlorophenyl)ethane" OR "1-Chloro-2-(2,2,2-trichloro-1-(4-chlorophenyl)ethyl)benzene" OR "1-Chloro-2-[2,2,2-trichloro-1-(4-chlorophenyl)ethyl]benzene" OR "2 (2 chlorophenyl) 2 (4 chlorophenyl) 1, 1, 1 trichloroethane" OR "2 (ortho chlorophenyl) 2 (para chlorophenyl) 1, 1, 1 trichloroethane" OR "2-(o-chlorophenyl)-2-(p-chlorophenyl)-1,1,1-trichloroethane" OR "2-(2-Chlorophenyl)-2-(4-chlorophenyl)-1,1,1-trichloroethane" OR "2, 2, 2 trichloro 1 (2 chlorophenyl) 1 (4 chlorophenyl) ethane" OR "2,4'-DDT" OR "o, p ddt" OR "o, p' DDT" OR "o, p dichlorodiphenyltrichloroethane" OR "o,p-DDT" OR "o,p'-dichlorodiphenyltrichloroethane" OR "op ddt" OR "ortho, para ddt" OR "ortho, para' ddt" OR "ortho,para'-DDT") OR TS=(**"Rotenone"** OR "03L9OT429T" OR "83-79-4" OR "Barbasco" OR "Canex" OR "Cubor" OR "Dactinol" OR "Deril" OR "Derrin" OR "Derris root" OR "Extrax" OR "Foliafume" OR "Gerane" OR "Haiari" OR "Mexide" OR "Nekoe" OR "Nicouline" OR "Noxfire" OR "Noxfish" OR "Nusyn" OR "Paraderil" OR "Prenfish" OR "Prentox" OR "protax" OR "Ronone" OR "Rotacide" OR "Rotefive" OR "Rotefour" OR "Rotenoid" OR "rotenon" OR "Rotenox" OR "Roteonone" OR "Rotessenol" OR "Rotocide" OR "Synpren" OR "tubatoxin" OR "Tubotoxin") OR TS=(**"Sucrose"** OR "57-50-1" OR "122880-25-5" OR "25702-74-3" OR "92004-84-7" OR "C151H8M554" OR "1-alpha-D-glucopyranosyl-2-beta-D-fructofuranoside" OR "alpha d glucopyranosyl beta d fructofuranoside" OR "Amerfand" OR "Amerfond" OR "beet sugar" OR "cane sugar" OR "Granulated sugar" OR "Microse" OR "microtal" OR "Polysucrose" OR "sacarosa" OR "saccharose" OR "Saccharum" OR "Sacharose" OR "Sucraloxum" OR "Sugar spheres" OR "tabfine" OR "Table sugar" OR "White sugar") OR TS=(**"tetrabromobisphenol A"** OR "FQI02RFC3A" OR "79-94-7" OR "bis(2,3-dibromopropylether)-2,2-bis(3,5-dibromo-4-(2,3-dibromopropoxy)phenyl)propane" OR "Bromdian" OR "Firemaster BP4A" OR "TBBPA" OR "tetrabromo 4, 4' isopropylidenediphenol" OR "TETRABROMO-4,4'-ISOPROPYLIDENEDIPHENOL" OR "TetrabromobisphenolA" OR "Tetrabromodian" OR "Tetrabromodiphenylopropane")

**Limited to the year range 1926-2016**

(1926 was the earliest possible date)

Part 1 – **3,292 results**, 7/13/18

Part 2 – **2,417 results**, 7/13/18

Total results = ((Part1) OR (Part 2)) – **5,373 results**, 7/13/18

**Final Mammalian Embase Search Strategy**

'rat'/exp OR 'rabbit'/exp OR (Rattus OR 'Sprague-Dawley' OR Wistar OR 'Long-Evans' OR rat OR rats OR Oryctolagus OR rabbit OR rabbits):ab,de,kw,lnk,ti

**AND**

'embryology'/exp OR 'embryo research'/exp OR 'mammalian embryo'/exp OR 'embryo'/exp OR 'fetus'/exp OR 'fetus (anatomy)'/de OR 'fetus bladder'/exp OR 'fetus brain'/exp OR 'fetus heart'/exp OR 'fetus kidney'/exp OR 'fetus liver'/exp OR 'fetus lung'/exp OR 'prenatal development'/de OR 'prenatal growth'/exp OR 'embryo development'/de OR 'fetus development'/exp OR 'organogenesis'/de OR 'mother'/de OR 'dam (animal)'/exp OR 'prenatal period'/exp OR 'prenatal exposure'/exp OR 'prenatal drug exposure'/exp OR 'maternal exposure'/exp OR 'pregnancy'/de OR 'gestation period'/exp OR (embryo* OR fetus* OR foetus* OR fetal* OR foetal* OR 'ductus arteriosus' OR 'endocardial cushion*' OR 'atrioventricular canal cushion*' OR 'truncus arteriosus' OR organogenesis OR mother* OR dam OR dams OR maternal* OR pregnancy OR pregnancies OR pregnant OR congenital* OR prenatal* OR 'pre-natal' OR 'pre-natally' OR intrauterine OR 'intra-uterine' OR antenatal* OR gestation* OR 'transplacental exposure*'):ab,de,kw,lnk,ti OR ((('tooth development'/exp OR 'lymphangiogenesis'/exp OR 'limb development'/exp OR 'musculoskeletal development'/exp OR 'craniofacial development'/exp OR 'nervous system development'/de OR 'sex differentiation'/exp OR 'brain development'/exp OR 'retina development'/exp OR 'eye development'/exp OR 'gonad development'/de OR 'ovary development'/exp OR 'testis development'/exp OR 'heart development'/exp OR 'kidney development'/exp OR 'liver development'/exp OR 'lung development'/exp) OR ('organ development' OR 'tooth development' OR 'tooth formation' OR 'tooth growth' OR 'tooth calcification' OR 'tooth mineralization' OR 'dental development' OR 'dental formation' OR cementogenesis OR cementification OR 'cementum formation' OR dentinogenesis OR dentinogeneses OR dentification OR 'dentin formation' OR dentogenesis OR odontogenesis OR odontogeneses OR amelogenesis OR amelogeneses OR 'enamel formation' OR lymphangiogenesis OR lymphangiogeneses OR 'musculoskeletal development' OR 'musculoskeletal system development' OR 'limb development' OR 'bone development' OR 'bone growth' OR 'physiologic calcification' OR 'physiological calcification' OR 'bone mineralization' OR 'maxillofacial development' OR 'craniofacial development' OR 'face development' OR 'facial development' OR 'skull development' OR 'cranial development' OR 'skull growth' OR osteogenesis OR osteogeneses OR 'bone formation' OR ossification OR osteoclastogenesis OR osteoclastogeneses OR chondrogenesis OR chondrogeneses OR 'muscle development' OR 'muscular development' OR myogenesis OR myogeneses OR myofibrillogenesis OR myofibrillogeneses OR neurogenesis OR neurogeneses OR 'nervous system development' OR 'neurologic development' OR 'brain development' OR 'brain cortex development' OR 'cerebral development' OR 'brain maturation' OR 'brain maturity' OR 'eye development' OR 'retina development' OR 'sex differentiation' OR 'sexual differentiation' OR 'gonad* development' OR 'gonad* differentiation' OR 'sex* gland development' OR 'ovar* development' OR 'ovar* maturation' OR 'follicle development' OR folliculogenesis OR 'ovar* follicle formation' OR 'follicle maturation' OR 'testis development' OR 'testicle development' OR 'testicular development' OR 'testi* descent' OR 'descensus testiculorum' OR 'descensus testis' OR 'testis descensus' OR 'heart development' OR 'cardiac development' OR 'heart growth' OR 'kidney development' OR 'kidney growth' OR 'renal development' OR 'renal growth' OR 'liver development' OR 'hepatic development' OR 'lung development' OR 'pulmonary development'):ab,de,kw,lnk,ti) AND (embryo* OR fetus* OR foetus* OR fetal* OR foetal* OR congenital* OR prenatal* OR 'pre-natal' OR 'pre-natally' OR intrauterine OR 'intra-uterine' OR antenatal* OR gestation*):ab,de,kw,lnk,ti)

**AND**

'toxicology'/exp OR 'comparative toxicology'/exp OR 'toxicological parameters'/de OR 'hormesis'/exp OR 'lethal dose'/exp OR 'IC50'/exp OR 'maximum tolerated dose'/exp OR 'maximum permissible dose'/exp OR 'no-observed-adverse-effect level'/exp OR 'protective index'/exp OR 'therapeutic index'/exp OR 'toxic dose'/exp OR 'body burden'/exp OR 'drug residue'/exp OR 'pesticide residue'/exp OR 'toxicokinetics'/exp OR 'toxicity testing'/de OR 'poison'/exp OR 'teratogenic agent'/exp OR 'teratogenesis'/exp OR 'teratology'/exp OR 'drug induced malformation'/exp OR (toxic* OR neurotoxi* OR hormesis OR hormeses OR hormetic OR 'lethal dos*' OR 'fatal dos*' OR LD10 OR LD100 OR LD50 OR L.D.50 OR 'LD 50' OR LD90 OR LD95 OR LD99 OR 'sublethal dos*' OR 'inhibitory concentration 50' OR IC50 OR 'IC 50' OR '50% inhibitory concentration' OR 'inhibitory concentration 50%' OR 'half maximal inhibitory concentration' OR 'half maximum inhibitory concentration' OR 'median inhibitory concentration' OR 'maximum tolerated dos*' OR 'maximal* tolerated dos*' OR 'maximum tolerable dos*' OR 'maximal* tolerable dos*' OR 'maximum permissible dos*' OR 'maximal permissible dos*' OR 'maximum permissible exposure level*' OR 'permissible level*' OR 'permissible limit*' OR 'maximum dos*' OR 'maximal dos*' OR 'no-observ*-adverse-effect* level*' OR NOAEL OR NOAELs OR 'no-observ*-effect* level*' OR 'no effect dos* level*' OR 'non-observ* effect dos* level*' OR 'non-observ*-effect level*' OR 'no-observ*-adverse-event* level*' OR 'protective index*' OR 'protective indices' OR 'therapeutic index*' OR 'therapeutic indices' OR 'safety window*' OR 'therapeutic ratio' OR 'therapeutic ratios' OR 'therapeutic window*' OR 'therapeutic drug index*' OR 'therapeutic drug indices' OR 'therapeutic drug window*' OR 'toxic dos*' OR TD50 OR 'body burden*' OR 'drug residue*' OR 'pesticide residue*' OR poison* OR teratogen* OR teratolog* OR teratomorph* OR teratotoxic* OR embryotoxi* OR fetotoxi* OR dysmorpholog* OR 'drug-induced' OR malform* OR (('OECD guideline*' OR 'OECD testing guideline*' OR 'test guideline*') AND 414) OR 'guideline 414' OR 'guideline no. 414' OR 'guideline number 414' OR 'OECD no. 414' OR 'OECD number 414' OR 'OECD 414' OR 'TG 414'):ab,de,kw,lnk,ti

**AND**

**Part 1**

**'alitretinoin'/exp** OR ('1UA8E65KDZ' OR '5300-03-8' OR '(9cis)-retinoic acid' OR '[3H]9-cis-retinoic acid' OR '9(Z)-Retinoic acid' OR '9-(Z)-retinoic Acid' OR '9-cis-RA' OR '9-cis-Retinoate' OR '9-cis-Retinoic acid' OR '9-cis-Tretinoin' OR '9CRA' OR '9C-RA' OR '9-CRA' OR '9cRA compound' OR '9-Retinoate' OR '9-Retinoic acid' OR 'agn 192013' OR 'agn192013' OR 'Alitretinoin' OR 'ALRT 1057' OR 'ALRT1057' OR 'BAL4079' OR 'BAL-4079' OR 'DB00523' OR 'LG100057' OR 'LG-100057' OR 'LGD 100057' OR 'LGD 1057' OR 'lgd100057' OR 'LGD1057' OR 'nsc 659772' OR 'nsc659772' OR 'Panretin' OR 'Panretyn' OR 'Panrexin' OR 'Ro-04-4079' OR 'Toctino'):ab,de,kw,lnk,rn,ti,tn OR **'fluorouracil'/exp** OR ('U3P01618RT' OR '51-21-8' OR '2, 4 dioxo 5 fluoropyrimidine' OR '5 fluoro 2, 4 pyrimidinedione' OR '5 fluoropyrimidine 2, 4 dione' OR '5 fu' OR '5-Faracil' OR '5-florouracil' OR '5-Fluoracil' OR '5-Fluoracyl' OR '5-fluorouacil' OR '5-fluorourasil' OR '5-Fluracil' OR '5-Ftouracyl' OR '5FU' OR '5F-uracil' OR '5-HU Hexal' OR 'accusite' OR 'actino-hermal' OR 'Adrucil' OR 'agicil' OR 'Arumel' OR 'Carac' OR 'Carzonal' OR 'cinkef-u' OR 'Effluderm' OR 'Efudex' OR 'Efudix' OR 'Efurix' OR 'eurofluor' OR 'F 6627' OR 'f6627' OR 'fivoflu' OR 'Fluoro Uracil' OR 'Fluoroblastin' OR 'Fluoroplex' OR 'Fluorouracil' OR 'Fluorouracile' OR 'Fluoro-uracile' OR 'Fluorouracilo Ferrer Far' OR 'Fluoruracil' OR 'Fluouracil' OR 'fluoxan' OR 'flurablastin' OR 'Fluracedyl' OR 'Fluracil' OR 'fluracilium' OR 'Fluracilum' OR 'Fluri' OR 'Fluril' OR 'Fluro Uracil' OR 'Fluroblastin' OR 'fluroblastine' OR 'Flurodex' OR 'Ftoruracil' OR 'Haemato-FU' OR 'ifacil' OR 'Kecimeton' OR 'Neofluor' OR 'nsc 18913' OR 'nsc18913' OR 'NSC19893' OR 'NSC-19893' OR 'oncofu' OR 'Onkofluor' OR 'Phthoruracil' OR 'Phtoruracil' OR 'Queroplex' OR 'Ribofluor' OR 'ro2 9757' OR 'Ro-29757' OR 'Ro-2-9757' OR 'Timazin' OR 'Tolak' OR 'uflahex' OR 'utoral'):ab,de,kw,lnk,rn,ti,tn OR **'mevinolin'/exp** OR ('9LHU78OQFD' OR '75330-75-5' OR '6alpha-Methylcompactin' OR '6-alpha-Methylcompactin' OR '6-Methylcompactin' OR 'Advicor' OR 'Altocor' OR 'Altoprev' OR 'Artein' OR 'Belvas' OR 'birotin' OR 'Cholestra' OR 'cid_53232' OR 'Closterol' OR 'Colevix' OR 'cysin' OR 'DB00227' OR 'ellanco' OR 'elstatin' OR 'Hipolip' OR 'Hipovastin' OR 'l 654969' OR 'L-154803' OR 'Lestatin' OR 'Lipdip' OR 'Lipivas' OR 'Lipofren' OR 'Liposcler' OR 'lofacol' OR 'lomar' OR 'lostatin' OR 'lovacel' OR 'lovacol' OR 'lovahexal' OR 'Lovalip' OR 'Lovalord' OR 'lovastan' OR 'Lovastatin' OR 'Lovasterol' OR 'Lovastin' OR 'lovatadin' OR 'lowachol' OR 'Lozutin' OR 'medostatin' OR 'Mevacor' OR 'meverstin' OR 'Mevinacor' OR 'Mevinolin' OR 'Mevlor' OR 'mk 0803' OR 'mk0803' OR 'MK803' OR 'MK-803' OR 'Monacolin K' OR 'Monakolin K' OR 'msd 803' OR 'neolipid' OR 'Nergadan' OR 'ovasta' OR 'Paschol' OR 'Rextat' OR 'Rodatin' OR 'Rovacor' OR 'Sivlor' OR 'Statosan' OR 'Taucor' OR 'Tecnolip' OR 'Teroltrat'):ab,de,kw,lnk,rn,ti,tn OR **'phthalic acid 2 ethylhexyl monoester'/exp** OR ('4376-20-9' OR 'FU2EWB60RT' OR '(2 ethylhexyl) phthalate' OR '(2-Ethylhexyl) hydrogen phthalate' OR '2 ethylhexyl phthalate' OR '2 ethylhexylphthalate' OR '2-Ethylhexyl hydrogen phthalate' OR 'MEHP' OR 'mono (2 ethylhexyl) phthalate' OR 'mono 2 ethylhexyl phthalate' OR 'mono-(2-ethyl)hexyl phthalate' OR 'Mono(2-ethylhexyl) phthalate' OR 'Mono-(2-ethylhexyl) phthalate' OR 'Mono(2-ethylhexyl)phthalate' OR 'Mono-(2-ethylhexyl)phthalate' OR 'mono(ethylhexyl) phthalate' OR 'mono-ethylhexyl' OR 'Monoethylhexyl phthalate' OR 'Monoethylhexyl phthalic acid' OR 'monoethylhexylphthalate' OR 'mono-ethylhexylphthalate' OR 'phthalic acid 2 ethylhexyl ester' OR 'phthalic acid 2 ethylhexyl monoester' OR 'phthalic acid mono (2 ethylhexyl) ester' OR 'Phthalic Acid Mono(2-ethylhexyl) Ester' OR 'PHTHALIC ACID MONO-2-ETHYLHEXYL ESTER' OR 'Phthalic acid mono-2-ethylhexylester' OR 'Phthalic acid, mono-(2-ethylhexyl) ester' OR 'Phthalic acid, mono-2-ethylhexyl ester' OR 'Phthalic Acid-d4 Mono(2-ethylhexyl) Ester'):ab,de,kw,lnk,rn,ti,tn OR **'caffeine'/exp** OR ('3G6A5W338E' OR '58-08-2' OR '95789-13-2' OR '1, 3, 7 trimethyl 2, 6 dioxopurine' OR '1,3,7-Trimethyl-2,6-dioxopurine' OR '1,3,7-Trimethylpurine-2,6-dione' OR '1,3,7-trimethylxanthine' OR '1,7-Trimethyl-2,6-dioxopurine' OR '1-methyltheobromine' OR '1-methyl-Theobromine' OR '7-methyl Theophylline' OR '7-Methyltheophylline' OR 'Alert-pep' OR 'animine' OR 'cafalgine' OR 'Cafamil' OR 'Cafecon' OR 'Cafeina' OR 'cafeine' OR 'Cafergot' OR 'Caffedrine' OR 'Caffein' OR 'Caffeina' OR 'Caffeine' OR 'Caffeinum' OR 'Caffine' OR 'Cafipel' OR 'coffein' OR 'Coffeine' OR 'Coffeinum' OR 'Darvon compound-65' OR 'Dasin' OR 'Dexitac' OR 'DHCplus' OR 'Durvitan' OR 'Eldiatric C' OR 'Enerjets' OR 'Ercatab' OR 'Guaranine' OR 'guarin' OR 'Hycomine' OR 'Kofein' OR 'Koffein' OR 'Lanorinal' OR 'Mateina' OR 'Methyltheobromide' OR 'Methyltheobromine' OR 'Methylxanthine theophylline' OR 'Miudol' OR 'Nix Nap' OR 'no doz' OR 'Nodaca' OR 'nodoz' OR 'nymusa' OR 'Organex' OR 'P-A-C Analgesic Tablets' OR 'pac compound' OR 'Pep-Back' OR 'Percoffedrinol N' OR 'Percutafeine' OR 'peyona' OR 'Phensal' OR 'Propoxyphene Compound 65' OR 'Quick Pep' OR 'QuickPep' OR 'Respia' OR 'SK-65 Compound' OR 'teina' OR 'Theine' OR 'Tirend' OR 'trimethylxanthine' OR 'Vivarin' OR 'Wigraine'):ab,de,kw,lnk,rn,ti,tn OR **'busulfan'/exp** OR ('G1LN9045DK' OR '55-98-1' OR '1, 4 bis (methanesulfonyloxy) butane' OR '1, 4 butanediol dimethanesulfonate' OR '1, 4 dimethanesulfonyloxybutane' OR '1, 4 dimethylsulfonyloxybutane' OR '1,4-Bis(methanesulfonoxy)butane' OR '1,4-Bis(methanesulfonyloxy)butane' OR '1,4-Butanedi yl dimethanesulfonate' OR '1,4-BUTANEDIOL DIMETHANESULFONATE' OR '1,4-Butanediol dimethanesulphonate' OR '1,4-Butanediol dimethylsulfonate' OR '1,4-butanedioldimethanesulfonate' OR '1,4-Butanediyl dimethanesulfonate' OR '1,4-Di(methylsulfonoxy)butane' OR '1,4-Dimesyloxybutane' OR '1,4-Dimethane sulfonyl oxybutane' OR '1,4-Dimethanesulfonoxybutane' OR '1,4-Dimethanesulfonoxylbutane' OR '1,4-Dimethanesulfonyloxybutane' OR '1,4-Dimethanesulphonyloxybutane' OR '1,4-Dimethylsulfonoxybutane' OR '1,4-Dimethylsulfonyloxybutane' OR 'Bisulfex' OR 'Busilvex' OR 'Busulfan' OR 'Busulfano' OR 'Busulfanum' OR 'busulfex' OR 'busulphan' OR 'Busulphane' OR 'butane-1,4-diyl dimethanesulfonate' OR 'Butanedioldimethanesulfonate' OR 'Buzulfan' OR 'citosulfan' OR 'cytoleukon' OR 'glyzophrol' OR 'krn 246' OR 'krn246' OR 'Leucosulfan' OR 'mablin' OR 'Mielevcin' OR 'Mielosan' OR 'mielucin' OR 'Milecitan' OR 'Mileran' OR 'misulban' OR 'mitistan' OR 'mitosan' OR 'mitostan' OR 'muleran' OR 'myelenkon' OR 'myeleran' OR 'Myeleukon' OR 'myeloleukon' OR 'Myelosan' OR 'Myelosanum' OR 'myeloxan' OR 'myelucin' OR 'mylecitan' OR 'Mylecytan' OR 'Myleran' OR 'Mylerlan' OR 'n-Butane-1,3-di(methylsulfonate)' OR 'nsc 750' OR 'NSC750' OR 'Sulfabutin' OR 'Sulphabutin' OR 'tetramethylene dimesylate'):ab,de,kw,lnk,rn,ti,tn OR **'warfarin'/exp** OR ('129-06-6' OR '5Q7ZVV76EI' OR '81-81-2' OR '1 (4 hydroxy 3 coumarinyl) 1 phenyl 3 butanone' OR '3 (alpha acetonylbenzyl) 4 hydroxycoumarin' OR '3 acetonylbenzonyl 4 hydroxy coumarinedimethylaminoethanol' OR '3 alpha phenyl beta acetylethyl 4 hydroxycoumarin' OR '3-(.alpha.-Acetonylbenzyl)-4-hydroxycoumarin' OR '3-(.alpha.-Phenyl-.beta.-acetylaethyl)-4-hydroxycumarin' OR '3-(.alpha.-Phenyl-.beta.-acetylethyl)-4-hydroxycoumarin' OR '3-(1-Phenyl-2-acetylethyl)-4-hydroxycoumarin' OR '3-(a-acetonylbenzyl)-4-hydroxycoumarin' OR '3-(Acetonylbenzyl)-4-hydroxycoumarin' OR '3-(alpha-Acetonylbenzyl)-4-hydroxycoumarin' OR '3-(alpha-Phenyl-beta-acetylaethyl)-4-hydroxycumarin' OR '3-(alpha-Phenyl-beta-acetylethyl)-4-hydroxycoumarin' OR '4-hydroxy-3-(3-oxo-1-phenylbutyl)-1-benzopyran-2-one' OR '4-Hydroxy-3-(3-oxo-1-phenylbutyl)-2H-1-benzopyran-2-one' OR 'acetonylbenzylhydroxycoumarin' OR 'adoisine' OR 'Aldocumar' OR 'alpha acetonylbenzyl 4 hydroxycoumarin dimethylaminoethanol' OR 'antrombin k' OR 'Athrombin' OR 'athrombine k' OR 'athrombinek' OR 'befarin' OR 'Brumolin' OR 'carfin' OR 'circuvit' OR 'CO-Rax' OR 'coumadan' OR 'coumadin' OR 'coumadine' OR 'Coumafen' OR 'coumafene' OR 'Coumaphen' OR 'coumaphene' OR 'Coumefene' OR 'Cov-R-Tox' OR 'dagonal' OR 'DB00682' OR 'delta-con' OR 'Dethmor' OR 'Dethnel' OR 'Dicusat E' OR 'farin' OR 'Frass-Ratron' OR 'jantoven' OR 'Kumader' OR 'Kumadu' OR 'kumatox' OR 'Kypfarin' OR 'maforan' OR 'marevan' OR 'Mar-Frin' OR 'Maveran' OR 'orfarin' OR 'panwarfarin' OR 'panwarfin' OR 'Prothromadin' OR 'Ratorex' OR 'Ratox' OR 'Ratoxin' OR 'Ratron' OR 'Rattunal' OR 'Rodafarin' OR 'Rosex' OR 'Sewarin' OR 'simarc-2' OR 'Sofarin' OR 'Solfarin' OR 'Sorexa plus' OR 'Tedicumar' OR 'Temus W' OR 'tintorane' OR 'uniwarfin' OR 'Vampirinip II' OR 'Vampirinip iii' OR 'wafarin' OR 'waran' OR 'Warf 10' OR 'Warf 42' OR 'Warfant' OR 'warfar' OR 'Warfarat' OR 'Warfarin' OR 'Warfarina' OR 'warfarine' OR 'Warfarinum' OR 'Warficide' OR 'warfil 5' OR 'warfilone' OR 'warnerin' OR 'Zoocoumarin'):ab,de,kw,lnk,rn,ti,tn OR **'ketoconazole'/exp** OR ('R9400W927I' OR '142128-59-4' OR '65277-42-1' OR '1 [4 [4 [ [2 (2, 4 dichlorophenyl) 2 (1h imidazol 1 ylmethyl) 1, 3 dioxolan 4 yl] methoxy] phenyl] 1 piperazinyl] ethanone' OR '1 [4 [4 [ [2 (2, 4 dichlorophenyl) 2 [ (1h imidazol 1 yl) methyl] 1, 3 dioxolan 4 yl] methoxy] phenyl] piperazin 1 yl] ethan 1 one' OR '1 acetyl 4 [4 [ [2 (2, 4 dichlorophenyl) 2 (1h imidazol 1 ylmethyl) 1, 3 dioxolan 4 yl] methoxy] phenyl] piperazine' OR '4 (4 acetylpiperazin 1 yl) alpha [2 (2, 4 dichlorophenyl) 2 imidazol 1 ylmethyl 1, 3 dioxolan 4 yl] anisole' OR 'akorazol' OR 'anfuhex' OR 'antanazol' OR 'beatoconazole' OR 'bigazol' OR 'cetonax' OR 'comozol' OR 'conazol' OR 'cremosan' OR 'daktagold' OR 'dezoral' OR 'dio 902' OR 'dio902' OR 'extina' OR 'formyco' OR 'fugen' OR 'funazole tabs' OR 'funet' OR 'fungarest' OR 'fungaway' OR 'fungazol tabs' OR 'fungiderm-k' OR 'funginoc' OR 'funginox tabs' OR 'fungoral' OR 'kenazol' OR 'kenazole' OR 'kesnazol' OR 'ketazol' OR 'ketocanazole' OR 'keto-comp' OR 'ketoconazol' OR 'Ketoconazole' OR 'Ketoconazolum' OR 'keto-crema' OR 'ketoderm' OR 'ketoisdin' OR 'ketomed' OR 'ketomicin' OR 'ketomicol' OR 'ketona' OR 'keto-shampoo' OR 'ketozal' OR 'ketozol' OR 'ketozole' OR 'kezon' OR 'konaturil' OR 'Kuric' OR 'kw 1414' OR 'lusanoc' OR 'micoral' OR 'mizole' OR 'mizoron' OR 'mycofebrin' OR 'nastil' OR 'nazole' OR 'neutrogena t/sal' OR 'nisoral' OR 'niz creme' OR 'niz shampoo' OR 'nizoral' OR 'oxocanazole' OR 'oxoconazole' OR 'oxonazol' OR 'panfungol' OR 'pasalen' OR 'picamic' OR 'prenalon' OR 'pristinex' OR 'profungal' OR 'r 41, 400' OR 'R 41,400' OR 'r 41400' OR 'R41,400' OR 'R41400' OR 'sebizole' OR 'sporium' OR 'sporoxyl' OR 'sporozol' OR 'termizol' OR 'terzolin' OR 'triatop lotion' OR 'Xolegel' OR 'zoralin tabs' OR 'zorinax'):ab,de,kw,lnk,rn,ti,tn OR **'hydroxyurea'/exp** OR ('X6Q56QN5QC' OR '127-07-1' OR '(HYDROXYCARBAMOYL)AMINYL' OR '1-oxidanylurea' OR 'aminohydroxamic acid' OR 'biosupressin' OR 'carbamic acid oxime' OR 'carbamide oxide' OR 'Carbamohydroxamic acid' OR 'Carbamohydroximic acid' OR 'Carbamohydroxyamic acid' OR 'Carbamoyl oxime' OR 'Carbamyl hydroxamate' OR 'Carbomohydroxamic acid' OR 'Carrbamoyl Oxime' OR 'Cytodrox' OR 'droxia' OR 'Hidrix' OR 'Hidroxicarbamida' OR 'hydab' OR 'hydrea' OR 'Hydreia' OR 'hydrine' OR 'Hydroxicarbamidum' OR 'hydroxy carbamide' OR 'hydroxy urea' OR 'hydroxyaminomethanamide' OR 'Hydroxycarbamid' OR 'hydroxycarbamide' OR 'Hydroxycarbamidum' OR 'Hydroxycarbamine' OR 'hydroxyl urea' OR 'Hydroxylurea' OR 'Hydroxyurea' OR 'Hydura' OR 'Hydurea' OR 'Idrossicarbamide' OR 'Litaler' OR 'litalir' OR 'mylocel' OR 'N-(Aminocarbonyl) Hydroxyamine' OR 'N-(Aminocarbonyl)hydroxylamine' OR 'N-Carbamoylhydroxylamine' OR 'neodrea' OR 'nsc 32065' OR 'NSC32065' OR 'oncocarbide' OR 'onco-carbide' OR 'oxycarbamide' OR 'oxyrea' OR 'oxyurea' OR 'siklos'):ab,de,kw,lnk,rn,ti,tn OR **'valproic acid'/exp** OR ('614OI1Z5WI' OR '99-66-1' OR '2 propylpentanoate' OR '2 propylpentanoic acid' OR '2 propylvalerate sodium' OR '2 propylvaleric acid' OR '2, 2 dipropyl acetic acid' OR '2-propyl-Pentanoate' OR '2-Propylpentanoic Acid' OR '2-PROPYL-PENTANOIC ACID' OR '2-PropylpentanoicAcid' OR '2-Propylvaleric acid' OR '4-Heptanecarboxylic acid' OR 'absenor' OR 'Acidum valproicum' OR 'alpha propylvalerate' OR 'alpha propylvaleric acid' OR 'apilepsin' OR 'atemperator' OR 'Avugane' OR 'Baceca' OR 'convulex' OR 'Convulsofin' OR 'delepsine' OR 'depacon' OR 'depakene' OR 'depakin' OR 'depakine' OR 'Depakote' OR 'depalept' OR 'deprakine' OR 'Deproic' OR 'di n propylacetate' OR 'di n propylacetic acid' OR 'di-n-propyl acetic acid' OR 'diplexil' OR 'Dipropyl Acetate' OR 'dipropyl acetic acid' OR 'dipropylacetate' OR 'dipropylacetatic acid' OR 'dipropylacetic acid' OR 'diprosin' OR 'Divalproex' OR 'Encorate' OR 'Epical' OR 'epilam' OR 'epilex' OR 'epilim' OR 'episenta' OR 'Epival' OR 'ergenyl' OR 'espa valept' OR 'Eurekene' OR 'everiden' OR 'goilim' OR 'hexaquin' OR 'kw 6066 n' OR 'labazene' OR 'leptilan' OR 'leptilanil' OR 'micropakine' OR 'mylproin' OR 'myproic acid' OR 'n dipropylacetic acid' OR 'orfil' OR 'orfiril' OR 'orlept' OR 'petilin' OR 'Propylisopropylacetic Acid' OR 'Propylvaleric acid' OR 'propymal' OR 'Savicol' OR 'sodium 2 propylpentanoate' OR 'sodium 2 propylvalerate' OR 'sodium di n propyl acetate' OR 'sodium di n propylacetate' OR 'sodium dipropyl acetate' OR 'sodium dipropylacetate' OR 'sodium n dipropylacetate' OR 'stavzor' OR 'valberg pr' OR 'valcote' OR 'Valdisoval' OR 'valepil' OR 'valeptol' OR 'valerin' OR 'valhel pr' OR 'valoin' OR 'valpakine' OR 'valparin' OR 'valporal' OR 'valprax' OR 'valpro' OR 'valproate' OR 'valprodura' OR 'Valproic Acid' OR 'valprosid' OR 'valprotek' OR 'valsup' OR 'Vupral'):ab,de,kw,lnk,rn,ti,tn OR **'retinoic acid'/exp** OR ('5688UTC01R' OR '302-79-4' OR '1 (8 carboxy 3, 7 dimethyl 1, 3, 5, 7 octatetraen 1 yl) 2, 6, 6 trimethyl 1 cyclohexene' OR '3, 7 dimethyl 9 (2, 6, 6 trimethyl 1 cyclohexen 1 yl) 2, 4, 6, 8 nonatetraenoic acid' OR '3, 7 dimethyl 9 (2, 6, 6 trimethyl 1 cyclohexen 1 yl) nona 2, 4, 6, 8 tetraen 1 oic acid' OR '3,7-Dimethyl-9-(2,6,6-trimethyl-1-cyclohexen-1-yl)-2,4,6,8-nonatetraenoic acid' OR '3,7-Dimethyl-9-(2,6,6-trimethyl-1-cyclohexene-1-yl)-2,4,6,8-nonatetraenoic acid' OR '3,7-dimethyl-9-(2,6,6-trimethyl-1-cyclohexenyl)nona-2,4,6,8-tetraenoic acid' OR '3,7-Dimethyl-9-(2,6,6-trimethylcyclohex-1-enyl)nona-2,4,6,8-all-trans-tetraenoic acid' OR '9-cis-RA' OR 'Aberel' OR 'Aberela' OR 'acid a vit' OR 'Acnavit' OR 'Airol' OR 'Aknefug' OR 'Aknoten' OR 'all-trans-Vitamin A1 acid' OR 'alquingel' OR 'alten' OR 'altinac' OR 'anhydroretinoic acid' OR 'ar 623' OR 'ar623' OR 'atra' OR 'atragen' OR 'atralin' OR 'avita' OR 'avitcid' OR 'Avitoin' OR 'betarretin' OR 'dermairol' OR 'dermik a' OR 'effederm' OR 'epi aberel' OR 'epiaberel' OR 'eudyna' OR 'facenol' OR 'ilotycin-a' OR 'locacid' OR 'Nexret' OR 'nsc 122758' OR 'nsc122758' OR 'prosome a cream' OR 'reacel-a' OR 'Refissa' OR 'Renova' OR 'Retacnyl' OR 'retavit' OR 'retiderma' OR 'Retin A' OR 'Retinoate' OR 'Retinoic acid' OR 'Retinova' OR 'Retionic acid' OR 'Retisol-A' OR 'retrieve cream' OR 'ro 01 5488' OR 'ro 1 5488' OR 'ro 15488' OR 'ro015488' OR 'ro15488' OR 'stieva a' OR 'stievaa' OR 'tracne' OR 'Trans-Retinoicacid' OR 'trentin' OR 'Tretin M' OR 'tretinoin' OR 'Tretinoinum' OR 'TRETINON' OR 'Vesanoid' OR 'Vitamin A acid' OR 'vitinoin'):ab,de,kw,lnk,rn,ti,tn OR **'retinol'/exp** OR ('11103-57-4' OR '68-26-8' OR '3, 7 dimethyl 9 (2, 6, 6 trimethyl 1 cyclohexen 1 yl) 2, 4, 6, 8 nonatetraen 1 ol' OR '3, 7 dimethyl 9 (2, 6, 6 trimethyl 1 cyclohexenyl) 2, 4, 6, 8 nonatetraen 1 ol' OR '3,7-Dimethyl-9-(2,6,6-trimethyl-1-cyclchexen-1-yl)-2,4,6,8-nonatetraen-1-ol' OR '3,7-Dimethyl-9-(2,6,6-trimethyl-1-cyclohexen-1-yl)-2,4,6,8-nonate-traen-1-ol' OR '3,7-Dimethyl-9-(2,6,6-trimethyl-1-cyclohexen-1-yl)-2,4,6,8-nonatetraen-1-ol, (all-E)-' OR '3,7-Dimethyl-9-(2,6,6-trimethyl-1-cyclohexen-1-yl)-2,4,6,8-nonatetraen-1-ol, all (E)-' OR '3,7-Dimethyl-9-(2,6,6-trimethyl-1-cyclohexenyl)-2,4,6,8-nonatetraen-1-ol' OR '3,7-dimethyl-9-(2,6,6-trimethyl-1-cyclohexenyl)-nona-2,4,6,8-tetraen-1-ol' OR 'a 313' OR 'a mulsal' OR 'a mulsin' OR 'a mulsine' OR 'a sol' OR 'a vi pel' OR 'a vitadit' OR 'a vitan' OR 'a313' OR 'acrisina' OR 'acrisine' OR 'actifral a' OR 'adatone' OR 'Afaxin' OR 'afaxine' OR 'afilina' OR 'afiline' OR 'agiolan' OR 'Agoncal' OR 'alcovit a' OR 'alfa monovite' OR 'alfaergin' OR 'alfaergine' OR 'alfamin' OR 'alfamine' OR 'alfamonovit' OR 'alfasir' OR 'alfasole' OR 'alfasterolo' OR 'alfatar' OR 'alfavena' OR 'alfavene' OR 'alfavitina' OR 'alfavitine' OR 'alfene' OR 'alin' OR 'all-trans-Retinyl alcohol' OR 'Alphalin' OR 'alphaline' OR 'alphasterol' OR 'amulsal' OR 'A-Mulsal' OR 'amulsin' OR 'amulsine' OR 'amulvit' OR 'Anatola' OR 'anavit' OR 'Anti-infective vitamin' OR 'Antixerophthalmic vitamin' OR 'Aoral' OR 'apexol' OR 'Apostavit' OR 'Aquasol A' OR 'Aquasola' OR 'Aquasynth' OR 'arcavit A' OR 'asol' OR 'A-Sol' OR 'asteril' OR 'Atars' OR 'aterapion' OR 'Avibon' OR 'avimin' OR 'avimine' OR 'avipel' OR 'A-Vi-Pel' OR 'avipur' OR 'avitabiol' OR 'avitadit' OR 'avital' OR 'avitaminum kolin' OR 'avitan' OR 'A-Vitan' OR 'avitana' OR 'avitane' OR 'avite' OR 'avitil' OR 'avitina' OR 'Avitol' OR 'avogina' OR 'avogine' OR 'avoleum' OR 'axerodina' OR 'axerodine' OR 'axerol' OR 'axerophthol' OR 'Axerophtholum' OR 'axerophthylium' OR 'bentavit a' OR 'bentavite a' OR 'biosterol' OR 'biotan' OR 'chivibit a' OR 'Chocola A' OR 'Cylasphere' OR 'cytobiase' OR 'dagravit a' OR 'davitamon a' OR 'difvitamin a' OR 'Disatabs Tabs' OR 'Dofsol' OR 'Dohyfral A' OR 'elageno a' OR 'endo a' OR 'envit a' OR 'Epiteliol' OR 'fletase' OR 'gadeol' OR 'gadol' OR 'halivitan' OR 'halivitane' OR 'homagenets aoral' OR 'Homagenets aorl' OR 'hydrosol' OR 'Hydrovit A' OR 'ido a' OR 'idratene' OR 'inovitan a' OR 'Lard Factor' OR 'meditalfa' OR 'mulsal a' OR 'multamine' OR 'oleovit a' OR 'Oleovitamin A' OR 'ophthalamin' OR 'panvita' OR 'Plivit A' OR 'Prepalin' OR 'prepaline' OR 'preparato a' OR 'primavit' OR 'quotivit' OR 'Retin-11,12-t2-ol (9CI)' OR 'retinol' OR 'Retinolo' OR 'Retinolum' OR 'Retinyl A' OR 'retinyl alcohol' OR 'Retrovitamin A' OR 'ro a vit' OR 'Rovimix A 500' OR 'Sehkraft A' OR 'Tegosphere VitA' OR 'Testavol' OR 'Thalasphere' OR 'ucemine a' OR 'vaconex' OR 'Vaflol' OR 'Vafol' OR 'Veroftal' OR 'viadenin' OR 'vialpha' OR 'Vi-Alpha' OR 'viatate' OR 'vidoma' OR 'vitadone' OR 'vitadral' OR 'vitalen a' OR 'vitalfa' OR 'vitama' OR 'Vitamin A' OR 'Vitamin A1' OR 'Vitamine A' OR 'Vitaminum A' OR 'vitaplex a' OR 'vitapur a' OR 'vitasan a' OR 'Vitavel A' OR 'vitpex' OR 'Vogan' OR 'wandervit a' OR 'xerophthol' OR 'Zinosan N'):ab,de,kw,lnk,rn,ti,tn OR **'aminopterin'/exp** OR ('JYB41CTM2Q' OR '54-62-6' OR '4 amino 4 deoxyfolic acid' OR '4 amino 4 desoxyfolic acid' OR '4 amino 9 methylpteroylglutamic acid' OR '4 aminofolic acid' OR '4 aminomethylpteroylglutamic acid' OR '4 aminopteroylglutamic acid' OR '4-Amino-4-deoxypteroylglutamate' OR '4-Aminofolate' OR '4-Aminopteroyl- glutamic acid' OR '4-Aminopteroyl-R-glutamic acid' OR '4-Aminopteroylglutamate' OR '4-Aminopteroylglutamic acid' OR '4-Aminopteroyl-glutamic acid' OR 'aminopterin' OR 'Aminopterine' OR 'Aminopterinum' OR 'Aminotrexate' OR 'n [para [ (2, 4 diaminopterid 6 ylmethyl) amino] benzoyl] glutamic acid' OR 'nsc 739' OR 'NSC739' OR 'Pteramina'):ab,de,kw,lnk,rn,ti,tn OR **'methotrexate'/exp** OR ('YL5FZ2Y5U1' OR '59-05-2' OR '4 amino 10 methylfolic acid' OR '4 amino 10 methylpteroylglutamic acid' OR '4 amino n10 methylpteroylglutamic acid' OR '4-Aminomethylpteroylglutamic acid' OR '4-amino-N(10)-methylpteroylglutamic acid' OR '4-Amino-N(sup 10)-methylpteroylglutamic acid' OR '4-Amino-N10-methylpteroyl-L-glutamic acid' OR 'a methopterine' OR 'Abitrexate' OR 'amethopterin' OR 'A-Methopterin' OR 'amethopterine' OR 'A-Methpterin' OR 'ametopterine' OR 'Antifolan' OR 'Arbitrexate' OR 'biotrexate' OR 'Brimexate' OR 'canceren' OR 'CL 14377' OR 'cl14377' OR 'Emtexate' OR 'emthexat' OR 'emthexate' OR 'emtrexate' OR 'enthexate' OR 'farmitrexat' OR 'farmitrexate' OR 'farmotrex' OR 'Fauldexato' OR 'Folex' OR 'ifamet' OR 'intradose MTX' OR 'jylamvo' OR 'Lantarel' OR 'ledertrexate' OR 'Lumexon' OR 'maxtrex' OR 'Medsatrexate' OR 'Metatrexan' OR 'metex' OR 'methoblastin' OR 'methohexate' OR 'Methotextrate' OR 'methotrate' OR 'Methotrexat' OR 'methotrexate' OR 'methotrexato' OR 'Methotrexatum' OR 'methoxtrexate' OR 'methrotrexate' OR 'Methylaminopterin' OR 'methylaminopterine' OR 'Methylaminopterinum' OR 'meticil' OR 'metoject' OR 'Metolate' OR 'metothrexate' OR 'Metotressato' OR 'metotrexat' OR 'metotrexate' OR 'metotrexin' OR 'metrex' OR 'Metrotex' OR 'Mexate' OR 'mpi 5004' OR 'mpi5004' OR 'MTX hydrate' OR 'n [4 [ (2, 4 diamino 6 pteridylmethyl) methylamino] benzoyl] glutamic acid' OR 'N-(4-(((2,4-DIAMINO-6-PTERIDINYL)METHYL)METHYLAMINO)BENZOYL)L-GLUTAMIC ACID' OR 'N-(4-(((2,4-Diamino-6-pteridinyl)methyl)methylamino)benzoyl)-L-glutamicacid' OR 'N-[4-[[(2,4-Diamino-6-pteridinyl)methyl] methylamino]benzoyl]-L-glutamic acid' OR 'N-[4-[[(2,4-diamino-6-pteridinyl)methyl]methylamino]benzoyl]-L-glutamic acid' OR 'N-Bismethylpteroylglutamic acid' OR 'neotrexate' OR 'nordimet' OR 'novatrex' OR 'nsc 740' OR 'NSC740' OR 'Otrexup' OR 'rasuvo' OR 'reumatrex' OR 'Rheumatrex' OR 'Texate' OR 'texorate' OR 'Tremetex' OR 'trexall' OR 'Trexeron' OR 'Trixilem' OR 'xaken' OR 'Xatmep' OR 'zexate'):ab,de,kw,lnk,rn,ti,tn OR **'phenytoin'/exp** OR ('6158TKW0C5' OR '57-41-0' OR '630-93-3' OR '5, 5 diphenyl 2, 4 imidazolidinedione' OR '5, 5 diphenylglycolylurea' OR '5, 5 diphenylhydantoin' OR '5, 5 diphenylimidazoline 2, 4 dione' OR '5,5-di(phenyl)imidazolidine-2,4-dione' OR '5,5-diphenyl hydantoin' OR '5,5-Diphenyl-2,4-imidazolidinedione' OR '5,5-Diphenylhydantoin' OR '5,5-Diphenylimidazolidin-2,4-dione' OR '5,5-diphenylimidazolidine-2,4-dione' OR '5,5-Diphenyl-imidazolidine-2,4-dione' OR 'alepsin' OR 'aleviatin' OR 'antilepsin' OR 'Antisacer' OR 'Auranile' OR 'cansoin' OR 'Causoin' OR 'Citrullamon' OR 'Citrulliamon' OR 'Comital' OR 'Comitoina' OR 'Convul' OR 'cumatil' OR 'Danten' OR 'Dantinal' OR 'dantoin' OR 'Dantoinal' OR 'Dantoine' OR 'denyl' OR 'Difenin' OR 'difetoin' OR 'differenin' OR 'difhydan' OR 'Dihycon' OR 'dihydan' OR 'di-hydan' OR 'Dihydantoin' OR 'Dilabid' OR 'Dilantin' OR 'Dilantine' OR 'Dillantin' OR 'dintoin' OR 'dintoina' OR 'Diphantoin' OR 'diphantoine' OR 'Diphedal' OR 'diphedan' OR 'Diphenat' OR 'Diphenin' OR 'Diphenine' OR 'Diphentoin' OR 'Diphentyn' OR 'diphenyl hydantoin' OR 'Diphenylan' OR 'diphenyldantoin' OR 'Diphenylhydantoin' OR 'Diphenylhydatanoin' OR 'diphenytoin' OR 'Di-Phetine' OR 'ditoin' OR 'Ditoinate' OR 'ditomed' OR 'Elepsindon' OR 'Enkelfel' OR 'Epamin' OR 'Epanutin' OR 'Epdantoin' OR 'Epdantoine simple' OR 'Epelin' OR 'Epifenyl' OR 'Epihydan' OR 'Epilan D' OR 'Epilantin' OR 'epileptin' OR 'Epinat' OR 'Episar' OR 'Epised' OR 'Epsolin' OR 'Eptal' OR 'Eptoin' OR 'felantin' OR 'fenantoin' OR 'Fenidantoin s' OR 'Fenigramon' OR 'Fenitoin' OR 'Fentoin' OR 'Fenylepsin' OR 'fenytoin' OR 'Fenytoine' OR 'Hidan' OR 'hidanil' OR 'Hidantal' OR 'Hidantilo' OR 'Hidantina' OR 'Hidantomin' OR 'Hindatal' OR 'Hydantal' OR 'Hydantin' OR 'hydantinal' OR 'Hydantoinal' OR 'Hydantol' OR 'Ictalis simple' OR 'Idantoil' OR 'Idantoin' OR 'Iphenylhydantoin' OR 'Kessodanten' OR 'Labopal' OR 'Lehydan' OR 'lepitoin' OR 'Lepsin' OR 'Minetoin' OR 'Neos-Hidantoina' OR 'Neosidantoina' OR 'neosidantoina' OR 'Novantoina' OR 'Novophenytoin' OR 'Om hidantoina simple' OR 'Om-Hydantoine' OR 'Oxylan' OR 'Phanantin' OR 'Phanatine' OR 'Phenatine' OR 'Phenatoine' OR 'Phenhydan' OR 'phenhydane' OR 'Phenhydanin' OR 'phenilep' OR 'Phenitoin' OR 'Phentoin' OR 'Phentytoin' OR 'phenybin' OR 'phenydan' OR 'phenydantin' OR 'phenytek' OR 'phenytex' OR 'phenytoin' OR 'Phenytoinum' OR 'phenytonium' OR 'pyoredol' OR 'Ritmenal' OR 'Saceril' OR 'sanepil' OR 'Silantin' OR 'Sinergina' OR 'Sodanthon' OR 'Sodantoin' OR 'sodanton' OR 'Sodium Diphenylhydantoinate' OR 'Solantin' OR 'Solantoin' OR 'solantyl' OR 'Sylantoic' OR 'Tacosal' OR 'Thilophenyl' OR 'Toin unicelles' OR 'vasilcon' OR 'Zentronal' OR 'Zentropil'):ab,de,kw,lnk,rn,ti,tn OR **'ethylene glycol'/exp** OR ('FC72KVT52F' OR '107-21-1' OR '1, 2 ethanediol' OR '1,2-dihydroxy ethane' OR '1,2-Dihydroxyethane' OR '1,2-Ethandiol' OR '1,2-ethane diol' OR '1,2-ethanediol' OR '1,2-ethyleneglycol' OR '2-hydroxyethanol' OR 'ethan-1,2-diol' OR 'Ethane-1,2-diol' OR 'ethane-1.2-diol' OR 'ethanediol' OR 'ethyl glycol' OR 'ethylen glycol' OR 'Ethylene alcohol' OR 'Ethylene dihydrate' OR 'Ethylene Glycol' OR 'ethyleneglycol' OR 'Ethylenglycol' OR 'Etilenglicol' OR 'etylene glycol' OR 'Glycol alcohol' OR 'glycol ethylene' OR 'Hypodicarbonous acid' OR 'Mono Ethylene Glycol' OR 'monoethylene glycol'):ab,de,kw,lnk,rn,ti,tn OR **'thiotepa'/exp** OR ('905Z5W3GKH' OR '52-24-4' OR 'AI3-24916' OR 'AI324916' OR 'Girostan' OR 'Ledertepa' OR 'methylenethiophosphoramide' OR 'n, n, n triethylenethiophosphoramide' OR 'NSC 6396' OR 'nsc 6996' OR 'NSC6396' OR 'Oncotepa' OR 'Oncothio-tepa' OR 'oncotiotepa' OR 'Phosphoric tri(ethyleneamide)' OR 'Phosphorothioic acid triethylenetriamide' OR 'PHOSPHOROTHIOIC TRI(ETHYLENEAMIDE)' OR 'Stepa' OR 'tepadina' OR 'tespa' OR 'Tespamin' OR 'Tespamine' OR 'thio tepa' OR 'Thiofozil' OR 'Thiophosphamide' OR 'Thiophosphamidum' OR 'Thioplex' OR 'Thiotef' OR 'Thio-Tep' OR 'thiotepa' OR 'Thiotepum' OR 'thiotriethylenephosphoramide' OR 'Tifosyl' OR 'tio tef' OR 'Tiofosfamid' OR 'Tiofosyl' OR 'Tiofozil' OR 'trethylenethiophosphoramide' OR 'Tri(1-aziridinyl)phosphine sulfide' OR 'Tri(aziridin-1-yl)phosphine sulfide' OR 'Tri(ethyleneimino)thiophosphoramide' OR 'Tri-1-aziridinylphosphine sulfide' OR 'Triaziridinylphosphine sulfide' OR 'triethylene thio phosphoramide' OR 'triethylene thiophosphamide' OR 'triethylene thiophosphoramide' OR 'triethylenethiophosphamide' OR 'Triethylenethiophosphoramide' OR 'Triethylenethiophosphorotriamide' OR 'triethylenethiophosphortriamide' OR 'tris (1 azaridinyl) phosphine sulfide' OR 'tris (1 aziridinyl) phosphine sulfide' OR 'tris (1 aziridinyl) phosphine sulphide' OR 'tris (1 aziridinyl) phosphinsulfide' OR 'tris (1 aziridinyl) phosphinsulphide' OR 'tris (ethylenimino) thiophosphate' OR 'Tris(1-aziridinyl)phosphine sulfide' OR 'Tris(1-aziridinyl)phosphine sulphide' OR 'tris(1-aziridinyl)-sulfanylidenephosphorane' OR 'tris(aziridin-1-yl)-sulfanylidenephosphorane' OR 'Tris(aziridinyl)phosphine sulfide' OR 'Tris(aziridinyl)-phosphine sulfide' OR 'Tris(ethylenimino)thiophosphate'):ab,de,kw,lnk,rn,ti,tn OR **'atrazine'/exp** OR ('QJA9M5H4IM' OR '1912-24-9' OR '2 chloro 4 ethylamino 6 isopropylamino 1, 3, 5 triazine' OR '2 chloro 4 ethylamino 6 isopropylamino s triazine' OR '2-Chloro-4-(ethylamino)-6-(isopropylamino)-1,3,5-triazine' OR '2-Chloro-4-(ethylamino)-6-(isopropylamino)-s-triazine' OR '2-Chloro-4-(ethylamino)-6-(isopropylamino)triazine' OR '2-Chloro-4-(ethylamino)-6-[(prop-2-yl)amino]-1,3,5-triazine' OR '2-Chloro-4-ethylamineisopropylamine-s-triazine' OR '2-Chloro-4-ethylamino-6-isopropylamino-1,3,5-triazine' OR '2-Chloro-4-ethylamino-6-isopropylamino-s-triazine' OR '2-chloro-4-ethyl-amino-6-isopropylamino-s-triazine' OR '2-CHLORO-4-ETHYLAMINO-6-ISOPROPYLAMINO-sym-TRIAZINE' OR '6 chloro n2 ethyl n4 isopropyl 1, 3, 5 triazine 2, 4 diamine' OR '6-Chloro-N2-ethyl-N4-isopropyl-1,3,5-triazine-2,4-diamine' OR 'Aatram' OR 'Aatrex' OR 'Actinite PK' OR 'Akticon' OR 'Aktikon' OR 'Aktinit A' OR 'Aktinit PK' OR 'Aneldazin' OR 'Argezin' OR 'Atazinax' OR 'Atraflow' OR 'Atranex' OR 'Atrasine' OR 'Atrataf' OR 'Atratol' OR 'Atrazin' OR 'atrazine' OR 'atrazinus' OR 'Atred' OR 'Atrex' OR 'Attrex' OR 'Azinotox 500' OR 'Azoprim' OR 'Candex' OR 'Ceasin 50' OR 'Cekuzina-T' OR 'Chromozin' OR 'Crisamina' OR 'Crisatrina' OR 'Crisazina' OR 'Crisazine' OR 'Cyazin' OR 'Cyazine' OR 'Farmozine' OR 'Fenamin' OR 'Fenamine' OR 'Fenatrol' OR 'Fogard' OR 'Gesamprim' OR 'Gesaprim' OR 'Gesaprin' OR 'Gesoprim' OR 'Griffex' OR 'Herbatoxol' OR 'Hungazin' OR 'Inakor' OR 'Laddock' OR 'Maizina' OR 'Mebazine' OR 'Oleogesaprim' OR 'Pitezin' OR 'Primatol' OR 'Primaze' OR 'Primitol A' OR 'Primoleo' OR 'Radazin' OR 'Radizin' OR 'Radizine' OR 'Strazine' OR 'Triazine A 1294' OR 'Vectal' OR 'Weedex A' OR 'Wonuk' OR 'Zeapho' OR 'Zeaphos' OR 'Zeapos' OR 'Zeazin' OR 'Zeazine' OR 'zeazint' OR 'Zeopos'):ab,de,kw,lnk,rn,ti,tn OR **'dinoseb'/exp** OR ('88-85-7' OR '2 (1 methylpropyl) 4, 6 dinitrophenol' OR '2 sec butyl 4, 6 dinitrophenol' OR '2-(1-Methylpropyl)-4,6-dinitrophenol' OR '2-(1-Methylpropyl)-4,6-dinitro-Phenol' OR '2-(sec-Butyl)-4,6-dinitrophenol' OR '2, 4 dinitro 6 sec butylphenol' OR '2,4-Dinitro-6-sec-butylphenol' OR '2,4-dinitro-6-sec-butyl-phenol' OR '2-[1-methylpropyl]-4,6-dinitrophenol' OR '2-sec-Butyl-4,6-dinitrophenol' OR '2-Sec-butyl-4,6-dinitro-Phenol' OR '4, 6 dinitro 2 (1 methylpropyl) phenol' OR '4, 6 dinitro 2 sec butylphenol' OR '4,6-Dinitro-2-(1-methyl-n-propyl)phenol' OR '4,6-Dinitro-2-(1-methyl-propyl)phenol' OR '4,6-Dinitro-2-sec-butylphenol' OR '4,6-Dinitro-o-sec-butylphenol' OR '6 sec butyl 2, 4 dinitrophenol' OR '6-sec-Butyl-2,4-dinitrophenol' OR 'Aatox' OR 'Aretit' OR 'Basanite' OR 'Blaartox' OR 'Butaphene' OR 'Caldon' OR 'Chemsect' OR 'Desicoil' OR 'Dibutox' OR 'Dinitrall' OR 'Dinitrax' OR 'dinitrobutylphenol' OR 'Dinitro-ortho-sec-butyl phenol' OR 'Dinitro-o-sec-butylphenol' OR 'dinoseb' OR 'Dynanap' OR 'Dytop' OR 'Elgetol 318' OR 'Gebutox' OR 'Hivertox' OR 'Ivosit' OR 'Kiloseb' OR 'Ladob' OR 'Laseb' OR 'Nitropone C' OR 'Phenotan' OR 'Premerg' OR 'Premerge' OR 'Sinox general' OR 'Sparic' OR 'Spurge' OR 'Subitex' OR 'Tubotox'):ab,de,kw,lnk,rn,ti,tn OR **'fluazinam'/exp** OR ('0P91PCK33Q' OR '79622-59-6' OR '3-Chloro-N-(3-chloro-2,6-dinitro-4-(trifluoromethyl)phenyl)-5-(trifluoromethyl)-2-pyridinamine' OR '3-Chloro-N-(3-chloro-2,6-dinitro-4-(trifluoromethyl)phenyl)-5-(trifluoromethyl)pyridin-2-amine' OR '3-chloro-N-(3-chloro-2,6-dinitro-4-trifluoromethylphenyl)-5-trifluoromethyl-2-pyridinamine' OR '3-Chloro-N-(3-chloro-2,6-dinitro-4-trifluoromethylphenyl)-5-trifluoromethyl-2-pyridylamine' OR '3-chloro-N-[3-chloro-2,6-dinitro-4-(trifluoromethyl)phenyl]-5-(trifluoromethyl)-2-pyridinamine' OR '3-chloro-N-[3-chloro-2,6-dinitro-4-(trifluoromethyl)phenyl]-5-(trifluoromethyl)pyridin-2-amine' OR 'Altima' OR 'Fluazinam' OR 'Fluaziname' OR 'Mapro' OR 'Sekoya' OR 'Shirlan'):ab,de,kw,lnk,rn,ti,tn OR **'myclobutanil'/exp** OR ('B6T1JTM6KZ' OR '88671-89-0' OR '(R)-2-p-chlorophenyl-2-(1H-1,2,4-triazol-1-ylmethyl)hexanenitrile' OR '.alpha.-Butyl-.alpha.-(4-chlorophenyl)-1H-1,2,4-triazole-1-propanenitrile' OR '2 (4 chlorophenyl) 2 (1h 1, 2, 4 triazol 1 ylmethyl) hexanenitrile' OR '2-(4-chlorophenyl)-2-(1,2,4-triazol-1-ylmethyl)hexanenitrile' OR '2-(4-Chlorophenyl)-2-(1H-1,2,4-triazol-1-ylmethyl)hexanenitrile' OR '2-p-Chlorophenyl-2-(1H-1,2,4-triazol-1-ylmethyl)hexanenitrile' OR 'alpha butyl alpha (4 chlorophenyl) 1h 1, 2, 4 triazole 1 propanenitrile' OR 'alpha-Butyl-alpha-(4-chlorophenyl)-1H-1,2,4-triazole-1-propanenitrile' OR 'alpha-n-butyl-alpha(4-chlorophenyl)-1H-1,2,4-triazole-1-propanenitrile' OR 'alpha-n-butyl-alpha-(4-chlorophenyl)-1H-1,2,4-triazole-1-propanenitrile' OR 'myclobutanil' OR 'Synthane 12E' OR 'Systhane'):ab,de,kw,lnk,rn,ti,tn OR **'ochratoxin'/exp** OR ('1779SX6LUY' OR '303-47-9' OR 'ochratoxin A' OR 'ochratoxine a'):ab,de,kw,lnk,rn,ti,tn OR **'spiroxamine'/exp** OR ('OUT5YHB7BO' OR '118134-30-8' OR '(8-tert-Butyl-1,4-dioxa-spiro[4.5]dec-2-ylmethyl)-ethyl-propyl-amine' OR '8-tert-butyl-1,4-dioxaspiro(4.5)decan-2-ylmethyl(ethyl)(propyl)amine' OR 'Spiroxamine'):ab,de,kw,lnk,rn,ti,tn OR **'thiacloprid'/exp** OR ('DSV3A944A4' OR '111988-49-9' OR '(3-((6-Chloro-3-pyridinyl)methyl)-2-thiazolidinylidene)cyanamide' OR '[3 (6 chloro 3 pyridinylmethyl) 2 thiazolidinylidene] cyanamide' OR '[3 [ (6 chloro 3 pyridinyl) methyl] 2 thiazolidinylidene] cyanamide' OR '[3 [ (6 chloropyridin 3 yl) methyl] 1, 3 thiazolidin 2 ylidene] cyanamide' OR '[3-[(6-chloranylpyridin-3-yl)methyl]-1,3-thiazolidin-2-ylidene]cyanamide' OR '[3-[(6-chloro-3-pyridinyl)methyl]-2-thiazolidinylidene]cyanamide' OR '[3-[(6-chloro-3-pyridyl)methyl]thiazolidin-2-ylidene]cyanamide' OR '[3-[(6-chloropyridin-3-yl)methyl]-1,3-thiazolidin-2-ylidene]cyanamide' OR '{3-[(6-chloropyridin-3-yl)methyl]-1,3-thiazolidin-2-ylidene}cyanamide' OR '3-((6-chloro-3-pyridinyl)methyl)-2-thiazolidinylidene cyanamide' OR 'Thiacloprid' OR 'thiaclopride'):ab,de,kw,lnk,rn,ti,tn OR **'thiram'/exp** OR ('0D771IS0FH' OR '137-26-8' OR '16c tetramethylthiuram disulfide' OR 'Aapirol' OR 'Aatiram' OR 'Accel TMT' OR 'Aceto TETD' OR 'Akrochem TMTD' OR 'Anles' OR 'Arasan' OR 'Atiram' OR 'Aules' OR 'Basultra' OR 'Betoxin' OR 'bis (dimethyl thiocarbamoyl) disulfide' OR 'bis (dimethylthiocarbamoyl) disulfide' OR 'Bis(dimethyl thiocarbamoyl)disulfide' OR 'Bis(dimethylthiocarbamoyl) disulfide' OR 'Bis(dimethylthiocarbamoyl) disulphide' OR 'Bis(dimethylthiocarbamyl) disulfide' OR 'bis[Dimethylthiocarbamyl] disulfide' OR 'Cunitex' OR 'Cyuram DS' OR 'Delsan' OR 'Ekagom TB' OR 'Falitiram' OR 'Fermide' OR 'Fernacol' OR 'Fernasan' OR 'Fernide' OR 'Formalsol' OR 'Granuflo' OR 'Hermal' OR 'Hermat TMT' OR 'Heryl' OR 'Hexathir' OR 'Kregasan' OR 'Mercuram' OR 'Methyl thiuramdisulfide' OR 'Methyl tuads' OR 'Methylthiuram disulfide' OR 'Metiur' OR 'Metiurac' OR 'Nobecutan' OR 'Nocceler TT' OR 'Nomersan' OR 'Normersan' OR 'NSC 1771' OR 'NSC1771' OR 'Panoram 75' OR 'Polyram ultra' OR 'Pomarsol' OR 'Pomasol' OR 'puralin' OR 'Radothiram' OR 'Rezifilm' OR 'rhenogran' OR 'Robac TMT' OR 'Sadoplon' OR 'Spotrete' OR 'Sranan-sf-X' OR 'Teramethylthiuram disulfide' OR 'Tersan' OR 'Tersantetramethyldiurane sulfide' OR 'tetra methyl thiuram disulfide' OR 'tetramethyl tetramethylthiuram disulfide' OR 'tetramethyl thiuram disulfide' OR 'Tetramethyl thiuramdisulfide' OR 'Tetramethyl thiurane disulfide' OR 'Tetramethyl thiurane disulphide' OR 'Tetramethyldiurane sulphite' OR 'Tetramethylenethiuram disulfide' OR 'Tetramethylenethiuram disulphide' OR 'Tetramethylthiocarbamoyldisulphide' OR 'Tetramethylthioperoxydicarbonic diamide' OR 'Tetramethylthioramdisulfide' OR 'Tetramethylthiouram disulfide' OR 'Tetramethylthiuram' OR 'tetramethylthiuramdisulfide' OR 'tetramethylthiuramidisulfide' OR 'Tetramethylthiuran disulphide' OR 'Tetramethylthiurane disulfide' OR 'Tetramethylthiurum disulfide' OR 'Tetramethylthiurum disulphide' OR 'Tetrapom' OR 'Tetrasipton' OR 'tetrathion' OR 'tetrathione' OR 'tetrathionine' OR 'Tetrathiuram disulfide' OR 'Tetrathiuram disulphide' OR 'Thianosan' OR 'Thillate' OR 'Thimar' OR 'Thimer' OR 'thiosan' OR 'Thioscabin' OR 'Thiotex' OR 'Thiotox' OR 'thiram' OR 'Thiramad' OR 'Thirampa' OR 'Thiramum' OR 'Thirasan' OR 'Thiulin' OR 'Thiulix' OR 'Thiurad' OR 'Thiuram' OR 'Thiuramin' OR 'thiuramyl' OR 'Thylate' OR 'Tiradin' OR 'tiram' OR 'tiramo' OR 'Tirampa' OR 'tiuramyl' OR 'TMT Disulfide' OR 'TMTD' OR 'TMTDS' OR 'Trametan' OR 'Tridipam' OR 'Tripomol' OR 'tuad' OR 'TUEX' OR 'Tulisan' OR 'Tutan' OR 'Tyradin' OR 'Vancide TM' OR 'Vulcafor TMT' OR 'Vulkacit TH' OR 'Vulkazam S'):ab,de,kw,lnk,rn,ti,tn OR **'triadimefon'/exp** OR ('43121-43-3' OR '1 (4 chlorophenoxy) 3, 3 dimethyl 1 (1, 2, 4 triazol 1 yl) 2 butanone' OR '1-(1,2,4-triazolyl)-1-(4-chlorophenoxy)-3,3-dimethylbutan-2-one' OR '1-(1,2,4-Triazoyl-1)-1-(4-chloro-phenoxy)-3,3-dimethylbutanone' OR '1-(4-chlorophenoxy)-3,3-dimethyl-1-(1,2,4-triazol-1-yl)butan-2-one' OR '1-(4-Chlorophenoxy)-3,3-dimethyl-1-(1,2,4-triazol-1-yl)-butan-2-one' OR '1-(4-Chlorophenoxy)-3,3-dimethyl-1-(1,2,4-triazol-1-yl)butanone' OR '1-(4-chlorophenoxy)-3,3-dimethyl-1-(1H-1,2,4-triazol-1-yl) butan-2-one' OR '1-(4-Chlorophenoxy)-3,3-dimethyl-1-(1H-1,2,4-triazol-1-yl)-2-butanone' OR '1-(4-chlorophenoxy)-3,3-dimethyl-1-(1H-1,2,4-triazol-1-yl)butan-2-one' OR '1-(4-Chlorophenoxy)-3,3-dimethyl-1-(1H-1,2,4-triazole -1-yl)-2-butanone' OR '1-(4-Chloro-phenoxy)-3,3-dimethyl-1-[1,2,4]triazol-1-yl-butan-2-one' OR 'Acizol' OR 'Adifon' OR 'Amiral' OR 'Azocene' OR 'Bayleton' OR 'Diametom B' OR 'Fenxiunin' OR 'Haleton' OR 'Miltek' OR 'Nurex' OR 'Otria 25' OR 'Rofon' OR 'Tidifon' OR 'triadimefon' OR 'Triadimefone' OR 'Triadimeform' OR 'triadimenol' OR 'Tripinacloraz'):ab,de,kw,lnk,rn,ti,tn OR **'flusilazole'/exp** OR ('F3WG2VVD87' OR '85509-19-9' OR 'Benocarp' OR 'bis (4 fluorophenyl) methyl (1, 2, 4 triazol 1 yl) silane' OR 'bis(4-fluorophenyl)(methyl)(1H-1,2,4-triazol-1-ylmethyl)silane' OR 'bis(4-fluorophenyl)-methyl-(1,2,4-triazol-1-ylmethyl)silane' OR 'Bis(4-fluorophenyl)methyl(1H-1,2,4-triazol-1-ylmethyl)silane' OR 'DPX 6573' OR 'dpx h6573' OR 'DPX-H 6573' OR 'dpxh6573' OR 'DPX-N 6573' OR 'DPX-N6573' OR 'Flusilazol' OR 'Flusilazole' OR 'Fluzilazol' OR 'Nustar' OR 'Olymp' OR 'PPX-H6573'):ab,de,kw,lnk,rn,ti,tn OR **'hexaconazole'/exp** OR ('SX9R3X1FQV' OR '79983-71-4' OR '2 (2, 4 dichlorophenyl) 1 (1h 1, 2, 4 triazol 1 yl) 2 hexanol' OR '2-(2,4-dichlorophenyl)-1-(1,2,4-triazol-1-yl)-2-hexanol' OR '2-(2,4-dichlorophenyl)-1-(1,2,4-triazol-1-yl)hexan-2-ol' OR '2-(2,4-dichlorophenyl)-1-(1H-1,2,4-triazol-1-yl)hexan-2-ol' OR 'alpha butyl alpha (2, 4 dichlorophenyl) 1h 1, 2, 4 triazole 1 ethanol' OR 'Canvil' OR 'Chlortriafol' OR 'Clortriafol' OR 'Contaf' OR 'Flowmax 5SC' OR 'Hexaconazol' OR 'Hexaconazole' OR 'Ranvil'):ab,de,kw,lnk,rn,ti,tn OR **'propiconazole'/exp** OR ('142KW8TBSR' OR '60207-90-1' OR '1 [2 (2, 4 dichlorophenyl) 4 propyl 1, 3 dioxolan 2 ylmethyl] 1h 1, 2, 4 triazole' OR '1-((2-(2,4-Dichlorophenyl)-4-propyl-1,3-dioxolan-2-yl)methyl)-1H-1,2,4-triazole' OR '1-(2-(2,4-Dichlorophenyl)-4-propyl-1,3-dioxolan-2-yl)methyl-1H-1,2,4-triazole' OR '1-(2-(2,4-Dichlorophenyl)-4-propyl-1,3-dioxolan-2-ylmethyl)-1H-1,2,4-triazole' OR '1-[[2-(2,4-dichlorophenyl)-4-propyl-1,3-dioxolan-2-yl]methyl]-1,2,4-triazole' OR '1-[[2-(2,4-Dichlorophenyl)-4-propyl-1,3-dioxolan-2-yl]methyl]-1H-1,2,4-triazole' OR '1-[2-(2,4-Dichloro-phenyl)-4-propyl-[1,3]dioxolan-2-ylmethyl]-1H-[1,2,4]triazole' OR '1-[2-(2,4-dichlorophenyl)-4-propyl-1,3-dioxolan-2-ylmethyl]-1H-1,2,4-triazole' OR '1-{[2-(2,4-dichlorophenyl)-4-propyl-1,3-dioxolan-2-yl]methyl}-1H-1,2,4-triazole' OR 'Bamper' OR 'cga 64250' OR 'cga64250' OR 'Desmel' OR 'Proconazole' OR 'Propiconazol' OR 'Propiconazole' OR 'Propyconazol' OR 'Wocosen' OR 'Wocosin 50TK'):ab,de,kw,lnk,rn,ti,tn OR **'endosulfan'/exp** OR ('OKA6A6ZD4K' OR '115-29-7' OR '5, 6 bis (hydroxymethyl) 1, 2, 3, 4, 7, 7 hexachloronorbornene sulfite' OR '5, 6 bis (hydroxymethyl) 1, 2, 3, 4, 7, 7 hexachloronorbornenesulfite' OR '5, 6 bis (hydroxymethyl) hexachlorobicyclo [2.2.1] hept 2 ene sulfite' OR '5, 6 bis (hydroxymethyl) hexachloronorcamphene sulfite' OR 'alpha endosulfane' OR 'benzoepin' OR 'beosit' OR 'beta endosulfane' OR 'chlorothiepine' OR 'chlorthiapinum' OR 'chlorthiepin' OR 'Chlortiepin' OR 'Crisulfan' OR 'cyclodan' OR 'devisulfan' OR 'Devisulphan' OR 'Endocel' OR 'endogan' OR 'Endosol' OR 'endosulfan' OR 'Endosulphan' OR 'Endotaf' OR 'ensawan' OR 'FMC 5462' OR 'fmc5462' OR 'hexachlorohexahydro 6, 9 methano 2, 4, 3 benzodioxathiepine 3 oxide' OR 'Hexachlorohexahydromethano 2,4,3-benzodioxathiepin-3-oxide' OR 'hexachloronorbornene 5, 6 bis (oxymethylene) sulfite' OR 'Hildan' OR 'Insectophene' OR 'malix' OR 'Rasayansulfan' OR 'Sialan' OR 'thifor' OR 'thimul' OR 'Thiodan' OR 'Thiodon' OR 'thiofor' OR 'thiomul' OR 'Thionate' OR 'thionex' OR 'Thiosulfan' OR 'Thiotox' OR 'thyodan' OR 'thyonex' OR 'tiodan' OR 'tionel' OR 'Tionex' OR 'Tiovel'):ab,de,kw,lnk,rn,ti,tn OR **'pyridaben'/exp** OR ('2E4JBA5272' OR '96489-71-3' OR '2-tert-Butyl-5-(4-tert-Butylbenzylthio)-4-chloropyridazin-3(2H)-one' OR '2-tert-Butyl-5-(4-tert-butyl-benzylthio)-4-chloropyridazin-3(2H)-one' OR '2-tert-butyl-5-[(4-tert-butylbenzyl)thio]-4-chloropyridazin-3(2H)-one' OR '2-tert-butyl-5-[(4-tert-butylbenzyl)thio]-4-chloro-pyridazin-3-one' OR 'Damanlin' OR 'Nexter' OR 'Pyramite' OR 'Pyridaben' OR 'Sanmite'):ab,de,kw,lnk,rn,ti,tn OR **'methanol'/exp** OR ('67-56-1' OR 'Y4S76JWI15' OR 'carbinol' OR 'Carbonal' OR 'hydroxymethan' OR 'Hydroxymethane' OR 'MeOH' OR 'methanol' OR 'Methoxy Group' OR 'methyl alcohol' OR 'Methyl hydrate' OR 'Methyl hydroxide' OR 'Methylalcohol' OR 'Methylic alcohol' OR 'Methylol' OR 'MetOH' OR 'Monohydroxymethane' OR 'monomethylol' OR 'wood alcohol' OR 'Wood naphtha' OR 'wood spirit'):ab,de,kw,lnk,rn,ti,tn OR **'cyproconazole'/exp** OR ('94361-06-5' OR '622B9C3E6T' OR 'Atemi' OR 'Cyproconazol' OR 'Cyproconazole'):ab,de,kw,lnk,rn,ti,tn OR **'triticonazole'/exp** OR ('131983-72-7' OR '(1RS)-(E)-5-((4-chlorophenyl)methylene)-2,2-dimethyl-1-(1H-1,2,4-triazol-1-ylmethyl)cyclopentan-1-ol' OR 'Triticonazole'):ab,de,kw,lnk,rn,ti,tn OR **'diniconazole'/exp** OR ('X82HVO1N83' OR '76714-16-4' OR '83657-24-3' OR '1-(2,4-dichlorophenyl)-4,4-dimethyl-2-(1,2,4-triazol-1-yl)-1-penten-3-ol' OR '1-(2,4-Dichlorophenyl)-4,4-dimethyl-2-(1H-1,2,4-triazol-1-yl)pent-1-en-3-ol' OR 'Diclopentezol' OR 'Diniconazole' OR 'S 3308' OR 'S-3308L'):ab,de,kw,lnk,rn,ti,tn OR **'zidovudine'/exp** OR ('4B9XT59T7S' OR '30516-87-1' OR '3 azido 2, 3 dideoxyribosylthymine' OR '3 azido 2, 3 dideoxythymidine' OR '3 Azido 2,3 Dideoxythymidine' OR '3 Azido 3 deoxythymidine' OR '3 azidothymidine' OR '3-azido3-deoxythymidine' OR '3-Azido-3-deoxythymidine' OR 'adovi' OR 'avirzid' OR 'azidodeoxythymidine' OR 'azidomine' OR 'Azidothymidine' OR 'Azitidin' OR 'AZT' OR 'bio zt' OR 'bw a 509 u' OR 'BW A509U' OR 'BW-A 509U' OR 'BWA509U' OR 'BWA-509U' OR 'Dendrigen' OR 'pranadox' OR 'retrocar' OR 'Retrovir' OR 'Retrovis' OR 'ZDV' OR 'zidis' OR 'zidovir' OR 'zidovudin' OR 'zidovudine' OR 'Zidovudinum' OR 'zudovidine' OR 'zydowin'):ab,de,kw,lnk,rn,ti,tn OR **'metoclopramide'/exp** OR ('L4YEB44I46' OR '364-62-5' OR '4 amino 5 chloro n (2 diethylaminoethyl) 2 methoxybenzamide' OR '4 amino 5 chloro n (2 diethylaminoethyl) o anisamide' OR '4 amino 5 chloro n (2 diethylaminoethyl) ortho anisamide' OR '4 Amino-5-chloro-N-(2-(diethylamino)ethyl)-2-methoxybenzamide' OR '4-Amino-5-chloro-N-(2-(diethylamino)ethyl)-o-anisamide' OR '4-Amino-5-chloro-N-(2-diethylamino-ethyl)-2-methoxy-benzamide' OR '4-amino-5-chloro-N-[2-(diethylamino)ethyl]-2-methoxybenzamide' OR '4-amino-5-chloro-N-[2-(diethylamino)ethyl]-2-methoxy-benzamide' OR '4-amino-5-chloro-N-[2-(diethylamino)-ethyl]-2-methoxybenzamide' OR '5 chloro 2 methoxyprocainamide' OR 'ahr 3070 c' OR 'ahr 3070c' OR 'ahr3070c' OR 'ametic' OR 'anausin' OR 'apo-metoclop' OR 'aputern' OR 'betaclopramide' OR 'carnotprim primperan' OR 'Cerucal' OR 'clodilion' OR 'clopamon' OR 'clopan' OR 'Clopra' OR 'clopram' OR 'Clopromate' OR 'Degan' OR 'del 1267' OR 'del1267' OR 'dibertil' OR 'Duraclamid' OR 'Elieten' OR 'emenil' OR 'emetal' OR 'emetard' OR 'Emetid' OR 'Emitasol' OR 'emperal' OR 'encil' OR 'enzimar' OR 'Eucil' OR 'gastro timelets' OR 'gastrobi' OR 'Gastrobid' OR 'Gastromax' OR 'Gastronerton' OR 'gastrosil' OR 'Gastrotablinen' OR 'gastrotem' OR 'gastrotimelets' OR 'gavistal' OR 'gensil' OR 'Gimoli' OR 'hemesis' OR 'hyrin' OR 'imperan' OR 'm 813' OR 'm813' OR 'maril' OR 'Maxeran' OR 'maxeron' OR 'maxolan' OR 'Maxolon' OR 'mcp-beta tropfen' OR 'meclomid' OR 'meclopamide' OR 'meclopramide' OR 'Meclopran' OR 'Megaldrate' OR 'meramide' OR 'Metaclopramide' OR 'Metadrate' OR 'metagliz' OR 'metamide' OR 'Methochlopramide' OR 'methoclopramide' OR 'methoclopramine' OR 'Methoxychlorprocainamide' OR 'metlazel' OR 'Metochlopramide' OR 'Metochloropramide' OR 'Metoclol' OR 'metoclopamide' OR 'metoclopramid' OR 'metoclopramide' OR 'Metoclopramidum' OR 'metoclopramine' OR 'metoclopranide hydrochloride' OR 'metoclor' OR 'metoclorpramide' OR 'metocobil' OR 'metocyl' OR 'metodopramide' OR 'metolon' OR 'metopram' OR 'metox' OR 'metozolv' OR 'metpamid' OR 'metram' OR 'Metramid' OR 'Moriperan' OR 'mygdalon' OR 'nausil' OR 'neopramiel' OR 'netaf' OR 'nilatika' OR 'normastin' OR 'Octamide' OR 'opram' OR 'Parmid' OR 'Paspertin' OR 'Peraprin' OR 'perinorm' OR 'pharmyork' OR 'Plasil' OR 'pramidin' OR 'Pramiel' OR 'pramin' OR 'pramotel' OR 'Primperan' OR 'primperil' OR 'prinparl' OR 'prokinyl lp' OR 'prowel' OR 'pulin' OR 'Pylomid' OR 'Reclomide' OR 'Regla' OR 'Reglan' OR 'Reliveran' OR 'rimetin' OR 'sensamide' OR 'sotatic-10' OR 'Terperan' OR 'tomid' OR 'vertivom' OR 'vomitrol' OR 'zumatrol'):ab,de,kw,lnk,rn,ti,tn OR **'acebutolol'/exp** OR ('67P356D8GH' OR '37517-30-9' OR '1 (2 acetyl 4 butyramidophenoxy) 2 hydroxy 3 isopropylaminopropane' OR '1-(2-Acetyl-4-n-butyramidophenoxy)-2-hydroxy-3-isopropylaminopropane' OR 'Acebrutololum' OR 'acebutolol' OR 'Acebutololo' OR 'Acebutololum' OR 'acecor' OR 'ApoAcebutolol' OR 'bay c 7705' OR 'bay c7705' OR 'diasectral' OR 'espesil' OR 'flebutol' OR 'grifobutol' OR 'il 17803a' OR 'il17803a' OR 'M & B 17803A' OR 'm and b 17803 a' OR 'M and B 17803A' OR 'M and B17803 A' OR 'M&B-17803 A' OR 'Monitan' OR 'n [3 acetyl 4 [2 hydroxy 3 [ (methylethyl) amino] propoxy] phenyl] butanamide' OR 'N-[3-acetyl-4-[2-hydroxy-3-[(1-methylethyl)amino]propoxy]phenyl]butanamide' OR 'Neptal' OR 'neptall' OR 'NovoAcebutolol' OR 'Prent' OR 'Rhotral' OR 'Sectral' OR 'Wesfalin'):ab,de,kw,lnk,rn,ti,tn

OR

**Part 2**

**'isoniazid'/exp** OR ('V83O1VOZ8L' OR '54-85-3' OR '4 pyridinecarbohydrazide' OR '4 pyridinecarboxylic acid hydrazide' OR 'Abdizide' OR 'Andrazide' OR 'Anidrasona' OR 'antimic' OR 'Antimicina' OR 'antimicine' OR 'Antituberkulosum' OR 'apacizina (aminosalicylate)' OR 'Armacide' OR 'Armazid' OR 'Armazide' OR 'atcotibin' OR 'Atcotibine' OR 'Azuren' OR 'bacillen' OR 'Bacillin' OR 'Cedin' OR 'Cemidon' OR 'Chemiazid' OR 'Chemidon' OR 'Continazine' OR 'Cortinazine' OR 'Cotinazin' OR 'cotinazine' OR 'Cotinizin' OR 'cotinizine' OR 'curazid forte' OR 'Defonin' OR 'dianicotyl' OR 'diazid' OR 'Dibutin' OR 'Diforin' OR 'Dinacrin' OR 'Ditubin' OR 'Ebidene' OR 'Eralon' OR 'eralone' OR 'Ertuban' OR 'Eutizon' OR 'eutizone' OR 'Evalon' OR 'Fetefu' OR 'Fimalene' OR 'fimaline' OR 'FRS-3' OR 'fsr3' OR 'Ftivazide' OR 'GINK' OR 'hain' OR 'hiconyl' OR 'Hid rasonil' OR 'hidraciber' OR 'Hidranizil' OR 'Hidrasonil' OR 'hidrazida' OR 'Hidrulta' OR 'hidrulte' OR 'Hidrun' OR 'Hycozid' OR 'hycozide' OR 'hydrasonil' OR 'Hydrazid' OR 'Hydrazide' OR 'hydrazin' OR 'Hyozid' OR 'Hyzyd' OR 'ido tebin' OR 'Idrazil' OR 'Inah' OR 'inh burgthal' OR 'INHd20' OR 'Inizid' OR 'inizide' OR 'Iscotin' OR 'iscotine' OR 'Isidrina' OR 'isidrine' OR 'Ismazide' OR 'Isobicina' OR 'isobicine' OR 'Isocid' OR 'Isocidene' OR 'isoco tin' OR 'Isocotin' OR 'isocotine' OR 'Isohydrazide' OR 'Isokin' OR 'Isolyn' OR 'isolyne' OR 'isomazide' OR 'isomerina' OR 'Isonerit' OR 'Isonex' OR 'isoniac' OR 'Isoniacid' OR 'isoniazid' OR 'Isoniazide' OR 'isoniazidine' OR 'Isoniazidum' OR 'isoniazone' OR 'isonicazid' OR 'Isonicazide' OR 'Isonicid' OR 'isonicide' OR 'Isonico' OR 'Isonicotan' OR 'isonicotane' OR 'isonicotic acid hydrazide' OR 'Isonicotil' OR 'isonicotinate hydrazide' OR 'Isonicotinhydrazid' OR 'isonicotinhydrazide' OR 'Isonicotinic acid hydrazide' OR 'isonicotinic acid hydrazine' OR 'isonicotinic acid hydrazone' OR 'Isonicotinic Acid Vanillylidenehydrazide' OR 'Isonicotinic hydrazide' OR 'isonicotinicacid hydrazide' OR 'Isonicotinohydrazide' OR 'Isonicotinoyl hydrazide' OR 'isonicotinoylhydrazide' OR 'Isonicotinoylhydrazine' OR 'Isonicotinyl hydrazide' OR 'Isonicotinyl hydrazine' OR 'Isonicotinylhydrazide' OR 'Isonicotinylhydrazine' OR 'Isonide' OR 'Isonidrin' OR 'isonidrine' OR 'Isonikazid' OR 'isonikazide' OR 'Isonilex' OR 'isonilyd' OR 'Isonin' OR 'Isonindon' OR 'isonindone' OR 'isonine' OR 'Isonirit' OR 'isonisin' OR 'isonisine' OR 'Isoniton' OR 'isonitone' OR 'isonivit' OR 'Isonizida' OR 'Isonizide' OR 'isopharmide' OR 'Isotamine' OR 'Isotebe' OR 'Isotebezid' OR 'isotebezide' OR 'isothiavit' OR 'Isotinyl' OR 'Isozid' OR 'Isozide' OR 'isozin' OR 'isozine' OR 'isozone' OR 'Isozyd' OR 'isozyde' OR 'izoniazid' OR 'L 1945' OR 'Laniazid' OR 'Laniozid' OR 'LANIZID' OR 'Mayambutol' OR 'micosan' OR 'micosane' OR 'milazide' OR 'Mybasan' OR 'mybasane' OR 'Neoteben' OR 'neotebene' OR 'Neo-Tizide' OR 'Neoxin' OR 'neoxine' OR 'neoxon' OR 'neoxone' OR 'Neumandin' OR 'Nevin' OR 'Niadrin' OR 'nicatibine' OR 'nicazid' OR 'Nicazide' OR 'Nicetal' OR 'Nicizina' OR 'nicodrin' OR 'Niconyl' OR 'nicosciorin' OR 'nicotibin' OR 'Nicotibina' OR 'Nicotibine' OR 'Nicotisan' OR 'nicotubin' OR 'nicotubine' OR 'nicozid' OR 'Nicozide' OR 'nicozyd' OR 'Nidaton' OR 'Nidrazid' OR 'nidrazide' OR 'Nikozid' OR 'nikozide' OR 'niosciorine' OR 'Niplen' OR 'Nitadon' OR 'Niteban' OR 'Nitebannsc 9659' OR 'nortibina' OR 'nortibine' OR 'Nydrazid' OR 'nydrazide' OR 'Nyscozid' OR 'nyscozide' OR 'Pelazid' OR 'pelazide' OR 'Percin' OR 'Phthisen' OR 'Phthivazid' OR 'Phthivazide' OR 'puran' OR 'Pycazide' OR 'Pyreazid' OR 'pyreazide' OR 'Pyricidin' OR 'pyricidine' OR 'Pyridicin' OR 'pyridine 4 carbohydrazide' OR 'pyridine 4 carbonic acid hydrazide' OR 'pyridine 4 carboxyhydrazide' OR 'pyridine-4-carboxylic acid hydrazide' OR 'Pyrizidin' OR 'pyrizidine' OR 'ramnanon' OR 'Raumanon' OR 'Razide' OR 'Retozide' OR 'rhymicid' OR 'Rifater' OR 'Rimicid' OR 'rimicide' OR 'Rimifon' OR 'Rimiphone' OR 'Rimitsid' OR 'Robiselin' OR 'Robisellin' OR 'robiselline' OR 'Roxifen' OR 'roxyfen miquel' OR 'RP 5015' OR 'Sanohidrazina' OR 'sanohydrazina' OR 'sanohydrazine' OR 'santerazid' OR 'santerazide' OR 'Sauterazid' OR 'Sauterzid' OR 'Stanozide' OR 'supercidin' OR 'tb phlogin' OR 'Tebecid' OR 'tebecide' OR 'tebecin' OR 'tebecine' OR 'tebemid' OR 'Tebenic' OR 'tebesium' OR 'tebetracin' OR 'tebetracine' OR 'Tebexin' OR 'tebexine' OR 'Tebilon' OR 'tebilone' OR 'Tebos' OR 'Teebaconin' OR 'teebaconine' OR 'Tekazin' OR 'tekazine' OR 'thiocevit' OR 'Tibazide' OR 'Tibemid' OR 'tibemide' OR 'Tibiazide' OR 'Tibinide' OR 'Tibison' OR 'tibisone' OR 'tibitan' OR 'tibitane' OR 'Tibivis' OR 'Tibizide' OR 'Tibusan' OR 'tibusane' OR 'Tisin' OR 'Tisiodrazida' OR 'tisiodrazide' OR 'tisiotrazida' OR 'Tizide' OR 'Tubazid' OR 'Tubazide' OR 'Tubeco' OR 'Tubecotubercid' OR 'tubercid' OR 'Tuberian' OR 'tuberiane' OR 'Tubicon' OR 'tubicone' OR 'Tubilysin' OR 'Tubizid' OR 'Tubomel' OR 'tubonil' OR 'tubylisin' OR 'tubylisine' OR 'tyrid' OR 'Tyvid' OR 'tyvide' OR 'Unicocyde' OR 'Unicozyde' OR 'valifol' OR 'Vazadrine' OR 'Vederon' OR 'vederone' OR 'vitazide' OR 'yuhan-zid' OR 'Zidafimia' OR 'Zinadon' OR 'zinadone' OR 'Zonazide'):ab,de,kw,lnk,rn,ti,tn OR **'saccharin'/exp** OR ('FST467XS7D' OR '81-07-2' OR '128-44-9' OR '1, 2 benzisothiazol 3 (2h) one 1, 1 dioxide' OR '1, 2 benzisothiazol 3 one 1, 1 dioxide' OR '1, 2 dihydro 2 ketobenzisosulfonazole' OR '1, 2-Benzisothiazol-3(2H)-one, 1,1-dioxide' OR '1,2-Benzisothiazol-3(2H)-one 1,1-dioxide' OR '1,2-Benzisothiazol-3(2H)-one, 1,1-dioxide' OR '1,2-Dihydro-2-ketobenzisosulfonazole' OR '1,2-Dihydro-2-ketobenzisosulphonazole' OR '2, 3 dihydro 3 oxobenzisosulfonazole' OR '2,3-Dihydro-3-oxobenzisosulfonazole' OR '2,3-Dihydro-3-oxo-Benzisosulfonazole' OR '2,3-Dihydro-3-oxobenzisosulphonazole' OR '2-Sulfobenzoic acid imide' OR '2-Sulfobenzoic imide' OR '2-Sulfobenzoicimide' OR '2-Sulphobenzoic imide' OR 'Anhydro-o-sulfaminebenzoic acid' OR 'Benzo-2-sulfiide' OR 'Benzo-2-sulphimide' OR 'Benzoic acid sulfimide' OR 'Benzoic sulfimide' OR 'Benzoic sulphimide' OR 'Benzosulfimide' OR 'Benzosulfinide' OR 'Benzosulphimide' OR 'Benzo-sulphinide' OR 'Benzoylsulfonic Imide' OR 'Cristallose' OR 'Crystallose' OR 'Garantose' OR 'Glucid' OR 'glucide' OR 'Gluside' OR 'Glycophenol' OR 'Hermesetas' OR 'Kandiset' OR 'Kristallose' OR 'Madhurin' OR 'Natreen' OR 'Neosaccharin' OR 'o-Benzoic acid sulfimide' OR 'o-Benzoic sulfimide' OR 'o-Benzoic sulphimide' OR 'o-Benzosulfimide' OR 'o-Benzosulphimide' OR 'o-Benzoyl sulfimide' OR 'o-Benzoyl sulphimide' OR 'O-Benzoylsulfimide' OR 'ortho sulfobenzimide' OR 'ortho sulfobenzoic acid imide' OR 'Ortho sulphobenzamide' OR 'o-Sulfobenzimide' OR 'o-Sulfobenzoic acid imide' OR 'O-Sulfobenzoic imide' OR 'o-Sulfonbenzoic acid imide sodium salt' OR 'Sacarina' OR 'Saccharimide' OR 'saccharin' OR 'Saccharina' OR 'Saccharine' OR 'Saccharinol' OR 'Saccharinose' OR 'saccharoid' OR 'Saccharol' OR 'Saxin' OR 'Sodium o-benzosulfimide' OR 'Sodium saccharide' OR 'Sucrette' OR 'Sucromat' OR 'sweet n low' OR 'Sweeta' OR 'sweetex' OR 'sweetnin' OR 'Sykose' OR 'Syncal' OR 'Willosetten' OR 'Zaharina'):ab,de,kw,lnk,rn,ti,tn OR **'penicillin G sodium'/exp** OR ('YS5LY7JF4N' OR '69-57-8' OR '1406-05-9' OR '6 (phenylacetamido) penicillanate sodium' OR '6 phenylacetamidopenicillanate sodium' OR 'American penicillin' OR 'Benpen' OR 'Benzylpenicillin sodium' OR 'benzylpenicillinate sodium' OR 'Benzylpenicillinic acid sodium salt' OR 'bupenna sodium' OR 'Coliriocilina' OR 'Crystapen' OR 'Kesso-Pen' OR 'Mycofarm' OR 'Novocillin' OR 'Parcillin' OR 'Pekamin' OR 'pen a brasive' OR 'Pengesod' OR 'Penibiot' OR 'Penicilina G Llorente' OR 'Penicillin G' OR 'Penicillin Grünenthal' OR 'penicillin ii sodium' OR 'penicillin sodium' OR 'penicilline g sodium' OR 'PenicillinGsodiumsalt' OR 'Penilaryn' OR 'Penilevel' OR 'Peniroger' OR 'Pfizerpen' OR 'Sodiopen' OR 'Sodipen' OR 'Sodium 6-(phenylacetamido)penicillanate' OR 'sodium benzyl penicillin' OR 'Sodium benzylpenicillin' OR 'Sodium benzylpenicillinate' OR 'Sodium penicillin' OR 'sodium penicilline g' OR 'Sugracillin sodium salt' OR 'Unicilina' OR 'Ursopen' OR 'Veticillin'):ab,de,kw,lnk,rn,ti,tn OR **'thalidomide'/exp** OR ('4Z8R6ORS6L' OR '50-35-1' OR '.alpha.-(N-Phthalimido)glutarimide' OR '.alpha.-N-Phthalylglutaramide' OR '.alpha.-Phthalimidoglutarimide' OR '3-Phthalimidoglutarimide' OR 'Algosediv' OR 'alpha (n phthalimido) glutarimide' OR 'alpha-(N-Phthalimido)glutarimide' OR 'alpha-N-Phthalylglutaramide' OR 'alpha-Phthalimidoglutarimide' OR 'Asidon 3' OR 'Asmadion' OR 'Asmaval' OR 'Bonbrain' OR 'Calmore' OR 'Calmorex' OR 'Celgene' OR 'Contergan' OR 'Corronarobetin' OR 'Distaval' OR 'Distaxal' OR 'Distoval' OR 'Ectiluran' OR 'Enterosediv' OR 'Gastrinide' OR 'Glupan' OR 'Glutanon' OR 'Grippex' OR 'Hippuzon' OR 'Imidene' OR 'Isomin' OR 'Kedavon' OR 'Kevadon' OR 'n (2, 6 dioxopiperid 3 yl) phthalimide' OR 'N-(2,6-dioxo-3-piperidinyl)phthalimide' OR 'N-(2,6-Dioxo-3-piperidyl)phthalimide' OR 'n-(2,6-dioxopiperidin-3-yl)phthalimide' OR 'Neaufatin' OR 'Neosedyn' OR 'Neosydyn' OR 'Nerosedyn' OR 'Neufatin' OR 'Neurodyn' OR 'Neurosedin' OR 'Neurosedym' OR 'Neurosedyn' OR 'neurosedyne' OR 'Nevrodyn' OR 'Nibrol' OR 'Noctosediv' OR 'Noxodyn' OR 'N-Phthalimidoglutamic acid imide' OR 'N-Phthaloylglutamimide' OR 'N-Phthalylglutamic acid imide' OR 'nsc 66847' OR 'NSC66847' OR 'Pangul' OR 'Pantosediv' OR 'Pharmion' OR 'Polygripan' OR 'Pro-Bam M' OR 'Pro-ban M' OR 'Profarmil' OR 'Quetimid' OR 'Quietoplex' OR 'Sandormin' OR 'Sedalis' OR 'Sedimide' OR 'Sedin' OR 'Sedisperil' OR 'Sedoval' OR 'shin naito' OR 'Shinnibrol' OR 'Sleepan' OR 'Slipro' OR 'Softenil' OR 'Softenon' OR 'Synovir' OR 'Talargan' OR 'Talidomide' OR 'Talimol' OR 'Talinol' OR 'Talismol' OR 'Talizer' OR 'Telagan' OR 'Telargan' OR 'Telargean' OR 'Tensival' OR 'thado' OR 'Thaled' OR 'thalidomid' OR 'thalidomide' OR 'Thalidomidum' OR 'Thalin' OR 'Thalinette' OR 'thalix' OR 'Thalomid' OR 'Thalomide' OR 'Theophilcholine' OR 'Valgis' OR 'Valgraine' OR 'Yodomin'):ab,de,kw,lnk,rn,ti,tn OR **'doxylamine'/exp** OR ('95QB77JKPL' OR '469-21-6' OR '562-10-7' OR '.alpha.-Dimethylaminoethoxyphenylmethyl-2-picoline' OR '2-Dimethylaminoethoxyphenylmethyl-2-picoline' OR 'alsadorm' OR 'decapryn' OR 'deoxylamine succinate' OR 'Diclectin' OR 'Dolased' OR 'donormyl' OR 'dormidina' OR 'Dossilamina' OR 'doxilamina' OR 'doxylamine' OR 'doxylaminesuccinate' OR 'doxylaminium succinate' OR 'doxylaminosuccinate' OR 'Doxylaminum' OR 'doxy-sleep-aid' OR 'Dozile' OR 'Evanorm' OR 'gittalun' OR 'histadoxylamine' OR 'hoggar' OR 'mereprine' OR 'Mersyndol' OR 'Restavit' OR 'sedaplus' OR 'Somnil' OR 'Syndol' OR 'Unisom' OR 'vicks nyquil'):ab,de,kw,lnk,rn,ti,tn OR **'diphenhydramine'/exp** OR ('8GTS82S83M' OR '58-73-1' OR '147-24-0' OR '.beta.-(Dimethylamino)ethyl benzhydryl ether' OR '.beta.-Dimethylamino-aethyl-benzhydryl-aether' OR '2 (benzhydroloxy) n, n dimethylethylamine' OR '2 (diphenylmethoxy) n, n dimethyl ethylamine' OR '2 (diphenylmethoxy) n, n dimethylethylamine' OR '2 benzhydryloxy n, n dimethylethylamine' OR '2 diphenylmethoxy n, n dimethylethylamine' OR '2-(Benzhydryloxy)-N,N-dimethylethanamine' OR '2-(Benzhydryloxy)-N,N-dimethylethylamine' OR '2-(Benzohydryloxy)-N,N-dimethylethylamine' OR '2-(diphenylmethoxy)-N,N-dimethylethanamine' OR '2-(Diphenylmethoxy)-N,N-dimethylethylamine' OR '2-(diphenylmethyl)oxy-N,N-dimethylethanamine' OR '2-(diphenylmethyl)oxy-N,N-dimethyl-ethanamine' OR '2-[(diphenylmethyl)oxy]-N,N-dimethylethanamine' OR '2-[di(phenyl)methoxy]-N,N-dimethylethanamine' OR '2-benzhydryloxy-N,N-dimethylethanamine' OR '2-benzhydryloxy-N,N-dimethyl-ethanamine' OR '2-diphenylmethoxy-N,N-demthylethanamine' OR '2-Diphenylmethoxy-N,N-dimethylethylamine' OR 'Aleryl' OR 'Alledryl' OR 'Allerdryl' OR 'Allergan' OR 'Allergeval' OR 'Allergical' OR 'Allergina' OR 'Allergival' OR 'Amidryl' OR 'Antistominum' OR 'Antomin' OR 'Automin' OR 'Bagodryl' OR 'banaril' OR 'Banophen' OR 'Baramine' OR 'Beldin' OR 'Belix' OR 'Benachlor' OR 'benadril' OR 'Benadrin' OR 'Benadryl' OR 'benadyl' OR 'Ben-allergin' OR 'Benapon' OR 'Benhydramin' OR 'benocten' OR 'Benodin' OR 'Benodine' OR 'Benylan' OR 'Benylin' OR 'Benzantine' OR 'Benzhydramine' OR 'Benzhydraminum' OR 'Benzhydroamina' OR 'beta dimethylaminoethyl benzhydryl ether' OR 'beta-Dimethylaminoethanol diphenylmethyl ether' OR 'beta-Dimethylaminoethylbenzhydrylether' OR 'Betramin' OR 'caladryl' OR 'carphenamine' OR 'carphenex' OR 'cathejell' OR 'Compoz' OR 'Dabylen' OR 'Debendrin' OR 'Dermistina' OR 'Dermodrin' OR 'Desentol' OR 'Diabenyl' OR 'Diabylen' OR 'dibadorm n' OR 'Dibendrin' OR 'Dibenil' OR 'Dibondrin' OR 'dibrondrin' OR 'Difedryl' OR 'Difenhydramin' OR 'Difenhydramine' OR 'Dihidral' OR 'Dimedrol' OR 'Dimedryl' OR 'Dimehydrinate' OR 'Dimethylamine benzhydryl ester' OR 'dimidril' OR 'dimiril' OR 'Diphantine' OR 'diphedryl' OR 'Diphen' OR 'diphenacen' OR 'diphendramine' OR 'Diphenhist' OR 'diphenhydramide' OR 'diphenhydramin' OR 'diphenhydramine' OR 'Diphenhydraminum' OR 'diphenydramine' OR 'Diphenylhydramin' OR 'Diphenylhydramine' OR 'Dobacen' OR 'Dormarex 2' OR 'Dormin' OR 'dryhistan' OR 'Dryistan' OR 'Drylistan' OR 'Dylamon' OR 'dytan' OR 'emesan' OR 'Etanautine' OR 'Genahist' OR 'Histacyl' OR 'Histaxin' OR 'histergan' OR 'Hyadrine' OR 'Hydramine' OR 'Hyrexin' OR 'Ibiodral' OR 'Medidryl' OR 'Mephadryl' OR 'n, n dimethyl 2 (diphenylmethoxy) ethylamine' OR 'N,N-Dimethyl-2-(diphenylmethoxy)-ethylamine hydrochloride' OR 'N,N-Dimethyl-2-diphenylmethyloxyethylamine' OR 'Nausen' OR 'neosynodorm' OR 'Novamina' OR 'nytol' OR 'o benzhydryldimethylaminoethanol' OR 'O-Benzhydryl(dimethylamino)ethanol' OR 'Probedryl' OR 'reisegold' OR 'resmin' OR 'restamin' OR 'Rigidil' OR 'Rigidyl' OR 'sediat' OR 'sedryl' OR 'Siladryl' OR 'Silphen' OR 'sleepeze' OR 'Sleep-Eze D' OR 'sominex' OR 'Syntedril' OR 'Syntodril' OR 'trux-adryl' OR 'tzoali' OR 'unisom sleepgels' OR 'valdrene' OR 'venasmin' OR 'vertirosan' OR 'vicks formula 44' OR 'vilbin' OR 'wehdryl' OR 'ziradryl'):ab,de,kw,lnk,rn,ti,tn OR **'clopyralid'/exp** OR ('10G14M0WDH' OR '1702-17-6' OR '3,6-Dichloropicolinic acid' OR 'Benzalox' OR 'Cirtoxin' OR 'Cliophar' OR 'Clopiralid' OR 'Clopyralid' OR 'Clopyralide' OR 'Cyronal' OR 'dichloropyridine acid' OR 'Dowco 290' OR 'Huiloralid' OR 'Loncid' OR 'Lontrel' OR 'Matrigon' OR 'Transline' OR 'Versatill'):ab,de,kw,lnk,rn,ti,tn OR **'camphor'/exp** OR ('76-22-2' OR '21368-68-3' OR '1, 7, 7 trimethylbicyclo [2.2.1] heptan 2 one' OR '1,7,7-Trimethyl-bicyclo(2,2,1)Heptan-2-one' OR '1,7,7-Trimethylbicyclo(2.2.1)-2-heptanone' OR '1,7,7-Trimethylbicyclo(2.2.1)heptan-2-one' OR '1,7,7-Trimethylbicyclo[2.2.1]-2-heptanone' OR '1,7,7-Trimethylbicyclo[2.2.1]heptan-2-one' OR '1,7,7-Trimethyl-bicyclo[2.2.1]heptan-2-one' OR '1,7,7-trimethyl-bicyclo[2.2.1]heptane-2-one' OR '2 bornanone' OR '2 camphanone' OR '2 keto 1, 7, 7 trimethylnorcamphane' OR '2 oxo 1, 7, 7 trimethylbicyclo (2.2.1) heptane' OR '2 oxobornane' OR '2-Camphanone' OR '2-Camphonone' OR '2-Keto-1,7,7-trimethylnorcamphane' OR 'Alcanfor' OR 'Alphanon' OR 'Bornan-2-one' OR 'camphor' OR 'camphora' OR 'd-2-Bornanone' OR 'd-2-Camphanone' OR 'korodin' OR 'pi hydroxycamphor' OR 'Root bark oil' OR 'Root bark spirit' OR 'sarna'):ab,de,kw,lnk,rn,ti,tn OR **'fipronil'/exp** OR ('QGH063955F' OR '120068-37-3' OR '5 amino 1 (2, 6 dichloro alpha, alpha, alpha trifluoro para tolyl) 4 trifluoromethylsulfinylpyrazole 3 carbonitrile' OR '5 amino 1 [2, 6 dichloro 4 (trifluoromethyl) phenyl] 4 [ (trifluoromethyl) sulfinyl] 1h pyrazole 3 carbonitrile' OR '5-amino-1 -(2,6-dichloro-4-trifluoromethylphenyl)-4-trifluoromethylsulfinyl-1 H-pyrazole-3-carbonitrile' OR '5-amino-1-(2,6-dichloro-4-(trifluoromethyl) phenyl)-4-((trifluoromethyl) sulfinyl)-1H-pyrazol-3-carbonitrile' OR '5-amino-1-(2,6-dichloro-4-(trifluoromethyl)phenyl)-4-((trifluoromethyl)sulfinyl)-1h-pyrazole-3-carbonitrile' OR '5-amino-1-(2,6-dichloro-alpha,alpha,alpha-trifluoro-p-tolyl)-4-trifluoromethylsulfinylpyrazole-3-carbonitile' OR '5-Amino-1-[2,6-dichloro-4-(trifluoromethyl)phenyl]-4-(trifluoromethyl)sulfinylpyrazole-3-carbonitrile' OR '5-amino-1-[2,6-dichloro-4-(trifluoromethyl)phenyl]-4-(trifluoromethylsulfinyl)pyrazole-3-carbonitrile' OR '5-amino-1-[2,6-dichloro-4-(trifluoromethyl)phenyl]-4-[(trifluoromethyl)sulfinyl]-1H-pyrazole-3-carbonitrile' OR '5-amino-1-[2,6-dichloro-4-(trifluoromethyl)phenyl]-4-trifluoromethanesulfinyl-1H-pyrazole-3-carbonitrile' OR 'fiprex' OR 'fipronil' OR 'Fluocyanobenpyrazole' OR 'Frontline Spot-on' OR 'Frontline Spray' OR 'Frontline Top Spot' OR 'Goliath gel' OR 'Granedo MC' OR 'Maxforce FC' OR 'Termidor'):ab,de,kw,lnk,rn,ti,tn OR **'glycerol'/exp** OR ('PDC6A3C0OX' OR '56-81-5' OR '107283-02-3' OR '144086-02-2' OR '144086-03-3' OR '25618-55-7' OR '8013-25-0' OR '1, 2, 3 propanetriol' OR '1, 2, 3 trihydroxypropane' OR '1,2,3-Propanetriol' OR '1,2,3-triglycerol' OR '1,2,3-TRIHYDROXYPROPAN-2-YL' OR '1,2,3-trihydroxypropane' OR '1,2,3-trihydroxypropanol' OR '1,3-Propanetriol' OR '1,3-Trihydroxypropane' OR 'alditol' OR 'Artificial tears' OR 'Bulbold' OR 'Citifluor AF 2' OR 'Cristal' OR 'Dagralax' OR 'Glicerina' OR 'Glyceol' OR 'glycerin' OR 'Glycerine' OR 'Glycerinum' OR 'Glyceritol' OR 'glycerol' OR 'Glycerolum' OR 'Glycyl alcohol' OR 'Glyrol' OR 'Glysanin' OR 'Glyzerin' OR 'Grocolene' OR 'microglycerin' OR 'Monoctanoin Component D' OR 'Neutracett' OR 'Oelsuess' OR 'Olsuss' OR 'Ophthalgan' OR 'Osmoglyn' OR 'Polyglycerin' OR 'Polyglycerine' OR 'Polyglycerol' OR 'Pricerine 9091' OR 'PROPANE-1,2,3-TRIOL' OR 'Propanetriol' OR 'Tegin M' OR 'Trihydroxypropane' OR 'Tryhydroxypropane' OR 'vilardell' OR 'Vitrosupos'):ab,de,kw,lnk,rn,ti,tn OR **'hexazinone'/exp** OR ('51235-04-2' OR 'Y51727MR1Y' OR '3 cyclohexyl 6 (dimethylamino) 1 methyl 1, 3, 5 triazine 2, 4 (1h, 3h) dione' OR '3-Cyclohexy-6-(dimethylamino)-1-methyl-1,3,5-triazine-2,4(1H,3H)-dione' OR '3-Cyclohexyl-6-(dimethylamino)-1-methyl-1,3,5-triazine-2,4(1H,3H)-dione' OR '3-cyclohexyl-6-(dimethylamino)-1-methyl-1,3,5-triazine-2,4-dione' OR 'Gridball' OR 'Hexazinoe' OR 'Hexazinon' OR 'Hexazinone' OR 'Velpar'):ab,de,kw,lnk,rn,ti,tn OR **'imazamox'/exp** OR ('UG6793ON5F' OR '114311-32-9' OR 'Imazamox'):ab,de,kw,lnk,rn,ti,tn OR **'imazapyr'/exp** OR ('81334-34-1' OR '2-(4-Isopropyl-4-methyl-5-oxo-2-imidazolin-2-yl)nicotinic acid' OR 'Imazapyr'):ab,de,kw,lnk,rn,ti,tn OR **'loratadine'/exp** OR ('7AJO3BO7QN' OR '79794-75-5' OR '4 (8 chloro 5, 6 dihydro 11h benzo [5, 6] cyclohepta [1, 2 b] pyridin 11 ylidene) 1 piperidinecarboxylic acid ethyl ester' OR '4-(8-Chloro-5,6-dihydro-11H-benzo(5,6)cyclohepta(1,2-b)pyridin-11-ylidene)-1-piperidinecarboxylic Acid Ethyl Ester' OR '4-(8-CHLORO-5,6-DIHYDRO-11H-BENZO[5,6]CYCLOHEPTA[1,2-B]PYRIDIN-11-YLIDENE)-1-PIPERIDINECARBOXYLATE' OR '4-(8-chloro-5,6-dihydro-11H-benzo[5,6]cyclohepta[1,2-b]pyridin-11-ylidene)-1-piperidinecarboxylic acid ethyl ester' OR '4-(8-Chloro-5,6-dihydro-11H-benzo[5,6]cyclohepta[1,2-b]pyridin-11-ylidene-1-piperidinecarboxylic acid ethyl ester' OR '8 chloro 11 (1 ethoxycarbonyl 4 piperidylidene) 6, 11 dihydro 5h benzo [5, 6] cyclohepta [1, 2 b] pyridine' OR '8 chloro 6, 11 dihydro 11 (1 carboethoxy 4 piperidylidene) 5h benzo [5, 6] cyclohepta [1, 2 b] pyridine' OR '8-chloro-11-(1-ethoxycarbonyl-4-piperidylidene)-6,11-dihydro-5H-benzo[5,6]cyclohepta[1,2-b]pyridine' OR '8-chloro-6,11-dihydro-11-(1- ethoxycarbonyl-4-piperidylidene)-5H-benzo[5,6]cyclohepta[1,2-b]pyridine' OR 'Aerotina' OR 'Alarin' OR 'Alavert' OR 'alerfast' OR 'alernitis' OR 'Alerpriv' OR 'alertadin' OR 'alertrin' OR 'allerta' OR 'Allertidin' OR 'allertyn' OR 'allohex' OR 'ambrace' OR 'analergal' OR 'Anhissen' OR 'anlos' OR 'ardin' OR 'Bactimicina allergy' OR 'Bedix Loratadina' OR 'Biloina' OR 'Bonalerg' OR 'caradine' OR 'carin' OR 'Civeran' OR 'clalodine' OR 'Claratyne' OR 'clarid' OR 'Clarinase' OR 'Claritin' OR 'Claritine' OR 'Clarityn' OR 'Clarityne' OR 'Clarium' OR 'cronitin' OR 'Cronopen' OR 'curyken' OR 'demazin anti-allergy' OR 'ethyl 4 (8 chloro 5, 6 dihydro 11h benzo [5, 6] cyclohepta [1, 2 b] pyridin 11 ylidene) 1 piperidinecarboxylate' OR 'Ethyl 4-(8-chloro-5,6-dihydro-11H-benzo(5,6)cyclohepta(1,2-b)pyridin-11-ylidene)-1-piperidinecarboxylate' OR 'Ethyl 4-(8-chloro-5,6-dihydro-11H-benzo[5,6]cyclohepta[1,2-b]pyridin-11-ylidene)-1-piperidinecarboxylate' OR 'ethyl 4-(8-chloro-5,6-dihydro-11H-benzo[5,6]cyclohepta[1,2-b]pyridin-11-ylidene)piperidine-1-carboxylate' OR 'Ethyl-4-(8-chloro-5,6-dihydro-11H-benzo [5,6] cyclohepta [1,2-b]pyridin-11-ylidene)-1-piperidinecarboxylate' OR 'ezasmin' OR 'ezede' OR 'finska' OR 'Flonidan' OR 'frenaler' OR 'Fristamin' OR 'genadine' OR 'halodin' OR 'hislorex' OR 'histalor' OR 'Histaloran' OR 'j-tadine' OR 'klarihist' OR 'Klaritin' OR 'klinset' OR 'laredine' OR 'lergia' OR 'Lergy' OR 'Lertamine' OR 'Lesidas' OR 'lindine' OR 'Lisino' OR 'lisono' OR 'lobeta' OR 'lodain' OR 'Lomilan' OR 'lorabasics' OR 'Loracert' OR 'loraclar' OR 'loraderm' OR 'Loradex' OR 'Loradif' OR 'loradin' OR 'lorahist' OR 'loralerg' OR 'lora-lich' OR 'lorano' OR 'Loranox' OR 'Lorantis' OR 'lorapaed' OR 'Lorastine' OR 'lora-tabs' OR 'loratadine' OR 'Loratadinum' OR 'loratadura' OR 'loratan' OR 'loratazine' OR 'loratidin' OR 'Loratidine' OR 'loraton' OR 'loratrim' OR 'Loratyne' OR 'Loraver' OR 'loraxin' OR 'loreen' OR 'Lorfast' OR 'lorihis' OR 'lorita' OR 'Loritine' OR 'lotadine' OR 'lotarin' OR 'Lowadina' OR 'mosedin' OR 'noratin' OR 'notamin' OR 'Nularef' OR 'onemin' OR 'Optimin' OR 'Polaratyne' OR 'proactin' OR 'Restamine' OR 'Rhinase' OR 'ridamin' OR 'rihest' OR 'rinityn' OR 'Rinolan' OR 'Rinomex' OR 'rityne' OR 'Roletra' OR 'Sanelor' OR 'Sch 29851' OR 'Sch29851' OR 'Sensibit' OR 'Sinhistan Dy' OR 'Sohotin' OR 'Symphoral' OR 'Tadine' OR 'Talorat Dy' OR 'tidilor' OR 'tirlor' OR 'toradine' OR 'Velodan' OR 'Versal' OR 'voratadine' OR 'Zeos'):ab,de,kw,lnk,rn,ti,tn OR **'novaluron'/exp** OR ('Z8H1B3CW0B' OR '116714-46-6' OR '1-(3-chloro-4-(1,1,2-trifluoro-2-trifluoromethoxyethoxy)phenyl)-3-(2,6-difluorobenzoyl)urea' OR '1-[3-Chloro-4-(1,1,2-trifluoro-2-trifluoromethoxyethoxy)phenyl]-3-(2,6-difluorobenzoyl)urea' OR '1-{3-CHLORO-4-[1,1,2-TRIFLUORO-2-(TRIFLUOROMETHOXY)ETHOXY]PHENYL}-3-(2,6-DIFLUOROBENZOYL)UREA' OR 'Novaluron' OR 'Rimon EC-10'):ab,de,kw,lnk,rn,ti,tn OR **'2 hydroxybiphenyl'/exp** OR ('90-43-7' OR '61788-42-9' OR 'D343Z75HT8' OR '1-Hydroxy-2-phenylbenzene' OR '2 biphenylol' OR '2 hydroxydiphenyl' OR '2 phenylphenol' OR '2-Biphenylol' OR '2-hydroxy biphenyl' OR '2-Hydroxy-1,1-biphenyl' OR '2-Hydroxybiphenyl' OR '2-Hydroxydiphenyl' OR '2-Phenyl phenol' OR '2-Phenylphenol' OR 'Amocid' OR 'Anthrapole 73' OR 'Biphenyl-2-ol' OR 'Biphenylol' OR 'Dowicide' OR 'Hydroxy-2-phenylbenzene' OR 'Hydroxybiphenyl' OR 'Invalon OP' OR 'Lyorthol' OR 'Nectryl' OR 'Nipacide OPP' OR 'o-Biphenylol' OR 'o-Diphenylol' OR 'o-Hydroxybiphenyl' OR 'o-Hydroxydiphenyl' OR 'o-Phenyl phenol' OR 'o-phenylphenate' OR 'o-phenylphenol' OR 'ortho hydroxybiphenyl' OR 'ortho hydroxydiphenyl' OR 'ortho phenylphenol' OR 'Orthohydroxydiphenyl' OR 'Orthophenyl phenol' OR 'ortho-phenylphenate' OR 'Orthophenylphenol' OR 'Orthoxenol' OR 'o-Xenol' OR 'o-Xonal' OR 'Preventol 3041' OR 'Preventol O extra' OR 'Remol TRF' OR 'Rotoline' OR 'sodium o-phenylphenoate' OR 'Stellisept' OR 'Tetrosin oe' OR 'Torsite' OR 'Xenol'):ab,de,kw,lnk,rn,ti,tn OR **'propylene glycol'/exp** OR ('6DC9Q167V3' OR '57-55-6' OR '123120-98-9' OR '63625-56-9' OR '1, 2 dihydroxypropane' OR '1, 2 propandiol' OR '1, 2 propanediol' OR '1, 2 propylenglycol' OR '1, 2-propanediol' OR '1,2 Propanediol' OR '1,2-(RS)-Propanediol' OR '1,2-dihydroxypropan-2-yl' OR '1,2-dihydroxypropane' OR '1,2-Dihydroxypropanl' OR '1,2-propandiol' OR '1,2-propane diol' OR '1.2-propanediol' OR '2,3-Propanediol' OR '2-Hydroxypropanol' OR 'Aliphatic alcohol' OR 'apopropanediol' OR 'Ilexan P' OR 'Isopropylene glycol' OR 'methyl ethyl glycol' OR 'methyl glycol' OR 'Methylethyl glycol' OR 'Methylethylene glycol' OR 'methylglycol' OR 'Monopropylene glycol' OR 'Prolugen' OR 'propan 1, 2 diol' OR 'Propan-1,2-Diol' OR 'propane 1, 2 diol' OR 'propane-1,2-diol' OR 'propanediol' OR 'propylene glycol' OR 'propyleneglycol' OR 'propylenglycol' OR 'Sirlene' OR 'Trimethyl glycol'):ab,de,kw,lnk,rn,ti,tn OR **'triclopyr'/exp** OR ('MV06PHJ6I0' OR '55335-06-3' OR '3,5,6-TPA' OR '((3,5,6-trichloro-2-pyridinyl)oxy)-acetic acid' OR '[(3,5,6-trichloro-2-pyridinyl)oxy]-acetic acid' OR '3,5,6-Trichloro-2-pyridinyloxyacetic Acid' OR '((3,5,6-trichloro-2-pyridyl)oxy)-acetic acid' OR '3,5,6-Trichloro-2-pyridyloxyacetic acid' OR 'Garlon' OR 'Grazon ET' OR 'Trichlopyr' OR 'Triclopyr' OR 'Turflon'):ab,de,kw,lnk,rn,ti,tn OR **'triethylene glycol'/exp** OR ('112-27-6' OR '103734-98-1' OR '122784-99-0' OR '137800-98-7' OR '145112-98-7' OR '3P5SU53360' OR '2, 2 ethylenedioxybis (ethanol)' OR '2,2-(Ethylenedioxy)diethanol' OR '2,2-Ethylenedioxybis(ethanol)' OR '2,2-Ethylenedioxydiethanol' OR '3, 6 dioxaoctane 1, 8 diol' OR '3,6-Dioxa-1,8-octanediol' OR '3,6-Dioxaoctane-1,8-diol' OR 'Bis(2-hydroxyethoxyethane)' OR 'Di-.beta.-hydroxyethoxyethane' OR 'Di-beta-hydroxyethoxyethane' OR 'Ethylene glycol dihydroxydiethyl ether' OR 'Ethylene glycol-bis-(2-hydroxyethyl ether)' OR 'Glycol bis(hydroxyethyl) ether' OR 'Tri-ethylene glycol' OR 'Triethylene Glycol' OR 'Triethyleneglycol' OR 'triethylenglycol' OR 'Trigenos' OR 'Triglycol' OR 'Trigol'):ab,de,kw,lnk,rn,ti,tn OR **'zoxamide'/exp** OR ('156052-68-5' OR 'RH 7281' OR 'RH7281' OR 'Zoxamid' OR 'Zoxamide' OR 'Zoxium'):ab,de,kw,lnk,rn,ti,tn OR **'pyriproxyfen'/exp** OR ('3Q9VOR705O' OR '95737-68-1' OR '126040-81-1' OR '2 [1 methyl 2 (4 phenoxyphenoxy) ethoxy] pyridine' OR '2-(1-Methyl-2-(4-phenoxyphenoxy)ethoxy)pyridine' OR '2-[ 1-methyl-2-(4-phenoxyphenoxy)ethoxy]pyridine' OR '2-[1-methyl-2-(4-phenoxyphenoxy)ethoxy] pyridine' OR '2-[1-Methyl-2-(4-phenoxyphenoxy)ethoxy]pyridine' OR '4-Phenoxyphenyl (RS)-2-(2-pyridyloxy)propyl ether' OR 'Archer IGR' OR 'Cyclio' OR 'juvinal' OR 'NyGuard IGR' OR 'Nylar' OR 'Pyriproxifen' OR 'Pyriproxyfen' OR 'S 31183' OR 'S31183' OR 'Sumilarv'):ab,de,kw,lnk,rn,ti,tn OR **'2 methoxyethanol'/exp** OR ('109-86-4' OR '9004-74-4' OR '95507-80-5' OR 'EK1L6XWI56' OR '1-Hydroxy-2-methoxyethane' OR '2-(methoxy)ethanol' OR '2-(methyloxy)ethanol' OR '2-HYDROXY-1-METHOXYETHYL' OR '2-Hydroxyethyl methyl ether' OR '2-methoxy ethanol' OR '2-Methoxy-1-ethanol' OR '2-Methoxyethan-1-Ol' OR '2methoxyethanol' OR '2-methoxyethanol' OR '2-Methoxyethyl alcohol' OR '2-methoxylethanol' OR '3-Oxa-1-butanol' OR 'alpha-hydro-omega-methoxypoly(oxyethylene)' OR 'beta-Methoxyethanol' OR 'dimethyleneglycol monomethylether' OR 'Dowanol 7' OR 'Dowanol EM' OR 'Ektasolve EM' OR 'Ethylene glycol methyl ether' OR 'Ethylene glycol monomethyl ether' OR 'ethylene glycol monomethylether' OR 'Ethyleneglycol monomethyl ether' OR 'ethyleneglycol monomethylether' OR 'ethylglycol monomethyl ether' OR 'Glycol ether EM' OR 'Glycol monomethyl ether' OR 'Glycolmethyl ether' OR 'Jeffersol EM' OR 'Karl Fischer Reagent' OR 'Methoxyhydroxyethane' OR 'Methyl cellosolve' OR 'Methyl ethoxol' OR 'Methyl icinol' OR 'Methyl oxitol' OR 'methylcellosolve' OR 'methylcello-solve' OR 'Monoethylene glycol methyl ether' OR 'Monomethyl ether of ethylene glycol' OR 'Monomethyl ethylene glycol ether' OR 'Monomethyl glycol' OR 'O-Methyl Glycol'):ab,de,kw,lnk,rn,ti,tn OR **'paracetamol'/exp** OR ('362O9ITL9D' OR '103-90-2' OR '4 hydroxyacetanilide' OR '4 hydroxyacetanilide' OR '4-(Acetylamino)phenol' OR '4-(N-Acetylamino)phenol' OR '4-acetamido phenol' OR '4-ACETAMIDOPHENYLOXIDANYL' OR 'A.F. Anacin' OR 'Abenol' OR 'Abensanil' OR 'Abrol' OR 'Abrolet' OR 'Acamol' OR 'Acenol' OR 'Acephen' OR 'Acertol' OR 'Acetaco' OR 'Acetagesic' OR 'Acetalgin' OR 'Acetamidophenol' OR 'acetamino phenol' OR 'Acetaminofen' OR 'acetaminophen' OR 'acetaminophene' OR 'acetaminophenol' OR 'Acetamol' OR 'Acetavance' OR 'Acetofen' OR 'acetomenophen' OR 'Acetominophen' OR 'acetominophene' OR 'acetylaminophenol' OR 'acetyl-p-aminophenol' OR 'Actamin' OR 'Actimol' OR 'adorem' OR 'Afebrin' OR 'Afebryl' OR 'Aferadol' OR 'Algesidal' OR 'algiafin' OR 'Algina' OR 'algocit' OR 'Algomol' OR 'Algotropyl' OR 'alphagesic' OR 'Alpiny' OR 'Alpinyl' OR 'Alvedon' OR 'Amadil' OR 'Aminofen' OR 'Anacin 3' OR 'Anacin3' OR 'anadin' OR 'Anaflon' OR 'analgiser' OR 'Analter' OR 'Anapap' OR 'Andox' OR 'Anelix' OR 'Anexsia' OR 'Anhiba' OR 'Antidol' OR 'Anuphen' OR 'Apacet' OR 'Apadon' OR 'Apamid' OR 'Apamide' OR 'APAP' OR 'apirex' OR 'Apitrelal' OR 'apotel' OR 'Arfen' OR 'Arthralgen' OR 'Asetam' OR 'Asomal' OR 'Aspac' OR 'Asplin' OR 'atamel' OR 'Atasol' OR 'Atralidon' OR 'Babikan' OR 'Bacetamol' OR 'Banesin' OR 'Benmyo' OR 'benuron' OR 'Ben-u-ron' OR 'Biocetamol' OR 'biogesic' OR 'bodrex' OR 'Bucet' OR 'Butapap' OR 'Cadafen' OR 'Calapol' OR 'Calmanticold' OR 'calodol' OR 'Calonal' OR 'Calpol' OR 'Capital with Codeine' OR 'Captin' OR 'Causalon' OR 'Cefalex' OR 'cemol' OR 'Cetadol' OR 'christamol' OR 'Citramon P' OR 'claradol' OR 'Claratal' OR 'Clixodyne' OR 'clocephen' OR 'Codabrol' OR 'Codalgin' OR 'Codapane' OR 'Codicet' OR 'Codisal' OR 'Codoliprane' OR 'Cofamol' OR 'Co-Gesic' OR 'Conacetol' OR 'Cosutone' OR 'cp 500' OR 'cp500' OR 'Cuponol' OR 'Curadon' OR 'Curpol' OR 'Dafalgan' OR 'Daphalgan' OR 'Darocet' OR 'Darvocet' OR 'Datril' OR 'Demilets' OR 'Deminofen' OR 'Democyl' OR 'Demogripal' OR 'depon' OR 'depyretin' OR 'Desfebre' OR 'Dhamol' OR 'Dimindol' OR 'Dirox' OR 'dismifen' OR 'Disprol' OR 'dolal' OR 'Dolcor' OR 'Dolefin' OR 'dolex' OR 'Dolgesic' OR 'Doliprane' OR 'dolitabs' OR 'Dolko' OR 'dolofen' OR 'Dolofugin' OR 'dolomol' OR 'Doloreduct' OR 'dolorol' OR 'Dolotec' OR 'dolotemp' OR 'Dolprone' OR 'doltem' OR 'Dorocoff' OR 'Dresan' OR 'drilan' OR 'dristan af' OR 'Duaneo' OR 'Dularin' OR 'Duorol' OR 'Duracetamol' OR 'Durapan' OR 'Dymadon' OR 'Ecosetol' OR 'efferalgan' OR 'efferalganodis' OR 'efferelgan' OR 'Elixodyne' OR 'Empracet' OR 'Endecon' OR 'Enelfa' OR 'Eneril' OR 'eraldor' OR 'eu med' OR 'Excipain' OR 'exopon' OR 'expandol' OR 'Fanalgic' OR 'Farmadol' OR 'Febranine' OR 'Febrectal' OR 'Febrectol' OR 'Febrex' OR 'Febricet' OR 'Febridol' OR 'Febrilix' OR 'Febrin' OR 'Febrinol' OR 'Febrolin' OR 'Fendon' OR 'Fensum' OR 'Fepanil' OR 'fervex' OR 'fibrinol' OR 'Finimal' OR 'Fluparmol' OR 'fortolin' OR 'Gelocatil' OR 'Geluprane' OR 'Genapap' OR 'Genebs' OR 'Grippostad' OR 'Gynospasmine' OR 'Hedex' OR 'helporal' OR 'Homoolan' OR 'Hydroxyacetanilide' OR 'Hy-Phen' OR 'Ildamol' OR 'Inalgex' OR 'infants feverall' OR 'Injectapap' OR 'Intensin' OR 'Janupap' OR 'kamolas' OR 'Kataprin' OR 'Korum' OR 'kyofen' OR 'Labamol' OR 'Lekadol' OR 'Lemgrip' OR 'Lemsip' OR 'Lestemp' OR 'letamol' OR 'Liqiprine' OR 'liquiprin' OR 'Lonarid' OR 'lotemp' OR 'Lupocet' OR 'Lyteca' OR 'Magnidol' OR 'Malgis' OR 'Malidens' OR 'Maxadol' OR 'medamol' OR 'Medocodene' OR 'meforagesic' OR 'Melabon Infantil' OR 'metagesic' OR 'metalid' OR 'Mexalen' OR 'Midol' OR 'Minafen' OR 'minopan' OR 'Minoset' OR 'Miralgin' OR 'Mono Praecimed' OR 'n acetyl 4 aminophenol' OR 'n acetyl para aminophenol' OR 'N-(4-Hydroxyphenyl)acetamide' OR 'N-(4-hydroxyphenyl)-acetamide' OR 'N-(4-Hydroxyphenyl)acetanilide' OR 'N-(4-hydroxyphenyl)ethanamide' OR 'n-acetyl-4-hydroxyaniline' OR 'N-Acetyl-p-aminophenol' OR 'Naldegesic' OR 'nalgesik' OR 'Napafen' OR 'napamol' OR 'NAPAP' OR 'naprex' OR 'Naprinol' OR 'Nealgyl' OR 'NeoCitran' OR 'neodalmin' OR 'Neodol' OR 'Neodolito' OR 'Neo-Fepramol' OR 'Neopap' OR 'Neuridon' OR 'nevral' OR 'nilapur' OR 'Nobedon' OR 'Nodolex' OR 'Noral' OR 'Norco' OR 'nysacetol' OR 'Ofirmev' OR 'Oltyl' OR 'Oralgan' OR 'Oraphen-PD' OR 'Ortensan' OR 'Oxycocet' OR 'p-(Acetylamino)phenol' OR 'Paceco' OR 'Pacemo' OR 'Pacemol' OR 'Pacet' OR 'p-Acetamidophenol' OR 'p-Acetaminophenol' OR 'p-Acetoaminophen' OR 'p-Acetylaminophenol' OR 'Pacimol' OR 'Paedialgon' OR 'Paedol' OR 'Painex' OR 'Paldesic' OR 'Pamol' OR 'Panacete' OR 'Panadeine' OR 'Panadiene' OR 'Panado-Co' OR 'Panadol' OR 'Panaleve' OR 'Panamax' OR 'Panasorb' OR 'Panasorbe' OR 'Panex' OR 'Panodil' OR 'Panofen' OR 'Pantalgin' OR 'para acetamidophenol' OR 'para acetylaminophenol' OR 'para hydroxyacetanilide' OR 'para suppo' OR 'Paracemol' OR 'Paracenol' OR 'Paracet' OR 'paracetaminophenol' OR 'Paracetamol' OR 'Paracetamole' OR 'Paracetamolum' OR 'Paracetanol' OR 'Paracetol' OR 'Paracin' OR 'Paracod' OR 'Paracodol' OR 'Parador' OR 'parageniol' OR 'paragin' OR 'Parakapton' OR 'Parake' OR 'Paralen' OR 'Paralief' OR 'Paralink' OR 'Paralyoc' OR 'paramax' OR 'paramidol' OR 'Paramol' OR 'Paramolan' OR 'Paranox' OR 'parapaed' OR 'Parapan' OR 'Parasedol' OR 'Parasin' OR 'Paraspen' OR 'paratabs' OR 'Para-Tabs' OR 'Parcetol' OR 'Parelan' OR 'Parmol' OR 'Parogal' OR 'Paroma' OR 'parvid' OR 'Pasolind' OR 'paximol' OR 'Pediapirin' OR 'Pediatrix' OR 'pedipan' OR 'Pedric' OR 'Perdolan Mono' OR 'Perfalgan' OR 'Phenaphen' OR 'Phendon' OR 'Phenipirin' OR 'Phogoglandin' OR 'Phrenilin' OR 'p-hydroxy-acetanilid' OR 'p-Hydroxyacetanilide' OR 'p-hydroxyacetoanilide' OR 'p-Hydroxyphenolacetamide' OR 'Pinex' OR 'Piramin' OR 'Pirinasol' OR 'Plicet' OR 'polarfen' OR 'Polmofen' OR 'Predimol' OR 'Prodol' OR 'Prontina' OR 'Propacet' OR 'Puernol' OR 'Pulmofen' OR 'Pyrigesic' OR 'Pyrinazine' OR 'Pyromed' OR 'Quiet World' OR 'raperon' OR 'rapidol' OR 'Redutemp' OR 'relaphen' OR 'Reliv' OR 'Remedol' OR 'Resfenol' OR 'Resprin' OR 'revanin' OR 'rhodapap' OR 'Rivalgyl' OR 'Robigesic' OR 'Rounox' OR 'Rubophen' OR 'Rupemol' OR 'Salzone' OR 'Sanicet' OR 'Sanicopyrine' OR 'Scanol' OR 'Sedalito' OR 'Sedapap' OR 'sedes a' OR 'Semolacin' OR 'serimol' OR 'Servigesic' OR 'Seskamol' OR 'Setakop' OR 'Setamol' OR 'Setol' OR 'Sifenol' OR 'Sinaspril' OR 'Sine-Aid' OR 'sinebriv' OR 'Sinedol' OR 'Sine-Off' OR 'Sinmol' OR 'sinpro' OR 'Sinubid' OR 'Snaplets-FR' OR 'St Joseph Aspirin-Free' OR 'Stanback' OR 'Stopain' OR 'Sunetheton' OR 'Supadol mono' OR 'Supofen' OR 'Suppap' OR 'Supramol-M' OR 'Tabalgin' OR 'tachipirin' OR 'tachipirina' OR 'taganopain' OR 'Talacen' OR 'Tapanol' OR 'Tapar' OR 'Tazamol' OR 'Tempanal' OR 'Tempra' OR 'tempte' OR 'Tencon' OR 'Termacet' OR 'Termalgin' OR 'Termalgine' OR 'Termofren' OR 'Theraflu' OR 'Tiffy' OR 'Titralgan' OR 'Toximer P' OR 'Tralgon' OR 'tramil' OR 'Treupel N' OR 'Treuphadol' OR 'Triaprin' OR 'Tricoton' OR 'turpan' OR 'Tussapap' OR 'Tycolet' OR 'Tylenol' OR 'Tylex' OR 'Tylol' OR 'Tylox' OR 'Tymol' OR 'Upsanol' OR 'Utragin' OR 'Valadol' OR 'Valgesic' OR 'Valorin' OR 'Veralgina' OR 'Vermidon' OR 'Verpol' OR 'Vicodin' OR 'Vivimed' OR 'Volpan' OR 'wegmal' OR 'winadol' OR 'winasorb' OR 'Wygesic' OR 'xebramol' OR 'Zatinol' OR 'Zolben' OR 'zydinol' OR 'Zydone'):ab,de,kw,lnk,rn,ti,tn OR **'ascorbic acid'/de** OR ('PQ6CK8PD0R' OR '50-81-7' OR '53262-66-1' OR '1-Xyloascorbic Acid' OR 'Acidum ascorbicum' OR 'acidylina' OR 'adenex' OR 'agrumina' OR 'allercorb' OR 'allescorb' OR 'Antiscorbic vitamin' OR 'Antiscorbutic factor' OR 'antiscorbutic vitamin' OR 'arcavit c' OR 'arcavite c' OR 'arkovital c' OR 'ascelat' OR 'ascofar' OR 'Ascoltin' OR 'ascomed' OR 'asconvita' OR 'ascor' OR 'ascorbate' OR 'ascorbic acid' OR 'ascorbicap' OR 'ascorbicin' OR 'ascorbico' OR 'ascorbin' OR 'ascorbina' OR 'ascorbinic acid' OR 'Ascorbinsaure' OR 'ascorbit' OR 'ascorbite' OR 'ascorbitol' OR 'ascorbivit' OR 'ascorbivite' OR 'ascorbone' OR 'ascorbutina' OR 'ascorbyl' OR 'ascorbyn' OR 'ascorgil' OR 'ascorin' OR 'ascormin' OR 'ascorteal' OR 'ascorval' OR 'ascorvel' OR 'ascorvit' OR 'ascorvite' OR 'ascorvitina' OR 'askorbin' OR 'austrovit c' OR 'austrovite c' OR 'bentavit c' OR 'bentavite c' OR 'c tamin' OR 'c vimin' OR 'c vit' OR 'c vita' OR 'cantan' OR 'cantaxin' OR 'catavin c' OR 'ce vi sol' OR 'cebetate' OR 'cebicure' OR 'Cebid' OR 'cebion' OR 'cebione' OR 'cecap' OR 'cecon' OR 'cecone' OR 'cecorbin' OR 'cecorbine' OR 'cecorbyl' OR 'cecorbyle' OR 'cecrisina' OR 'cedon' OR 'cedone' OR 'cedoxon' OR 'cedoxone' OR 'ceevifil' OR 'cegiolan' OR 'Ceklin' OR 'celaskon' OR 'celaskone' OR 'celin' OR 'Cemagyl' OR 'Cemill' OR 'cenetone' OR 'cenol' OR 'cenolate' OR 'cequinyl' OR 'cereon' OR 'cergona' OR 'cescorbat' OR 'cetamican' OR 'cetamid' OR 'cetamine' OR 'cetebe' OR 'Cetemican' OR 'ceterapion' OR 'ceterapione' OR 'cetrinets' OR 'cevalin' OR 'cevaline' OR 'cevatine' OR 'cevex' OR 'cevibid' OR 'Cevi-Bid' OR 'cevibram' OR 'cevigal' OR 'cevigen' OR 'cevigol' OR 'cevilat' OR 'cevimin' OR 'cevimine' OR 'cevisol' OR 'ce-vi-sol' OR 'cevit' OR 'cevita' OR 'Cevital' OR 'Cevitamate' OR 'cevitamic acid' OR 'cevitamin' OR 'cevitaminic acid' OR 'cevitan' OR 'cevite' OR 'cevitex' OR 'cevitil' OR 'cevitol' OR 'cewin' OR 'chewcee' OR 'chivibit c' OR 'Chromagen' OR 'ciamin' OR 'ciergin' OR 'cifilina' OR 'cipca' OR 'cisir' OR 'citamino' OR 'citoascorbina' OR 'citoxyl' OR 'citran' OR 'citravite' OR 'Citriscorb' OR 'citritabs' OR 'Citrovit' OR 'citrovitamina' OR 'civigor' OR 'civitin' OR 'civitine' OR 'Colascor' OR 'concemin' OR 'cortalex' OR 'c-vimin' OR 'dagrascorbin' OR 'dagravit c' OR 'dancimin c' OR 'davitamon c' OR 'dayvital' OR 'difvitamin c' OR 'dumovit c' OR 'dumovite c' OR 'Duoscorb' OR 'erftamin c' OR 'erftamine c' OR 'esuron' OR 'esurvit' OR 'esurvite' OR 'Ferancee' OR 'godabion c' OR 'gregovite c' OR 'hicee' OR 'hybrin' OR 'ido c' OR 'inovitan c' OR 'irocevit' OR 'irocevite' OR 'jarexin' OR 'jarexine' OR 'Juvamine' OR 'l 3 keto hexuronic acid lactone' OR 'L-3-ketothreohexuronic acid' OR 'lacivit' OR 'lacivite' OR 'laroscorbine' OR 'L-AscorbicAcid' OR 'leder c' OR 'lemascorb' OR 'limcee' OR 'L-lyxoascorbate' OR 'L-Lyxoascorbic acid' OR 'L-Threoascorbic acid' OR 'L-threo-Ascorbic acid' OR 'L-xyloascorbate' OR 'Magnorbin' OR 'myascorbin' OR 'natrascorb' OR 'novo ascorbic' OR 'nybadol' OR 'Parentrovite' OR 'parkovit c' OR 'pharmascorbine' OR 'pharmatovit c' OR 'pharmatovite c' OR 'planavit c' OR 'planavite c' OR 'plivit c' OR 'plivite c' OR 'proscorbin' OR 'proscorbine' OR 'redoxon' OR 'ribena' OR 'Rovimix C' OR 'scorbacid' OR 'scorbacide' OR 'scorbex' OR 'scorbin c' OR 'scorbitol' OR 'scorbumine' OR 'scottavit c' OR 'scottavite c' OR 'secorbate' OR 'Semidehydroascorbate' OR 'sevalin' OR 'sigmavit c' OR 'sigmavite c' OR 'sodascorbate' OR 'Sunkist' OR 'synum c' OR 'tanvimil-c' OR 'testascorbic' OR 'Tolfrinic' OR 'ucemine c' OR 'upsa-c' OR 'vicef' OR 'vicelat' OR 'vicetrin' OR 'viciman' OR 'vicin' OR 'vicitina' OR 'vicon' OR 'viforcit' OR 'viforcite' OR 'viscorin' OR 'viscorine' OR 'vitace' OR 'vitacee' OR 'vitacimin' OR 'vitacimine' OR 'vitacin' OR 'vitacine' OR 'vitamin C' OR 'Vitamisin' OR 'vitaplex c' OR 'vitapric' OR 'vitapur c' OR 'vitasan c' OR 'vitascorbin' OR 'vitascorbine' OR 'vitascorbol' OR 'vitelix c' OR 'wandervit c' OR 'wandervite c' OR 'witamina c' OR 'xitix' OR 'xyloascorbic acid'):ab,de,kw,lnk,rn,ti,tn OR **'butyl paraben'/exp** OR ('3QPI1U3FV8' OR '94-26-8' OR '4 hydroxybenzoic acid butyl ester' OR '4-(Butoxycarbonyl)phenol' OR '4-Hydroxybenzoic acid-n-butyl ester' OR 'Aseptoform butyl' OR 'butoben' OR 'butyl 4 hydroxybenzoate' OR 'butyl butex' OR 'butyl hydroxybenzoic acid' OR 'butyl para hydroxybenzoate' OR 'Butyl paraben' OR 'butyl parabenbutyl parahydroxybenzoatebutyl p-hydroxybenzoate' OR 'Butyl parahydroxybenzoate' OR 'Butyl p-hydroxybenzoate' OR 'butylhydroxybenzoate' OR 'butylparaben' OR 'n-Butyl hydroxybenzoate' OR 'Nipabutyl' OR 'para hydroxybenzoic acid butyl ester' OR 'p-Hydroxy butyl benzoate' OR 'p-Hydroxybenzoic acid butyl ester' OR 'p-Hydroxybenzoic acid n-butyl ester' OR 'p-Hydroxybenzoic butyl ester'):ab,de,kw,lnk,rn,ti,tn OR **'carbamazepine'/exp** OR ('33CM23913M' OR '298-46-4' OR '(5h) dibenz (b, f) azepine 5 carboxamide' OR '5 carbamoyl 5h dibenz [b, f] azepine' OR '5-Carbamoyl-5H-dibenz(b,f)azepine' OR '5-Carbamoyl-5H-dibenz[b,f]azepine' OR '5-Carbamoyl-5H-dibenzo(b,f)azepine' OR '5h dibenz [b, f] azepine 5 carboxamide' OR '5H-Dibenz(b,f)azepine-5-carboxamide' OR '5H-Dibenz[ b, f]azepine-5-carboxamide' OR '5H-Dibenz[b,f]azepine-5-carboxamide' OR '5H-Dibenzo[b,f]azepine-5-carboxamide' OR 'amizepin' OR 'amizepine' OR 'apo-carbamazepine' OR 'atretol' OR 'Bipotrol' OR 'biston' OR 'calepsin' OR 'camapine' OR 'carbadac' OR 'Carbamazepen' OR 'carbamazepin' OR 'carbamazepine' OR 'Carbamazepinum' OR 'Carbamezepine' OR 'carbategral' OR 'carbatol' OR 'carbatrol' OR 'carbazene' OR 'carbazep' OR 'Carbazepin' OR 'Carbazepine' OR 'carbazina' OR 'Carbelan' OR 'carmaz' OR 'carnexiv' OR 'carpaz' OR 'carzepin' OR 'carzepine' OR 'clostedal' OR 'convuline' OR 'epileptol' OR 'epimax' OR 'epitol' OR 'equetro' OR 'espa-lepsin' OR 'finlepsin' OR 'foxalepsin' OR 'g 32883' OR 'g32883' OR 'hermolepsin' OR 'Iminostilbene-N-carboxamide' OR 'karbamazepin' OR 'kodapan' OR 'lexin' OR 'mazepine' OR 'mazetol' OR 'neugeron' OR 'neurotol' OR 'neurotop' OR 'nordotol' OR 'Novo-Carbamaz' OR 'panitol' OR 'servimazepin' OR 'sirtal' OR 'spd 417' OR 'spd417' OR 'Stazepin' OR 'Stazepine' OR 'tardotol' OR 'taver' OR 'tegol' OR 'tegral' OR 'tegretal' OR 'tegretol' OR 'tegrital' OR 'telesmin' OR 'temporol' OR 'teril' OR 'timonil' OR 'Trimonil'):ab,de,kw,lnk,rn,ti,tn OR **'phthalic acid dimethyl ester'/exp** OR ('08X7F5UDJM' OR '131-11-3' OR 'avolin' OR 'citrola' OR 'Dimethyl benzeneorthodicarboxylate' OR 'Dimethyl o-phthalate' OR 'Dimethyl orthophthalate' OR 'dimethyl phthalate' OR 'dimethylphthalate' OR 'dmp 30' OR 'dmp30' OR 'fermine' OR 'Kemester DMP' OR 'Kodaflex DMP' OR 'methyl phthalate' OR 'mipax' OR 'mugia' OR 'palatinol m' OR 'Phthalic acid dimethyl ester' OR 'Repeftal' OR 'sketofax' OR 'Solvanom' OR 'Solvarone' OR 'Unimoll DM' OR 'Uniplex 110'):ab,de,kw,lnk,rn,ti,tn OR **'dimethylamine'/exp** OR ('124-40-3' OR '6912-12-5' OR 'ARQ8157E0Q' OR 'dimethlamine' OR 'dimethyamine' OR 'dimethyl amine' OR 'dimethylamine' OR 'di-methylamine' OR 'dimethylammonia' OR 'dimethylammonium chloride' OR 'dimethylammonium formate' OR 'dimethylarnine' OR 'dirnethylamine'):ab,de,kw,lnk,rn,ti,tn OR **'penicillamine'/exp** OR ('GNN1DV99GX' OR '52-67-5' OR '3, 3 dimethylcysteine' OR '3,3-Dimethyl-D(-)-cysteine' OR '3,3-Dimethyl-D-cysteine' OR '3-Mercapto-D-valine' OR '3-sulfanyl-D-valine' OR 'adaleen' OR 'alpha amino beta methyl beta mercaptobutyric acid' OR 'artamin' OR 'Artamine' OR 'atamir' OR 'beta, beta dimethylcysteamine' OR 'beta-Thiovaline' OR 'byanodine' OR 'Copper penicillaminate' OR 'cuprenil' OR 'cuprim' OR 'cuprimin' OR 'cuprimine' OR 'cuprimune' OR 'cupripen' OR 'Depamine' OR 'depen' OR 'dextropenicillamine' OR 'dimethyl cysteine' OR 'Dimethylcysteine' OR 'distamine' OR 'd-penamine' OR 'D-Penicilamine' OR 'D-Penicyllamine' OR 'd-penil' OR 'gerodyl' OR 'kelatin' OR 'kelatine' OR 'Kuprenil' OR 'Mercaptovaline' OR 'mercaptyl' OR 'metalcaptase' OR 'pemine' OR 'pendramine' OR 'penicillame' OR 'penicillamin' OR 'Penicillamina' OR 'penicillamine' OR 'Penicillaminum' OR 'penicillinamine' OR 'Perdolat' OR 'Sufirtan' OR 'Sufortan' OR 'sufortanon' OR 'trolovol'):ab,de,kw,lnk,rn,ti,tn OR **'folic acid'/exp** OR ('935E97BOY8' OR '59-30-3' OR '32108-06-8' OR 'acfol' OR 'Acidum folicum' OR 'Acifolic' OR 'Aspol' OR 'Cytofol' OR 'Dosfolat B activ' OR 'Facid' OR 'filicine' OR 'Folacid' OR 'folacin' OR 'Folan' OR 'folart' OR 'Folasic' OR 'folate' OR 'Folbal' OR 'Folcidin' OR 'Folcysteine' OR 'foldine' OR 'Folettes' OR 'foliamin' OR 'Folic' OR 'folicet' OR 'folicid' OR 'folinsyre' OR 'Folipac' OR 'folitab' OR 'folium acid' OR 'folivit' OR 'Folovit' OR 'Folsaeure' OR 'folsan' OR 'Folsaure' OR 'Folsav' OR 'folverlan' OR 'folvite' OR 'Folvron' OR 'gravi-fol' OR 'Incafolic' OR 'ingafol' OR 'lactobacillus casei factor' OR 'lafol' OR 'lexpec' OR 'megafol' OR 'Millafol' OR 'Mittafol' OR 'n [para [ (2 amino 4 hydroxy 6 pteridylmethyl) amino] benzoyl] glutamic acid' OR 'N-(p-(((2-Amino-4-hydroxy-6-pteridinyl)methyl)amino)benzoyl)-L-glutamic acid' OR 'N-{p-[(2-amino-4-hydroxypteridin-6-yl)methylamino]benzoyl}glutamic acid' OR 'neocepri' OR 'Novofolacid' OR 'nsc 3073' OR 'PteGlu' OR 'Pteroyglutamic acid' OR 'pteroyl glutamate' OR 'pteroyl l glutamic acid' OR 'pteroyl monoglutamate' OR 'pteroylglutamate' OR 'pteroylglutamic acid' OR 'Pteroyl-L-glutamate' OR 'Pteroyl-L-monoglutamate' OR 'Pteroyl-L-monoglutamic acid' OR 'pteroylmonoglutamate' OR 'pteroylmonoglutamic acid' OR 'rubiefol' OR 'vifolin' OR 'Vitamin B11' OR 'Vitamin B9' OR 'vitamin bc' OR 'vitamin m'):ab,de,kw,lnk,rn,ti,tn OR **'genistein'/exp** OR ('DH2M523P0H' OR '446-72-0' OR '690224-00-1' OR '4, 5, 7 trihydroxyisoflavone' OR '4,5, 7-Trihydroxyisoflavone' OR '4,5,7-Trihydroxy Iso-Flavone' OR '4,5,7-Trihydroxy isoflavone' OR '4,5,7-Trihydroxyisoflavone' OR '4,5,7-Trihydroxyisoflavone' OR 'Bonistein' OR 'differenol a' OR 'Genestein' OR 'genistein' OR 'genisteine' OR 'Genisteol' OR 'Genisterin' OR 'prunetol' OR 'Sophoricol'):ab,de,kw,lnk,rn,ti,tn OR **'methoxyacetic acid'/exp** OR ('625-45-6' OR 'F11T1H7Q7W' OR '(methyloxy)acetic acid' OR 'methoxy acetic acid' OR 'methoxyacetate' OR 'Methoxyacetic acid' OR 'methoxyessigs' OR 'Methoxyethanoic acid' OR 'methyloxyacetic acid'):ab,de,kw,lnk,rn,ti,tn OR **'1 methyl 2 pyrrolidinone'/exp** OR ('JR9CE63FPM' OR '872-50-4' OR '30207-69-3' OR '51013-18-4' OR '1 methyl 2 pyrrolidene' OR '1-methyl pyrrolidinone' OR '1-methyl-2-pyrolidinone' OR '1-Methylazacyclopentan-2-one' OR '1-Methylazacyclopentane-2-one' OR '1-methylpyrrolid-2-one' OR '1-methyl-pyrrolidin-2-one' OR '1-methylpyrrolidine-2-one' OR '1-N-methyl-2-pyrrolidinone' OR 'Agsolex 1' OR 'methyl pyrrolidone' OR 'Methyl-2-pyrrolidinone' OR 'methyl-2-pyrrolidone' OR 'methylpyrrolidin-2-one' OR 'Methylpyrrolidinone' OR 'Methylpyrrolidone' OR 'M-Pyrol' OR 'N-methyl 2-pyrolidone' OR 'N-methyl pirrolidone' OR 'N-methyl pyrollidone' OR 'N-methyl pyrrolidinone' OR 'n-methyl pyrrolidon' OR 'N-Methyl-.alpha.-pyrrolidinone' OR 'N-Methyl-.alpha.-pyrrolidone' OR 'N-Methyl-.gamma.-butyrolactam' OR 'N-methyl-2-pyrolidinone' OR 'N-methyl-2-pyrolidone' OR 'N-methyl-2-pyrrolidinon' OR 'N-Methyl-2-pyrrolidon' OR 'N-Methyl-alpha-pyrrolidinone' OR 'N-Methyl-alpha-pyrrolidone' OR 'n-methylbutyrolactam' OR 'N-Methyl-gamma-butyrolactam' OR 'N-methylpyrolidin-2-one' OR 'N-Methylpyrrolid-2-one' OR 'N-methyl-pyrrolid-2-one' OR 'N-methyl-pyrrolidin-2-one' OR 'N-methylpyrrolidin-2-one' OR 'N-methylpyrrolidine-2-one' OR 'N-Methylpyrrolidinon' OR 'N-Methylpyrrolidinone' OR 'N-Methylpyrrolidon' OR 'N-methyl-pyrrolidon' OR 'pharmasolve' OR 'Pyrol M'):ab,de,kw,lnk,rn,ti,tn OR **'1,1,1 trichloro 2 (2 chlorophenyl) 2 (4 chlorophenyl)ethane'/exp** OR ('D4K93Z1TBH' OR '789-02-6' OR '1 (2 chlorophenyl) 1 (4 chlorophenyl) 2, 2, 2 trichloroethane' OR '1 (ortho chlorophenyl) 1 (para chlorophenyl) 2, 2, 2 trichloroethane' OR '1, 1, 1 trichloro 2 (ortho chlorophenyl) 2 (para chlorophenyl) ethane' OR '1,1,1-trichloro-2-(2-chlorophenyl)-2-(4-chlorophenyl)ethane' OR '1,1,1-Trichloro-2-(o-chlorophenyl)-2-(p-chlorophenyl)ethane' OR '1-Chloro-2-(2,2,2-trichloro-1-(4-chlorophenyl)ethyl)benzene' OR '1-Chloro-2-[2,2,2-trichloro-1-(4-chlorophenyl)ethyl]benzene' OR '2 (2 chlorophenyl) 2 (4 chlorophenyl) 1, 1, 1 trichloroethane' OR '2 (ortho chlorophenyl) 2 (para chlorophenyl) 1, 1, 1 trichloroethane' OR '2-(o-chlorophenyl)-2-(p-chlorophenyl)-1,1,1-trichloroethane' OR '2-(2-Chlorophenyl)-2-(4-chlorophenyl)-1,1,1-trichloroethane' OR '2, 2, 2 trichloro 1 (2 chlorophenyl) 1 (4 chlorophenyl) ethane' OR '2,4-DDT' OR 'o, p ddt' OR 'o, p dichlorodiphenyltrichloroethane' OR 'o,p-DDT' OR 'o,p-DDT' OR 'o,p-dichlorodiphenyltrichloroethane' OR 'op ddt' OR 'ortho, para ddt' OR 'ortho,para-DDT'):ab,de,kw,lnk,rn,ti,tn OR **'rotenone'/exp** OR ('03L9OT429T' OR '83-79-4' OR 'Barbasco' OR 'Canex' OR 'Cubor' OR 'Dactinol' OR 'Deril' OR 'Derrin' OR 'Derris root' OR 'Extrax' OR 'Foliafume' OR 'Gerane' OR 'Haiari' OR 'Mexide' OR 'Nekoe' OR 'Nicouline' OR 'Noxfire' OR 'Noxfish' OR 'Nusyn' OR 'Paraderil' OR 'Prenfish' OR 'Prentox' OR 'protax' OR 'Ronone' OR 'Rotacide' OR 'Rotefive' OR 'Rotefour' OR 'Rotenoid' OR 'rotenon' OR 'rotenone' OR 'Rotenox' OR 'Roteonone' OR 'Rotessenol' OR 'Rotocide' OR 'Synpren' OR 'tubatoxin' OR 'Tubotoxin'):ab,de,kw,lnk,rn,ti,tn OR **'sucrose'/exp** OR ('57-50-1' OR '122880-25-5' OR '25702-74-3' OR '92004-84-7' OR 'C151H8M554' OR '1-alpha-D-glucopyranosyl-2-beta-D-fructofuranoside' OR 'alpha d glucopyranosyl beta d fructofuranoside' OR 'Amerfand' OR 'Amerfond' OR 'beet sugar' OR 'cane sugar' OR 'Granulated sugar' OR 'Microse' OR 'microtal' OR 'Polysucrose' OR 'sacarosa' OR 'saccharose' OR 'Saccharum' OR 'Sacharose' OR 'Sucraloxum' OR 'sucrose' OR 'Sugar spheres' OR 'tabfine' OR 'Table sugar' OR 'White sugar'):ab,de,kw,lnk,rn,ti,tn OR **'tetrabromobisphenol A'/exp** OR ('FQI02RFC3A' OR '79-94-7' OR 'bis(2,3-dibromopropylether)-2,2-bis(3,5-dibromo-4-(2,3-dibromopropoxy)phenyl)propane' OR 'Bromdian' OR 'Firemaster BP4A' OR 'TBBPA' OR 'tetrabromo 4, 4 isopropylidenediphenol' OR 'TETRABROMO-4,4-ISOPROPYLIDENEDIPHENOL' OR 'Tetrabromobisphenol A' OR 'TetrabromobisphenolA' OR 'Tetrabromodian' OR 'Tetrabromodiphenylopropane'):ab,de,kw,lnk,rn,ti,tn

**AND**

[<1966-2016]/py

Part 1 – **2,485 results**, 7/13/18

Part 2 – **1,871 results**, 7/13/18

**Final Mammalian PubMed Search Strategy**

"Rats"[Mesh] OR "Rabbits"[Mesh] OR Rattus[tw] OR Sprague-Dawley[tw] OR Wistar[tw] OR Long-Evans[tw] OR rat[tw] OR rats[tw] OR Oryctolagus[tw] OR rabbit[tw] OR rabbits[tw]

**AND**

"Embryology"[Mesh:NoExp] OR "Embryo Research"[Mesh] OR "Embryo, Mammalian"[Mesh:NoExp] OR "Fetus"[Mesh:NoExp] OR "Fetal Heart"[Mesh] OR "Embryonic and Fetal Development"[Mesh:NoExp] OR "Embryonic Development"[Mesh:NoExp] OR "Fetal Development"[Mesh] OR "Organogenesis"[Mesh:NoExp] OR "Fetal Organ Maturity"[Mesh] OR "embryology" [Subheading] OR "Mothers"[Mesh] OR "Maternal Exposure"[Mesh] OR "Pregnancy"[Mesh:NoExp] OR "Pregnancy, Animal"[Mesh:NoExp] OR "Maternal-Fetal Exchange"[Mesh] OR embryo*[tw] OR fetus*[tw] OR foetus*[tw] OR fetal*[tw] OR foetal*[tw] OR ductus arteriosus[tw] OR endocardial cushion*[tw] OR atrioventricular canal cushion*[tw] OR "truncus arteriosus"[tw] OR organogenesis[tw] OR mother*[tw] OR dam[tw] OR dams[tw] OR maternal*[tw] OR pregnancy[tw] OR pregnancies[tw] OR pregnant[tw] OR congenital*[tw] OR prenatal*[tw] OR pre-natal[tw] OR pre-natally[tw] OR intrauterine[tw] OR intra-uterine[tw] OR antenatal*[tw] OR gestation*[tw] OR transplacental exposure*[tw] OR (("Cementogenesis"[Mesh] OR ("Dental Cementum"[Mesh] AND ("1967/01/01"[PDAT] : "2001/12/31"[PDAT])) OR "Dentinogenesis"[Mesh] OR "Odontogenesis"[Mesh] OR "Lymphangiogenesis"[Mesh] OR "Musculoskeletal Development"[Mesh] OR ("Musculoskeletal System/growth and development"[Mesh] AND ("1991/01/01"[PDAT] : "2003/12/31"[PDAT])) OR "Face/growth and development"[Mesh] OR "Skull/growth and development"[Mesh] OR "Neurogenesis"[Mesh] OR "Brain/growth and development"[Mesh] OR "Eye/growth and development"[Mesh] OR "Sex Differentiation"[Mesh] OR "Gonads/growth and development"[Mesh] OR "Ovary/growth and development"[Mesh] OR "Testis/growth and development"[Mesh] OR "Heart/growth and development"[Mesh] OR "Kidney/growth and development"[Mesh] OR "Liver/growth and development"[Mesh] OR "Lung/growth and development"[Mesh] OR "organ development"[tw] OR tooth development[tw] OR tooth formation[tw] OR tooth growth[tw] OR tooth calcification[tw] OR tooth mineralization[tw] OR dental development[tw] OR dental formation[tw] OR cementogenesis[tw] OR cementification[tw] OR cementum formation[tw] OR dentinogenesis[tw] OR dentinogeneses[tw] OR dentification[tw] OR dentin formation[tw] OR dentogenesis[tw] OR odontogenesis[tw] OR odontogeneses[tw] OR amelogenesis[tw] OR amelogeneses[tw] OR enamel formation[tw] OR lymphangiogenesis[tw] OR lymphangiogeneses[tw] OR musculoskeletal development[tw] OR musculoskeletal system development[tw] OR limb development[tw] OR bone development[tw] OR bone growth[tw] OR physiologic calcification[tw] OR physiological calcification[tw] OR bone mineralization[tw] OR maxillofacial development[tw] OR craniofacial development[tw] OR face development[tw] OR facial development[tw] OR skull development[tw] OR cranial development[tw] OR skull growth[tw] OR osteogenesis[tw] OR osteogeneses[tw] OR bone formation[tw] OR ossification[tw] OR osteoclastogenesis[tw] OR osteoclastogeneses[tw] OR chondrogenesis[tw] OR chondrogeneses[tw] OR muscle development[tw] OR muscular development[tw] OR myogenesis[tw] OR myogeneses[tw] OR myofibrillogenesis[tw] OR myofibrillogeneses[tw] OR neurogenesis[tw] OR neurogeneses[tw] OR nervous system development[tw] OR neurologic development[tw] OR brain development[tw] OR brain cortex development[tw] OR cerebral development[tw] OR brain maturation[tw] OR brain maturity[tw] OR eye development[tw] OR retina development[tw] OR sex differentiation[tw] OR sexual differentiation[tw] OR gonad development[tw] OR gonadal development[tw] OR gonad differentiation[tw] OR gonadal differentiation[tw] OR sex gland development[tw] OR "sexual gland development"[tw] OR ovary development[tw] OR ovarian development[tw] OR ovary maturation[tw] OR ovarian maturation[tw] OR follicle development[tw] OR folliculogenesis[tw] OR "ovary follicle formation"[tw] OR ovarian follicle formation[tw] OR follicle maturation[tw] OR testis development[tw] OR testicle development[tw] OR testicular development[tw] OR testis descent[tw] OR "testicle descent"[tw] OR testicular descent[tw] OR "descensus testiculorum"[tw] OR "descensus testis"[tw] OR "testis descensus"[tw] OR heart development[tw] OR cardiac development[tw] OR heart growth[tw] OR kidney development[tw] OR kidney growth[tw] OR renal development[tw] OR renal growth[tw] OR liver development[tw] OR hepatic development[tw] OR lung development[tw] OR pulmonary development[tw]) AND (embryo*[tw] OR fetus*[tw] OR foetus*[tw] OR fetal*[tw] OR foetal*[tw] OR congenital*[tw] OR prenatal*[tw] OR pre-natal[tw] OR pre-natally[tw] OR intrauterine[tw] OR intra-uterine[tw] OR antenatal*[tw] OR gestation*[tw]))

**AND**

"Toxicology"[Mesh:NoExp] OR "Toxicological Phenomena"[Mesh:NoExp] OR "Hormesis"[Mesh] OR "Lethal Dose 50"[Mesh] OR "Inhibitory Concentration 50"[Mesh] OR ("Cell Survival/drug effects"[Mesh] AND ("1971/01/01"[PDAT] : "1998/12/31"[PDAT])) OR "Maximum Tolerated Dose"[Mesh] OR "No-Observed-Adverse-Effect Level"[Mesh] OR "Therapeutic Index"[Mesh] OR "Body Burden"[Mesh] OR "Toxicokinetics"[Mesh] OR "Toxicity Tests"[Mesh:NoExp] OR "Toxicity Tests, Acute"[Mesh:NoExp] OR "Toxicity Tests, Chronic"[Mesh] OR "Toxicity Tests, Subacute"[Mesh] OR "Toxicity Tests, Subchronic"[Mesh] OR "toxicity"[Subheading] OR "Poisons"[Mesh:NoExp] OR "Teratogens"[Mesh] OR "Teratogenesis"[Mesh] OR "Teratology"[Mesh] OR "Abnormalities, Drug-Induced"[Mesh] OR toxic*[tw] OR neurotoxi*[tw] OR hormesis[tw] OR hormeses[tw] OR hormetic[tw] OR lethal dos*[tw] OR fatal dos*[tw] OR LD10[tw] OR LD100[tw] OR LD50[tw] OR L.D.50[tw] OR LD 50[tw] OR LD90[tw] OR LD95[tw] OR LD99[tw] OR sublethal dos*[tw] OR inhibitory concentration 50[tw] OR IC50[tw] OR IC-50[tw] OR 50% inhibitory concentration[tw] OR inhibitory concentration 50%[tw] OR half maximal inhibitory concentration[tw] OR half maximum inhibitory concentration[tw] OR median inhibitory concentration[tw] OR maximum tolerated dos*[tw] OR maximally tolerated dos*[tw] OR maximal tolerated dos*[tw] OR maximum tolerable dos*[tw] OR maximal tolerable dos*[tw] OR maximally tolerable dos*[tw] OR maximum permissible dos*[tw] OR maximal permissible dos*[tw] OR maximum permissible exposure level*[tw] OR permissible level*[tw] OR permissible limit*[tw] OR maximum dos*[tw] OR maximal dos*[tw] OR no-observed-adverse-effect level*[tw] OR no-observed-adverse-effects level*[tw] OR NOAEL[tw] OR NOAELs[tw] OR no-observed-effect level*[tw] OR no-observed-effects level*[tw] OR no observable effect level*[tw] OR no observable effects level*[tw] OR no effect dose level*[tw] OR non-observed effect dose level*[tw] OR non-observed-effect level*[tw] OR no-observed-adverse-event level*[tw] OR "no-observed-adverse-events level"[tw] OR "no-observed-adverse-events levels"[tw] OR "no-observable-adverse-event level"[tw] OR "no-observable-adverse-event levels"[tw] OR "no-observable-adverse-events level"[tw] OR "no-observable-adverse-events levels"[tw] OR protective index*[tw] OR protective indices[tw] OR therapeutic index*[tw] OR therapeutic indices[tw] OR safety window*[tw] OR therapeutic ratio[tw] OR therapeutic ratios[tw] OR therapeutic window*[tw] OR "therapeutic drug index"[tw] OR "therapeutic drug indexes"[tw] OR "therapeutic drug indices"[tw] OR therapeutic drug window*[tw] OR toxic dos*[tw] OR TD50[tw] OR body burden*[tw] OR drug residue*[tw] OR pesticide residue*[tw] OR poison*[tw] OR teratogen*[tw] OR teratolog*[tw] OR teratomorph*[tw] OR teratotoxic*[tw] OR embryotoxi*[tw] OR fetotoxi*[tw] OR dysmorpholog*[tw] OR "drug-induced"[tw] OR malform*[tw] OR ((OECD guideline*[tw] OR OECD testing guideline*[tw] OR test guideline*[tw]) AND 414[tw]) OR "guideline 414"[tw] OR "guideline no. 414"[tw] OR "guideline number 414"[tw] OR "OECD no. 414"[tw] OR "OECD number 414"[tw] OR "OECD 414"[tw] OR "TG 414"[tw]

**AND**

**"alitretinoin" [Supplementary Concept]** OR "1UA8E65KDZ"[rn] OR "5300-03-8"[rn] OR "1UA8E65KDZ"[tw] OR "5300-03-8"[tw] OR "(9cis)-retinoic acid"[tw] OR "[3H]9-cis-retinoic acid"[tw] OR "9(Z)-Retinoic acid"[tw] OR "9-(Z)-retinoic Acid"[tw] OR "9-cis-RA"[tw] OR "9-cis-Retinoate"[tw] OR "9-cis-Retinoic acid"[tw] OR "9-cis-Tretinoin"[tw] OR "9CRA"[tw] OR "9C-RA"[tw] OR "9-CRA"[tw] OR "9cRA compound"[tw] OR "9-Retinoate"[tw] OR "9-Retinoic acid"[tw] OR "agn 192013"[tw] OR "agn192013"[tw] OR "Alitretinoin"[tw] OR "ALRT 1057"[tw] OR "ALRT1057"[tw] OR "BAL4079"[tw] OR "BAL-4079"[tw] OR "DB00523"[tw] OR "LG100057"[tw] OR "LG-100057"[tw] OR "LGD 100057"[tw] OR "LGD 1057"[tw] OR "lgd100057"[tw] OR "LGD1057"[tw] OR "nsc 659772"[tw] OR "nsc659772"[tw] OR "Panretin"[tw] OR "Panretyn"[tw] OR "Panrexin"[tw] OR "Ro-04-4079"[tw] OR "Toctino"[tw] OR **"Fluorouracil"[Mesh:NoExp]** OR "U3P01618RT"[rn] OR "51-21-8"[rn] OR "2, 4 dioxo 5 fluoropyrimidine"[tw] OR "5 fluoro 2, 4 pyrimidinedione"[tw] OR "5 fluoropyrimidine 2, 4 dione"[tw] OR "5 fu"[tw] OR "51-21-8"[tw] OR "5-Faracil"[tw] OR "5-florouracil"[tw] OR "5-Fluoracil"[tw] OR "5-Fluoracyl"[tw] OR "5-fluorouacil"[tw] OR "5-fluorourasil"[tw] OR "5-Fluracil"[tw] OR "5-Ftouracyl"[tw] OR "5FU"[tw] OR "5F-uracil"[tw] OR "5-HU Hexal"[tw] OR "accusite"[tw] OR "actino-hermal"[tw] OR "Adrucil"[tw] OR "agicil"[tw] OR "Arumel"[tw] OR "Carac"[tw] OR "Carzonal"[tw] OR "cinkef-u"[tw] OR "Effluderm"[tw] OR "Efudex"[tw] OR "Efudix"[tw] OR "Efurix"[tw] OR "eurofluor"[tw] OR "F 6627"[tw] OR "f6627"[tw] OR "fivoflu"[tw] OR "Fluoro Uracil"[tw] OR "Fluoroblastin"[tw] OR "Fluoroplex"[tw] OR "Fluorouracil"[tw] OR "Fluorouracile"[tw] OR "Fluoro-uracile"[tw] OR "Fluorouracilo Ferrer Far"[tw] OR "Fluoruracil"[tw] OR "Fluouracil"[tw] OR "fluoxan"[tw] OR "flurablastin"[tw] OR "Fluracedyl"[tw] OR "Fluracil"[tw] OR "fluracilium"[tw] OR "Fluracilum"[tw] OR "Fluri"[tw] OR "Fluril"[tw] OR "Fluro Uracil"[tw] OR "Fluroblastin"[tw] OR "fluroblastine"[tw] OR "Flurodex"[tw] OR "Ftoruracil"[tw] OR "Haemato-FU"[tw] OR "ifacil"[tw] OR "Kecimeton"[tw] OR "Neofluor"[tw] OR "nsc 18913"[tw] OR "nsc18913"[tw] OR "NSC19893"[tw] OR "NSC-19893"[tw] OR "oncofu"[tw] OR "Onkofluor"[tw] OR "Phthoruracil"[tw] OR "Phtoruracil"[tw] OR "Queroplex"[tw] OR "Ribofluor"[tw] OR "ro2 9757"[tw] OR "Ro-29757"[tw] OR "Ro-2-9757"[tw] OR "Timazin"[tw] OR "Tolak"[tw] OR "U3P01618RT"[tw] OR "uflahex"[tw] OR "utoral"[tw] OR **"Lovastatin"[Mesh]** OR "9LHU78OQFD"[rn] OR "75330-75-5"[rn] OR "6alpha-Methylcompactin"[tw] OR "6-alpha-Methylcompactin"[tw] OR "6-Methylcompactin"[tw] OR "75330-75-5"[tw] OR "9LHU78OQFD"[tw] OR "Advicor"[tw] OR "Altocor"[tw] OR "Altoprev"[tw] OR "Artein"[tw] OR "Belvas"[tw] OR "birotin"[tw] OR "Cholestra"[tw] OR "cid_53232"[tw] OR "Closterol"[tw] OR "Colevix"[tw] OR "cysin"[tw] OR "DB00227"[tw] OR "ellanco"[tw] OR "elstatin"[tw] OR "Hipolip"[tw] OR "Hipovastin"[tw] OR "l 654969"[tw] OR "L-154803"[tw] OR "Lestatin"[tw] OR "Lipdip"[tw] OR "Lipivas"[tw] OR "Lipofren"[tw] OR "Liposcler"[tw] OR "lofacol"[tw] OR "lomar"[tw] OR "lostatin"[tw] OR "lovacel"[tw] OR "lovacol"[tw] OR "lovahexal"[tw] OR "Lovalip"[tw] OR "Lovalord"[tw] OR "lovastan"[tw] OR "Lovastatin"[tw] OR "Lovasterol"[tw] OR "Lovastin"[tw] OR "lovatadin"[tw] OR "lowachol"[tw] OR "Lozutin"[tw] OR "medostatin"[tw] OR "Mevacor"[tw] OR "meverstin"[tw] OR "Mevinacor"[tw] OR "Mevinolin"[tw] OR "Mevlor"[tw] OR "mk 0803"[tw] OR "mk0803"[tw] OR "MK803"[tw] OR "MK-803"[tw] OR "Monacolin K"[tw] OR "Monakolin K"[tw] OR "msd 803"[tw] OR "neolipid"[tw] OR "Nergadan"[tw] OR "ovasta"[tw] OR "Paschol"[tw] OR "Rextat"[tw] OR "Rodatin"[tw] OR "Rovacor"[tw] OR "Sivlor"[tw] OR "Statosan"[tw] OR "Taucor"[tw] OR "Tecnolip"[tw] OR "Teroltrat"[tw] OR **"mono-(2-ethylhexyl)phthalate" [Supplementary Concept]** OR ("Phthalic Acids"[Mesh] AND ("1978/01/01"[PDAT] : "1979/12/31"[PDAT])) OR "4376-20-9"[rn] OR "FU2EWB60RT"[rn] OR "(2 ethylhexyl) phthalate"[tw] OR "(2-Ethylhexyl) hydrogen phthalate"[tw] OR "2 ethylhexyl phthalate"[tw] OR "2 ethylhexylphthalate"[tw] OR "2-Ethylhexyl hydrogen phthalate"[tw] OR "4376-20-9"[tw] OR "FU2EWB60RT"[tw] OR "MEHP"[tw] OR "mono (2 ethylhexyl) phthalate"[tw] OR "mono 2 ethylhexyl phthalate"[tw] OR "mono-(2-ethyl)hexyl phthalate"[tw] OR "Mono(2-ethylhexyl) phthalate"[tw] OR "Mono-(2-ethylhexyl) phthalate"[tw] OR "Mono(2-ethylhexyl)phthalate"[tw] OR "Mono-(2-ethylhexyl)phthalate"[tw] OR "mono(ethylhexyl) phthalate"[tw] OR "mono-ethylhexyl"[tw] OR "Monoethylhexyl phthalate"[tw] OR "Monoethylhexyl phthalic acid"[tw] OR "monoethylhexylphthalate"[tw] OR "mono-ethylhexylphthalate"[tw] OR "phthalic acid 2 ethylhexyl ester"[tw] OR "phthalic acid 2 ethylhexyl monoester"[tw] OR "phthalic acid mono (2 ethylhexyl) ester"[tw] OR "Phthalic Acid Mono(2-ethylhexyl) Ester"[tw] OR "PHTHALIC ACID MONO-2-ETHYLHEXYL ESTER"[tw] OR "Phthalic acid mono-2-ethylhexylester"[tw] OR "Phthalic acid, mono-(2-ethylhexyl) ester"[tw] OR "Phthalic acid, mono-2-ethylhexyl ester"[tw] OR "Phthalic Acid-d4 Mono(2-ethylhexyl) Ester"[tw] OR **"Caffeine"[Mesh]** OR "3G6A5W338E"[rn] OR "58-08-2"[rn] OR "95789-13-2"[rn] OR "1, 3, 7 trimethyl 2, 6 dioxopurine"[tw] OR "1,3,7-Trimethyl-2,6-dioxopurine"[tw] OR "1,3,7-Trimethylpurine-2,6-dione"[tw] OR "1,3,7-trimethylxanthine"[tw] OR "1,7-Trimethyl-2,6-dioxopurine"[tw] OR "1-methyltheobromine"[tw] OR "1-methyl-Theobromine"[tw] OR "3G6A5W338E"[tw] OR "58-08-2"[tw] OR "7-methyl Theophylline"[tw] OR "7-Methyltheophylline"[tw] OR "95789-13-2"[tw] OR "Alert-pep"[tw] OR "animine"[tw] OR "cafalgine"[tw] OR "Cafamil"[tw] OR "Cafecon"[tw] OR "Cafeina"[tw] OR "cafeine"[tw] OR "Cafergot"[tw] OR "Caffedrine"[tw] OR "Caffein"[tw] OR "Caffeina"[tw] OR "Caffeine"[tw] OR "Caffeinum"[tw] OR "Caffine"[tw] OR "Cafipel"[tw] OR "coffein"[tw] OR "Coffeine"[tw] OR "Coffeinum"[tw] OR "Darvon compound-65"[tw] OR "Dasin"[tw] OR "Dexitac"[tw] OR "DHCplus"[tw] OR "Durvitan"[tw] OR "Eldiatric C"[tw] OR "Enerjets"[tw] OR "Ercatab"[tw] OR "Guaranine"[tw] OR "guarin"[tw] OR "Hycomine"[tw] OR "Kofein"[tw] OR "Koffein"[tw] OR "Lanorinal"[tw] OR "Mateina"[tw] OR "Methyltheobromide"[tw] OR "Methyltheobromine"[tw] OR "Methylxanthine theophylline"[tw] OR "Miudol"[tw] OR "Nix Nap"[tw] OR "no doz"[tw] OR "Nodaca"[tw] OR "nodoz"[tw] OR "nymusa"[tw] OR "Organex"[tw] OR "P-A-C Analgesic Tablets"[tw] OR "pac compound"[tw] OR "Pep-Back"[tw] OR "Percoffedrinol N"[tw] OR "Percutafeine"[tw] OR "peyona"[tw] OR "Phensal"[tw] OR "Propoxyphene Compound 65"[tw] OR "Quick Pep"[tw] OR "QuickPep"[tw] OR "Respia"[tw] OR "SK-65 Compound"[tw] OR "teina"[tw] OR "Theine"[tw] OR "Tirend"[tw] OR "trimethylxanthine"[tw] OR "Vivarin"[tw] OR "Wigraine"[tw] OR **"Busulfan"[Mesh]** OR "G1LN9045DK"[rn] OR "55-98-1"[rn] OR "1, 4 bis (methanesulfonyloxy) butane"[tw] OR "1, 4 butanediol dimethanesulfonate"[tw] OR "1, 4 dimethanesulfonyloxybutane"[tw] OR "1, 4 dimethylsulfonyloxybutane"[tw] OR "1,4-Bis(methanesulfonoxy)butane"[tw] OR "1,4-Bis(methanesulfonyloxy)butane"[tw] OR "1,4-Butanedi yl dimethanesulfonate"[tw] OR "1,4-BUTANEDIOL DIMETHANESULFONATE"[tw] OR "1,4-Butanediol dimethanesulphonate"[tw] OR "1,4-Butanediol dimethylsulfonate"[tw] OR "1,4-butanedioldimethanesulfonate"[tw] OR "1,4-Butanediyl dimethanesulfonate"[tw] OR "1,4-Di(methylsulfonoxy)butane"[tw] OR "1,4-Dimesyloxybutane"[tw] OR "1,4-Dimethane sulfonyl oxybutane"[tw] OR "1,4-Dimethanesulfonoxybutane"[tw] OR "1,4-Dimethanesulfonoxylbutane"[tw] OR "1,4-Dimethanesulfonyloxybutane"[tw] OR "1,4-Dimethanesulphonyloxybutane"[tw] OR "1,4-Dimethylsulfonoxybutane"[tw] OR "1,4-Dimethylsulfonyloxybutane"[tw] OR "55-98-1"[tw] OR "Bisulfex"[tw] OR "Busilvex"[tw] OR "Busulfan"[tw] OR "Busulfano"[tw] OR "Busulfanum"[tw] OR "busulfex"[tw] OR "busulphan"[tw] OR "Busulphane"[tw] OR "butane-1,4-diyl dimethanesulfonate"[tw] OR "Butanedioldimethanesulfonate"[tw] OR "Buzulfan"[tw] OR "citosulfan"[tw] OR "cytoleukon"[tw] OR "G1LN9045DK"[tw] OR "glyzophrol"[tw] OR "krn 246"[tw] OR "krn246"[tw] OR "Leucosulfan"[tw] OR "mablin"[tw] OR "Mielevcin"[tw] OR "Mielosan"[tw] OR "mielucin"[tw] OR "Milecitan"[tw] OR "Mileran"[tw] OR "misulban"[tw] OR "mitistan"[tw] OR "mitosan"[tw] OR "mitostan"[tw] OR "muleran"[tw] OR "myelenkon"[tw] OR "myeleran"[tw] OR "Myeleukon"[tw] OR "myeloleukon"[tw] OR "Myelosan"[tw] OR "Myelosanum"[tw] OR "myeloxan"[tw] OR "myelucin"[tw] OR "mylecitan"[tw] OR "Mylecytan"[tw] OR "Myleran"[tw] OR "Mylerlan"[tw] OR "n-Butane-1,3-di(methylsulfonate)"[tw] OR "nsc 750"[tw] OR "NSC750"[tw] OR "Sulfabutin"[tw] OR "Sulphabutin"[tw] OR "tetramethylene dimesylate"[tw] OR **"Warfarin"[Mesh]** OR "129-06-6"[rn] OR "5Q7ZVV76EI"[rn] OR "81-81-2"[rn] OR "1 (4' hydroxy 3' coumarinyl) 1 phenyl 3 butanone"[tw] OR "129-06-6"[tw] OR "3 (alpha acetonylbenzyl) 4 hydroxycoumarin"[tw] OR "3 acetonylbenzonyl 4 hydroxy coumarinedimethylaminoethanol"[tw] OR "3 alpha phenyl beta acetylethyl 4 hydroxycoumarin"[tw] OR "3-(.alpha.-Acetonylbenzyl)-4-hydroxycoumarin"[tw] OR "3-(.alpha.-Phenyl-.beta.-acetylaethyl)-4-hydroxycumarin"[tw] OR "3-(.alpha.-Phenyl-.beta.-acetylethyl)-4-hydroxycoumarin"[tw] OR "3-(1'-Phenyl-2'-acetylethyl)-4-hydroxycoumarin"[tw] OR "3-(a-acetonylbenzyl)-4-hydroxycoumarin"[tw] OR "3-(Acetonylbenzyl)-4-hydroxycoumarin"[tw] OR "3-(alpha-Acetonylbenzyl)-4-hydroxycoumarin"[tw] OR "3-(alpha-Phenyl-beta-acetylaethyl)-4-hydroxycumarin"[tw] OR "3-(alpha-Phenyl-beta-acetylethyl)-4-hydroxycoumarin"[tw] OR "4-hydroxy-3-(3-oxo-1-phenylbutyl)-1-benzopyran-2-one"[tw] OR "4-Hydroxy-3-(3-oxo-1-phenylbutyl)-2H-1-benzopyran-2-one"[tw] OR "5Q7ZVV76EI"[tw] OR "81-81-2"[tw] OR "acetonylbenzylhydroxycoumarin"[tw] OR "adoisine"[tw] OR "Aldocumar"[tw] OR "alpha acetonylbenzyl 4 hydroxycoumarin dimethylaminoethanol"[tw] OR "antrombin k"[tw] OR "Athrombin"[tw] OR "athrombine k"[tw] OR "athrombinek"[tw] OR "befarin"[tw] OR "Brumolin"[tw] OR "carfin"[tw] OR "circuvit"[tw] OR "CO-Rax"[tw] OR "coumadan"[tw] OR "coumadin"[tw] OR "coumadine"[tw] OR "Coumafen"[tw] OR "coumafene"[tw] OR "Coumaphen"[tw] OR "coumaphene"[tw] OR "Coumefene"[tw] OR "Cov-R-Tox"[tw] OR "dagonal"[tw] OR "DB00682"[tw] OR "delta-con"[tw] OR "Dethmor"[tw] OR "Dethnel"[tw] OR "Dicusat E"[tw] OR "farin"[tw] OR "Frass-Ratron"[tw] OR "jantoven"[tw] OR "Kumader"[tw] OR "Kumadu"[tw] OR "kumatox"[tw] OR "Kypfarin"[tw] OR "maforan"[tw] OR "marevan"[tw] OR "Mar-Frin"[tw] OR "Maveran"[tw] OR "orfarin"[tw] OR "panwarfarin"[tw] OR "panwarfin"[tw] OR "Prothromadin"[tw] OR "Ratorex"[tw] OR "Ratox"[tw] OR "Ratoxin"[tw] OR "Ratron"[tw] OR "Rattunal"[tw] OR "Rodafarin"[tw] OR "Rosex"[tw] OR "Sewarin"[tw] OR "simarc-2"[tw] OR "Sofarin"[tw] OR "Solfarin"[tw] OR "Sorexa plus"[tw] OR "Tedicumar"[tw] OR "Temus W"[tw] OR "tintorane"[tw] OR "uniwarfin"[tw] OR "Vampirinip II"[tw] OR "Vampirinip iii"[tw] OR "wafarin"[tw] OR "waran"[tw] OR "Warf 10"[tw] OR "Warf 42"[tw] OR "Warfant"[tw] OR "warfar"[tw] OR "Warfarat"[tw] OR "Warfarin"[tw] OR "Warfarina"[tw] OR "warfarine"[tw] OR "Warfarinum"[tw] OR "Warficide"[tw] OR "warfil 5"[tw] OR "warfilone"[tw] OR "warnerin"[tw] OR "Zoocoumarin"[tw] OR **"Ketoconazole"[Mesh]** OR "R9400W927I"[rn] OR "142128-59-4"[rn] OR "65277-42-1"[rn] OR "1 [4 [4 [ [2 (2, 4 dichlorophenyl) 2 (1h imidazol 1 ylmethyl) 1, 3 dioxolan 4 yl] methoxy] phenyl] 1 piperazinyl] ethanone"[tw] OR "1 [4 [4 [ [2 (2, 4 dichlorophenyl) 2 [ (1h imidazol 1 yl) methyl] 1, 3 dioxolan 4 yl] methoxy] phenyl] piperazin 1 yl] ethan 1 one"[tw] OR "1 acetyl 4 [4 [ [2 (2, 4 dichlorophenyl) 2 (1h imidazol 1 ylmethyl) 1, 3 dioxolan 4 yl] methoxy] phenyl] piperazine"[tw] OR "142128-59-4"[tw] OR "4 (4 acetylpiperazin 1 yl) alpha [2 (2, 4 dichlorophenyl) 2 imidazol 1 ylmethyl 1, 3 dioxolan 4 yl] anisole"[tw] OR "65277-42-1"[tw] OR "akorazol"[tw] OR "anfuhex"[tw] OR "antanazol"[tw] OR "beatoconazole"[tw] OR "bigazol"[tw] OR "cetonax"[tw] OR "comozol"[tw] OR "conazol"[tw] OR "cremosan"[tw] OR "daktagold"[tw] OR "dezoral"[tw] OR "dio 902"[tw] OR "dio902"[tw] OR "extina"[tw] OR "formyco"[tw] OR "fugen"[tw] OR "funazole tabs"[tw] OR "funet"[tw] OR "fungarest"[tw] OR "fungaway"[tw] OR "fungazol tabs"[tw] OR "fungiderm-k"[tw] OR "funginoc"[tw] OR "funginox tabs"[tw] OR "fungoral"[tw] OR "kenazol"[tw] OR "kenazole"[tw] OR "kesnazol"[tw] OR "ketazol"[tw] OR "ketocanazole"[tw] OR "keto-comp"[tw] OR "ketoconazol"[tw] OR "Ketoconazole"[tw] OR "Ketoconazolum"[tw] OR "keto-crema"[tw] OR "ketoderm"[tw] OR "ketoisdin"[tw] OR "ketomed"[tw] OR "ketomicin"[tw] OR "ketomicol"[tw] OR "ketona"[tw] OR "keto-shampoo"[tw] OR "ketozal"[tw] OR "ketozol"[tw] OR "ketozole"[tw] OR "kezon"[tw] OR "konaturil"[tw] OR "Kuric"[tw] OR "kw 1414"[tw] OR "lusanoc"[tw] OR "micoral"[tw] OR "mizole"[tw] OR "mizoron"[tw] OR "mycofebrin"[tw] OR "nastil"[tw] OR "nazole"[tw] OR "neutrogena t/sal"[tw] OR "nisoral"[tw] OR "niz creme"[tw] OR "niz shampoo"[tw] OR "nizoral"[tw] OR "oxocanazole"[tw] OR "oxoconazole"[tw] OR "oxonazol"[tw] OR "panfungol"[tw] OR "pasalen"[tw] OR "picamic"[tw] OR "prenalon"[tw] OR "pristinex"[tw] OR "profungal"[tw] OR "r 41, 400"[tw] OR "R 41,400"[tw] OR "r 41400"[tw] OR "R41,400"[tw] OR "R41400"[tw] OR "R9400W927I"[tw] OR "sebizole"[tw] OR "sporium"[tw] OR "sporoxyl"[tw] OR "sporozol"[tw] OR "termizol"[tw] OR "terzolin"[tw] OR "triatop lotion"[tw] OR "Xolegel"[tw] OR "zoralin tabs"[tw] OR "zorinax"[tw] OR **"Hydroxyurea"[Mesh]** OR ("Urea"[Mesh] AND ("1966/01/01"[PDAT] : "1966/12/31"[PDAT])) OR "X6Q56QN5QC"[rn] OR "127-07-1"[rn] OR "(HYDROXYCARBAMOYL)AMINYL"[tw] OR "127-07-1"[tw] OR "1-oxidanylurea"[tw] OR "aminohydroxamic acid"[tw] OR "biosupressin"[tw] OR "carbamic acid oxime"[tw] OR "carbamide oxide"[tw] OR "Carbamohydroxamic acid"[tw] OR "Carbamohydroximic acid"[tw] OR "Carbamohydroxyamic acid"[tw] OR "Carbamoyl oxime"[tw] OR "Carbamyl hydroxamate"[tw] OR "Carbomohydroxamic acid"[tw] OR "Carrbamoyl Oxime"[tw] OR "Cytodrox"[tw] OR "droxia"[tw] OR "Hidrix"[tw] OR "Hidroxicarbamida"[tw] OR "hydab"[tw] OR "hydrea"[tw] OR "Hydreia"[tw] OR "hydrine"[tw] OR "Hydroxicarbamidum"[tw] OR "hydroxy carbamide"[tw] OR "hydroxy urea"[tw] OR "hydroxyaminomethanamide"[tw] OR "Hydroxycarbamid"[tw] OR "hydroxycarbamide"[tw] OR "Hydroxycarbamidum"[tw] OR "Hydroxycarbamine"[tw] OR "hydroxyl urea"[tw] OR "Hydroxylurea"[tw] OR "Hydroxyurea"[tw] OR "Hydura"[tw] OR "Hydurea"[tw] OR "Idrossicarbamide"[tw] OR "Litaler"[tw] OR "litalir"[tw] OR "mylocel"[tw] OR "N-(Aminocarbonyl) Hydroxyamine"[tw] OR "N-(Aminocarbonyl)hydroxylamine"[tw] OR "N-Carbamoylhydroxylamine"[tw] OR "neodrea"[tw] OR "nsc 32065"[tw] OR "NSC32065"[tw] OR "oncocarbide"[tw] OR "onco-carbide"[tw] OR "oxycarbamide"[tw] OR "oxyrea"[tw] OR "oxyurea"[tw] OR "siklos"[tw] OR "X6Q56QN5QC"[tw] OR **"Valproic Acid"[Mesh]** OR ("Valerates"[Mesh] AND ("1966/01/01"[PDAT] : "1974/12/31"[PDAT])) OR "614OI1Z5WI"[rn] OR "99-66-1"[rn] OR "2 propylpentanoate"[tw] OR "2 propylpentanoic acid"[tw] OR "2 propylvalerate sodium"[tw] OR "2 propylvaleric acid"[tw] OR "2, 2 dipropyl acetic acid"[tw] OR "2-propyl-Pentanoate"[tw] OR "2-Propylpentanoic Acid"[tw] OR "2-PROPYL-PENTANOIC ACID"[tw] OR "2-PropylpentanoicAcid"[tw] OR "2-Propylvaleric acid"[tw] OR "4-Heptanecarboxylic acid"[tw] OR "614OI1Z5WI"[tw] OR "99-66-1"[tw] OR "absenor"[tw] OR "Acidum valproicum"[tw] OR "alpha propylvalerate"[tw] OR "alpha propylvaleric acid"[tw] OR "apilepsin"[tw] OR "atemperator"[tw] OR "Avugane"[tw] OR "Baceca"[tw] OR "convulex"[tw] OR "Convulsofin"[tw] OR "delepsine"[tw] OR "depacon"[tw] OR "depakene"[tw] OR "depakin"[tw] OR "depakine"[tw] OR "Depakote"[tw] OR "depalept"[tw] OR "deprakine"[tw] OR "Deproic"[tw] OR "di n propylacetate"[tw] OR "di n propylacetic acid"[tw] OR "di-n-propyl acetic acid"[tw] OR "diplexil"[tw] OR "Dipropyl Acetate"[tw] OR "dipropyl acetic acid"[tw] OR "dipropylacetate"[tw] OR "dipropylacetatic acid"[tw] OR "dipropylacetic acid"[tw] OR "diprosin"[tw] OR "Divalproex"[tw] OR "Encorate"[tw] OR "Epical"[tw] OR "epilam"[tw] OR "epilex"[tw] OR "epilim"[tw] OR "episenta"[tw] OR "Epival"[tw] OR "ergenyl"[tw] OR "espa valept"[tw] OR "Eurekene"[tw] OR "everiden"[tw] OR "goilim"[tw] OR "hexaquin"[tw] OR "kw 6066 n"[tw] OR "labazene"[tw] OR "leptilan"[tw] OR "leptilanil"[tw] OR "micropakine"[tw] OR "mylproin"[tw] OR "myproic acid"[tw] OR "n dipropylacetic acid"[tw] OR "orfil"[tw] OR "orfiril"[tw] OR "orlept"[tw] OR "petilin"[tw] OR "Propylisopropylacetic Acid"[tw] OR "Propylvaleric acid"[tw] OR "propymal"[tw] OR "Savicol"[tw] OR "sodium 2 propylpentanoate"[tw] OR "sodium 2 propylvalerate"[tw] OR "sodium di n propyl acetate"[tw] OR "sodium di n propylacetate"[tw] OR "sodium dipropyl acetate"[tw] OR "sodium dipropylacetate"[tw] OR "sodium n dipropylacetate"[tw] OR "stavzor"[tw] OR "valberg pr"[tw] OR "valcote"[tw] OR "Valdisoval"[tw] OR "valepil"[tw] OR "valeptol"[tw] OR "valerin"[tw] OR "valhel pr"[tw] OR "valoin"[tw] OR "valpakine"[tw] OR "valparin"[tw] OR "valporal"[tw] OR "valprax"[tw] OR "valpro"[tw] OR "valproate"[tw] OR "valprodura"[tw] OR "Valproic Acid"[tw] OR "valprosid"[tw] OR "valprotek"[tw] OR "valsup"[tw] OR "Vupral"[tw] OR **"Tretinoin"[Mesh]** OR ("Vitamin A"[Mesh] AND ("1966/01/01"[PDAT] : "1974/12/31"[PDAT])) OR "5688UTC01R"[rn] OR "302-79-4"[rn] OR "1 (8 carboxy 3, 7 dimethyl 1, 3, 5, 7 octatetraen 1 yl) 2, 6, 6 trimethyl 1 cyclohexene"[tw] OR "3, 7 dimethyl 9 (2, 6, 6 trimethyl 1 cyclohexen 1 yl) 2, 4, 6, 8 nonatetraenoic acid"[tw] OR "3, 7 dimethyl 9 (2, 6, 6 trimethyl 1 cyclohexen 1 yl) nona 2, 4, 6, 8 tetraen 1 oic acid"[tw] OR "3,7-Dimethyl-9-(2,6,6-trimethyl-1-cyclohexen-1-yl)-2,4,6,8-nonatetraenoic acid"[tw] OR "3,7-Dimethyl-9-(2,6,6-trimethyl-1-cyclohexene-1-yl)-2,4,6,8-nonatetraenoic acid"[tw] OR "3,7-dimethyl-9-(2,6,6-trimethyl-1-cyclohexenyl)nona-2,4,6,8-tetraenoic acid"[tw] OR "3,7-Dimethyl-9-(2,6,6-trimethylcyclohex-1-enyl)nona-2,4,6,8-all-trans-tetraenoic acid"[tw] OR "302-79-4"[tw] OR "5688UTC01R"[tw] OR "9-cis-RA"[tw] OR "Aberel"[tw] OR "Aberela"[tw] OR "acid a vit"[tw] OR "Acnavit"[tw] OR "Airol"[tw] OR "Aknefug"[tw] OR "Aknoten"[tw] OR "all-trans-Vitamin A1 acid"[tw] OR "alquingel"[tw] OR "alten"[tw] OR "altinac"[tw] OR "anhydroretinoic acid"[tw] OR "ar 623"[tw] OR "ar623"[tw] OR "atra"[tw] OR "atragen"[tw] OR "atralin"[tw] OR "avita"[tw] OR "avitcid"[tw] OR "Avitoin"[tw] OR "betarretin"[tw] OR "dermairol"[tw] OR "dermik a"[tw] OR "effederm"[tw] OR "epi aberel"[tw] OR "epiaberel"[tw] OR "eudyna"[tw] OR "facenol"[tw] OR "ilotycin-a"[tw] OR "locacid"[tw] OR "Nexret"[tw] OR "nsc 122758"[tw] OR "nsc122758"[tw] OR "prosome a cream"[tw] OR "reacel-a"[tw] OR "Refissa"[tw] OR "Renova"[tw] OR "Retacnyl"[tw] OR "retavit"[tw] OR "retiderma"[tw] OR "Retin A"[tw] OR "Retinoate"[tw] OR "Retinoic acid"[tw] OR "Retinova"[tw] OR "Retionic acid"[tw] OR "Retisol-A"[tw] OR "retrieve cream"[tw] OR "ro 01 5488"[tw] OR "ro 1 5488"[tw] OR "ro 15488"[tw] OR "ro015488"[tw] OR "ro15488"[tw] OR "stieva a"[tw] OR "stievaa"[tw] OR "tracne"[tw] OR "Trans-Retinoicacid"[tw] OR "trentin"[tw] OR "Tretin M"[tw] OR "tretinoin"[tw] OR "Tretinoinum"[tw] OR "TRETINON"[tw] OR "Vesanoid"[tw] OR "Vitamin A acid"[tw] OR "vitinoin"[tw] OR **"Vitamin A"[Mesh]** OR "11103-57-4"[rn] OR "68-26-8"[rn] OR "11103-57-4"[tw] OR "3, 7 dimethyl 9 (2, 6, 6 trimethyl 1 cyclohexen 1 yl) 2, 4, 6, 8 nonatetraen 1 ol"[tw] OR "3, 7 dimethyl 9 (2, 6, 6 trimethyl 1 cyclohexenyl) 2, 4, 6, 8 nonatetraen 1 ol"[tw] OR "3,7-Dimethyl-9-(2,6,6-trimethyl-1-cyclchexen-1-yl)-2,4,6,8-nonatetraen-1-ol"[tw] OR "3,7-Dimethyl-9-(2,6,6-trimethyl-1-cyclohexen-1-yl)-2,4,6,8-nonate-traen-1-ol"[tw] OR "3,7-Dimethyl-9-(2,6,6-trimethyl-1-cyclohexen-1-yl)-2,4,6,8-nonatetraen-1-ol, (all-E)-"[tw] OR "3,7-Dimethyl-9-(2,6,6-trimethyl-1-cyclohexen-1-yl)-2,4,6,8-nonatetraen-1-ol, all (E)-"[tw] OR "3,7-Dimethyl-9-(2,6,6-trimethyl-1-cyclohexenyl)-2,4,6,8-nonatetraen-1-ol"[tw] OR "3,7-dimethyl-9-(2,6,6-trimethyl-1-cyclohexenyl)-nona-2,4,6,8-tetraen-1-ol"[tw] OR "68-26-8"[tw] OR "a 313"[tw] OR "a mulsal"[tw] OR "a mulsin"[tw] OR "a mulsine"[tw] OR "a sol"[tw] OR "a vi pel"[tw] OR "a vitadit"[tw] OR "a vitan"[tw] OR "a313"[tw] OR "acrisina"[tw] OR "acrisine"[tw] OR "actifral a"[tw] OR "adatone"[tw] OR "Afaxin"[tw] OR "afaxine"[tw] OR "afilina"[tw] OR "afiline"[tw] OR "agiolan"[tw] OR "Agoncal"[tw] OR "alcovit a"[tw] OR "alfa monovite"[tw] OR "alfaergin"[tw] OR "alfaergine"[tw] OR "alfamin"[tw] OR "alfamine"[tw] OR "alfamonovit"[tw] OR "alfasir"[tw] OR "alfasole"[tw] OR "alfasterolo"[tw] OR "alfatar"[tw] OR "alfavena"[tw] OR "alfavene"[tw] OR "alfavitina"[tw] OR "alfavitine"[tw] OR "alfene"[tw] OR "alin"[tw] OR "all-trans-Retinyl alcohol"[tw] OR "Alphalin"[tw] OR "alphaline"[tw] OR "alphasterol"[tw] OR "amulsal"[tw] OR "A-Mulsal"[tw] OR "amulsin"[tw] OR "amulsine"[tw] OR "amulvit"[tw] OR "Anatola"[tw] OR "anavit"[tw] OR "Anti-infective vitamin"[tw] OR "Antixerophthalmic vitamin"[tw] OR "Aoral"[tw] OR "apexol"[tw] OR "Apostavit"[tw] OR "Aquasol A"[tw] OR "Aquasola"[tw] OR "Aquasynth"[tw] OR "arcavit A"[tw] OR "asol"[tw] OR "A-Sol"[tw] OR "asteril"[tw] OR "Atars"[tw] OR "aterapion"[tw] OR "Avibon"[tw] OR "avimin"[tw] OR "avimine"[tw] OR "avipel"[tw] OR "A-Vi-Pel"[tw] OR "avipur"[tw] OR "avitabiol"[tw] OR "avitadit"[tw] OR "avital"[tw] OR "avitaminum kolin"[tw] OR "avitan"[tw] OR "A-Vitan"[tw] OR "avitana"[tw] OR "avitane"[tw] OR "avite"[tw] OR "avitil"[tw] OR "avitina"[tw] OR "Avitol"[tw] OR "avogina"[tw] OR "avogine"[tw] OR "avoleum"[tw] OR "axerodina"[tw] OR "axerodine"[tw] OR "axerol"[tw] OR "axerophthol"[tw] OR "Axerophtholum"[tw] OR "axerophthylium"[tw] OR "bentavit a"[tw] OR "bentavite a"[tw] OR "biosterol"[tw] OR "biotan"[tw] OR "chivibit a"[tw] OR "Chocola A"[tw] OR "Cylasphere"[tw] OR "cytobiase"[tw] OR "dagravit a"[tw] OR "davitamon a"[tw] OR "difvitamin a"[tw] OR "Disatabs Tabs"[tw] OR "Dofsol"[tw] OR "Dohyfral A"[tw] OR "elageno a"[tw] OR "endo a"[tw] OR "envit a"[tw] OR "Epiteliol"[tw] OR "fletase"[tw] OR "gadeol"[tw] OR "gadol"[tw] OR "halivitan"[tw] OR "halivitane"[tw] OR "homagenets aoral"[tw] OR "Homagenets aorl"[tw] OR "hydrosol"[tw] OR "Hydrovit A"[tw] OR "ido a"[tw] OR "idratene"[tw] OR "inovitan a"[tw] OR "Lard Factor"[tw] OR "meditalfa"[tw] OR "mulsal a"[tw] OR "multamine"[tw] OR "oleovit a"[tw] OR "Oleovitamin A"[tw] OR "ophthalamin"[tw] OR "panvita"[tw] OR "Plivit A"[tw] OR "Prepalin"[tw] OR "prepaline"[tw] OR "preparato a"[tw] OR "primavit"[tw] OR "quotivit"[tw] OR "Retin-11,12-t2-ol (9CI)"[tw] OR "retinol"[tw] OR "Retinolo"[tw] OR "Retinolum"[tw] OR "Retinyl A"[tw] OR "retinyl alcohol"[tw] OR "Retrovitamin A"[tw] OR "ro a vit"[tw] OR "Rovimix A 500"[tw] OR "Sehkraft A"[tw] OR "Tegosphere VitA"[tw] OR "Testavol"[tw] OR "Thalasphere"[tw] OR "ucemine a"[tw] OR "vaconex"[tw] OR "Vaflol"[tw] OR "Vafol"[tw] OR "Veroftal"[tw] OR "viadenin"[tw] OR "vialpha"[tw] OR "Vi-Alpha"[tw] OR "viatate"[tw] OR "vidoma"[tw] OR "vitadone"[tw] OR "vitadral"[tw] OR "vitalen a"[tw] OR "vitalfa"[tw] OR "vitama"[tw] OR "Vitamin A"[tw] OR "Vitamin A1"[tw] OR "Vitamine A"[tw] OR "Vitaminum A"[tw] OR "vitaplex a"[tw] OR "vitapur a"[tw] OR "vitasan a"[tw] OR "Vitavel A"[tw] OR "vitpex"[tw] OR "Vogan"[tw] OR "wandervit a"[tw] OR "xerophthol"[tw] OR "Zinosan N"[tw] OR **"Aminopterin"[Mesh:NoExp]** OR "JYB41CTM2Q"[rn] OR "54-62-6"[rn] OR "4 amino 4 deoxyfolic acid"[tw] OR "4 amino 4 desoxyfolic acid"[tw] OR "4 amino 9 methylpteroylglutamic acid"[tw] OR "4 aminofolic acid"[tw] OR "4 aminomethylpteroylglutamic acid"[tw] OR "4 aminopteroylglutamic acid"[tw] OR "4-Amino-4-deoxypteroylglutamate"[tw] OR "4-Aminofolate"[tw] OR "4-Aminopteroyl- glutamic acid"[tw] OR "4-Aminopteroyl-<R>glutamic acid"[tw] OR "4-Aminopteroylglutamate"[tw] OR "4-Aminopteroylglutamic acid"[tw] OR "4-Aminopteroyl-glutamic acid"[tw] OR "54-62-6"[tw] OR "aminopterin"[tw] OR "Aminopterine"[tw] OR "Aminopterinum"[tw] OR "Aminotrexate"[tw] OR "JYB41CTM2Q"[tw] OR "n [para [ (2, 4 diaminopterid 6 ylmethyl) amino] benzoyl] glutamic acid"[tw] OR "nsc 739"[tw] OR "NSC739"[tw] OR "Pteramina"[tw] OR **"Methotrexate"[Mesh]** OR "YL5FZ2Y5U1"[rn] OR "59-05-2"[rn] OR "4 amino 10 methylfolic acid"[tw] OR "4 amino 10 methylpteroylglutamic acid"[tw] OR "4 amino n10 methylpteroylglutamic acid"[tw] OR "4-Aminomethylpteroylglutamic acid"[tw] OR "4-amino-N(10)-methylpteroylglutamic acid"[tw] OR "4-Amino-N(sup 10)-methylpteroylglutamic acid"[tw] OR "4-Amino-N10-methylpteroyl-L-glutamic acid"[tw] OR "59-05-2"[tw] OR "a methopterine"[tw] OR "Abitrexate"[tw] OR "amethopterin"[tw] OR "A-Methopterin"[tw] OR "amethopterine"[tw] OR "A-Methpterin"[tw] OR "ametopterine"[tw] OR "Antifolan"[tw] OR "Arbitrexate"[tw] OR "biotrexate"[tw] OR "Brimexate"[tw] OR "canceren"[tw] OR "CL 14377"[tw] OR "cl14377"[tw] OR "Emtexate"[tw] OR "emthexat"[tw] OR "emthexate"[tw] OR "emtrexate"[tw] OR "enthexate"[tw] OR "farmitrexat"[tw] OR "farmitrexate"[tw] OR "farmotrex"[tw] OR "Fauldexato"[tw] OR "Folex"[tw] OR "ifamet"[tw] OR "intradose MTX"[tw] OR "jylamvo"[tw] OR "Lantarel"[tw] OR "ledertrexate"[tw] OR "Lumexon"[tw] OR "maxtrex"[tw] OR "Medsatrexate"[tw] OR "Metatrexan"[tw] OR "metex"[tw] OR "methoblastin"[tw] OR "methohexate"[tw] OR "Methotextrate"[tw] OR "methotrate"[tw] OR "Methotrexat"[tw] OR "methotrexate"[tw] OR "methotrexato"[tw] OR "Methotrexatum"[tw] OR "methoxtrexate"[tw] OR "methrotrexate"[tw] OR "Methylaminopterin"[tw] OR "methylaminopterine"[tw] OR "Methylaminopterinum"[tw] OR "meticil"[tw] OR "metoject"[tw] OR "Metolate"[tw] OR "metothrexate"[tw] OR "Metotressato"[tw] OR "metotrexat"[tw] OR "metotrexate"[tw] OR "metotrexin"[tw] OR "metrex"[tw] OR "Metrotex"[tw] OR "Mexate"[tw] OR "mpi 5004"[tw] OR "mpi5004"[tw] OR "MTX hydrate"[tw] OR "n [4 [ (2, 4 diamino 6 pteridylmethyl) methylamino] benzoyl] glutamic acid"[tw] OR "N-(4-(((2,4-DIAMINO-6-PTERIDINYL)METHYL)METHYLAMINO)BENZOYL)L-GLUTAMIC ACID"[tw] OR "N-(4-(((2,4-Diamino-6-pteridinyl)methyl)methylamino)benzoyl)-L-glutamicacid"[tw] OR "N-[4-[[(2,4-Diamino-6-pteridinyl)methyl] methylamino]benzoyl]-L-glutamic acid"[tw] OR "N-[4-[[(2,4-diamino-6-pteridinyl)methyl]methylamino]benzoyl]-L-glutamic acid"[tw] OR "N-Bismethylpteroylglutamic acid"[tw] OR "neotrexate"[tw] OR "nordimet"[tw] OR "novatrex"[tw] OR "nsc 740"[tw] OR "NSC740"[tw] OR "Otrexup"[tw] OR "rasuvo"[tw] OR "reumatrex"[tw] OR "Rheumatrex"[tw] OR "Texate"[tw] OR "texorate"[tw] OR "Tremetex"[tw] OR "trexall"[tw] OR "Trexeron"[tw] OR "Trixilem"[tw] OR "xaken"[tw] OR "Xatmep"[tw] OR "YL5FZ2Y5U1"[tw] OR "zexate"[tw] OR **"Phenytoin"[Mesh]** OR "6158TKW0C5"[rn] OR "57-41-0"[rn] OR "630-93-3"[rn] OR "5, 5 diphenyl 2, 4 imidazolidinedione"[tw] OR "5, 5 diphenylglycolylurea"[tw] OR "5, 5 diphenylhydantoin"[tw] OR "5, 5' diphenylhydantoin"[tw] OR "5, 5 diphenylimidazoline 2, 4 dione"[tw] OR "5,5-di(phenyl)imidazolidine-2,4-dione"[tw] OR "5,5-diphenyl hydantoin"[tw] OR "5,5-Diphenyl-2,4-imidazolidinedione"[tw] OR "5,5-Diphenylhydantoin"[tw] OR "5,5-Diphenylimidazolidin-2,4-dione"[tw] OR "5,5-diphenylimidazolidine-2,4-dione"[tw] OR "5,5-Diphenyl-imidazolidine-2,4-dione"[tw] OR "57-41-0"[tw] OR "6158TKW0C5"[tw] OR "630-93-3"[tw] OR "alepsin"[tw] OR "aleviatin"[tw] OR "antilepsin"[tw] OR "Antisacer"[tw] OR "Auranile"[tw] OR "cansoin"[tw] OR "Causoin"[tw] OR "Citrullamon"[tw] OR "Citrulliamon"[tw] OR "Comital"[tw] OR "Comitoina"[tw] OR "Convul"[tw] OR "cumatil"[tw] OR "Danten"[tw] OR "Dantinal"[tw] OR "dantoin"[tw] OR "Dantoinal"[tw] OR "Dantoine"[tw] OR "denyl"[tw] OR "Difenin"[tw] OR "difetoin"[tw] OR "differenin"[tw] OR "difhydan"[tw] OR "Dihycon"[tw] OR "dihydan"[tw] OR "di-hydan"[tw] OR "Dihydantoin"[tw] OR "Dilabid"[tw] OR "Dilantin"[tw] OR "Dilantine"[tw] OR "Dillantin"[tw] OR "dintoin"[tw] OR "dintoina"[tw] OR "Diphantoin"[tw] OR "diphantoine"[tw] OR "Diphedal"[tw] OR "diphedan"[tw] OR "Diphenat"[tw] OR "Diphenin"[tw] OR "Diphenine"[tw] OR "Diphentoin"[tw] OR "Diphentyn"[tw] OR "diphenyl hydantoin"[tw] OR "Diphenylan"[tw] OR "diphenyldantoin"[tw] OR "Diphenylhydantoin"[tw] OR "Diphenylhydatanoin"[tw] OR "diphenytoin"[tw] OR "Di-Phetine"[tw] OR "ditoin"[tw] OR "Ditoinate"[tw] OR "ditomed"[tw] OR "Elepsindon"[tw] OR "Enkelfel"[tw] OR "Epamin"[tw] OR "Epanutin"[tw] OR "Epdantoin"[tw] OR "Epdantoine simple"[tw] OR "Epelin"[tw] OR "Epifenyl"[tw] OR "Epihydan"[tw] OR "Epilan D"[tw] OR "Epilantin"[tw] OR "epileptin"[tw] OR "Epinat"[tw] OR "Episar"[tw] OR "Epised"[tw] OR "Epsolin"[tw] OR "Eptal"[tw] OR "Eptoin"[tw] OR "felantin"[tw] OR "fenantoin"[tw] OR "Fenidantoin s"[tw] OR "Fenigramon"[tw] OR "Fenitoin"[tw] OR "Fentoin"[tw] OR "Fenylepsin"[tw] OR "fenytoin"[tw] OR "Fenytoine"[tw] OR "Hidan"[tw] OR "hidanil"[tw] OR "Hidantal"[tw] OR "Hidantilo"[tw] OR "Hidantina"[tw] OR "Hidantomin"[tw] OR "Hindatal"[tw] OR "Hydantal"[tw] OR "Hydantin"[tw] OR "hydantinal"[tw] OR "Hydantoinal"[tw] OR "Hydantol"[tw] OR "Ictalis simple"[tw] OR "Idantoil"[tw] OR "Idantoin"[tw] OR "Iphenylhydantoin"[tw] OR "Kessodanten"[tw] OR "Labopal"[tw] OR "Lehydan"[tw] OR "lepitoin"[tw] OR "Lepsin"[tw] OR "Minetoin"[tw] OR "Neos-Hidantoina"[tw] OR "Neosidantoina"[tw] OR "neosidantoina"[tw] OR "Novantoina"[tw] OR "Novophenytoin"[tw] OR "Om hidantoina simple"[tw] OR "Om-Hydantoine"[tw] OR "Oxylan"[tw] OR "Phanantin"[tw] OR "Phanatine"[tw] OR "Phenatine"[tw] OR "Phenatoine"[tw] OR "Phenhydan"[tw] OR "phenhydane"[tw] OR "Phenhydanin"[tw] OR "phenilep"[tw] OR "Phenitoin"[tw] OR "Phentoin"[tw] OR "Phentytoin"[tw] OR "phenybin"[tw] OR "phenydan"[tw] OR "phenydantin"[tw] OR "phenytek"[tw] OR "phenytex"[tw] OR "phenytoin"[tw] OR "Phenytoinum"[tw] OR "phenytonium"[tw] OR "pyoredol"[tw] OR "Ritmenal"[tw] OR "Saceril"[tw] OR "sanepil"[tw] OR "Silantin"[tw] OR "Sinergina"[tw] OR "Sodanthon"[tw] OR "Sodantoin"[tw] OR "sodanton"[tw] OR "Sodium Diphenylhydantoinate"[tw] OR "Solantin"[tw] OR "Solantoin"[tw] OR "solantyl"[tw] OR "Sylantoic"[tw] OR "Tacosal"[tw] OR "Thilophenyl"[tw] OR "Toin unicelles"[tw] OR "vasilcon"[tw] OR "Zentronal"[tw] OR "Zentropil"[tw] OR **"Ethylene Glycol"[Mesh]** OR "FC72KVT52F"[rn] OR "107-21-1"[rn] OR "1, 2 ethanediol"[tw] OR "1,2-dihydroxy ethane"[tw] OR "1,2-Dihydroxyethane"[tw] OR "1,2-Ethandiol"[tw] OR "1,2-ethane diol"[tw] OR "1,2-ethanediol"[tw] OR "1,2-ethyleneglycol"[tw] OR "107-21-1"[tw] OR "2-hydroxyethanol"[tw] OR "ethan-1,2-diol"[tw] OR "Ethane-1,2-diol"[tw] OR "ethane-1.2-diol"[tw] OR "ethanediol"[tw] OR "ethyl glycol"[tw] OR "ethylen glycol"[tw] OR "Ethylene alcohol"[tw] OR "Ethylene dihydrate"[tw] OR "Ethylene Glycol"[tw] OR "ethyleneglycol"[tw] OR "Ethylenglycol"[tw] OR "Etilenglicol"[tw] OR "etylene glycol"[tw] OR "FC72KVT52F"[tw] OR "Glycol alcohol"[tw] OR "glycol ethylene"[tw] OR "Hypodicarbonous acid"[tw] OR "Mono Ethylene Glycol"[tw] OR "monoethylene glycol"[tw] OR **"Thiotepa"[Mesh]** OR "905Z5W3GKH"[rn] OR "52-24-4"[rn] OR "52-24-4"[tw] OR "AI3-24916"[tw] OR "AI324916"[tw] OR "905Z5W3GKH"[tw] OR "Girostan"[tw] OR "Ledertepa"[tw] OR "methylenethiophosphoramide"[tw] OR "n, n', n'' triethylenethiophosphoramide"[tw] OR "NSC 6396"[tw] OR "nsc 6996"[tw] OR "NSC6396"[tw] OR "Oncotepa"[tw] OR "Oncothio-tepa"[tw] OR "oncotiotepa"[tw] OR "Phosphoric tri(ethyleneamide)"[tw] OR "Phosphorothioic acid triethylenetriamide"[tw] OR "PHOSPHOROTHIOIC TRI(ETHYLENEAMIDE)"[tw] OR "Stepa"[tw] OR "tepadina"[tw] OR "tespa"[tw] OR "Tespamin"[tw] OR "Tespamine"[tw] OR "thio tepa"[tw] OR "Thiofozil"[tw] OR "Thiophosphamide"[tw] OR "Thiophosphamidum"[tw] OR "Thioplex"[tw] OR "Thiotef"[tw] OR "Thio-Tep"[tw] OR "thiotepa"[tw] OR "Thiotepum"[tw] OR "thiotriethylenephosphoramide"[tw] OR "Tifosyl"[tw] OR "tio tef"[tw] OR "Tiofosfamid"[tw] OR "Tiofosyl"[tw] OR "Tiofozil"[tw] OR "trethylenethiophosphoramide"[tw] OR "Tri(1-aziridinyl)phosphine sulfide"[tw] OR "Tri(aziridin-1-yl)phosphine sulfide"[tw] OR "Tri(ethyleneimino)thiophosphoramide"[tw] OR "Tri-1-aziridinylphosphine sulfide"[tw] OR "Triaziridinylphosphine sulfide"[tw] OR "triethylene thio phosphoramide"[tw] OR "triethylene thiophosphamide"[tw] OR "triethylene thiophosphoramide"[tw] OR "triethylenethiophosphamide"[tw] OR "Triethylenethiophosphoramide"[tw] OR "Triethylenethiophosphorotriamide"[tw] OR "triethylenethiophosphortriamide"[tw] OR "tris (1 azaridinyl) phosphine sulfide"[tw] OR "tris (1 aziridinyl) phosphine sulfide"[tw] OR "tris (1 aziridinyl) phosphine sulphide"[tw] OR "tris (1 aziridinyl) phosphinsulfide"[tw] OR "tris (1 aziridinyl) phosphinsulphide"[tw] OR "tris (ethylenimino) thiophosphate"[tw] OR "Tris(1-aziridinyl)phosphine sulfide"[tw] OR "Tris(1-aziridinyl)phosphine sulphide"[tw] OR "tris(1-aziridinyl)-sulfanylidenephosphorane"[tw] OR "tris(aziridin-1-yl)-sulfanylidenephosphorane"[tw] OR "Tris(aziridinyl)phosphine sulfide"[tw] OR "Tris(aziridinyl)-phosphine sulfide"[tw] OR "Tris(ethylenimino)thiophosphate"[tw] OR **"Atrazine"[Mesh]** OR "QJA9M5H4IM"[rn] OR "1912-24-9"[rn] OR "1912-24-9"[tw] OR "2 chloro 4 ethylamino 6 isopropylamino 1, 3, 5 triazine"[tw] OR "2 chloro 4 ethylamino 6 isopropylamino s triazine"[tw] OR "2-Chloro-4-(ethylamino)-6-(isopropylamino)-1,3,5-triazine"[tw] OR "2-Chloro-4-(ethylamino)-6-(isopropylamino)-s-triazine"[tw] OR "2-Chloro-4-(ethylamino)-6-(isopropylamino)triazine"[tw] OR "2-Chloro-4-(ethylamino)-6-[(prop-2-yl)amino]-1,3,5-triazine"[tw] OR "2-Chloro-4-ethylamineisopropylamine-s-triazine"[tw] OR "2-Chloro-4-ethylamino-6-isopropylamino-1,3,5-triazine"[tw] OR "2-Chloro-4-ethylamino-6-isopropylamino-s-triazine"[tw] OR "2-chloro-4-ethyl-amino-6-isopropylamino-s-triazine"[tw] OR "2-CHLORO-4-ETHYLAMINO-6-ISOPROPYLAMINO-sym-TRIAZINE"[tw] OR "6 chloro n2 ethyl n4 isopropyl 1, 3, 5 triazine 2, 4 diamine"[tw] OR "6-Chloro-N2-ethyl-N4-isopropyl-1,3,5-triazine-2,4-diamine"[tw] OR "Aatram"[tw] OR "Aatrex"[tw] OR "Actinite PK"[tw] OR "Akticon"[tw] OR "Aktikon"[tw] OR "Aktinit A"[tw] OR "Aktinit PK"[tw] OR "Aneldazin"[tw] OR "Argezin"[tw] OR "Atazinax"[tw] OR "Atraflow"[tw] OR "Atranex"[tw] OR "Atrasine"[tw] OR "Atrataf"[tw] OR "Atratol"[tw] OR "Atrazin"[tw] OR "atrazine"[tw] OR "atrazinus"[tw] OR "Atred"[tw] OR "Atrex"[tw] OR "Attrex"[tw] OR "Azinotox 500"[tw] OR "Azoprim"[tw] OR "Candex"[tw] OR "Ceasin 50"[tw] OR "Cekuzina-T"[tw] OR "Chromozin"[tw] OR "Crisamina"[tw] OR "Crisatrina"[tw] OR "Crisazina"[tw] OR "Crisazine"[tw] OR "Cyazin"[tw] OR "Cyazine"[tw] OR "Farmozine"[tw] OR "Fenamin"[tw] OR "Fenamine"[tw] OR "Fenatrol"[tw] OR "Fogard"[tw] OR "Gesamprim"[tw] OR "Gesaprim"[tw] OR "Gesaprin"[tw] OR "Gesoprim"[tw] OR "Griffex"[tw] OR "Herbatoxol"[tw] OR "Hungazin"[tw] OR "Inakor"[tw] OR "Laddock"[tw] OR "Maizina"[tw] OR "Mebazine"[tw] OR "Oleogesaprim"[tw] OR "Pitezin"[tw] OR "Primatol"[tw] OR "Primaze"[tw] OR "Primitol A"[tw] OR "Primoleo"[tw] OR "QJA9M5H4IM"[tw] OR "Radazin"[tw] OR "Radizin"[tw] OR "Radizine"[tw] OR "Strazine"[tw] OR "Triazine A 1294"[tw] OR "Vectal"[tw] OR "Weedex A"[tw] OR "Wonuk"[tw] OR "Zeapho"[tw] OR "Zeaphos"[tw] OR "Zeapos"[tw] OR "Zeazin"[tw] OR "Zeazine"[tw] OR "zeazint"[tw] OR "Zeopos"[tw] OR **"dinoseb" [Supplementary Concept]** OR "88-85-7"[rn] OR "2 (1 methylpropyl) 4, 6 dinitrophenol"[tw] OR "2 sec butyl 4, 6 dinitrophenol"[tw] OR "2-(1-Methylpropyl)-4,6-dinitrophenol"[tw] OR "2-(1-Methylpropyl)-4,6-dinitro-Phenol"[tw] OR "2-(sec-Butyl)-4,6-dinitrophenol"[tw] OR "2, 4 dinitro 6 sec butylphenol"[tw] OR "2,4-Dinitro-6-sec-butylphenol"[tw] OR "2,4-dinitro-6-sec-butyl-phenol"[tw] OR "2-[1-methylpropyl]-4,6-dinitrophenol"[tw] OR "2-sec-Butyl-4,6-dinitrophenol"[tw] OR "2-Sec-butyl-4,6-dinitro-Phenol"[tw] OR "4, 6 dinitro 2 (1 methylpropyl) phenol"[tw] OR "4, 6 dinitro 2 sec butylphenol"[tw] OR "4,6-Dinitro-2-(1-methyl-n-propyl)phenol"[tw] OR "4,6-Dinitro-2-(1-methyl-propyl)phenol"[tw] OR "4,6-Dinitro-2-sec-butylphenol"[tw] OR "4,6-Dinitro-o-sec-butylphenol"[tw] OR "6 sec butyl 2, 4 dinitrophenol"[tw] OR "6-sec-Butyl-2,4-dinitrophenol"[tw] OR "88-85-7"[tw] OR "Aatox"[tw] OR "Aretit"[tw] OR "Basanite"[tw] OR "Blaartox"[tw] OR "Butaphene"[tw] OR "Caldon"[tw] OR "Chemsect"[tw] OR "Desicoil"[tw] OR "Dibutox"[tw] OR "Dinitrall"[tw] OR "Dinitrax"[tw] OR "dinitrobutylphenol"[tw] OR "Dinitro-ortho-sec-butyl phenol"[tw] OR "Dinitro-o-sec-butylphenol"[tw] OR "dinoseb"[tw] OR "Dynanap"[tw] OR "Dytop"[tw] OR "Elgetol 318"[tw] OR "Gebutox"[tw] OR "Hivertox"[tw] OR "Ivosit"[tw] OR "Kiloseb"[tw] OR "Ladob"[tw] OR "Laseb"[tw] OR "Nitropone C"[tw] OR "Phenotan"[tw] OR "Premerg"[tw] OR "Premerge"[tw] OR "Sinox general"[tw] OR "Sparic"[tw] OR "Spurge"[tw] OR "Subitex"[tw] OR "Tubotox"[tw] OR **"fluazinam" [Supplementary Concept]** OR "0P91PCK33Q"[rn] OR "79622-59-6"[rn] OR "0P91PCK33Q"[tw] OR "3-Chloro-N-(3-chloro-2,6-dinitro-4-(trifluoromethyl)phenyl)-5-(trifluoromethyl)-2-pyridinamine"[tw] OR "3-Chloro-N-(3-chloro-2,6-dinitro-4-(trifluoromethyl)phenyl)-5-(trifluoromethyl)pyridin-2-amine"[tw] OR "3-chloro-N-(3-chloro-2,6-dinitro-4-trifluoromethylphenyl)-5-trifluoromethyl-2-pyridinamine"[tw] OR "3-Chloro-N-(3-chloro-2,6-dinitro-4-trifluoromethylphenyl)-5-trifluoromethyl-2-pyridylamine"[tw] OR "3-chloro-N-[3-chloro-2,6-dinitro-4-(trifluoromethyl)phenyl]-5-(trifluoromethyl)-2-pyridinamine"[tw] OR "3-chloro-N-[3-chloro-2,6-dinitro-4-(trifluoromethyl)phenyl]-5-(trifluoromethyl)pyridin-2-amine"[tw] OR "79622-59-6"[tw] OR "Altima"[tw] OR "Fluazinam"[tw] OR "Fluaziname"[tw] OR "Mapro"[tw] OR "Sekoya"[tw] OR "Shirlan"[tw] OR **"systhane" [Supplementary Concept]** OR "B6T1JTM6KZ"[rn] OR "88671-89-0"[rn] OR "(R)-2-p-chlorophenyl-2-(1H-1,2,4-triazol-1-ylmethyl)hexanenitrile"[tw] OR ".alpha.-Butyl-.alpha.-(4-chlorophenyl)-1H-1,2,4-triazole-1-propanenitrile"[tw] OR "2 (4 chlorophenyl) 2 (1h 1, 2, 4 triazol 1 ylmethyl) hexanenitrile"[tw] OR "2-(4-chlorophenyl)-2-(1,2,4-triazol-1-ylmethyl)hexanenitrile"[tw] OR "2-(4-Chlorophenyl)-2-(1H-1,2,4-triazol-1-ylmethyl)hexanenitrile"[tw] OR "2-p-Chlorophenyl-2-(1H-1,2,4-triazol-1-ylmethyl)hexanenitrile"[tw] OR "88671-89-0"[tw] OR "alpha butyl alpha (4 chlorophenyl) 1h 1, 2, 4 triazole 1 propanenitrile"[tw] OR "alpha-Butyl-alpha-(4-chlorophenyl)-1H-1,2,4-triazole-1-propanenitrile"[tw] OR "alpha-n-butyl-alpha(4-chlorophenyl)-1H-1,2,4-triazole-1-propanenitrile"[tw] OR "alpha-n-butyl-alpha-(4-chlorophenyl)-1H-1,2,4-triazole-1-propanenitrile"[tw] OR "B6T1JTM6KZ"[tw] OR "myclobutanil"[tw] OR "Synthane 12E"[tw] OR "Systhane"[tw] OR **"ochratoxin A" [Supplementary Concept]** OR "1779SX6LUY"[rn] OR "303-47-9"[rn] OR "1779SX6LUY"[tw] OR "303-47-9"[tw] OR "ochratoxin A"[tw] OR "ochratoxine a"[tw] OR **"spiroxamine" [Supplementary Concept]** OR "OUT5YHB7BO"[rn] OR "118134-30-8"[rn] OR "(8-tert-Butyl-1,4-dioxa-spiro[4.5]dec-2-ylmethyl)-ethyl-propyl-amine"[tw] OR "118134-30-8"[tw] OR "8-tert-butyl-1,4-dioxaspiro(4.5)decan-2-ylmethyl(ethyl)(propyl)amine"[tw] OR "OUT5YHB7BO"[tw] OR "Spiroxamine"[tw] OR **"thiacloprid" [Supplementary Concept]** OR "DSV3A944A4"[rn] OR "111988-49-9"[rn] OR "(3-((6-Chloro-3-pyridinyl)methyl)-2-thiazolidinylidene)cyanamide"[tw] OR "[3 (6 chloro 3 pyridinylmethyl) 2 thiazolidinylidene] cyanamide"[tw] OR "[3 [ (6 chloro 3 pyridinyl) methyl] 2 thiazolidinylidene] cyanamide"[tw] OR "[3 [ (6 chloropyridin 3 yl) methyl] 1, 3 thiazolidin 2 ylidene] cyanamide"[tw] OR "[3-[(6-chloranylpyridin-3-yl)methyl]-1,3-thiazolidin-2-ylidene]cyanamide"[tw] OR "[3-[(6-chloro-3-pyridinyl)methyl]-2-thiazolidinylidene]cyanamide"[tw] OR "[3-[(6-chloro-3-pyridyl)methyl]thiazolidin-2-ylidene]cyanamide"[tw] OR "[3-[(6-chloropyridin-3-yl)methyl]-1,3-thiazolidin-2-ylidene]cyanamide"[tw] OR "{3-[(6-chloropyridin-3-yl)methyl]-1,3-thiazolidin-2-ylidene}cyanamide"[tw] OR "111988-49-9"[tw] OR "3-((6-chloro-3-pyridinyl)methyl)-2-thiazolidinylidene cyanamide"[tw] OR "DSV3A944A4"[tw] OR "Thiacloprid"[tw] OR "thiaclopride"[tw] OR **"Thiram"[Mesh]** OR "0D771IS0FH"[rn] OR "137-26-8"[rn] OR "0D771IS0FH"[tw] OR "137-26-8"[tw] OR "16c tetramethylthiuram disulfide"[tw] OR "Aapirol"[tw] OR "Aatiram"[tw] OR "Accel TMT"[tw] OR "Aceto TETD"[tw] OR "Akrochem TMTD"[tw] OR "Anles"[tw] OR "Arasan"[tw] OR "Atiram"[tw] OR "Aules"[tw] OR "Basultra"[tw] OR "Betoxin"[tw] OR "bis (dimethyl thiocarbamoyl) disulfide"[tw] OR "bis (dimethylthiocarbamoyl) disulfide"[tw] OR "Bis(dimethyl thiocarbamoyl)disulfide"[tw] OR "Bis(dimethylthiocarbamoyl) disulfide"[tw] OR "Bis(dimethylthiocarbamoyl) disulphide"[tw] OR "Bis(dimethylthiocarbamyl) disulfide"[tw] OR "bis[Dimethylthiocarbamyl] disulfide"[tw] OR "Cunitex"[tw] OR "Cyuram DS"[tw] OR "Delsan"[tw] OR "Ekagom TB"[tw] OR "Falitiram"[tw] OR "Fermide"[tw] OR "Fernacol"[tw] OR "Fernasan"[tw] OR "Fernide"[tw] OR "Formalsol"[tw] OR "Granuflo"[tw] OR "Hermal"[tw] OR "Hermat TMT"[tw] OR "Heryl"[tw] OR "Hexathir"[tw] OR "Kregasan"[tw] OR "Mercuram"[tw] OR "Methyl thiuramdisulfide"[tw] OR "Methyl tuads"[tw] OR "Methylthiuram disulfide"[tw] OR "Metiur"[tw] OR "Metiurac"[tw] OR "Nobecutan"[tw] OR "Nocceler TT"[tw] OR "Nomersan"[tw] OR "Normersan"[tw] OR "NSC 1771"[tw] OR "NSC1771"[tw] OR "Panoram 75"[tw] OR "Polyram ultra"[tw] OR "Pomarsol"[tw] OR "Pomasol"[tw] OR "puralin"[tw] OR "Radothiram"[tw] OR "Rezifilm"[tw] OR "rhenogran"[tw] OR "Robac TMT"[tw] OR "Sadoplon"[tw] OR "Spotrete"[tw] OR "Sranan-sf-X"[tw] OR "Teramethylthiuram disulfide"[tw] OR "Tersan"[tw] OR "Tersantetramethyldiurane sulfide"[tw] OR "tetra methyl thiuram disulfide"[tw] OR "tetramethyl tetramethylthiuram disulfide"[tw] OR "tetramethyl thiuram disulfide"[tw] OR "Tetramethyl thiuramdisulfide"[tw] OR "Tetramethyl thiurane disulfide"[tw] OR "Tetramethyl thiurane disulphide"[tw] OR "Tetramethyldiurane sulphite"[tw] OR "Tetramethylenethiuram disulfide"[tw] OR "Tetramethylenethiuram disulphide"[tw] OR "Tetramethylthiocarbamoyldisulphide"[tw] OR "Tetramethylthioperoxydicarbonic diamide"[tw] OR "Tetramethylthioramdisulfide"[tw] OR "Tetramethylthiouram disulfide"[tw] OR "Tetramethylthiuram"[tw] OR "tetramethylthiuramdisulfide"[tw] OR "tetramethylthiuramidisulfide"[tw] OR "Tetramethylthiuran disulphide"[tw] OR "Tetramethylthiurane disulfide"[tw] OR "Tetramethylthiurum disulfide"[tw] OR "Tetramethylthiurum disulphide"[tw] OR "Tetrapom"[tw] OR "Tetrasipton"[tw] OR "tetrathion"[tw] OR "tetrathione"[tw] OR "tetrathionine"[tw] OR "Tetrathiuram disulfide"[tw] OR "Tetrathiuram disulphide"[tw] OR "Thianosan"[tw] OR "Thillate"[tw] OR "Thimar"[tw] OR "Thimer"[tw] OR "thiosan"[tw] OR "Thioscabin"[tw] OR "Thiotex"[tw] OR "Thiotox"[tw] OR "thiram"[tw] OR "Thiramad"[tw] OR "Thirampa"[tw] OR "Thiramum"[tw] OR "Thirasan"[tw] OR "Thiulin"[tw] OR "Thiulix"[tw] OR "Thiurad"[tw] OR "Thiuram"[tw] OR "Thiuramin"[tw] OR "thiuramyl"[tw] OR "Thylate"[tw] OR "Tiradin"[tw] OR "tiram"[tw] OR "tiramo"[tw] OR "Tirampa"[tw] OR "tiuramyl"[tw] OR "TMT Disulfide"[tw] OR "TMTD"[tw] OR "TMTDS"[tw] OR "Trametan"[tw] OR "Tridipam"[tw] OR "Tripomol"[tw] OR "tuad"[tw] OR "TUEX"[tw] OR "Tulisan"[tw] OR "Tutan"[tw] OR "Tyradin"[tw] OR "Vancide TM"[tw] OR "Vulcafor TMT"[tw] OR "Vulkacit TH"[tw] OR "Vulkazam S"[tw] OR **"triadimefon" [Supplementary Concept]** OR "43121-43-3"[rn] OR "1 (4 chlorophenoxy) 3, 3 dimethyl 1 (1, 2, 4 triazol 1 yl) 2 butanone"[tw] OR "1-(1,2,4-triazolyl)-1-(4-chlorophenoxy)-3,3-dimethylbutan-2-one"[tw] OR "1-(1,2,4-Triazoyl-1)-1-(4-chloro-phenoxy)-3,3-dimethylbutanone"[tw] OR "1-(4-chlorophenoxy)-3,3-dimethyl-1-(1,2,4-triazol-1-yl)butan-2-one"[tw] OR "1-(4-Chlorophenoxy)-3,3-dimethyl-1-(1,2,4-triazol-1-yl)-butan-2-one"[tw] OR "1-(4-Chlorophenoxy)-3,3-dimethyl-1-(1,2,4-triazol-1-yl)butanone"[tw] OR "1-(4-chlorophenoxy)-3,3-dimethyl-1-(1H-1,2,4-triazol-1-yl) butan-2-one"[tw] OR "1-(4-Chlorophenoxy)-3,3-dimethyl-1-(1H-1,2,4-triazol-1-yl)-2-butanone"[tw] OR "1-(4-chlorophenoxy)-3,3-dimethyl-1-(1H-1,2,4-triazol-1-yl)butan-2-one"[tw] OR "1-(4-Chlorophenoxy)-3,3-dimethyl-1-(1H-1,2,4-triazole -1-yl)-2-butanone"[tw] OR "1-(4-Chloro-phenoxy)-3,3-dimethyl-1-[1,2,4]triazol-1-yl-butan-2-one"[tw] OR "43121-43-3"[tw] OR "Acizol"[tw] OR "Adifon"[tw] OR "Amiral"[tw] OR "Azocene"[tw] OR "Bayleton"[tw] OR "Diametom B"[tw] OR "Fenxiunin"[tw] OR "Haleton"[tw] OR "Miltek"[tw] OR "Nurex"[tw] OR "Otria 25"[tw] OR "Rofon"[tw] OR "Tidifon"[tw] OR "triadimefon"[tw] OR "Triadimefone"[tw] OR "Triadimeform"[tw] OR "triadimenol"[tw] OR "Tripinacloraz"[tw] OR **"flusilazole" [Supplementary Concept]** OR "F3WG2VVD87"[rn] OR "85509-19-9"[rn] OR "85509-19-9"[tw] OR "Benocarp"[tw] OR "bis (4 fluorophenyl) methyl (1, 2, 4 triazol 1 yl) silane"[tw] OR "bis(4-fluorophenyl)(methyl)(1H-1,2,4-triazol-1-ylmethyl)silane"[tw] OR "bis(4-fluorophenyl)-methyl-(1,2,4-triazol-1-ylmethyl)silane"[tw] OR "Bis(4-fluorophenyl)methyl(1H-1,2,4-triazol-1-ylmethyl)silane"[tw] OR "DPX 6573"[tw] OR "dpx h6573"[tw] OR "DPX-H 6573"[tw] OR "dpxh6573"[tw] OR "DPX-N 6573"[tw] OR "DPX-N6573"[tw] OR "F3WG2VVD87"[tw] OR "Flusilazol"[tw] OR "Flusilazole"[tw] OR "Fluzilazol"[tw] OR "Nustar"[tw] OR "Olymp"[tw] OR "PPX-H6573"[tw] OR **"hexaconazole" [Supplementary Concept]** OR "SX9R3X1FQV"[rn] OR "79983-71-4"[rn] OR "2 (2, 4 dichlorophenyl) 1 (1h 1, 2, 4 triazol 1 yl) 2 hexanol"[tw] OR "2-(2,4-dichlorophenyl)-1-(1,2,4-triazol-1-yl)-2-hexanol"[tw] OR "2-(2,4-dichlorophenyl)-1-(1,2,4-triazol-1-yl)hexan-2-ol"[tw] OR "2-(2,4-dichlorophenyl)-1-(1H-1,2,4-triazol-1-yl)hexan-2-ol"[tw] OR "79983-71-4"[tw] OR "alpha butyl alpha (2, 4 dichlorophenyl) 1h 1, 2, 4 triazole 1 ethanol"[tw] OR "Canvil"[tw] OR "Chlortriafol"[tw] OR "Clortriafol"[tw] OR "Contaf"[tw] OR "Flowmax 5SC"[tw] OR "Hexaconazol"[tw] OR "Hexaconazole"[tw] OR "Ranvil"[tw] OR "SX9R3X1FQV"[tw] OR **"propiconazole" [Supplementary Concept]** OR "142KW8TBSR"[rn] OR "60207-90-1"[rn] OR "1 [2 (2, 4 dichlorophenyl) 4 propyl 1, 3 dioxolan 2 ylmethyl] 1h 1, 2, 4 triazole"[tw] OR "1-((2-(2,4-Dichlorophenyl)-4-propyl-1,3-dioxolan-2-yl)methyl)-1H-1,2,4-triazole"[tw] OR "1-(2-(2,4-Dichlorophenyl)-4-propyl-1,3-dioxolan-2-yl)methyl-1H-1,2,4-triazole"[tw] OR "1-(2-(2,4-Dichlorophenyl)-4-propyl-1,3-dioxolan-2-ylmethyl)-1H-1,2,4-triazole"[tw] OR "1-[[2-(2,4-dichlorophenyl)-4-propyl-1,3-dioxolan-2-yl]methyl]-1,2,4-triazole"[tw] OR "1-[[2-(2,4-Dichlorophenyl)-4-propyl-1,3-dioxolan-2-yl]methyl]-1H-1,2,4-triazole"[tw] OR "1-[2-(2,4-Dichloro-phenyl)-4-propyl-[1,3]dioxolan-2-ylmethyl]-1H-[1,2,4]triazole"[tw] OR "1-[2-(2,4-dichlorophenyl)-4-propyl-1,3-dioxolan-2-ylmethyl]-1H-1,2,4-triazole"[tw] OR "1-{[2-(2,4-dichlorophenyl)-4-propyl-1,3-dioxolan-2-yl]methyl}-1H-1,2,4-triazole"[tw] OR "142KW8TBSR"[tw] OR "60207-90-1"[tw] OR "Bamper"[tw] OR "cga 64250"[tw] OR "cga64250"[tw] OR "Desmel"[tw] OR "Proconazole"[tw] OR "Propiconazol"[tw] OR "Propiconazole"[tw] OR "Propyconazol"[tw] OR "Wocosen"[tw] OR "Wocosin 50TK"[tw] OR **"Endosulfan"[Mesh]** OR "OKA6A6ZD4K"[rn] OR "115-29-7"[rn] OR "115-29-7"[tw] OR "5, 6 bis (hydroxymethyl) 1, 2, 3, 4, 7, 7 hexachloronorbornene sulfite"[tw] OR "5, 6 bis (hydroxymethyl) 1, 2, 3, 4, 7, 7 hexachloronorbornenesulfite"[tw] OR "5, 6 bis (hydroxymethyl) hexachlorobicyclo [2.2.1] hept 2 ene sulfite"[tw] OR "5, 6 bis (hydroxymethyl) hexachloronorcamphene sulfite"[tw] OR "alpha endosulfane"[tw] OR "benzoepin"[tw] OR "beosit"[tw] OR "beta endosulfane"[tw] OR "chlorothiepine"[tw] OR "chlorthiapinum"[tw] OR "chlorthiepin"[tw] OR "Chlortiepin"[tw] OR "Crisulfan"[tw] OR "cyclodan"[tw] OR "devisulfan"[tw] OR "Devisulphan"[tw] OR "Endocel"[tw] OR "endogan"[tw] OR "Endosol"[tw] OR "endosulfan"[tw] OR "Endosulphan"[tw] OR "Endotaf"[tw] OR "ensawan"[tw] OR "FMC 5462"[tw] OR "fmc5462"[tw] OR "hexachlorohexahydro 6, 9 methano 2, 4, 3 benzodioxathiepine 3 oxide"[tw] OR "Hexachlorohexahydromethano 2,4,3-benzodioxathiepin-3-oxide"[tw] OR "hexachloronorbornene 5, 6 bis (oxymethylene) sulfite"[tw] OR "Hildan"[tw] OR "Insectophene"[tw] OR "malix"[tw] OR "OKA6A6ZD4K"[tw] OR "Rasayansulfan"[tw] OR "Sialan"[tw] OR "thifor"[tw] OR "thimul"[tw] OR "Thiodan"[tw] OR "Thiodon"[tw] OR "thiofor"[tw] OR "thiomul"[tw] OR "Thionate"[tw] OR "thionex"[tw] OR "Thiosulfan"[tw] OR "Thiotox"[tw] OR "thyodan"[tw] OR "thyonex"[tw] OR "tiodan"[tw] OR "tionel"[tw] OR "Tionex"[tw] OR "Tiovel"[tw] OR **"pyridaben" [Supplementary Concept]** OR "2E4JBA5272"[rn] OR "96489-71-3"[rn] OR "2E4JBA5272"[tw] OR "2-tert-Butyl-5-(4-tert-Butylbenzylthio)-4-chloropyridazin-3(2H)-one"[tw] OR "2-tert-Butyl-5-(4-tert-butyl-benzylthio)-4-chloropyridazin-3(2H)-one"[tw] OR "2-tert-butyl-5-[(4-tert-butylbenzyl)thio]-4-chloropyridazin-3(2H)-one"[tw] OR "2-tert-butyl-5-[(4-tert-butylbenzyl)thio]-4-chloro-pyridazin-3-one"[tw] OR "96489-71-3"[tw] OR "Damanlin"[tw] OR "Nexter"[tw] OR "Pyramite"[tw] OR "Pyridaben"[tw] OR "Sanmite"[tw] OR **"Methanol"[Mesh]** OR "67-56-1"[rn] OR "Y4S76JWI15"[rn] OR "67-56-1"[tw] OR "carbinol"[tw] OR "Carbonal"[tw] OR "hydroxymethan"[tw] OR "Hydroxymethane"[tw] OR "MeOH"[tw] OR "methanol"[tw] OR "Methoxy Group"[tw] OR "methyl alcohol"[tw] OR "Methyl hydrate"[tw] OR "Methyl hydroxide"[tw] OR "Methylalcohol"[tw] OR "Methylic alcohol"[tw] OR "Methylol"[tw] OR "MetOH"[tw] OR "Monohydroxymethane"[tw] OR "monomethylol"[tw] OR "Y4S76JWI15"[tw] OR "wood alcohol"[tw] OR "Wood naphtha"[tw] OR "wood spirit"[tw] OR **"cyproconazole" [Supplementary Concept]** OR "94361-06-5"[rn] OR "622B9C3E6T"[rn] OR "622B9C3E6T"[tw] OR "94361-06-5"[tw] OR "Atemi"[tw] OR "Cyproconazol"[tw] OR "Cyproconazole"[tw] OR **"triticonazole" [Supplementary Concept]** OR "131983-72-7"[rn] OR "(1RS)-(E)-5-((4-chlorophenyl)methylene)-2,2-dimethyl-1-(1H-1,2,4-triazol-1-ylmethyl)cyclopentan-1-ol"[tw] OR "131983-72-7"[tw] OR "Triticonazole"[tw] OR **"diniconazole" [Supplementary Concept]** OR "X82HVO1N83"[rn] OR "76714-16-4"[rn] OR "83657-24-3"[rn] OR "1-(2,4-dichlorophenyl)-4,4-dimethyl-2-(1,2,4-triazol-1-yl)-1-penten-3-ol"[tw] OR "1-(2,4-Dichlorophenyl)-4,4-dimethyl-2-(1H-1,2,4-triazol-1-yl)pent-1-en-3-ol"[tw] OR "76714-16-4"[tw] OR "83657-24-3"[tw] OR "Diclopentezol"[tw] OR "Diniconazole"[tw] OR "S 3308"[tw] OR "S-3308L"[tw] OR "X82HVO1N83"[tw] OR **"Zidovudine"[Mesh]** OR "4B9XT59T7S"[rn] OR "30516-87-1"[rn] OR "3' azido 2', 3' dideoxyribosylthymine"[tw] OR "3' azido 2', 3' dideoxythymidine"[tw] OR "3' Azido 2',3' Dideoxythymidine"[tw] OR "3' Azido 3' deoxythymidine"[tw] OR "3' azido 3' deoxythymidine"[tw] OR "3' azidothymidine"[tw] OR "30516-87-1"[tw] OR "3'azido-3'deoxythymidine"[tw] OR "3'-azido3'-deoxythymidine"[tw] OR "3-Azido-3-deoxythymidine"[tw] OR "3'-Azido-3'deoxythymidine"[tw] OR "4B9XT59T7S"[tw] OR "adovi"[tw] OR "avirzid"[tw] OR "azidodeoxythymidine"[tw] OR "azidomine"[tw] OR "Azidothymidine"[tw] OR "Azitidin"[tw] OR "AZT"[tw] OR "bio zt"[tw] OR "bw a 509 u"[tw] OR "BW A509U"[tw] OR "BW-A 509U"[tw] OR "BWA509U"[tw] OR "BWA-509U"[tw] OR "Dendrigen"[tw] OR "pranadox"[tw] OR "retrocar"[tw] OR "Retrovir"[tw] OR "Retrovis"[tw] OR "ZDV"[tw] OR "zidis"[tw] OR "zidovir"[tw] OR "zidovudin"[tw] OR "zidovudine"[tw] OR "Zidovudinum"[tw] OR "zudovidine"[tw] OR "zydowin"[tw] OR **"Metoclopramide"[Mesh]** OR "L4YEB44I46"[rn] OR "364-62-5"[rn] OR "364-62-5"[tw] OR "4 amino 5 chloro n (2 diethylaminoethyl) 2 methoxybenzamide"[tw] OR "4 amino 5 chloro n (2 diethylaminoethyl) o anisamide"[tw] OR "4 amino 5 chloro n (2 diethylaminoethyl) ortho anisamide"[tw] OR "4 Amino-5-chloro-N-(2-(diethylamino)ethyl)-2-methoxybenzamide"[tw] OR "4-Amino-5-chloro-N-(2-(diethylamino)ethyl)-o-anisamide"[tw] OR "4-Amino-5-chloro-N-(2-diethylamino-ethyl)-2-methoxy-benzamide"[tw] OR "4-amino-5-chloro-N-[2-(diethylamino)ethyl]-2-methoxybenzamide"[tw] OR "4-amino-5-chloro-N-[2-(diethylamino)ethyl]-2-methoxy-benzamide"[tw] OR "4-amino-5-chloro-N-[2-(diethylamino)-ethyl]-2-methoxybenzamide"[tw] OR "5 chloro 2 methoxyprocainamide"[tw] OR "ahr 3070 c"[tw] OR "ahr 3070c"[tw] OR "ahr3070c"[tw] OR "ametic"[tw] OR "anausin"[tw] OR "apo-metoclop"[tw] OR "aputern"[tw] OR "betaclopramide"[tw] OR "carnotprim primperan"[tw] OR "Cerucal"[tw] OR "clodilion"[tw] OR "clopamon"[tw] OR "clopan"[tw] OR "Clopra"[tw] OR "clopram"[tw] OR "Clopromate"[tw] OR "Degan"[tw] OR "del 1267"[tw] OR "del1267"[tw] OR "dibertil"[tw] OR "Duraclamid"[tw] OR "Elieten"[tw] OR "emenil"[tw] OR "emetal"[tw] OR "emetard"[tw] OR "Emetid"[tw] OR "Emitasol"[tw] OR "emperal"[tw] OR "encil"[tw] OR "enzimar"[tw] OR "Eucil"[tw] OR "gastro timelets"[tw] OR "gastrobi"[tw] OR "Gastrobid"[tw] OR "Gastromax"[tw] OR "Gastronerton"[tw] OR "gastrosil"[tw] OR "Gastrotablinen"[tw] OR "gastrotem"[tw] OR "gastrotimelets"[tw] OR "gavistal"[tw] OR "gensil"[tw] OR "Gimoli"[tw] OR "hemesis"[tw] OR "hyrin"[tw] OR "imperan"[tw] OR "L4YEB44I46"[tw] OR "m 813"[tw] OR "m813"[tw] OR "maril"[tw] OR "Maxeran"[tw] OR "maxeron"[tw] OR "maxolan"[tw] OR "Maxolon"[tw] OR "mcp-beta tropfen"[tw] OR "meclomid"[tw] OR "meclopamide"[tw] OR "meclopramide"[tw] OR "Meclopran"[tw] OR "Megaldrate"[tw] OR "meramide"[tw] OR "Metaclopramide"[tw] OR "Metadrate"[tw] OR "metagliz"[tw] OR "metamide"[tw] OR "Methochlopramide"[tw] OR "methoclopramide"[tw] OR "methoclopramine"[tw] OR "Methoxychlorprocainamide"[tw] OR "metlazel"[tw] OR "Metochlopramide"[tw] OR "Metochloropramide"[tw] OR "Metoclol"[tw] OR "metoclopamide"[tw] OR "metoclopramid"[tw] OR "metoclopramide"[tw] OR "Metoclopramidum"[tw] OR "metoclopramine"[tw] OR "metoclopranide hydrochloride"[tw] OR "metoclor"[tw] OR "metoclorpramide"[tw] OR "metocobil"[tw] OR "metocyl"[tw] OR "metodopramide"[tw] OR "metolon"[tw] OR "metopram"[tw] OR "metox"[tw] OR "metozolv"[tw] OR "metpamid"[tw] OR "metram"[tw] OR "Metramid"[tw] OR "Moriperan"[tw] OR "mygdalon"[tw] OR "nausil"[tw] OR "neopramiel"[tw] OR "netaf"[tw] OR "nilatika"[tw] OR "normastin"[tw] OR "Octamide"[tw] OR "opram"[tw] OR "Parmid"[tw] OR "Paspertin"[tw] OR "Peraprin"[tw] OR "perinorm"[tw] OR "pharmyork"[tw] OR "Plasil"[tw] OR "pramidin"[tw] OR "Pramiel"[tw] OR "pramin"[tw] OR "pramotel"[tw] OR "Primperan"[tw] OR "primperil"[tw] OR "prinparl"[tw] OR "prokinyl lp"[tw] OR "prowel"[tw] OR "pulin"[tw] OR "Pylomid"[tw] OR "Reclomide"[tw] OR "Regla"[tw] OR "Reglan"[tw] OR "Reliveran"[tw] OR "rimetin"[tw] OR "sensamide"[tw] OR "sotatic-10"[tw] OR "Terperan"[tw] OR "tomid"[tw] OR "vertivom"[tw] OR "vomitrol"[tw] OR "zumatrol"[tw] OR **"Acebutolol"[Mesh]** OR "67P356D8GH"[rn] OR "37517-30-9"[rn] OR "1 (2 acetyl 4 butyramidophenoxy) 2 hydroxy 3 isopropylaminopropane"[tw] OR "1-(2-Acetyl-4-n-butyramidophenoxy)-2-hydroxy-3-isopropylaminopropane"[tw] OR "37517-30-9"[tw] OR "67P356D8GH"[tw] OR "Acebrutololum"[tw] OR "acebutolol"[tw] OR "Acebutololo"[tw] OR "Acebutololum"[tw] OR "acecor"[tw] OR "ApoAcebutolol"[tw] OR "bay c 7705"[tw] OR "bay c7705"[tw] OR "diasectral"[tw] OR "espesil"[tw] OR "flebutol"[tw] OR "grifobutol"[tw] OR "il 17803a"[tw] OR "il17803a"[tw] OR "M & B 17803A"[tw] OR "m and b 17803 a"[tw] OR "M and B 17803A"[tw] OR "M and B17803 A"[tw] OR "M&B-17803 A"[tw] OR "Monitan"[tw] OR "n [3 acetyl 4 [2 hydroxy 3 [ (methylethyl) amino] propoxy] phenyl] butanamide"[tw] OR "N-[3-acetyl-4-[2-hydroxy-3-[(1-methylethyl)amino]propoxy]phenyl]butanamide"[tw] OR "Neptal"[tw] OR "neptall"[tw] OR "NovoAcebutolol"[tw] OR "Prent"[tw] OR "Rhotral"[tw] OR "Sectral"[tw] OR "Wesfalin"[tw] OR **"Isoniazid"[Mesh]** OR "V83O1VOZ8L"[rn] OR "54-85-3"[rn] OR "4 pyridinecarbohydrazide"[tw] OR "4 pyridinecarboxylic acid hydrazide"[tw] OR "54-85-3"[tw] OR "Abdizide"[tw] OR "Andrazide"[tw] OR "Anidrasona"[tw] OR "antimic"[tw] OR "Antimicina"[tw] OR "antimicine"[tw] OR "Antituberkulosum"[tw] OR "apacizina (aminosalicylate)"[tw] OR "Armacide"[tw] OR "Armazid"[tw] OR "Armazide"[tw] OR "atcotibin"[tw] OR "Atcotibine"[tw] OR "Azuren"[tw] OR "bacillen"[tw] OR "Bacillin"[tw] OR "Cedin"[tw] OR "Cemidon"[tw] OR "Chemiazid"[tw] OR "Chemidon"[tw] OR "Continazine"[tw] OR "Cortinazine"[tw] OR "Cotinazin"[tw] OR "cotinazine"[tw] OR "Cotinizin"[tw] OR "cotinizine"[tw] OR "curazid forte"[tw] OR "Defonin"[tw] OR "dianicotyl"[tw] OR "diazid"[tw] OR "Dibutin"[tw] OR "Diforin"[tw] OR "Dinacrin"[tw] OR "Ditubin"[tw] OR "Ebidene"[tw] OR "Eralon"[tw] OR "eralone"[tw] OR "Ertuban"[tw] OR "Eutizon"[tw] OR "eutizone"[tw] OR "Evalon"[tw] OR "Fetefu"[tw] OR "Fimalene"[tw] OR "fimaline"[tw] OR "FRS-3"[tw] OR "fsr3"[tw] OR "Ftivazide"[tw] OR "GINK"[tw] OR "hain"[tw] OR "hiconyl"[tw] OR "Hid rasonil"[tw] OR "hidraciber"[tw] OR "Hidranizil"[tw] OR "Hidrasonil"[tw] OR "hidrazida"[tw] OR "Hidrulta"[tw] OR "hidrulte"[tw] OR "Hidrun"[tw] OR "Hycozid"[tw] OR "hycozide"[tw] OR "hydrasonil"[tw] OR "Hydrazid"[tw] OR "Hydrazide"[tw] OR "hydrazin"[tw] OR "Hyozid"[tw] OR "Hyzyd"[tw] OR "ido tebin"[tw] OR "Idrazil"[tw] OR "Inah"[tw] OR "inh burgthal"[tw] OR "INHd20"[tw] OR "Inizid"[tw] OR "inizide"[tw] OR "Iscotin"[tw] OR "iscotine"[tw] OR "Isidrina"[tw] OR "isidrine"[tw] OR "Ismazide"[tw] OR "Isobicina"[tw] OR "isobicine"[tw] OR "Isocid"[tw] OR "Isocidene"[tw] OR "isoco tin"[tw] OR "Isocotin"[tw] OR "isocotine"[tw] OR "Isohydrazide"[tw] OR "Isokin"[tw] OR "Isolyn"[tw] OR "isolyne"[tw] OR "isomazide"[tw] OR "isomerina"[tw] OR "Isonerit"[tw] OR "Isonex"[tw] OR "isoniac"[tw] OR "Isoniacid"[tw] OR "isoniazid"[tw] OR "Isoniazide"[tw] OR "isoniazidine"[tw] OR "Isoniazidum"[tw] OR "isoniazone"[tw] OR "isonicazid"[tw] OR "Isonicazide"[tw] OR "Isonicid"[tw] OR "isonicide"[tw] OR "Isonico"[tw] OR "Isonicotan"[tw] OR "isonicotane"[tw] OR "isonicotic acid hydrazide"[tw] OR "Isonicotil"[tw] OR "isonicotinate hydrazide"[tw] OR "Isonicotinhydrazid"[tw] OR "isonicotinhydrazide"[tw] OR "Isonicotinic acid hydrazide"[tw] OR "isonicotinic acid hydrazine"[tw] OR "isonicotinic acid hydrazone"[tw] OR "Isonicotinic Acid Vanillylidenehydrazide"[tw] OR "Isonicotinic hydrazide"[tw] OR "isonicotinicacid hydrazide"[tw] OR "Isonicotinohydrazide"[tw] OR "Isonicotinoyl hydrazide"[tw] OR "isonicotinoylhydrazide"[tw] OR "Isonicotinoylhydrazine"[tw] OR "Isonicotinyl hydrazide"[tw] OR "Isonicotinyl hydrazine"[tw] OR "Isonicotinylhydrazide"[tw] OR "Isonicotinylhydrazine"[tw] OR "Isonide"[tw] OR "Isonidrin"[tw] OR "isonidrine"[tw] OR "Isonikazid"[tw] OR "isonikazide"[tw] OR "Isonilex"[tw] OR "isonilyd"[tw] OR "Isonin"[tw] OR "Isonindon"[tw] OR "isonindone"[tw] OR "isonine"[tw] OR "Isonirit"[tw] OR "isonisin"[tw] OR "isonisine"[tw] OR "Isoniton"[tw] OR "isonitone"[tw] OR "isonivit"[tw] OR "Isonizida"[tw] OR "Isonizide"[tw] OR "isopharmide"[tw] OR "Isotamine"[tw] OR "Isotebe"[tw] OR "Isotebezid"[tw] OR "isotebezide"[tw] OR "isothiavit"[tw] OR "Isotinyl"[tw] OR "Isozid"[tw] OR "Isozide"[tw] OR "isozin"[tw] OR "isozine"[tw] OR "isozone"[tw] OR "Isozyd"[tw] OR "isozyde"[tw] OR "izoniazid"[tw] OR "L 1945"[tw] OR "Laniazid"[tw] OR "Laniozid"[tw] OR "LANIZID"[tw] OR "Mayambutol"[tw] OR "micosan"[tw] OR "micosane"[tw] OR "milazide"[tw] OR "Mybasan"[tw] OR "mybasane"[tw] OR "Neoteben"[tw] OR "neotebene"[tw] OR "Neo-Tizide"[tw] OR "Neoxin"[tw] OR "neoxine"[tw] OR "neoxon"[tw] OR "neoxone"[tw] OR "Neumandin"[tw] OR "Nevin"[tw] OR "Niadrin"[tw] OR "nicatibine"[tw] OR "nicazid"[tw] OR "Nicazide"[tw] OR "Nicetal"[tw] OR "Nicizina"[tw] OR "nicodrin"[tw] OR "Niconyl"[tw] OR "nicosciorin"[tw] OR "nicotibin"[tw] OR "Nicotibina"[tw] OR "Nicotibine"[tw] OR "Nicotisan"[tw] OR "nicotubin"[tw] OR "nicotubine"[tw] OR "nicozid"[tw] OR "Nicozide"[tw] OR "nicozyd"[tw] OR "Nidaton"[tw] OR "Nidrazid"[tw] OR "nidrazide"[tw] OR "Nikozid"[tw] OR "nikozide"[tw] OR "niosciorine"[tw] OR "Niplen"[tw] OR "Nitadon"[tw] OR "Niteban"[tw] OR "Nitebannsc 9659"[tw] OR "nortibina"[tw] OR "nortibine"[tw] OR "Nydrazid"[tw] OR "nydrazide"[tw] OR "Nyscozid"[tw] OR "nyscozide"[tw] OR "Pelazid"[tw] OR "pelazide"[tw] OR "Percin"[tw] OR "Phthisen"[tw] OR "Phthivazid"[tw] OR "Phthivazide"[tw] OR "puran"[tw] OR "Pycazide"[tw] OR "Pyreazid"[tw] OR "pyreazide"[tw] OR "Pyricidin"[tw] OR "pyricidine"[tw] OR "Pyridicin"[tw] OR "pyridine 4 carbohydrazide"[tw] OR "pyridine 4 carbonic acid hydrazide"[tw] OR "pyridine 4 carboxyhydrazide"[tw] OR "pyridine-4-carboxylic acid hydrazide"[tw] OR "Pyrizidin"[tw] OR "pyrizidine"[tw] OR "ramnanon"[tw] OR "Raumanon"[tw] OR "Razide"[tw] OR "Retozide"[tw] OR "rhymicid"[tw] OR "Rifater"[tw] OR "Rimicid"[tw] OR "rimicide"[tw] OR "Rimifon"[tw] OR "Rimiphone"[tw] OR "Rimitsid"[tw] OR "Robiselin"[tw] OR "Robisellin"[tw] OR "robiselline"[tw] OR "Roxifen"[tw] OR "roxyfen miquel"[tw] OR "RP 5015"[tw] OR "Sanohidrazina"[tw] OR "sanohydrazina"[tw] OR "sanohydrazine"[tw] OR "santerazid"[tw] OR "santerazide"[tw] OR "Sauterazid"[tw] OR "Sauterzid"[tw] OR "Stanozide"[tw] OR "supercidin"[tw] OR "tb phlogin"[tw] OR "Tebecid"[tw] OR "tebecide"[tw] OR "tebecin"[tw] OR "tebecine"[tw] OR "tebemid"[tw] OR "Tebenic"[tw] OR "tebesium"[tw] OR "tebetracin"[tw] OR "tebetracine"[tw] OR "Tebexin"[tw] OR "tebexine"[tw] OR "Tebilon"[tw] OR "tebilone"[tw] OR "Tebos"[tw] OR "Teebaconin"[tw] OR "teebaconine"[tw] OR "Tekazin"[tw] OR "tekazine"[tw] OR "thiocevit"[tw] OR "Tibazide"[tw] OR "Tibemid"[tw] OR "tibemide"[tw] OR "Tibiazide"[tw] OR "Tibinide"[tw] OR "Tibison"[tw] OR "tibisone"[tw] OR "tibitan"[tw] OR "tibitane"[tw] OR "Tibivis"[tw] OR "Tibizide"[tw] OR "Tibusan"[tw] OR "tibusane"[tw] OR "Tisin"[tw] OR "Tisiodrazida"[tw] OR "tisiodrazide"[tw] OR "tisiotrazida"[tw] OR "Tizide"[tw] OR "Tubazid"[tw] OR "Tubazide"[tw] OR "Tubeco"[tw] OR "Tubecotubercid"[tw] OR "tubercid"[tw] OR "Tuberian"[tw] OR "tuberiane"[tw] OR "Tubicon"[tw] OR "tubicone"[tw] OR "Tubilysin"[tw] OR "Tubizid"[tw] OR "Tubomel"[tw] OR "tubonil"[tw] OR "tubylisin"[tw] OR "tubylisine"[tw] OR "tyrid"[tw] OR "Tyvid"[tw] OR "tyvide"[tw] OR "Unicocyde"[tw] OR "Unicozyde"[tw] OR "V83O1VOZ8L"[tw] OR "valifol"[tw] OR "Vazadrine"[tw] OR "Vederon"[tw] OR "vederone"[tw] OR "vitazide"[tw] OR "yuhan-zid"[tw] OR "Zidafimia"[tw] OR "Zinadon"[tw] OR "zinadone"[tw] OR "Zonazide"[tw] OR **"Saccharin"[Mesh]** OR "FST467XS7D"[rn] OR "81-07-2"[rn] OR "128-44-9"[rn] OR "1, 2 benzisothiazol 3 (2h) one 1, 1 dioxide"[tw] OR "1, 2 benzisothiazol 3 one 1, 1 dioxide"[tw] OR "1, 2 dihydro 2 ketobenzisosulfonazole"[tw] OR "1, 2-Benzisothiazol-3(2H)-one, 1,1-dioxide"[tw] OR "1,2-Benzisothiazol-3(2H)-one 1,1-dioxide"[tw] OR "1,2-Benzisothiazol-3(2H)-one, 1,1-dioxide"[tw] OR "1,2-Dihydro-2-ketobenzisosulfonazole"[tw] OR "1,2-Dihydro-2-ketobenzisosulphonazole"[tw] OR "128-44-9"[tw] OR "2, 3 dihydro 3 oxobenzisosulfonazole"[tw] OR "2,3-Dihydro-3-oxobenzisosulfonazole"[tw] OR "2,3-Dihydro-3-oxo-Benzisosulfonazole"[tw] OR "2,3-Dihydro-3-oxobenzisosulphonazole"[tw] OR "2-Sulfobenzoic acid imide"[tw] OR "2-Sulfobenzoic imide"[tw] OR "2-Sulfobenzoicimide"[tw] OR "2-Sulphobenzoic imide"[tw] OR "81-07-2"[tw] OR "Anhydro-o-sulfaminebenzoic acid"[tw] OR "Benzo-2-sulfiide"[tw] OR "Benzo-2-sulphimide"[tw] OR "Benzoic acid sulfimide"[tw] OR "Benzoic sulfimide"[tw] OR "Benzoic sulphimide"[tw] OR "Benzosulfimide"[tw] OR "Benzosulfinide"[tw] OR "Benzosulphimide"[tw] OR "Benzo-sulphinide"[tw] OR "Benzoylsulfonic Imide"[tw] OR "Cristallose"[tw] OR "Crystallose"[tw] OR "FST467XS7D"[tw] OR "Garantose"[tw] OR "Glucid"[tw] OR "glucide"[tw] OR "Gluside"[tw] OR "Glycophenol"[tw] OR "Hermesetas"[tw] OR "Kandiset"[tw] OR "Kristallose"[tw] OR "Madhurin"[tw] OR "Natreen"[tw] OR "Neosaccharin"[tw] OR "o-Benzoic acid sulfimide"[tw] OR "o-Benzoic sulfimide"[tw] OR "o-Benzoic sulphimide"[tw] OR "o-Benzosulfimide"[tw] OR "o-Benzosulphimide"[tw] OR "o-Benzoyl sulfimide"[tw] OR "o-Benzoyl sulphimide"[tw] OR "O-Benzoylsulfimide"[tw] OR "ortho sulfobenzimide"[tw] OR "ortho sulfobenzoic acid imide"[tw] OR "Ortho sulphobenzamide"[tw] OR "o-Sulfobenzimide"[tw] OR "o-Sulfobenzoic acid imide"[tw] OR "O-Sulfobenzoic imide"[tw] OR "o-Sulfonbenzoic acid imide sodium salt"[tw] OR "Sacarina"[tw] OR "Saccharimide"[tw] OR "saccharin"[tw] OR "Saccharina"[tw] OR "Saccharine"[tw] OR "Saccharinol"[tw] OR "Saccharinose"[tw] OR "saccharoid"[tw] OR "Saccharol"[tw] OR "Saxin"[tw] OR "Sodium o-benzosulfimide"[tw] OR "Sodium saccharide"[tw] OR "Sucrette"[tw] OR "Sucromat"[tw] OR "sweet n low"[tw] OR "Sweeta"[tw] OR "sweetex"[tw] OR "sweetnin"[tw] OR "Sykose"[tw] OR "Syncal"[tw] OR "Willosetten"[tw] OR "Zaharina"[tw] OR **"Penicillin G"[Mesh:NoExp]** OR "YS5LY7JF4N"[rn] OR "69-57-8"[rn] OR "1406-05-9"[rn] OR "1406-05-9"[tw] OR "6 (phenylacetamido) penicillanate sodium"[tw] OR "6 phenylacetamidopenicillanate sodium"[tw] OR "69-57-8"[tw] OR "American penicillin"[tw] OR "Benpen"[tw] OR "Benzylpenicillin sodium"[tw] OR "benzylpenicillinate sodium"[tw] OR "Benzylpenicillinic acid sodium salt"[tw] OR "bupenna sodium"[tw] OR "Coliriocilina"[tw] OR "Crystapen"[tw] OR "Kesso-Pen"[tw] OR "Mycofarm"[tw] OR "Novocillin"[tw] OR "Parcillin"[tw] OR "Pekamin"[tw] OR "pen a brasive"[tw] OR "Pengesod"[tw] OR "Penibiot"[tw] OR "Penicilina G Llorente"[tw] OR "Penicillin G"[tw] OR "Penicillin Grünenthal"[tw] OR "penicillin ii sodium"[tw] OR "penicillin sodium"[tw] OR "penicilline g sodium"[tw] OR "PenicillinGsodiumsalt"[tw] OR "Penilaryn"[tw] OR "Penilevel"[tw] OR "Peniroger"[tw] OR "Pfizerpen"[tw] OR "Sodiopen"[tw] OR "Sodipen"[tw] OR "Sodium 6-(phenylacetamido)penicillanate"[tw] OR "sodium benzyl penicillin"[tw] OR "Sodium benzylpenicillin"[tw] OR "Sodium benzylpenicillinate"[tw] OR "Sodium penicillin"[tw] OR "sodium penicilline g"[tw] OR "Sugracillin sodium salt"[tw] OR "Unicilina"[tw] OR "YS5LY7JF4N"[tw] OR "Ursopen"[tw] OR "Veticillin"[tw] OR **"Thalidomide"[Mesh]** OR "4Z8R6ORS6L"[rn] OR "50-35-1"[rn] OR ".alpha.-(N-Phthalimido)glutarimide"[tw] OR ".alpha.-N-Phthalylglutaramide"[tw] OR ".alpha.-Phthalimidoglutarimide"[tw] OR "3-Phthalimidoglutarimide"[tw] OR "4Z8R6ORS6L"[tw] OR "50-35-1"[tw] OR "Algosediv"[tw] OR "alpha (n phthalimido) glutarimide"[tw] OR "alpha-(N-Phthalimido)glutarimide"[tw] OR "alpha-N-Phthalylglutaramide"[tw] OR "alpha-Phthalimidoglutarimide"[tw] OR "Asidon 3"[tw] OR "Asmadion"[tw] OR "Asmaval"[tw] OR "Bonbrain"[tw] OR "Calmore"[tw] OR "Calmorex"[tw] OR "Celgene"[tw] OR "Contergan"[tw] OR "Corronarobetin"[tw] OR "Distaval"[tw] OR "Distaxal"[tw] OR "Distoval"[tw] OR "Ectiluran"[tw] OR "Enterosediv"[tw] OR "Gastrinide"[tw] OR "Glupan"[tw] OR "Glutanon"[tw] OR "Grippex"[tw] OR "Hippuzon"[tw] OR "Imidene"[tw] OR "Isomin"[tw] OR "Kedavon"[tw] OR "Kevadon"[tw] OR "n (2, 6 dioxopiperid 3 yl) phthalimide"[tw] OR "N-(2,6-dioxo-3-piperidinyl)phthalimide"[tw] OR "N-(2,6-Dioxo-3-piperidyl)phthalimide"[tw] OR "n-(2,6-dioxopiperidin-3-yl)phthalimide"[tw] OR "Neaufatin"[tw] OR "Neosedyn"[tw] OR "Neosydyn"[tw] OR "Nerosedyn"[tw] OR "Neufatin"[tw] OR "Neurodyn"[tw] OR "Neurosedin"[tw] OR "Neurosedym"[tw] OR "Neurosedyn"[tw] OR "neurosedyne"[tw] OR "Nevrodyn"[tw] OR "Nibrol"[tw] OR "Noctosediv"[tw] OR "Noxodyn"[tw] OR "N-Phthalimidoglutamic acid imide"[tw] OR "N-Phthaloylglutamimide"[tw] OR "N-Phthalylglutamic acid imide"[tw] OR "nsc 66847"[tw] OR "NSC66847"[tw] OR "Pangul"[tw] OR "Pantosediv"[tw] OR "Pharmion"[tw] OR "Polygripan"[tw] OR "Pro-Bam M"[tw] OR "Pro-ban M"[tw] OR "Profarmil"[tw] OR "Quetimid"[tw] OR "Quietoplex"[tw] OR "Sandormin"[tw] OR "Sedalis"[tw] OR "Sedimide"[tw] OR "Sedin"[tw] OR "Sedisperil"[tw] OR "Sedoval"[tw] OR "shin naito"[tw] OR "Shinnibrol"[tw] OR "Sleepan"[tw] OR "Slipro"[tw] OR "Softenil"[tw] OR "Softenon"[tw] OR "Synovir"[tw] OR "Talargan"[tw] OR "Talidomide"[tw] OR "Talimol"[tw] OR "Talinol"[tw] OR "Talismol"[tw] OR "Talizer"[tw] OR "Telagan"[tw] OR "Telargan"[tw] OR "Telargean"[tw] OR "Tensival"[tw] OR "thado"[tw] OR "Thaled"[tw] OR "thalidomid"[tw] OR "thalidomide"[tw] OR "Thalidomidum"[tw] OR "Thalin"[tw] OR "Thalinette"[tw] OR "thalix"[tw] OR "Thalomid"[tw] OR "Thalomide"[tw] OR "Theophilcholine"[tw] OR "Valgis"[tw] OR "Valgraine"[tw] OR "Yodomin"[tw] OR **"Doxylamine"[Mesh]** OR "95QB77JKPL"[rn] OR "469-21-6"[rn] OR "562-10-7"[rn] OR ".alpha.-Dimethylaminoethoxyphenylmethyl-2-picoline"[tw] OR "2-Dimethylaminoethoxyphenylmethyl-2-picoline"[tw] OR "469-21-6"[tw] OR "562-10-7"[tw] OR "95QB77JKPL"[tw] OR "alsadorm"[tw] OR "decapryn"[tw] OR "deoxylamine succinate"[tw] OR "Diclectin"[tw] OR "Dolased"[tw] OR "donormyl"[tw] OR "dormidina"[tw] OR "Dossilamina"[tw] OR "doxilamina"[tw] OR "doxylamine"[tw] OR "doxylaminesuccinate"[tw] OR "doxylaminium succinate"[tw] OR "doxylaminosuccinate"[tw] OR "Doxylaminum"[tw] OR "doxy-sleep-aid"[tw] OR "Dozile"[tw] OR "Evanorm"[tw] OR "gittalun"[tw] OR "histadoxylamine"[tw] OR "hoggar"[tw] OR "mereprine"[tw] OR "Mersyndol"[tw] OR "Restavit"[tw] OR "sedaplus"[tw] OR "Somnil"[tw] OR "Syndol"[tw] OR "Unisom"[tw] OR "vicks nyquil"[tw] OR **"Diphenhydramine"[Mesh:NoExp]** OR "8GTS82S83M"[rn] OR "58-73-1"[rn] OR "147-24-0"[rn] OR ".beta.-(Dimethylamino)ethyl benzhydryl ether"[tw] OR ".beta.-Dimethylamino-aethyl-benzhydryl-aether"[tw] OR "147-24-0"[tw] OR "2 (benzhydroloxy) n, n dimethylethylamine"[tw] OR "2 (diphenylmethoxy) n, n dimethyl ethylamine"[tw] OR "2 (diphenylmethoxy) n, n dimethylethylamine"[tw] OR "2 benzhydryloxy n, n dimethylethylamine"[tw] OR "2 diphenylmethoxy n, n dimethylethylamine"[tw] OR "2-(Benzhydryloxy)-N,N-dimethylethanamine"[tw] OR "2-(Benzhydryloxy)-N,N-dimethylethylamine"[tw] OR "2-(Benzohydryloxy)-N,N-dimethylethylamine"[tw] OR "2-(diphenylmethoxy)-N,N-dimethylethanamine"[tw] OR "2-(Diphenylmethoxy)-N,N-dimethylethylamine"[tw] OR "2-(diphenylmethyl)oxy-N,N-dimethylethanamine"[tw] OR "2-(diphenylmethyl)oxy-N,N-dimethyl-ethanamine"[tw] OR "2-[(diphenylmethyl)oxy]-N,N-dimethylethanamine"[tw] OR "2-[di(phenyl)methoxy]-N,N-dimethylethanamine"[tw] OR "2-benzhydryloxy-N,N-dimethylethanamine"[tw] OR "2-benzhydryloxy-N,N-dimethyl-ethanamine"[tw] OR "2-diphenylmethoxy-N,N-demthylethanamine"[tw] OR "2-Diphenylmethoxy-N,N-dimethylethylamine"[tw] OR "58-73-1"[tw] OR "8GTS82S83M"[tw] OR "Aleryl"[tw] OR "Alledryl"[tw] OR "Allerdryl"[tw] OR "Allergan"[tw] OR "Allergeval"[tw] OR "Allergical"[tw] OR "Allergina"[tw] OR "Allergival"[tw] OR "Amidryl"[tw] OR "Antistominum"[tw] OR "Antomin"[tw] OR "Automin"[tw] OR "Bagodryl"[tw] OR "banaril"[tw] OR "Banophen"[tw] OR "Baramine"[tw] OR "Beldin"[tw] OR "Belix"[tw] OR "Benachlor"[tw] OR "benadril"[tw] OR "Benadrin"[tw] OR "Benadryl"[tw] OR "benadyl"[tw] OR "Ben-allergin"[tw] OR "Benapon"[tw] OR "Benhydramin"[tw] OR "benocten"[tw] OR "Benodin"[tw] OR "Benodine"[tw] OR "Benylan"[tw] OR "Benylin"[tw] OR "Benzantine"[tw] OR "Benzhydramine"[tw] OR "Benzhydraminum"[tw] OR "Benzhydroamina"[tw] OR "beta dimethylaminoethyl benzhydryl ether"[tw] OR "beta-Dimethylaminoethanol diphenylmethyl ether"[tw] OR "beta-Dimethylaminoethylbenzhydrylether"[tw] OR "Betramin"[tw] OR "caladryl"[tw] OR "carphenamine"[tw] OR "carphenex"[tw] OR "cathejell"[tw] OR "Compoz"[tw] OR "Dabylen"[tw] OR "Debendrin"[tw] OR "Dermistina"[tw] OR "Dermodrin"[tw] OR "Desentol"[tw] OR "Diabenyl"[tw] OR "Diabylen"[tw] OR "dibadorm n"[tw] OR "Dibendrin"[tw] OR "Dibenil"[tw] OR "Dibondrin"[tw] OR "dibrondrin"[tw] OR "Difedryl"[tw] OR "Difenhydramin"[tw] OR "Difenhydramine"[tw] OR "Dihidral"[tw] OR "Dimedrol"[tw] OR "Dimedryl"[tw] OR "Dimehydrinate"[tw] OR "Dimethylamine benzhydryl ester"[tw] OR "dimidril"[tw] OR "dimiril"[tw] OR "Diphantine"[tw] OR "diphedryl"[tw] OR "Diphen"[tw] OR "diphenacen"[tw] OR "diphendramine"[tw] OR "Diphenhist"[tw] OR "diphenhydramide"[tw] OR "diphenhydramin"[tw] OR "diphenhydramine"[tw] OR "Diphenhydraminum"[tw] OR "diphenydramine"[tw] OR "Diphenylhydramin"[tw] OR "Diphenylhydramine"[tw] OR "Dobacen"[tw] OR "Dormarex 2"[tw] OR "Dormin"[tw] OR "dryhistan"[tw] OR "Dryistan"[tw] OR "Drylistan"[tw] OR "Dylamon"[tw] OR "dytan"[tw] OR "emesan"[tw] OR "Etanautine"[tw] OR "Genahist"[tw] OR "Histacyl"[tw] OR "Histaxin"[tw] OR "histergan"[tw] OR "Hyadrine"[tw] OR "Hydramine"[tw] OR "Hyrexin"[tw] OR "Ibiodral"[tw] OR "Medidryl"[tw] OR "Mephadryl"[tw] OR "n, n dimethyl 2 (diphenylmethoxy) ethylamine"[tw] OR "N,N-Dimethyl-2-(diphenylmethoxy)-ethylamine hydrochloride"[tw] OR "N,N-Dimethyl-2-diphenylmethyloxyethylamine"[tw] OR "Nausen"[tw] OR "neosynodorm"[tw] OR "Novamina"[tw] OR "nytol"[tw] OR "o benzhydryldimethylaminoethanol"[tw] OR "O-Benzhydryl(dimethylamino)ethanol"[tw] OR "Probedryl"[tw] OR "reisegold"[tw] OR "resmin"[tw] OR "restamin"[tw] OR "Rigidil"[tw] OR "Rigidyl"[tw] OR "sediat"[tw] OR "sedryl"[tw] OR "Siladryl"[tw] OR "Silphen"[tw] OR "sleepeze"[tw] OR "Sleep-Eze D"[tw] OR "sominex"[tw] OR "Syntedril"[tw] OR "Syntodril"[tw] OR "trux-adryl"[tw] OR "tzoali"[tw] OR "unisom sleepgels"[tw] OR "valdrene"[tw] OR "venasmin"[tw] OR "vertirosan"[tw] OR "vicks formula 44"[tw] OR "vilbin"[tw] OR "wehdryl"[tw] OR "ziradryl"[tw] OR **"clopyralid" [Supplementary Concept]** OR "10G14M0WDH"[rn] OR "1702-17-6"[rn] OR "10G14M0WDH"[tw] OR "1702-17-6"[tw] OR "3,6-Dichloropicolinic acid"[tw] OR "Benzalox"[tw] OR "Cirtoxin"[tw] OR "Cliophar"[tw] OR "Clopiralid"[tw] OR "Clopyralid"[tw] OR "Clopyralide"[tw] OR "Cyronal"[tw] OR "dichloropyridine acid"[tw] OR "Dowco 290"[tw] OR "Huiloralid"[tw] OR "Loncid"[tw] OR "Lontrel"[tw] OR "Matrigon"[tw] OR "Transline"[tw] OR "Versatill"[tw] OR **"Camphor"[Mesh]** OR "76-22-2"[rn] OR "21368-68-3"[rn] OR "1, 7, 7 trimethylbicyclo [2.2.1] heptan 2 one"[tw] OR "1,7,7-Trimethyl-bicyclo(2,2,1)Heptan-2-one"[tw] OR "1,7,7-Trimethylbicyclo(2.2.1)-2-heptanone"[tw] OR "1,7,7-Trimethylbicyclo(2.2.1)heptan-2-one"[tw] OR "1,7,7-Trimethylbicyclo[2.2.1]-2-heptanone"[tw] OR "1,7,7-Trimethylbicyclo[2.2.1]heptan-2-one"[tw] OR "1,7,7-Trimethyl-bicyclo[2.2.1]heptan-2-one"[tw] OR "1,7,7-trimethyl-bicyclo[2.2.1]heptane-2-one"[tw] OR "2 bornanone"[tw] OR "2 camphanone"[tw] OR "2 keto 1, 7, 7 trimethylnorcamphane"[tw] OR "2 oxo 1, 7, 7 trimethylbicyclo (2.2.1) heptane"[tw] OR "2 oxobornane"[tw] OR "21368-68-3"[tw] OR "2-Camphanone"[tw] OR "2-Camphonone"[tw] OR "2-Keto-1,7,7-trimethylnorcamphane"[tw] OR "76-22-2"[tw] OR "Alcanfor"[tw] OR "Alphanon"[tw] OR "Bornan-2-one"[tw] OR "camphor"[tw] OR "camphora"[tw] OR "d-2-Bornanone"[tw] OR "d-2-Camphanone"[tw] OR "korodin"[tw] OR "pi hydroxycamphor"[tw] OR "Root bark oil"[tw] OR "Root bark spirit"[tw] OR "sarna"[tw] OR **"fipronil" [Supplementary Concept]** OR "QGH063955F"[rn] OR "120068-37-3"[rn] OR "120068-37-3"[tw] OR "5 amino 1 (2, 6 dichloro alpha, alpha, alpha trifluoro para tolyl) 4 trifluoromethylsulfinylpyrazole 3 carbonitrile"[tw] OR "5 amino 1 [2, 6 dichloro 4 (trifluoromethyl) phenyl] 4 [ (trifluoromethyl) sulfinyl] 1h pyrazole 3 carbonitrile"[tw] OR "5-amino-1 -(2,6-dichloro-4-trifluoromethylphenyl)-4-trifluoromethylsulfinyl-1 H-pyrazole-3-carbonitrile"[tw] OR "5-amino-1-(2,6-dichloro-4-(trifluoromethyl) phenyl)-4-((trifluoromethyl) sulfinyl)-1H-pyrazol-3-carbonitrile"[tw] OR "5-amino-1-(2,6-dichloro-4-(trifluoromethyl)phenyl)-4-((trifluoromethyl)sulfinyl)-1h-pyrazole-3-carbonitrile"[tw] OR "5-amino-1-(2,6-dichloro-alpha,alpha,alpha-trifluoro-p-tolyl)-4-trifluoromethylsulfinylpyrazole-3-carbonitile"[tw] OR "5-Amino-1-[2,6-dichloro-4-(trifluoromethyl)phenyl]-4-(trifluoromethyl)sulfinylpyrazole-3-carbonitrile"[tw] OR "5-amino-1-[2,6-dichloro-4-(trifluoromethyl)phenyl]-4-(trifluoromethylsulfinyl)pyrazole-3-carbonitrile"[tw] OR "5-amino-1-[2,6-dichloro-4-(trifluoromethyl)phenyl]-4-[(trifluoromethyl)sulfinyl]-1H-pyrazole-3-carbonitrile"[tw] OR "5-amino-1-[2,6-dichloro-4-(trifluoromethyl)phenyl]-4-trifluoromethanesulfinyl-1H-pyrazole-3-carbonitrile"[tw] OR "fiprex"[tw] OR "fipronil"[tw] OR "Fluocyanobenpyrazole"[tw] OR "Frontline Spot-on"[tw] OR "Frontline Spray"[tw] OR "Frontline Top Spot"[tw] OR "Goliath gel"[tw] OR "Granedo MC"[tw] OR "Maxforce FC"[tw] OR "QGH063955F"[tw] OR "Termidor"[tw] OR **"Glycerol"[Mesh]** OR "PDC6A3C0OX"[rn] OR "56-81-5"[rn] OR "107283-02-3"[rn] OR "144086-02-2"[rn] OR "144086-03-3"[rn] OR "25618-55-7"[rn] OR "8013-25-0"[rn] OR "1, 2, 3 propanetriol"[tw] OR "1, 2, 3 trihydroxypropane"[tw] OR "1,2,3-Propanetriol"[tw] OR "1,2,3-triglycerol"[tw] OR "1,2,3-TRIHYDROXYPROPAN-2-YL"[tw] OR "1,2,3-trihydroxypropane"[tw] OR "1,2,3-trihydroxypropanol"[tw] OR "1,3-Propanetriol"[tw] OR "1,3-Trihydroxypropane"[tw] OR "107283-02-3"[tw] OR "144086-02-2"[tw] OR "144086-03-3"[tw] OR "25618-55-7"[tw] OR "56-81-5"[tw] OR "8013-25-0"[tw] OR "alditol"[tw] OR "Artificial tears"[tw] OR "Bulbold"[tw] OR "Citifluor AF 2"[tw] OR "Cristal"[tw] OR "Dagralax"[tw] OR "Glicerina"[tw] OR "Glyceol"[tw] OR "glycerin"[tw] OR "Glycerine"[tw] OR "Glycerinum"[tw] OR "Glyceritol"[tw] OR "glycerol"[tw] OR "Glycerolum"[tw] OR "Glycyl alcohol"[tw] OR "Glyrol"[tw] OR "Glysanin"[tw] OR "Glyzerin"[tw] OR "Grocolene"[tw] OR "microglycerin"[tw] OR "Monoctanoin Component D"[tw] OR "Neutracett"[tw] OR "Oelsuess"[tw] OR "Olsuss"[tw] OR "Ophthalgan"[tw] OR "Osmoglyn"[tw] OR "PDC6A3C0OX"[tw] OR "Polyglycerin"[tw] OR "Polyglycerine"[tw] OR "Polyglycerol"[tw] OR "Pricerine 9091"[tw] OR "PROPANE-1,2,3-TRIOL"[tw] OR "Propanetriol"[tw] OR "Tegin M"[tw] OR "Trihydroxypropane"[tw] OR "Tryhydroxypropane"[tw] OR "vilardell"[tw] OR "Vitrosupos"[tw] OR **"hexazinone" [Supplementary Concept]** OR "51235-04-2"[rn] OR "Y51727MR1Y"[rn] OR "3 cyclohexyl 6 (dimethylamino) 1 methyl 1, 3, 5 triazine 2, 4 (1h, 3h) dione"[tw] OR "3-Cyclohexy-6-(dimethylamino)-1-methyl-1,3,5-triazine-2,4(1H,3H)-dione"[tw] OR "3-Cyclohexyl-6-(dimethylamino)-1-methyl-1,3,5-triazine-2,4(1H,3H)-dione"[tw] OR "3-cyclohexyl-6-(dimethylamino)-1-methyl-1,3,5-triazine-2,4-dione"[tw] OR "51235-04-2"[tw] OR "Gridball"[tw] OR "Hexazinoe"[tw] OR "Hexazinon"[tw] OR "Hexazinone"[tw] OR "Velpar"[tw] OR "Y51727MR1Y"[tw] OR **"imazamox" [Supplementary Concept]** OR "UG6793ON5F"[rn] OR "114311-32-9"[rn] OR "114311-32-9"[tw] OR "Imazamox"[tw] OR "UG6793ON5F"[tw] OR **"imazapyr" [Supplementary Concept]** OR "81334-34-1"[rn] OR "2-(4-Isopropyl-4-methyl-5-oxo-2-imidazolin-2-yl)nicotinic acid"[tw] OR "81334-34-1"[tw] OR "Imazapyr"[tw] OR **"Loratadine"[Mesh]** OR ("Cyproheptadine/analogs and derivatives"[Mesh] AND ("1987/01/01"[PDAT] : "1992/12/31"[PDAT])) OR "7AJO3BO7QN"[rn] OR "79794-75-5"[rn] OR "4 (8 chloro 5, 6 dihydro 11h benzo [5, 6] cyclohepta [1, 2 b] pyridin 11 ylidene) 1 piperidinecarboxylic acid ethyl ester"[tw] OR "4-(8-Chloro-5,6-dihydro-11H-benzo(5,6)cyclohepta(1,2-b)pyridin-11-ylidene)-1-piperidinecarboxylic Acid Ethyl Ester"[tw] OR "4-(8-CHLORO-5,6-DIHYDRO-11H-BENZO[5,6]CYCLOHEPTA[1,2-B]PYRIDIN-11-YLIDENE)-1-PIPERIDINECARBOXYLATE"[tw] OR "4-(8-chloro-5,6-dihydro-11H-benzo[5,6]cyclohepta[1,2-b]pyridin-11-ylidene)-1-piperidinecarboxylic acid ethyl ester"[tw] OR "4-(8-Chloro-5,6-dihydro-11H-benzo[5,6]cyclohepta[1,2-b]pyridin-11-ylidene-1-piperidinecarboxylic acid ethyl ester"[tw] OR "79794-75-5"[tw] OR "7AJO3BO7QN"[tw] OR "8 chloro 11 (1 ethoxycarbonyl 4 piperidylidene) 6, 11 dihydro 5h benzo [5, 6] cyclohepta [1, 2 b] pyridine"[tw] OR "8 chloro 6, 11 dihydro 11 (1 carboethoxy 4 piperidylidene) 5h benzo [5, 6] cyclohepta [1, 2 b] pyridine"[tw] OR "8-chloro-11-(1-ethoxycarbonyl-4-piperidylidene)-6,11-dihydro-5H-benzo[5,6]cyclohepta[1,2-b]pyridine"[tw] OR "8-chloro-6,11-dihydro-11-(1- ethoxycarbonyl-4-piperidylidene)-5H-benzo[5,6]cyclohepta[1,2-b]pyridine"[tw] OR "Aerotina"[tw] OR "Alarin"[tw] OR "Alavert"[tw] OR "alerfast"[tw] OR "alernitis"[tw] OR "Alerpriv"[tw] OR "alertadin"[tw] OR "alertrin"[tw] OR "allerta"[tw] OR "Allertidin"[tw] OR "allertyn"[tw] OR "allohex"[tw] OR "ambrace"[tw] OR "analergal"[tw] OR "Anhissen"[tw] OR "anlos"[tw] OR "ardin"[tw] OR "Bactimicina allergy"[tw] OR "Bedix Loratadina"[tw] OR "Biloina"[tw] OR "Bonalerg"[tw] OR "caradine"[tw] OR "carin"[tw] OR "Civeran"[tw] OR "clalodine"[tw] OR "Claratyne"[tw] OR "clarid"[tw] OR "Clarinase"[tw] OR "Claritin"[tw] OR "Claritine"[tw] OR "Clarityn"[tw] OR "Clarityne"[tw] OR "Clarium"[tw] OR "cronitin"[tw] OR "Cronopen"[tw] OR "curyken"[tw] OR "demazin anti-allergy"[tw] OR "ethyl 4 (8 chloro 5, 6 dihydro 11h benzo [5, 6] cyclohepta [1, 2 b] pyridin 11 ylidene) 1 piperidinecarboxylate"[tw] OR "Ethyl 4-(8-chloro-5,6-dihydro-11H-benzo(5,6)cyclohepta(1,2-b)pyridin-11-ylidene)-1-piperidinecarboxylate"[tw] OR "Ethyl 4-(8-chloro-5,6-dihydro-11H-benzo[5,6]cyclohepta[1,2-b]pyridin-11-ylidene)-1-piperidinecarboxylate"[tw] OR "ethyl 4-(8-chloro-5,6-dihydro-11H-benzo[5,6]cyclohepta[1,2-b]pyridin-11-ylidene)piperidine-1-carboxylate"[tw] OR "Ethyl-4-(8-chloro-5,6-dihydro-11H-benzo [5,6] cyclohepta [1,2-b]pyridin-11-ylidene)-1-piperidinecarboxylate"[tw] OR "ezasmin"[tw] OR "ezede"[tw] OR "finska"[tw] OR "Flonidan"[tw] OR "frenaler"[tw] OR "Fristamin"[tw] OR "genadine"[tw] OR "halodin"[tw] OR "hislorex"[tw] OR "histalor"[tw] OR "Histaloran"[tw] OR "j-tadine"[tw] OR "klarihist"[tw] OR "Klaritin"[tw] OR "klinset"[tw] OR "laredine"[tw] OR "lergia"[tw] OR "Lergy"[tw] OR "Lertamine"[tw] OR "Lesidas"[tw] OR "lindine"[tw] OR "Lisino"[tw] OR "lisono"[tw] OR "lobeta"[tw] OR "lodain"[tw] OR "Lomilan"[tw] OR "lorabasics"[tw] OR "Loracert"[tw] OR "loraclar"[tw] OR "loraderm"[tw] OR "Loradex"[tw] OR "Loradif"[tw] OR "loradin"[tw] OR "lorahist"[tw] OR "loralerg"[tw] OR "lora-lich"[tw] OR "lorano"[tw] OR "Loranox"[tw] OR "Lorantis"[tw] OR "lorapaed"[tw] OR "Lorastine"[tw] OR "lora-tabs"[tw] OR "loratadine"[tw] OR "Loratadinum"[tw] OR "loratadura"[tw] OR "loratan"[tw] OR "loratazine"[tw] OR "loratidin"[tw] OR "Loratidine"[tw] OR "loraton"[tw] OR "loratrim"[tw] OR "Loratyne"[tw] OR "Loraver"[tw] OR "loraxin"[tw] OR "loreen"[tw] OR "Lorfast"[tw] OR "lorihis"[tw] OR "lorita"[tw] OR "Loritine"[tw] OR "lotadine"[tw] OR "lotarin"[tw] OR "Lowadina"[tw] OR "mosedin"[tw] OR "noratin"[tw] OR "notamin"[tw] OR "Nularef"[tw] OR "onemin"[tw] OR "Optimin"[tw] OR "Polaratyne"[tw] OR "proactin"[tw] OR "Restamine"[tw] OR "Rhinase"[tw] OR "ridamin"[tw] OR "rihest"[tw] OR "rinityn"[tw] OR "Rinolan"[tw] OR "Rinomex"[tw] OR "rityne"[tw] OR "Roletra"[tw] OR "Sanelor"[tw] OR "Sch 29851"[tw] OR "Sch29851"[tw] OR "Sensibit"[tw] OR "Sinhistan Dy"[tw] OR "Sohotin"[tw] OR "Symphoral"[tw] OR "Tadine"[tw] OR "Talorat Dy"[tw] OR "tidilor"[tw] OR "tirlor"[tw] OR "toradine"[tw] OR "Velodan"[tw] OR "Versal"[tw] OR "voratadine"[tw] OR "Zeos"[tw] OR **"novaluron" [Supplementary Concept]** OR "Z8H1B3CW0B"[rn] OR "116714-46-6"[rn] OR "1-(3-chloro-4-(1,1,2-trifluoro-2-trifluoromethoxyethoxy)phenyl)-3-(2,6-difluorobenzoyl)urea"[tw] OR "1-[3-Chloro-4-(1,1,2-trifluoro-2-trifluoromethoxyethoxy)phenyl]-3-(2,6-difluorobenzoyl)urea"[tw] OR "1-{3-CHLORO-4-[1,1,2-TRIFLUORO-2-(TRIFLUOROMETHOXY)ETHOXY]PHENYL}-3-(2,6-DIFLUOROBENZOYL)UREA"[tw] OR "116714-46-6"[tw] OR "Novaluron"[tw] OR "Rimon EC-10"[tw] OR "Z8H1B3CW0B"[tw] OR **"2-phenylphenol" [Supplementary Concept]** OR "90-43-7"[rn] OR "61788-42-9"[rn] OR "D343Z75HT8"[rn] OR "1-Hydroxy-2-phenylbenzene"[tw] OR "2 biphenylol"[tw] OR "2 hydroxydiphenyl"[tw] OR "2 phenylphenol"[tw] OR "2-Biphenylol"[tw] OR "2-hydroxy biphenyl"[tw] OR "2-Hydroxy-1,1'-biphenyl"[tw] OR "2-Hydroxybiphenyl"[tw] OR "2-Hydroxydiphenyl"[tw] OR "2-Phenyl phenol"[tw] OR "2-Phenylphenol"[tw] OR "61788-42-9"[tw] OR "90-43-7"[tw] OR "Amocid"[tw] OR "Anthrapole 73"[tw] OR "Biphenyl-2-ol"[tw] OR "Biphenylol"[tw] OR "D343Z75HT8"[tw] OR "Dowicide"[tw] OR "Hydroxy-2-phenylbenzene"[tw] OR "Hydroxybiphenyl"[tw] OR "Invalon OP"[tw] OR "Lyorthol"[tw] OR "Nectryl"[tw] OR "Nipacide OPP"[tw] OR "o-Biphenylol"[tw] OR "o-Diphenylol"[tw] OR "o-Hydroxybiphenyl"[tw] OR "o-Hydroxydiphenyl"[tw] OR "o-Phenyl phenol"[tw] OR "o-phenylphenate"[tw] OR "o-phenylphenol"[tw] OR "ortho hydroxybiphenyl"[tw] OR "ortho hydroxydiphenyl"[tw] OR "ortho phenylphenol"[tw] OR "Orthohydroxydiphenyl"[tw] OR "Orthophenyl phenol"[tw] OR "ortho-phenylphenate"[tw] OR "Orthophenylphenol"[tw] OR "Orthoxenol"[tw] OR "o-Xenol"[tw] OR "o-Xonal"[tw] OR "Preventol 3041"[tw] OR "Preventol O extra"[tw] OR "Remol TRF"[tw] OR "Rotoline"[tw] OR "sodium o-phenylphenoate"[tw] OR "Stellisept"[tw] OR "Tetrosin oe"[tw] OR "Torsite"[tw] OR "Xenol"[tw] OR **"Propylene Glycol"[Mesh]** OR "6DC9Q167V3"[rn] OR "57-55-6"[rn] OR "123120-98-9"[rn] OR "63625-56-9"[rn] OR "1, 2 dihydroxypropane"[tw] OR "1, 2 propandiol"[tw] OR "1, 2 propanediol"[tw] OR "1, 2 propylenglycol"[tw] OR "1, 2-propanediol"[tw] OR "1,2 Propanediol"[tw] OR "1,2-(RS)-Propanediol"[tw] OR "1,2-dihydroxypropan-2-yl"[tw] OR "1,2-dihydroxypropane"[tw] OR "1,2-Dihydroxypropanl"[tw] OR "1,2-propandiol"[tw] OR "1,2-propane diol"[tw] OR "1.2-propanediol"[tw] OR "123120-98-9"[tw] OR "2,3-Propanediol"[tw] OR "2-Hydroxypropanol"[tw] OR "57-55-6"[tw] OR "63625-56-9"[tw] OR "6DC9Q167V3"[tw] OR "Aliphatic alcohol"[tw] OR "apopropanediol"[tw] OR "Ilexan P"[tw] OR "Isopropylene glycol"[tw] OR "methyl ethyl glycol"[tw] OR "methyl glycol"[tw] OR "Methylethyl glycol"[tw] OR "Methylethylene glycol"[tw] OR "methylglycol"[tw] OR "Monopropylene glycol"[tw] OR "Prolugen"[tw] OR "propan 1, 2 diol"[tw] OR "Propan-1,2-Diol"[tw] OR "propane 1, 2 diol"[tw] OR "propane-1,2-diol"[tw] OR "propanediol"[tw] OR "propylene glycol"[tw] OR "propyleneglycol"[tw] OR "propylenglycol"[tw] OR "Sirlene"[tw] OR "Trimethyl glycol"[tw] OR **"triclopyr" [Supplementary Concept]** OR "MV06PHJ6I0"[rn] OR "55335-06-3"[rn] OR "3,5,6-TPA"[tw] OR "((3,5,6-trichloro-2-pyridinyl)oxy)-acetic acid"[tw] OR "[(3,5,6-trichloro-2-pyridinyl)oxy]-acetic acid"[tw] OR "3,5,6-Trichloro-2-pyridinyloxyacetic Acid"[tw] OR "((3,5,6-trichloro-2-pyridyl)oxy)-acetic acid"[tw] OR "3,5,6-Trichloro-2-pyridyloxyacetic acid"[tw] OR "55335-06-3"[tw] OR "Garlon"[tw] OR "Grazon ET"[tw] OR "MV06PHJ6I0"[tw] OR "Trichlopyr"[tw] OR "Triclopyr"[tw] OR "Turflon"[tw] OR **"triethylene glycol" [Supplementary Concept]** OR "112-27-6"[rn] OR "103734-98-1"[rn] OR "122784-99-0"[rn] OR "137800-98-7"[rn] OR "145112-98-7"[rn] OR "3P5SU53360"[rn] OR "103734-98-1"[tw] OR "112-27-6"[tw] OR "122784-99-0"[tw] OR "137800-98-7"[tw] OR "145112-98-7"[tw] OR "2, 2' ethylenedioxybis (ethanol)"[tw] OR "2,2'-(Ethylenedioxy)diethanol"[tw] OR "2,2-(Ethylenedioxy)diethanol"[tw] OR "2,2'-Ethylenedioxybis(ethanol)"[tw] OR "2,2'-Ethylenedioxydiethanol"[tw] OR "3, 6 dioxaoctane 1, 8 diol"[tw] OR "3,6-Dioxa-1,8-octanediol"[tw] OR "3,6-Dioxaoctane-1,8-diol"[tw] OR "3P5SU53360"[tw] OR "Bis(2-hydroxyethoxyethane)"[tw] OR "Di-.beta.-hydroxyethoxyethane"[tw] OR "Di-beta-hydroxyethoxyethane"[tw] OR "Ethylene glycol dihydroxydiethyl ether"[tw] OR "Ethylene glycol-bis-(2-hydroxyethyl ether)"[tw] OR "Glycol bis(hydroxyethyl) ether"[tw] OR "Tri-ethylene glycol"[tw] OR "Triethylene Glycol"[tw] OR "Triethyleneglycol"[tw] OR "triethylenglycol"[tw] OR "Trigenos"[tw] OR "Triglycol"[tw] OR "Trigol"[tw] OR **"zoxamide" [Supplementary Concept]** OR "156052-68-5"[rn] OR "156052-68-5"[tw] OR "RH 7281"[tw] OR "RH7281"[tw] OR "Zoxamid"[tw] OR "Zoxamide"[tw] OR "Zoxium"[tw] OR **"pyriproxyfen" [Supplementary Concept]** OR "3Q9VOR705O"[rn] OR "95737-68-1"[rn] OR "126040-81-1"[rn] OR "126040-81-1"[tw] OR "2 [1 methyl 2 (4 phenoxyphenoxy) ethoxy] pyridine"[tw] OR "2-(1-Methyl-2-(4-phenoxyphenoxy)ethoxy)pyridine"[tw] OR "2-[ 1-methyl-2-(4-phenoxyphenoxy)ethoxy]pyridine"[tw] OR "2-[1-methyl-2-(4-phenoxyphenoxy)ethoxy] pyridine"[tw] OR "2-[1-Methyl-2-(4-phenoxyphenoxy)ethoxy]pyridine"[tw] OR "3Q9VOR705O"[tw] OR "4-Phenoxyphenyl (RS)-2-(2-pyridyloxy)propyl ether"[tw] OR "95737-68-1"[tw] OR "Archer IGR"[tw] OR "Cyclio"[tw] OR "juvinal"[tw] OR "NyGuard IGR"[tw] OR "Nylar"[tw] OR "Pyriproxifen"[tw] OR "Pyriproxyfen"[tw] OR "S 31183"[tw] OR "S31183"[tw] OR "Sumilarv"[tw] OR **"methyl cellosolve" [Supplementary Concept]** OR "109-86-4"[rn] OR "9004-74-4"[rn] OR "95507-80-5"[rn] OR "EK1L6XWI56"[rn] OR "109-86-4"[tw] OR "1-Hydroxy-2-methoxyethane"[tw] OR "2-(methoxy)ethanol"[tw] OR "2-(methyloxy)ethanol"[tw] OR "2-HYDROXY-1-METHOXYETHYL"[tw] OR "2-Hydroxyethyl methyl ether"[tw] OR "2-methoxy ethanol"[tw] OR "2-Methoxy-1-ethanol"[tw] OR "2-Methoxyethan-1-Ol"[tw] OR "2methoxyethanol"[tw] OR "2-methoxyethanol"[tw] OR "2-Methoxyethyl alcohol"[tw] OR "2-methoxylethanol"[tw] OR "3-Oxa-1-butanol"[tw] OR "9004-74-4"[tw] OR "95507-80-5"[tw] OR "alpha-hydro-omega-methoxypoly(oxyethylene)"[tw] OR "beta-Methoxyethanol"[tw] OR "dimethyleneglycol monomethylether"[tw] OR "Dowanol 7"[tw] OR "Dowanol EM"[tw] OR "EK1L6XWI56"[tw] OR "Ektasolve EM"[tw] OR "Ethylene glycol methyl ether"[tw] OR "Ethylene glycol monomethyl ether"[tw] OR "ethylene glycol monomethylether"[tw] OR "Ethyleneglycol monomethyl ether"[tw] OR "ethyleneglycol monomethylether"[tw] OR "ethylglycol monomethyl ether"[tw] OR "Glycol ether EM"[tw] OR "Glycol monomethyl ether"[tw] OR "Glycolmethyl ether"[tw] OR "Jeffersol EM"[tw] OR "Karl Fischer Reagent"[tw] OR "Methoxyhydroxyethane"[tw] OR "Methyl cellosolve"[tw] OR "Methyl ethoxol"[tw] OR "Methyl icinol"[tw] OR "Methyl oxitol"[tw] OR "methylcellosolve"[tw] OR "methylcello-solve"[tw] OR "Monoethylene glycol methyl ether"[tw] OR "Monomethyl ether of ethylene glycol"[tw] OR "Monomethyl ethylene glycol ether"[tw] OR "Monomethyl glycol"[tw] OR "O-Methyl Glycol"[tw] OR **"Acetaminophen"[Mesh]** OR "362O9ITL9D"[rn] OR "103-90-2"[rn] OR "103-90-2"[tw] OR "362O9ITL9D"[tw] OR "4 hydroxyacetanilide"[tw] OR "4' hydroxyacetanilide"[tw] OR "4-(Acetylamino)phenol"[tw] OR "4-(N-Acetylamino)phenol"[tw] OR "4-acetamido phenol"[tw] OR "4-ACETAMIDOPHENYLOXIDANYL"[tw] OR "A.F. Anacin"[tw] OR "Abenol"[tw] OR "Abensanil"[tw] OR "Abrol"[tw] OR "Abrolet"[tw] OR "Acamol"[tw] OR "Acenol"[tw] OR "Acephen"[tw] OR "Acertol"[tw] OR "Acetaco"[tw] OR "Acetagesic"[tw] OR "Acetalgin"[tw] OR "Acetamidophenol"[tw] OR "acetamino phenol"[tw] OR "Acetaminofen"[tw] OR "acetaminophen"[tw] OR "acetaminophene"[tw] OR "acetaminophenol"[tw] OR "Acetamol"[tw] OR "Acetavance"[tw] OR "Acetofen"[tw] OR "acetomenophen"[tw] OR "Acetominophen"[tw] OR "acetominophene"[tw] OR "acetylaminophenol"[tw] OR "acetyl-p-aminophenol"[tw] OR "Actamin"[tw] OR "Actimol"[tw] OR "adorem"[tw] OR "Afebrin"[tw] OR "Afebryl"[tw] OR "Aferadol"[tw] OR "Algesidal"[tw] OR "algiafin"[tw] OR "Algina"[tw] OR "algocit"[tw] OR "Algomol"[tw] OR "Algotropyl"[tw] OR "alphagesic"[tw] OR "Alpiny"[tw] OR "Alpinyl"[tw] OR "Alvedon"[tw] OR "Amadil"[tw] OR "Aminofen"[tw] OR "Anacin 3"[tw] OR "Anacin3"[tw] OR "anadin"[tw] OR "Anaflon"[tw] OR "analgiser"[tw] OR "Analter"[tw] OR "Anapap"[tw] OR "Andox"[tw] OR "Anelix"[tw] OR "Anexsia"[tw] OR "Anhiba"[tw] OR "Antidol"[tw] OR "Anuphen"[tw] OR "Apacet"[tw] OR "Apadon"[tw] OR "Apamid"[tw] OR "Apamide"[tw] OR "APAP"[tw] OR "apirex"[tw] OR "Apitrelal"[tw] OR "apotel"[tw] OR "Arfen"[tw] OR "Arthralgen"[tw] OR "Asetam"[tw] OR "Asomal"[tw] OR "Aspac"[tw] OR "Asplin"[tw] OR "atamel"[tw] OR "Atasol"[tw] OR "Atralidon"[tw] OR "Babikan"[tw] OR "Bacetamol"[tw] OR "Banesin"[tw] OR "Benmyo"[tw] OR "benuron"[tw] OR "Ben-u-ron"[tw] OR "Biocetamol"[tw] OR "biogesic"[tw] OR "bodrex"[tw] OR "Bucet"[tw] OR "Butapap"[tw] OR "Cadafen"[tw] OR "Calapol"[tw] OR "Calmanticold"[tw] OR "calodol"[tw] OR "Calonal"[tw] OR "Calpol"[tw] OR "Capital with Codeine"[tw] OR "Captin"[tw] OR "Causalon"[tw] OR "Cefalex"[tw] OR "cemol"[tw] OR "Cetadol"[tw] OR "christamol"[tw] OR "Citramon P"[tw] OR "claradol"[tw] OR "Claratal"[tw] OR "Clixodyne"[tw] OR "clocephen"[tw] OR "Codabrol"[tw] OR "Codalgin"[tw] OR "Codapane"[tw] OR "Codicet"[tw] OR "Codisal"[tw] OR "Codoliprane"[tw] OR "Cofamol"[tw] OR "Co-Gesic"[tw] OR "Conacetol"[tw] OR "Cosutone"[tw] OR "cp 500"[tw] OR "cp500"[tw] OR "Cuponol"[tw] OR "Curadon"[tw] OR "Curpol"[tw] OR "Dafalgan"[tw] OR "Daphalgan"[tw] OR "Darocet"[tw] OR "Darvocet"[tw] OR "Datril"[tw] OR "Demilets"[tw] OR "Deminofen"[tw] OR "Democyl"[tw] OR "Demogripal"[tw] OR "depon"[tw] OR "depyretin"[tw] OR "Desfebre"[tw] OR "Dhamol"[tw] OR "Dimindol"[tw] OR "Dirox"[tw] OR "dismifen"[tw] OR "Disprol"[tw] OR "dolal"[tw] OR "Dolcor"[tw] OR "Dolefin"[tw] OR "dolex"[tw] OR "Dolgesic"[tw] OR "Doliprane"[tw] OR "dolitabs"[tw] OR "Dolko"[tw] OR "dolofen"[tw] OR "Dolofugin"[tw] OR "dolomol"[tw] OR "Doloreduct"[tw] OR "dolorol"[tw] OR "Dolotec"[tw] OR "dolotemp"[tw] OR "Dolprone"[tw] OR "doltem"[tw] OR "Dorocoff"[tw] OR "Dresan"[tw] OR "drilan"[tw] OR "dristan af"[tw] OR "Duaneo"[tw] OR "Dularin"[tw] OR "Duorol"[tw] OR "Duracetamol"[tw] OR "Durapan"[tw] OR "Dymadon"[tw] OR "Ecosetol"[tw] OR "efferalgan"[tw] OR "efferalganodis"[tw] OR "efferelgan"[tw] OR "Elixodyne"[tw] OR "Empracet"[tw] OR "Endecon"[tw] OR "Enelfa"[tw] OR "Eneril"[tw] OR "eraldor"[tw] OR "eu med"[tw] OR "Excipain"[tw] OR "exopon"[tw] OR "expandol"[tw] OR "Fanalgic"[tw] OR "Farmadol"[tw] OR "Febranine"[tw] OR "Febrectal"[tw] OR "Febrectol"[tw] OR "Febrex"[tw] OR "Febricet"[tw] OR "Febridol"[tw] OR "Febrilix"[tw] OR "Febrin"[tw] OR "Febrinol"[tw] OR "Febrolin"[tw] OR "Fendon"[tw] OR "Fensum"[tw] OR "Fepanil"[tw] OR "fervex"[tw] OR "fibrinol"[tw] OR "Finimal"[tw] OR "Fluparmol"[tw] OR "fortolin"[tw] OR "Gelocatil"[tw] OR "Geluprane"[tw] OR "Genapap"[tw] OR "Genebs"[tw] OR "Grippostad"[tw] OR "Gynospasmine"[tw] OR "Hedex"[tw] OR "helporal"[tw] OR "Homoolan"[tw] OR "Hydroxyacetanilide"[tw] OR "Hy-Phen"[tw] OR "Ildamol"[tw] OR "Inalgex"[tw] OR "infants' feverall"[tw] OR "Injectapap"[tw] OR "Intensin"[tw] OR "Janupap"[tw] OR "kamolas"[tw] OR "Kataprin"[tw] OR "Korum"[tw] OR "kyofen"[tw] OR "Labamol"[tw] OR "Lekadol"[tw] OR "Lemgrip"[tw] OR "Lemsip"[tw] OR "Lestemp"[tw] OR "letamol"[tw] OR "Liqiprine"[tw] OR "liquiprin"[tw] OR "Lonarid"[tw] OR "lotemp"[tw] OR "Lupocet"[tw] OR "Lyteca"[tw] OR "Magnidol"[tw] OR "Malgis"[tw] OR "Malidens"[tw] OR "Maxadol"[tw] OR "medamol"[tw] OR "Medocodene"[tw] OR "meforagesic"[tw] OR "Melabon Infantil"[tw] OR "metagesic"[tw] OR "metalid"[tw] OR "Mexalen"[tw] OR "Midol"[tw] OR "Minafen"[tw] OR "minopan"[tw] OR "Minoset"[tw] OR "Miralgin"[tw] OR "Mono Praecimed"[tw] OR "n acetyl 4 aminophenol"[tw] OR "n acetyl para aminophenol"[tw] OR "N-(4-Hydroxyphenyl)acetamide"[tw] OR "N-(4-hydroxyphenyl)-acetamide"[tw] OR "N-(4-Hydroxyphenyl)acetanilide"[tw] OR "N-(4-hydroxyphenyl)ethanamide"[tw] OR "n-acetyl-4-hydroxyaniline"[tw] OR "N-Acetyl-p-aminophenol"[tw] OR "Naldegesic"[tw] OR "nalgesik"[tw] OR "Napafen"[tw] OR "napamol"[tw] OR "NAPAP"[tw] OR "naprex"[tw] OR "Naprinol"[tw] OR "Nealgyl"[tw] OR "NeoCitran"[tw] OR "neodalmin"[tw] OR "Neodol"[tw] OR "Neodolito"[tw] OR "Neo-Fepramol"[tw] OR "Neopap"[tw] OR "Neuridon"[tw] OR "nevral"[tw] OR "nilapur"[tw] OR "Nobedon"[tw] OR "Nodolex"[tw] OR "Noral"[tw] OR "Norco"[tw] OR "nysacetol"[tw] OR "Ofirmev"[tw] OR "Oltyl"[tw] OR "Oralgan"[tw] OR "Oraphen-PD"[tw] OR "Ortensan"[tw] OR "Oxycocet"[tw] OR "p-(Acetylamino)phenol"[tw] OR "Paceco"[tw] OR "Pacemo"[tw] OR "Pacemol"[tw] OR "Pacet"[tw] OR "p-Acetamidophenol"[tw] OR "p-Acetaminophenol"[tw] OR "p-Acetoaminophen"[tw] OR "p-Acetylaminophenol"[tw] OR "Pacimol"[tw] OR "Paedialgon"[tw] OR "Paedol"[tw] OR "Painex"[tw] OR "Paldesic"[tw] OR "Pamol"[tw] OR "Panacete"[tw] OR "Panadeine"[tw] OR "Panadiene"[tw] OR "Panado-Co"[tw] OR "Panadol"[tw] OR "Panaleve"[tw] OR "Panamax"[tw] OR "Panasorb"[tw] OR "Panasorbe"[tw] OR "Panex"[tw] OR "Panodil"[tw] OR "Panofen"[tw] OR "Pantalgin"[tw] OR "para acetamidophenol"[tw] OR "para acetylaminophenol"[tw] OR "para hydroxyacetanilide"[tw] OR "para suppo"[tw] OR "Paracemol"[tw] OR "Paracenol"[tw] OR "Paracet"[tw] OR "paracetaminophenol"[tw] OR "Paracetamol"[tw] OR "Paracetamole"[tw] OR "Paracetamolum"[tw] OR "Paracetanol"[tw] OR "Paracetol"[tw] OR "Paracin"[tw] OR "Paracod"[tw] OR "Paracodol"[tw] OR "Parador"[tw] OR "parageniol"[tw] OR "paragin"[tw] OR "Parakapton"[tw] OR "Parake"[tw] OR "Paralen"[tw] OR "Paralief"[tw] OR "Paralink"[tw] OR "Paralyoc"[tw] OR "paramax"[tw] OR "paramidol"[tw] OR "Paramol"[tw] OR "Paramolan"[tw] OR "Paranox"[tw] OR "parapaed"[tw] OR "Parapan"[tw] OR "Parasedol"[tw] OR "Parasin"[tw] OR "Paraspen"[tw] OR "paratabs"[tw] OR "Para-Tabs"[tw] OR "Parcetol"[tw] OR "Parelan"[tw] OR "Parmol"[tw] OR "Parogal"[tw] OR "Paroma"[tw] OR "parvid"[tw] OR "Pasolind"[tw] OR "paximol"[tw] OR "Pediapirin"[tw] OR "Pediatrix"[tw] OR "pedipan"[tw] OR "Pedric"[tw] OR "Perdolan Mono"[tw] OR "Perfalgan"[tw] OR "Phenaphen"[tw] OR "Phendon"[tw] OR "Phenipirin"[tw] OR "Phogoglandin"[tw] OR "Phrenilin"[tw] OR "p-hydroxy-acetanilid"[tw] OR "p-Hydroxyacetanilide"[tw] OR "p-hydroxyacetoanilide"[tw] OR "p-Hydroxyphenolacetamide"[tw] OR "Pinex"[tw] OR "Piramin"[tw] OR "Pirinasol"[tw] OR "Plicet"[tw] OR "polarfen"[tw] OR "Polmofen"[tw] OR "Predimol"[tw] OR "Prodol"[tw] OR "Prontina"[tw] OR "Propacet"[tw] OR "Puernol"[tw] OR "Pulmofen"[tw] OR "Pyrigesic"[tw] OR "Pyrinazine"[tw] OR "Pyromed"[tw] OR "Quiet World"[tw] OR "raperon"[tw] OR "rapidol"[tw] OR "Redutemp"[tw] OR "relaphen"[tw] OR "Reliv"[tw] OR "Remedol"[tw] OR "Resfenol"[tw] OR "Resprin"[tw] OR "revanin"[tw] OR "rhodapap"[tw] OR "Rivalgyl"[tw] OR "Robigesic"[tw] OR "Rounox"[tw] OR "Rubophen"[tw] OR "Rupemol"[tw] OR "Salzone"[tw] OR "Sanicet"[tw] OR "Sanicopyrine"[tw] OR "Scanol"[tw] OR "Sedalito"[tw] OR "Sedapap"[tw] OR "sedes a"[tw] OR "Semolacin"[tw] OR "serimol"[tw] OR "Servigesic"[tw] OR "Seskamol"[tw] OR "Setakop"[tw] OR "Setamol"[tw] OR "Setol"[tw] OR "Sifenol"[tw] OR "Sinaspril"[tw] OR "Sine-Aid"[tw] OR "sinebriv"[tw] OR "Sinedol"[tw] OR "Sine-Off"[tw] OR "Sinmol"[tw] OR "sinpro"[tw] OR "Sinubid"[tw] OR "Snaplets-FR"[tw] OR "St Joseph Aspirin-Free"[tw] OR "Stanback"[tw] OR "Stopain"[tw] OR "Sunetheton"[tw] OR "Supadol mono"[tw] OR "Supofen"[tw] OR "Suppap"[tw] OR "Supramol-M"[tw] OR "Tabalgin"[tw] OR "tachipirin"[tw] OR "tachipirina"[tw] OR "taganopain"[tw] OR "Talacen"[tw] OR "Tapanol"[tw] OR "Tapar"[tw] OR "Tazamol"[tw] OR "Tempanal"[tw] OR "Tempra"[tw] OR "tempte"[tw] OR "Tencon"[tw] OR "Termacet"[tw] OR "Termalgin"[tw] OR "Termalgine"[tw] OR "Termofren"[tw] OR "Theraflu"[tw] OR "Tiffy"[tw] OR "Titralgan"[tw] OR "Toximer P"[tw] OR "Tralgon"[tw] OR "tramil"[tw] OR "Treupel N"[tw] OR "Treuphadol"[tw] OR "Triaprin"[tw] OR "Tricoton"[tw] OR "turpan"[tw] OR "Tussapap"[tw] OR "Tycolet"[tw] OR "Tylenol"[tw] OR "Tylex"[tw] OR "Tylol"[tw] OR "Tylox"[tw] OR "Tymol"[tw] OR "Upsanol"[tw] OR "Utragin"[tw] OR "Valadol"[tw] OR "Valgesic"[tw] OR "Valorin"[tw] OR "Veralgina"[tw] OR "Vermidon"[tw] OR "Verpol"[tw] OR "Vicodin"[tw] OR "Vivimed"[tw] OR "Volpan"[tw] OR "wegmal"[tw] OR "winadol"[tw] OR "winasorb"[tw] OR "Wygesic"[tw] OR "xebramol"[tw] OR "Zatinol"[tw] OR "Zolben"[tw] OR "zydinol"[tw] OR "Zydone"[tw] OR **"Ascorbic Acid"[Mesh:NoExp]** OR "PQ6CK8PD0R"[rn] OR "50-81-7"[rn] OR "53262-66-1"[rn] OR "1-Xyloascorbic Acid"[tw] OR "50-81-7"[tw] OR "53262-66-1"[tw] OR "Acidum ascorbicum"[tw] OR "acidylina"[tw] OR "adenex"[tw] OR "agrumina"[tw] OR "allercorb"[tw] OR "allescorb"[tw] OR "Antiscorbic vitamin"[tw] OR "Antiscorbutic factor"[tw] OR "antiscorbutic vitamin"[tw] OR "arcavit c"[tw] OR "arcavite c"[tw] OR "arkovital c"[tw] OR "ascelat"[tw] OR "ascofar"[tw] OR "Ascoltin"[tw] OR "ascomed"[tw] OR "asconvita"[tw] OR "ascor"[tw] OR "ascorbate"[tw] OR "ascorbic acid"[tw] OR "ascorbicap"[tw] OR "ascorbicin"[tw] OR "ascorbico"[tw] OR "ascorbin"[tw] OR "ascorbina"[tw] OR "ascorbinic acid"[tw] OR "Ascorbinsaure"[tw] OR "ascorbit"[tw] OR "ascorbite"[tw] OR "ascorbitol"[tw] OR "ascorbivit"[tw] OR "ascorbivite"[tw] OR "ascorbone"[tw] OR "ascorbutina"[tw] OR "ascorbyl"[tw] OR "ascorbyn"[tw] OR "ascorgil"[tw] OR "ascorin"[tw] OR "ascormin"[tw] OR "ascorteal"[tw] OR "ascorval"[tw] OR "ascorvel"[tw] OR "ascorvit"[tw] OR "ascorvite"[tw] OR "ascorvitina"[tw] OR "askorbin"[tw] OR "austrovit c"[tw] OR "austrovite c"[tw] OR "bentavit c"[tw] OR "bentavite c"[tw] OR "c tamin"[tw] OR "c vimin"[tw] OR "c vit"[tw] OR "c vita"[tw] OR "cantan"[tw] OR "cantaxin"[tw] OR "catavin c"[tw] OR "ce vi sol"[tw] OR "cebetate"[tw] OR "cebicure"[tw] OR "Cebid"[tw] OR "cebion"[tw] OR "cebione"[tw] OR "cecap"[tw] OR "cecon"[tw] OR "cecone"[tw] OR "cecorbin"[tw] OR "cecorbine"[tw] OR "cecorbyl"[tw] OR "cecorbyle"[tw] OR "cecrisina"[tw] OR "cedon"[tw] OR "cedone"[tw] OR "cedoxon"[tw] OR "cedoxone"[tw] OR "ceevifil"[tw] OR "cegiolan"[tw] OR "Ceklin"[tw] OR "celaskon"[tw] OR "celaskone"[tw] OR "celin"[tw] OR "Cemagyl"[tw] OR "Cemill"[tw] OR "cenetone"[tw] OR "cenol"[tw] OR "cenolate"[tw] OR "cequinyl"[tw] OR "cereon"[tw] OR "cergona"[tw] OR "cescorbat"[tw] OR "cetamican"[tw] OR "cetamid"[tw] OR "cetamine"[tw] OR "cetebe"[tw] OR "Cetemican"[tw] OR "ceterapion"[tw] OR "ceterapione"[tw] OR "cetrinets"[tw] OR "cevalin"[tw] OR "cevaline"[tw] OR "cevatine"[tw] OR "cevex"[tw] OR "cevibid"[tw] OR "Cevi-Bid"[tw] OR "cevibram"[tw] OR "cevigal"[tw] OR "cevigen"[tw] OR "cevigol"[tw] OR "cevilat"[tw] OR "cevimin"[tw] OR "cevimine"[tw] OR "cevisol"[tw] OR "ce-vi-sol"[tw] OR "cevit"[tw] OR "cevita"[tw] OR "Cevital"[tw] OR "Cevitamate"[tw] OR "cevitamic acid"[tw] OR "cevitamin"[tw] OR "cevitaminic acid"[tw] OR "cevitan"[tw] OR "cevite"[tw] OR "cevitex"[tw] OR "cevitil"[tw] OR "cevitol"[tw] OR "cewin"[tw] OR "chewcee"[tw] OR "chivibit c"[tw] OR "Chromagen"[tw] OR "ciamin"[tw] OR "ciergin"[tw] OR "cifilina"[tw] OR "cipca"[tw] OR "cisir"[tw] OR "citamino"[tw] OR "citoascorbina"[tw] OR "citoxyl"[tw] OR "citran"[tw] OR "citravite"[tw] OR "Citriscorb"[tw] OR "citritabs"[tw] OR "Citrovit"[tw] OR "citrovitamina"[tw] OR "civigor"[tw] OR "civitin"[tw] OR "civitine"[tw] OR "Colascor"[tw] OR "concemin"[tw] OR "cortalex"[tw] OR "c-vimin"[tw] OR "dagrascorbin"[tw] OR "dagravit c"[tw] OR "dancimin c"[tw] OR "davitamon c"[tw] OR "dayvital"[tw] OR "difvitamin c"[tw] OR "dumovit c"[tw] OR "dumovite c"[tw] OR "Duoscorb"[tw] OR "erftamin c"[tw] OR "erftamine c"[tw] OR "esuron"[tw] OR "esurvit"[tw] OR "esurvite"[tw] OR "Ferancee"[tw] OR "godabion c"[tw] OR "gregovite c"[tw] OR "hicee"[tw] OR "hybrin"[tw] OR "ido c"[tw] OR "inovitan c"[tw] OR "irocevit"[tw] OR "irocevite"[tw] OR "jarexin"[tw] OR "jarexine"[tw] OR "Juvamine"[tw] OR "l 3 keto hexuronic acid lactone"[tw] OR "L-3-ketothreohexuronic acid"[tw] OR "lacivit"[tw] OR "lacivite"[tw] OR "laroscorbine"[tw] OR "L-AscorbicAcid"[tw] OR "leder c"[tw] OR "lemascorb"[tw] OR "limcee"[tw] OR "L-lyxoascorbate"[tw] OR "L-Lyxoascorbic acid"[tw] OR "L-Threoascorbic acid"[tw] OR "L-threo-Ascorbic acid"[tw] OR "L-xyloascorbate"[tw] OR "Magnorbin"[tw] OR "myascorbin"[tw] OR "natrascorb"[tw] OR "novo ascorbic"[tw] OR "nybadol"[tw] OR "Parentrovite"[tw] OR "parkovit c"[tw] OR "pharmascorbine"[tw] OR "pharmatovit c"[tw] OR "pharmatovite c"[tw] OR "planavit c"[tw] OR "planavite c"[tw] OR "plivit c"[tw] OR "plivite c"[tw] OR "PQ6CK8PD0R"[tw] OR "proscorbin"[tw] OR "proscorbine"[tw] OR "redoxon"[tw] OR "ribena"[tw] OR "Rovimix C"[tw] OR "scorbacid"[tw] OR "scorbacide"[tw] OR "scorbex"[tw] OR "scorbin c"[tw] OR "scorbitol"[tw] OR "scorbumine"[tw] OR "scottavit c"[tw] OR "scottavite c"[tw] OR "secorbate"[tw] OR "Semidehydroascorbate"[tw] OR "sevalin"[tw] OR "sigmavit c"[tw] OR "sigmavite c"[tw] OR "sodascorbate"[tw] OR "Sunkist"[tw] OR "synum c"[tw] OR "tanvimil-c"[tw] OR "testascorbic"[tw] OR "Tolfrinic"[tw] OR "ucemine c"[tw] OR "upsa-c"[tw] OR "vicef"[tw] OR "vicelat"[tw] OR "vicetrin"[tw] OR "viciman"[tw] OR "vicin"[tw] OR "vicitina"[tw] OR "vicon"[tw] OR "viforcit"[tw] OR "viforcite"[tw] OR "viscorin"[tw] OR "viscorine"[tw] OR "vitace"[tw] OR "vitacee"[tw] OR "vitacimin"[tw] OR "vitacimine"[tw] OR "vitacin"[tw] OR "vitacine"[tw] OR "vitamin C"[tw] OR "Vitamisin"[tw] OR "vitaplex c"[tw] OR "vitapric"[tw] OR "vitapur c"[tw] OR "vitasan c"[tw] OR "vitascorbin"[tw] OR "vitascorbine"[tw] OR "vitascorbol"[tw] OR "vitelix c"[tw] OR "wandervit c"[tw] OR "wandervite c"[tw] OR "witamina c"[tw] OR "xitix"[tw] OR "xyloascorbic acid"[tw] OR **"butylparaben" [Supplementary Concept]** OR "3QPI1U3FV8"[rn] OR "94-26-8"[rn] OR "3QPI1U3FV8"[tw] OR "4 hydroxybenzoic acid butyl ester"[tw] OR "4-(Butoxycarbonyl)phenol"[tw] OR "4-Hydroxybenzoic acid-n-butyl ester"[tw] OR "94-26-8"[tw] OR "Aseptoform butyl"[tw] OR "butoben"[tw] OR "butyl 4 hydroxybenzoate"[tw] OR "butyl butex"[tw] OR "butyl hydroxybenzoic acid"[tw] OR "butyl para hydroxybenzoate"[tw] OR "Butyl paraben"[tw] OR "butyl parabenbutyl parahydroxybenzoatebutyl p-hydroxybenzoate"[tw] OR "Butyl parahydroxybenzoate"[tw] OR "Butyl p-hydroxybenzoate"[tw] OR "butylhydroxybenzoate"[tw] OR "butylparaben"[tw] OR "n-Butyl hydroxybenzoate"[tw] OR "Nipabutyl"[tw] OR "para hydroxybenzoic acid butyl ester"[tw] OR "p-Hydroxy butyl benzoate"[tw] OR "p-Hydroxybenzoic acid butyl ester"[tw] OR "p-Hydroxybenzoic acid n-butyl ester"[tw] OR "p-Hydroxybenzoic butyl ester"[tw] OR **"Carbamazepine"[Mesh]** OR "33CM23913M"[rn] OR "298-46-4"[rn] OR "(5h) dibenz (b, f) azepine 5 carboxamide"[tw] OR "298-46-4"[tw] OR "33CM23913M"[tw] OR "5 carbamoyl 5h dibenz [b, f] azepine"[tw] OR "5-Carbamoyl-5H-dibenz(b,f)azepine"[tw] OR "5-Carbamoyl-5H-dibenz[b,f]azepine"[tw] OR "5-Carbamoyl-5H-dibenzo(b,f)azepine"[tw] OR "5h dibenz [b, f] azepine 5 carboxamide"[tw] OR "5H-Dibenz(b,f)azepine-5-carboxamide"[tw] OR "5H-Dibenz[ b, f]azepine-5-carboxamide"[tw] OR "5H-Dibenz[b,f]azepine-5-carboxamide"[tw] OR "5H-Dibenzo[b,f]azepine-5-carboxamide"[tw] OR "amizepin"[tw] OR "amizepine"[tw] OR "apo-carbamazepine"[tw] OR "atretol"[tw] OR "Bipotrol"[tw] OR "biston"[tw] OR "calepsin"[tw] OR "camapine"[tw] OR "carbadac"[tw] OR "Carbamazepen"[tw] OR "carbamazepin"[tw] OR "carbamazepine"[tw] OR "Carbamazepinum"[tw] OR "Carbamezepine"[tw] OR "carbategral"[tw] OR "carbatol"[tw] OR "carbatrol"[tw] OR "carbazene"[tw] OR "carbazep"[tw] OR "Carbazepin"[tw] OR "Carbazepine"[tw] OR "carbazina"[tw] OR "Carbelan"[tw] OR "carmaz"[tw] OR "carnexiv"[tw] OR "carpaz"[tw] OR "carzepin"[tw] OR "carzepine"[tw] OR "clostedal"[tw] OR "convuline"[tw] OR "epileptol"[tw] OR "epimax"[tw] OR "epitol"[tw] OR "equetro"[tw] OR "espa-lepsin"[tw] OR "finlepsin"[tw] OR "foxalepsin"[tw] OR "g 32883"[tw] OR "g32883"[tw] OR "hermolepsin"[tw] OR "Iminostilbene-N-carboxamide"[tw] OR "karbamazepin"[tw] OR "kodapan"[tw] OR "lexin"[tw] OR "mazepine"[tw] OR "mazetol"[tw] OR "neugeron"[tw] OR "neurotol"[tw] OR "neurotop"[tw] OR "nordotol"[tw] OR "Novo-Carbamaz"[tw] OR "panitol"[tw] OR "servimazepin"[tw] OR "sirtal"[tw] OR "spd 417"[tw] OR "spd417"[tw] OR "Stazepin"[tw] OR "Stazepine"[tw] OR "tardotol"[tw] OR "taver"[tw] OR "tegol"[tw] OR "tegral"[tw] OR "tegretal"[tw] OR "tegretol"[tw] OR "tegrital"[tw] OR "telesmin"[tw] OR "temporol"[tw] OR "teril"[tw] OR "timonil"[tw] OR "Trimonil"[tw] OR **"dimethyl phthalate" [Supplementary Concept]** OR "08X7F5UDJM"[rn] OR "131-11-3"[rn] OR "08X7F5UDJM"[tw] OR "131-11-3"[tw] OR "avolin"[tw] OR "citrola"[tw] OR "Dimethyl benzeneorthodicarboxylate"[tw] OR "Dimethyl o-phthalate"[tw] OR "Dimethyl orthophthalate"[tw] OR "dimethyl phthalate"[tw] OR "dimethylphthalate"[tw] OR "dmp 30"[tw] OR "dmp30"[tw] OR "fermine"[tw] OR "Kemester DMP"[tw] OR "Kodaflex DMP"[tw] OR "methyl phthalate"[tw] OR "mipax"[tw] OR "mugia"[tw] OR "palatinol m"[tw] OR "Phthalic acid dimethyl ester"[tw] OR "Repeftal"[tw] OR "sketofax"[tw] OR "Solvanom"[tw] OR "Solvarone"[tw] OR "Unimoll DM"[tw] OR "Uniplex 110"[tw] OR **"dimethylamine" [Supplementary Concept]** OR "124-40-3"[rn] OR "6912-12-5"[rn] OR "ARQ8157E0Q"[rn] OR "124-40-3"[tw] OR "6912-12-5"[tw] OR "ARQ8157E0Q"[tw] OR "dimethlamine"[tw] OR "dimethyamine"[tw] OR "dimethyl amine"[tw] OR "dimethylamine"[tw] OR "di-methylamine"[tw] OR "dimethylammonia"[tw] OR "dimethylammonium chloride"[tw] OR "dimethylammonium formate"[tw] OR "dimethylarnine"[tw] OR "dirnethylamine"[tw] OR **"Penicillamine"[Mesh:NoExp]** OR "GNN1DV99GX"[rn] OR "52-67-5"[rn] OR "3, 3 dimethylcysteine"[tw] OR "3,3-Dimethyl-D(-)-cysteine"[tw] OR "3,3-Dimethyl-D-cysteine"[tw] OR "3-Mercapto-D-valine"[tw] OR "3-sulfanyl-D-valine"[tw] OR "52-67-5"[tw] OR "adaleen"[tw] OR "alpha amino beta methyl beta mercaptobutyric acid"[tw] OR "artamin"[tw] OR "Artamine"[tw] OR "atamir"[tw] OR "beta, beta dimethylcysteamine"[tw] OR "beta-Thiovaline"[tw] OR "byanodine"[tw] OR "Copper penicillaminate"[tw] OR "cuprenil"[tw] OR "cuprim"[tw] OR "cuprimin"[tw] OR "cuprimine"[tw] OR "cuprimune"[tw] OR "cupripen"[tw] OR "Depamine"[tw] OR "depen"[tw] OR "dextropenicillamine"[tw] OR "dimethyl cysteine"[tw] OR "Dimethylcysteine"[tw] OR "distamine"[tw] OR "d-penamine"[tw] OR "D-Penicilamine"[tw] OR "D-Penicyllamine"[tw] OR "d-penil"[tw] OR "gerodyl"[tw] OR "GNN1DV99GX"[tw] OR "kelatin"[tw] OR "kelatine"[tw] OR "Kuprenil"[tw] OR "Mercaptovaline"[tw] OR "mercaptyl"[tw] OR "metalcaptase"[tw] OR "pemine"[tw] OR "pendramine"[tw] OR "penicillame"[tw] OR "penicillamin"[tw] OR "Penicillamina"[tw] OR "penicillamine"[tw] OR "Penicillaminum"[tw] OR "penicillinamine"[tw] OR "Perdolat"[tw] OR "Sufirtan"[tw] OR "Sufortan"[tw] OR "sufortanon"[tw] OR "trolovol"[tw] OR **"Folic Acid"[Mesh:NoExp]** OR "935E97BOY8"[rn] OR "59-30-3"[rn] OR "32108-06-8"[rn] OR "32108-06-8"[tw] OR "59-30-3"[tw] OR "935E97BOY8"[tw] OR "acfol"[tw] OR "Acidum folicum"[tw] OR "Acifolic"[tw] OR "Aspol"[tw] OR "Cytofol"[tw] OR "Dosfolat B activ"[tw] OR "Facid"[tw] OR "filicine"[tw] OR "Folacid"[tw] OR "folacin"[tw] OR "Folan"[tw] OR "folart"[tw] OR "Folasic"[tw] OR "folate"[tw] OR "Folbal"[tw] OR "Folcidin"[tw] OR "Folcysteine"[tw] OR "foldine"[tw] OR "Folettes"[tw] OR "foliamin"[tw] OR "Folic"[tw] OR "folicet"[tw] OR "folicid"[tw] OR "folinsyre"[tw] OR "Folipac"[tw] OR "folitab"[tw] OR "folium acid"[tw] OR "folivit"[tw] OR "Folovit"[tw] OR "Folsaeure"[tw] OR "folsan"[tw] OR "Folsaure"[tw] OR "Folsav"[tw] OR "folverlan"[tw] OR "folvite"[tw] OR "Folvron"[tw] OR "gravi-fol"[tw] OR "Incafolic"[tw] OR "ingafol"[tw] OR "lactobacillus casei factor"[tw] OR "lafol"[tw] OR "lexpec"[tw] OR "megafol"[tw] OR "Millafol"[tw] OR "Mittafol"[tw] OR "n [para [ (2 amino 4 hydroxy 6 pteridylmethyl) amino] benzoyl] glutamic acid"[tw] OR "N-(p-(((2-Amino-4-hydroxy-6-pteridinyl)methyl)amino)benzoyl)-L-glutamic acid"[tw] OR "N-{p-[(2-amino-4-hydroxypteridin-6-yl)methylamino]benzoyl}glutamic acid"[tw] OR "neocepri"[tw] OR "Novofolacid"[tw] OR "nsc 3073"[tw] OR "PteGlu"[tw] OR "Pteroyglutamic acid"[tw] OR "pteroyl glutamate"[tw] OR "pteroyl l glutamic acid"[tw] OR "pteroyl monoglutamate"[tw] OR "pteroylglutamate"[tw] OR "pteroylglutamic acid"[tw] OR "Pteroyl-L-glutamate"[tw] OR "Pteroyl-L-monoglutamate"[tw] OR "Pteroyl-L-monoglutamic acid"[tw] OR "pteroylmonoglutamate"[tw] OR "pteroylmonoglutamic acid"[tw] OR "rubiefol"[tw] OR "vifolin"[tw] OR "Vitamin B11"[tw] OR "Vitamin B9"[tw] OR "vitamin bc"[tw] OR "vitamin m"[tw] OR **"Genistein"[Mesh]** OR "DH2M523P0H"[rn] OR "446-72-0"[rn] OR "690224-00-1"[rn] OR "4', 5, 7 trihydroxyisoflavone"[tw] OR "4',5, 7-Trihydroxyisoflavone"[tw] OR "4,5,7-Trihydroxy Iso-Flavone"[tw] OR "4',5,7-Trihydroxy isoflavone"[tw] OR "4,5,7-Trihydroxyisoflavone"[tw] OR "4',5,7-Trihydroxyisoflavone"[tw] OR "446-72-0"[tw] OR "690224-00-1"[tw] OR "Bonistein"[tw] OR "DH2M523P0H"[tw] OR "differenol a"[tw] OR "Genestein"[tw] OR "genistein"[tw] OR "genisteine"[tw] OR "Genisteol"[tw] OR "Genisterin"[tw] OR "prunetol"[tw] OR "Sophoricol"[tw] OR **"methoxyacetic acid" [Supplementary Concept]** OR "625-45-6"[rn] OR "F11T1H7Q7W"[rn] OR "(methyloxy)acetic acid"[tw] OR "625-45-6"[tw] OR "F11T1H7Q7W"[tw] OR "methoxy acetic acid"[tw] OR "methoxyacetate"[tw] OR "Methoxyacetic acid"[tw] OR "methoxyessigs"[tw] OR "Methoxyethanoic acid"[tw] OR "methyloxyacetic acid"[tw] OR **"N-methylpyrrolidone" [Supplementary Concept]** OR "JR9CE63FPM"[rn] OR "872-50-4"[rn] OR "30207-69-3"[rn] OR "51013-18-4"[rn] OR "1 methyl 2 pyrrolidene"[tw] OR "1-methyl pyrrolidinone"[tw] OR "1-methyl-2-pyrolidinone"[tw] OR "1-Methylazacyclopentan-2-one"[tw] OR "1-Methylazacyclopentane-2-one"[tw] OR "1-methylpyrrolid-2-one"[tw] OR "1-methyl-pyrrolidin-2-one"[tw] OR "1-methylpyrrolidine-2-one"[tw] OR "1-N-methyl-2-pyrrolidinone"[tw] OR "30207-69-3"[tw] OR "51013-18-4"[tw] OR "872-50-4"[tw] OR "Agsolex 1"[tw] OR "JR9CE63FPM"[tw] OR "methyl pyrrolidone"[tw] OR "Methyl-2-pyrrolidinone"[tw] OR "methyl-2-pyrrolidone"[tw] OR "methylpyrrolidin-2-one"[tw] OR "Methylpyrrolidinone"[tw] OR "Methylpyrrolidone"[tw] OR "M-Pyrol"[tw] OR "N-methyl 2-pyrolidone"[tw] OR "N-methyl pirrolidone"[tw] OR "N-methyl pyrollidone"[tw] OR "N-methyl pyrrolidinone"[tw] OR "n-methyl pyrrolidon"[tw] OR "N-Methyl-.alpha.-pyrrolidinone"[tw] OR "N-Methyl-.alpha.-pyrrolidone"[tw] OR "N-Methyl-.gamma.-butyrolactam"[tw] OR "N-methyl-2-pyrolidinone"[tw] OR "N-methyl-2-pyrolidone"[tw] OR "N-methyl-2-pyrrolidinon"[tw] OR "N-Methyl-2-pyrrolidon"[tw] OR "N-Methyl-alpha-pyrrolidinone"[tw] OR "N-Methyl-alpha-pyrrolidone"[tw] OR "n-methylbutyrolactam"[tw] OR "N-Methyl-gamma-butyrolactam"[tw] OR "N-methylpyrolidin-2-one"[tw] OR "N-Methylpyrrolid-2-one"[tw] OR "N-methyl-pyrrolid-2-one"[tw] OR "N-methyl-pyrrolidin-2-one"[tw] OR "N-methylpyrrolidin-2-one"[tw] OR "N-methylpyrrolidine-2-one"[tw] OR "N-Methylpyrrolidinon"[tw] OR "N-Methylpyrrolidinone"[tw] OR "N-Methylpyrrolidon"[tw] OR "N-methyl-pyrrolidon"[tw] OR "pharmasolve"[tw] OR "Pyrol M"[tw] OR **"o,p'-DDT" [Supplementary Concept]** OR "D4K93Z1TBH"[rn] OR "789-02-6"[rn] OR "1 (2 chlorophenyl) 1 (4 chlorophenyl) 2, 2, 2 trichloroethane"[tw] OR "1 (ortho chlorophenyl) 1 (para chlorophenyl) 2, 2, 2 trichloroethane"[tw] OR "1, 1, 1 trichloro 2 (ortho chlorophenyl) 2 (para chlorophenyl) ethane"[tw] OR "1,1,1-trichloro-2-(2-chlorophenyl)-2-(4-chlorophenyl)ethane"[tw] OR "1,1,1-Trichloro-2-(o-chlorophenyl)-2-(p-chlorophenyl)ethane"[tw] OR "1-Chloro-2-(2,2,2-trichloro-1-(4-chlorophenyl)ethyl)benzene"[tw] OR "1-Chloro-2-[2,2,2-trichloro-1-(4-chlorophenyl)ethyl]benzene"[tw] OR "2 (2 chlorophenyl) 2 (4 chlorophenyl) 1, 1, 1 trichloroethane"[tw] OR "2 (ortho chlorophenyl) 2 (para chlorophenyl) 1, 1, 1 trichloroethane"[tw] OR "2-(o-chlorophenyl)-2-(p-chlorophenyl)-1,1,1-trichloroethane"[tw] OR "2-(2-Chlorophenyl)-2-(4-chlorophenyl)-1,1,1-trichloroethane"[tw] OR "2, 2, 2 trichloro 1 (2 chlorophenyl) 1 (4 chlorophenyl) ethane"[tw] OR "2,4'-DDT"[tw] OR "789-02-6"[tw] OR "D4K93Z1TBH"[tw] OR "o, p ddt"[tw] OR "o, p' DDT"[tw] OR "o, p dichlorodiphenyltrichloroethane"[tw] OR "o,p-DDT"[tw] OR "o,p'-DDT"[tw] OR "o,p'-dichlorodiphenyltrichloroethane"[tw] OR "op ddt"[tw] OR "ortho, para ddt"[tw] OR "ortho, para' ddt"[tw] OR "ortho,para'-DDT"[tw] OR **"Rotenone"[Mesh]** OR "03L9OT429T"[rn] OR "83-79-4"[rn] OR "03L9OT429T"[tw] OR "83-79-4"[tw] OR "Barbasco"[tw] OR "Canex"[tw] OR "Cubor"[tw] OR "Dactinol"[tw] OR "Deril"[tw] OR "Derrin"[tw] OR "Derris root"[tw] OR "Extrax"[tw] OR "Foliafume"[tw] OR "Gerane"[tw] OR "Haiari"[tw] OR "Mexide"[tw] OR "Nekoe"[tw] OR "Nicouline"[tw] OR "Noxfire"[tw] OR "Noxfish"[tw] OR "Nusyn"[tw] OR "Paraderil"[tw] OR "Prenfish"[tw] OR "Prentox"[tw] OR "protax"[tw] OR "Ronone"[tw] OR "Rotacide"[tw] OR "Rotefive"[tw] OR "Rotefour"[tw] OR "Rotenoid"[tw] OR "rotenon"[tw] OR "rotenone"[tw] OR "Rotenox"[tw] OR "Roteonone"[tw] OR "Rotessenol"[tw] OR "Rotocide"[tw] OR "Synpren"[tw] OR "tubatoxin"[tw] OR "Tubotoxin"[tw] OR **"Sucrose"[Mesh:NoExp]** OR "57-50-1"[rn] OR "122880-25-5"[rn] OR "25702-74-3"[rn] OR "92004-84-7"[rn] OR "C151H8M554"[rn] OR "122880-25-5"[tw] OR "1-alpha-D-glucopyranosyl-2-beta-D-fructofuranoside"[tw] OR "25702-74-3"[tw] OR "57-50-1"[tw] OR "92004-84-7"[tw] OR "alpha d glucopyranosyl beta d fructofuranoside"[tw] OR "Amerfand"[tw] OR "Amerfond"[tw] OR "beet sugar"[tw] OR "C151H8M554"[tw] OR "cane sugar"[tw] OR "Granulated sugar"[tw] OR "Microse"[tw] OR "microtal"[tw] OR "Polysucrose"[tw] OR "sacarosa"[tw] OR "saccharose"[tw] OR "Saccharum"[tw] OR "Sacharose"[tw] OR "Sucraloxum"[tw] OR "sucrose"[tw] OR "Sugar spheres"[tw] OR "tabfine"[tw] OR "Table sugar"[tw] OR "White sugar"[tw] OR **"tetrabromobisphenol A" [Supplementary Concept]** OR "FQI02RFC3A"[rn] OR "79-94-7"[rn] OR "79-94-7"[tw] OR "bis(2,3-dibromopropylether)-2,2-bis(3,5-dibromo-4-(2,3-dibromopropoxy)phenyl)propane"[tw] OR "Bromdian"[tw] OR "Firemaster BP4A"[tw] OR "FQI02RFC3A"[tw] OR "TBBPA"[tw] OR "tetrabromo 4, 4' isopropylidenediphenol"[tw] OR "TETRABROMO-4,4'-ISOPROPYLIDENEDIPHENOL"[tw] OR "Tetrabromobisphenol A"[tw] OR "TetrabromobisphenolA"[tw] OR "Tetrabromodian"[tw] OR "Tetrabromodiphenylopropane"[tw]

**AND**

"0001/01/01"[PDat] : "2016/12/31"[PDat]

**2,745 results** – 7/13/18

**Final Mammalian TOXLINE Search Strategy**

Sprague-Dawley OR Wistar OR "Long-Evans" OR Rattus OR rat OR rats OR Oryctolagus OR rabbit OR rabbits

**AND**

embryo* OR fetus* OR foetus* OR fetal* OR foetal*

OR

"ductus arteriosus" OR "endocardial cushion" OR "endocardial cushions" OR "atrioventricular canal cushion" OR "atrioventricular canal cushions" OR "truncus arteriosus" OR organogenesis OR mother* OR dam OR dams OR maternal* OR pregnancy OR pregnancies OR pregnant OR congenital* OR prenatal* OR "pre-natal" OR "pre-natally" OR intrauterine OR "intra-uterine" OR antenatal* OR gestation* OR "transplacental exposure" OR "transplacental exposures"

OR

(("organ development" OR "tooth development" OR "tooth formation" OR "tooth growth" OR "tooth calcification" OR "tooth mineralization" OR "dental development" OR "dental formation" OR cementogenesis OR cementification OR "cementum formation" OR dentinogenesis OR dentinogeneses OR dentification OR "dentin formation" OR dentogenesis OR odontogenesis OR odontogeneses OR amelogenesis OR amelogeneses OR "enamel formation" OR lymphangiogenesis OR lymphangiogeneses OR "musculoskeletal development" OR "musculoskeletal system development" OR "limb development" OR "bone development" OR "bone growth" OR "physiologic calcification" OR "physiological calcification" OR "bone mineralization" OR "maxillofacial development" OR "craniofacial development" OR "face development" OR "facial development" OR "skull development" OR "cranial development" OR "skull growth" OR osteogenesis OR osteogeneses OR "bone formation" OR ossification OR osteoclastogenesis OR osteoclastogeneses OR chondrogenesis OR chondrogeneses OR "muscle development" OR "muscular development" OR myogenesis OR myogeneses OR myofibrillogenesis OR myofibrillogeneses

OR

neurogenesis OR neurogeneses OR "nervous system development" OR "neurologic development" OR "brain development" OR "brain cortex development" OR "cerebral development" OR "brain maturation" OR "brain maturity" OR "eye development" OR "retina development" OR "sex differentiation" OR "sexual differentiation" OR "gonad development" OR "gonadal development" OR "gonad differentiation" OR "gonadal differentiation" OR "sex gland development" OR "sexual gland development" OR "ovary development" OR "ovarian development" OR "ovary maturation" OR "ovarian maturation" OR "follicle development" OR folliculogenesis OR "ovary follicle formation" OR "ovarian follicle formation" OR "follicle maturation" OR "testis development" OR "testicle development" OR "testicular development" OR "testis descent" OR "testicle descent" OR "testicular descent" OR "descensus testiculorum" OR "descensus testis" OR "testis descensus" OR "heart development" OR "cardiac development" OR "heart growth" OR "kidney development" OR "kidney growth" OR "renal development" OR "renal growth" OR "liver development" OR "hepatic development" OR "lung development" OR "pulmonary development")

AND

(embryo* OR fetus* OR foetus* OR fetal* OR foetal*

OR

congenital* OR prenatal* OR "pre-natal" OR "pre-natally" OR intrauterine OR "intra-uterine" OR antenatal* OR gestation*))

**AND**

toxic OR toxical OR toxically OR toxicant OR toxicant's OR toxicants OR toxicants' OR toxicated OR toxication OR toxications OR toxicities OR toxicities' OR toxicity OR toxicity's OR toxico OR toxicochemical OR toxicodynamic OR toxicodynamically OR toxicodynamics OR toxicokinetic OR toxicokinetical OR toxicokinetically OR toxicokinetics OR toxicol OR toxicolethal OR toxicologic OR toxicological OR toxicologically OR toxicologies OR toxicology OR toxicology's OR toxicometric OR toxicometry OR toxicomorphomics OR toxicon OR toxicopathic OR toxicopathies OR toxicopathogenesis OR toxicopathologic OR toxicopathological OR toxicopathology OR toxicopathophysiology OR toxicopathy OR toxicopharmacological OR toxicopharmacology OR toxics OR toxics' OR neurotoxi*

OR

hormesis OR hormeses OR hormetic OR "lethal dose" OR "lethal doses" OR "lethal dosage" OR "lethal dosages" OR "fatal dose" OR "fatal doses" OR "fatal dosage" OR "fatal dosages" OR LD10 OR LD100 OR LD50 OR "L.D.50" OR "LD 50" OR LD90 OR LD95 OR LD99 OR "sublethal dose" OR "sublethal doses" OR "sublethal dosage" OR "sublethal dosages" OR "inhibitory concentration 50" OR IC50 OR "IC-50" OR "50% inhibitory concentration" OR "inhibitory concentration 50%" OR "half maximal inhibitory concentration" OR "half maximum inhibitory concentration" OR "median inhibitory concentration" OR "maximum tolerated dose" OR "maximum tolerated doses" OR "maximum tolerated dosage" OR "maximum tolerated dosages" OR "maximally tolerated dose" OR "maximally tolerated doses" OR "maximally tolerated dosage" OR "maximally tolerated dosages" OR "maximal tolerated dose" OR "maximal tolerated doses" OR "maximal tolerated dosage" OR "maximal tolerated dosages" OR "maximum tolerable dose" OR "maximum tolerable doses" OR "maximum tolerable dosage" OR "maximum tolerable dosages" OR "maximal tolerable dose" OR "maximal tolerable doses" OR "maximal tolerable dosage" OR "maximal tolerable dosages" OR "maximally tolerable dose" OR "maximally tolerable doses" OR "maximally tolerable dosage" OR "maximally tolerable dosages" OR "maximum permissible dose" OR "maximum permissible doses" OR "maximum permissible dosage" OR "maximum permissible dosages" OR "maximal permissible dose" OR "maximal permissible doses" OR "maximal permissible dosage" OR "maximal permissible dosages" OR "maximum permissible exposure level" OR "maximum permissible exposure levels" OR "permissible level" OR "permissible levels" OR "permissible limit" OR "permissible limits" OR "maximum dose" OR "maximum doses" OR "maximum dosage" OR "maximum dosages" OR "maximal dose" OR "maximal doses" OR "maximal dosage" OR "maximal dosages"

OR

"no-observed-adverse-effect level" OR "no-observed-adverse-effect levels" OR "no-observed-adverse-effects level" OR "no-observed-adverse-effects levels" OR NOAEL OR NOAELs OR "no-observed-effect level" OR "no-observed-effect levels" OR "no-observed-effects level" OR "no-observed-effects levels" OR "no observable effect level" OR "no observable effect levels" OR "no observable effects level" OR "no observable effects levels" OR "no effect dose level" OR "no effect dose levels" OR "non-observed effect dose level" OR "non-observed effect dose levels" OR "non-observed-effect level" OR "non-observed-effect levels" OR "no-observed-adverse-event level" OR "no-observed-adverse-event levels" OR "no-observed-adverse-events level" OR "no-observed-adverse-events levels" OR "no-observable-adverse-event level" OR "no-observable-adverse-event levels" OR "no-observable-adverse-events level" OR "no-observable-adverse-events levels"

OR

"protective index" OR "protective indexes" OR "protective indices" OR "therapeutic index" OR "therapeutic indexes" OR "therapeutic indices" OR "safety window" OR "safety windows" OR "therapeutic ratio" OR "therapeutic ratios" OR "therapeutic window" OR "therapeutic windows" OR "therapeutic drug index" OR "therapeutic drug indexes" OR "therapeutic drug indices" OR "therapeutic drug window" OR "therapeutic drug windows" OR "toxic dose" OR "toxic doses" OR "toxic dosage" OR "toxic dosages" OR TD50 OR "body burden" OR "body burdens" OR "drug residue" OR "drug residues" OR "pesticide residue" OR "pesticide residues" OR poison*

OR

teratogen* OR teratolog* OR teratomorph* OR teratotoxic* OR embryotoxi* OR fetotoxi* OR dysmorpholog* OR "drug-induced" OR malform*

OR

(("OECD guideline" OR "OECD guidelines" OR "OECD testing guideline" OR "OECD testing guidelines" OR "test guideline" OR "test guidelines") AND 414) OR "guideline 414" OR "guideline no. 414" OR "guideline number 414" OR "OECD no. 414" OR "OECD number 414" OR "OECD 414" OR "TG 414"

**AND**

**"alitretinoin"** OR "1UA8E65KDZ" OR "5300-03-8" OR "(9cis)-retinoic acid" OR "[3H]9-cis-retinoic acid" OR "9(Z)-Retinoic acid" OR "9-(Z)-retinoic Acid" OR "9-cis-RA" OR "9-cis-Retinoate" OR "9-cis-Retinoic acid" OR "9-cis-Tretinoin" OR "9CRA" OR "9C-RA" OR "9-CRA" OR "9cRA compound" OR "9-Retinoate" OR "9-Retinoic acid" OR "agn 192013" OR "agn192013" OR "ALRT 1057" OR "ALRT1057" OR "BAL4079" OR "BAL-4079" OR "DB00523" OR "LG100057" OR "LG-100057" OR "LGD 100057" OR "LGD 1057" OR "lgd100057" OR "LGD1057" OR "nsc 659772" OR "nsc659772" OR "Panretin" OR "Panretyn" OR "Panrexin" OR "Ro-04-4079" OR "Toctino"

**21 results**, 7/13/18

OR

"5F-uracil" OR **"Fluorouracil"** OR "U3P01618RT" OR "51-21-8" OR "2, 4 dioxo 5 fluoropyrimidine" OR "5 fluoro 2, 4 pyrimidinedione" OR "5 fluoropyrimidine 2, 4 dione" OR "5 fu" OR "5-Faracil" OR "5-florouracil" OR "5-Fluoracil" OR "5-Fluoracyl" OR "5-fluorouacil" OR "5-fluorourasil" OR "5-Fluracil" OR "5-Ftouracyl" OR "5FU" OR "5-HU Hexal" OR "accusite" OR "actino-hermal" OR "Adrucil" OR "agicil" OR "Arumel" OR "Carac" OR "Carzonal" OR "cinkef-u" OR "Effluderm" OR "Efudex" OR "Efudix" OR "Efurix" OR "eurofluor" OR "F 6627" OR "f6627" OR "fivoflu" OR "Fluoro Uracil" OR "Fluoroblastin" OR "Fluoroplex" OR "Fluorouracile" OR "Fluoro-uracile" OR "Fluorouracilo Ferrer Far" OR "Fluoruracil" OR "Fluouracil" OR "fluoxan" OR "flurablastin" OR "Fluracedyl" OR "Fluracil" OR "fluracilium" OR "Fluracilum" OR "Fluri" OR "Fluril" OR "Fluro Uracil" OR "Fluroblastin" OR "fluroblastine" OR "Flurodex" OR "Ftoruracil" OR "Haemato-FU" OR "ifacil" OR "Kecimeton" OR "Neofluor" OR "nsc 18913" OR "nsc18913" OR "NSC19893" OR "NSC-19893" OR "oncofu" OR "Onkofluor" OR "Phthoruracil" OR "Phtoruracil" OR "Queroplex" OR "Ribofluor" OR "ro2 9757" OR "Ro-29757" OR "Ro-2-9757" OR "Timazin" OR "Tolak" OR "uflahex" OR "utoral"

**122 results**, 7/13/18

OR

**"Lovastatin"** OR "9LHU78OQFD" OR "75330-75-5" OR "6alpha-Methylcompactin" OR "6-alpha-Methylcompactin" OR "6-Methylcompactin" OR "Advicor" OR "Altocor" OR "Altoprev" OR "Artein" OR "Belvas" OR "birotin" OR "Cholestra" OR "cid_53232" OR "Closterol" OR "Colevix" OR "cysin" OR "DB00227" OR "ellanco" OR "elstatin" OR "Hipolip" OR "Hipovastin" OR "l 654969" OR "L-154803" OR "Lestatin" OR "Lipdip" OR "Lipivas" OR "Lipofren" OR "Liposcler" OR "lofacol" OR "lomar" OR "lostatin" OR "lovacel" OR "lovacol" OR "lovahexal" OR "Lovalip" OR "Lovalord" OR "lovastan" OR "Lovasterol" OR "Lovastin" OR "lovatadin" OR "lowachol" OR "Lozutin" OR "medostatin" OR "Mevacor" OR "meverstin" OR "Mevinacor" OR "Mevinolin" OR "Mevlor" OR "mk 0803" OR "mk0803" OR "MK803" OR "MK-803" OR "Monacolin K" OR "Monakolin K" OR "msd 803" OR "neolipid" OR "Nergadan" OR "ovasta" OR "Paschol" OR "Rextat" OR "Rodatin" OR "Rovacor" OR "Sivlor" OR "Statosan" OR "Taucor" OR "Tecnolip" OR "Teroltrat"

**15 results**, 7/13/18

OR

"mono 2 ethylhexyl phthalate" OR "2 ethylhexyl phthalate" OR **"mono-(2-ethylhexyl)phthalate"** OR "4376-20-9" OR "FU2EWB60RT" OR "2 ethylhexylphthalate" OR "2-Ethylhexyl hydrogen phthalate" OR "MEHP" OR "mono-ethylhexyl" OR "Monoethylhexyl phthalate" OR "Monoethylhexyl phthalic acid" OR "monoethylhexylphthalate" OR "mono-ethylhexylphthalate" OR "phthalic acid 2 ethylhexyl ester" OR "phthalic acid 2 ethylhexyl monoester" OR "phthalic acid mono (2 ethylhexyl) ester" OR "Phthalic Acid Mono(2-ethylhexyl) Ester" OR "PHTHALIC ACID MONO-2-ETHYLHEXYL ESTER" OR "Phthalic acid mono-2-ethylhexylester" OR "Phthalic acid, mono-(2-ethylhexyl) ester" OR "Phthalic acid, mono-2-ethylhexyl ester" OR "Phthalic Acid-d4 Mono(2-ethylhexyl) Ester"

**215 results**, 7/13/18

OR

"Propoxyphene Compound 65" OR "no doz" OR "Methylxanthine theophylline" OR **"Caffeine"** OR "3G6A5W338E" OR "58-08-2" OR "95789-13-2" OR "1, 3, 7 trimethyl 2, 6 dioxopurine" OR "1,3,7-Trimethyl-2,6-dioxopurine" OR "1,3,7-Trimethylpurine-2,6-dione" OR "1,3,7-trimethylxanthine" OR "1,7-Trimethyl-2,6-dioxopurine" OR "1-methyltheobromine" OR "1-methyl-Theobromine" OR "7-methyl Theophylline" OR "7-Methyltheophylline" OR "Alert-pep" OR "animine" OR "cafalgine" OR "Cafamil" OR "Cafecon" OR "Cafeina" OR "cafeine" OR "Cafergot" OR "Caffedrine" OR "Caffein" OR "Caffeina" OR "Caffeinum" OR "Caffine" OR "Cafipel" OR "coffein" OR "Coffeine" OR "Coffeinum" OR "Darvon compound-65" OR "Dasin" OR "Dexitac" OR "DHCplus" OR "Durvitan" OR "Eldiatric C" OR "Enerjets" OR "Ercatab" OR "Guaranine" OR "guarin" OR "Hycomine" OR "Kofein" OR "Koffein" OR "Lanorinal" OR "Mateina" OR "Methyltheobromide" OR "Methyltheobromine" OR "Nodaca" OR "nodoz" OR "nymusa" OR "Organex" OR "pac compound" OR "Pep-Back" OR "Percoffedrinol N" OR "Percutafeine" OR "peyona" OR "Phensal" OR "Quick Pep" OR "QuickPep" OR "Respia" OR "SK-65 Compound" OR "teina" OR "Theine" OR "Tirend" OR "trimethylxanthine" OR "Vivarin" OR "Wigraine"

**365 results**, 7/13/18

OR

**"Busulfan"** OR "G1LN9045DK" OR "55-98-1" OR "1, 4 bis (methanesulfonyloxy) butane" OR "1, 4 butanediol dimethanesulfonate" OR "1, 4 dimethanesulfonyloxybutane" OR "1, 4 dimethylsulfonyloxybutane" OR "1,4-Bis(methanesulfonoxy)butane" OR "1,4-Bis(methanesulfonyloxy)butane" OR "1,4-Butanedi yl dimethanesulfonate" OR "1,4-BUTANEDIOL DIMETHANESULFONATE" OR "1,4-Butanediol dimethanesulphonate" OR "1,4-Butanediol dimethylsulfonate" OR "1,4-butanedioldimethanesulfonate" OR "1,4-Butanediyl dimethanesulfonate" OR "1,4-Di(methylsulfonoxy)butane" OR "1,4-Dimesyloxybutane" OR "1,4-Dimethane sulfonyl oxybutane" OR "1,4-Dimethanesulfonoxybutane" OR "1,4-Dimethanesulfonoxylbutane" OR "1,4-Dimethanesulfonyloxybutane" OR "1,4-Dimethanesulphonyloxybutane" OR "1,4-Dimethylsulfonoxybutane" OR "1,4-Dimethylsulfonyloxybutane" OR "Bisulfex" OR "Busilvex" OR "Busulfano" OR "Busulfanum" OR "busulfex" OR "busulphan" OR "Busulphane" OR "butane-1,4-diyl dimethanesulfonate" OR "Butanedioldimethanesulfonate" OR "Buzulfan" OR "citosulfan" OR "cytoleukon" OR "glyzophrol" OR "krn 246" OR "krn246" OR "Leucosulfan" OR "mablin" OR "Mielevcin" OR "Mielosan" OR "mielucin" OR "Milecitan" OR "Mileran" OR "misulban" OR "mitistan" OR "mitosan" OR "mitostan" OR "muleran" OR "myelenkon" OR "myeleran" OR "Myeleukon" OR "myeloleukon" OR "Myelosan" OR "Myelosanum" OR "myeloxan" OR "myelucin" OR "mylecitan" OR "Mylecytan" OR "Myleran" OR "Mylerlan" OR "n-Butane-1,3-di(methylsulfonate)" OR "nsc 750" OR "NSC750" OR "Sulfabutin" OR "Sulphabutin" OR "tetramethylene dimesylate"

**84 results**, 7/13/18

OR

"farin" OR "Dethmor" OR "dagonal" OR "coumaphene" OR "coumafene" OR "Coumafen" OR **"Warfarin"** OR "129-06-6" OR "5Q7ZVV76EI" OR "81-81-2" OR "1 (4' hydroxy 3' coumarinyl) 1 phenyl 3 butanone" OR "3 (alpha acetonylbenzyl) 4 hydroxycoumarin" OR "3 acetonylbenzonyl 4 hydroxy coumarinedimethylaminoethanol" OR "3 alpha phenyl beta acetylethyl 4 hydroxycoumarin" OR "3-(.alpha.-Acetonylbenzyl)-4-hydroxycoumarin" OR "3-(.alpha.-Phenyl-.beta.-acetylaethyl)-4-hydroxycumarin" OR "3-(.alpha.-Phenyl-.beta.-acetylethyl)-4-hydroxycoumarin" OR "3-(1'-Phenyl-2'-acetylethyl)-4-hydroxycoumarin" OR "3-(a-acetonylbenzyl)-4-hydroxycoumarin" OR "3-(Acetonylbenzyl)-4-hydroxycoumarin" OR "3-(alpha-Acetonylbenzyl)-4-hydroxycoumarin" OR "3-(alpha-Phenyl-beta-acetylaethyl)-4-hydroxycumarin" OR "3-(alpha-Phenyl-beta-acetylethyl)-4-hydroxycoumarin" OR "4-hydroxy-3-(3-oxo-1-phenylbutyl)-1-benzopyran-2-one" OR "4-Hydroxy-3-(3-oxo-1-phenylbutyl)-2H-1-benzopyran-2-one" OR "acetonylbenzylhydroxycoumarin" OR "adoisine" OR "Aldocumar" OR "antrombin k" OR "Athrombin" OR "athrombine k" OR "athrombinek" OR "befarin" OR "Brumolin" OR "carfin" OR "circuvit" OR "CO-Rax" OR "coumadan" OR "coumadin" OR "coumadine" OR "kumatox" OR "Kypfarin" OR "maforan" OR "marevan" OR "Mar-Frin" OR "Maveran" OR "orfarin" OR "panwarfarin" OR "panwarfin" OR "Prothromadin" OR "Ratorex" OR "Ratox" OR "Ratoxin" OR "Ratron" OR "Rattunal" OR "Rodafarin" OR "Rosex" OR "Sewarin" OR "simarc-2" OR "Sofarin" OR "Solfarin" OR "Sorexa plus" OR "Tedicumar" OR "Temus W" OR "tintorane" OR "uniwarfin" OR "Vampirinip II" OR "Vampirinip iii" OR "wafarin" OR "waran" OR "Warf 10" OR "Warf 42" OR "Warfant" OR "warfar" OR "Warfarat" OR "Warfarina" OR "warfarine" OR "Warfarinum" OR "Warficide" OR "warfil 5" OR "warfilone" OR "warnerin" OR "Zoocoumarin"

**46 results**, 7/13/18

OR

**"Ketoconazole"** OR "R9400W927I" OR "142128-59-4" OR "65277-42-1" OR "1 [4 [4 [ [2 (2, 4 dichlorophenyl) 2 (1h imidazol 1 ylmethyl) 1, 3 dioxolan 4 yl] methoxy] phenyl] 1 piperazinyl] ethanone" OR "1 [4 [4 [ [2 (2, 4 dichlorophenyl) 2 [ (1h imidazol 1 yl) methyl] 1, 3 dioxolan 4 yl] methoxy] phenyl] piperazin 1 yl] ethan 1 one" OR "1 acetyl 4 [4 [ [2 (2, 4 dichlorophenyl) 2 (1h imidazol 1 ylmethyl) 1, 3 dioxolan 4 yl] methoxy] phenyl] piperazine" OR "4 (4 acetylpiperazin 1 yl) alpha [2 (2, 4 dichlorophenyl) 2 imidazol 1 ylmethyl 1, 3 dioxolan 4 yl] anisole" OR "akorazol" OR "anfuhex" OR "antanazol" OR "beatoconazole" OR "bigazol" OR "cetonax" OR "comozol" OR "conazol" OR "cremosan" OR "daktagold" OR "dezoral" OR "dio 902" OR "dio902" OR "extina" OR "formyco" OR "fugen" OR "funazole tabs" OR "funet" OR "fungarest" OR "fungaway" OR "fungazol tabs" OR "fungiderm-k" OR "funginoc" OR "funginox tabs" OR "fungoral" OR "kenazol" OR "kenazole" OR "kesnazol" OR "ketazol" OR "ketocanazole" OR "keto-comp" OR "ketoconazol" OR "Ketoconazolum" OR "keto-crema" OR "ketoderm" OR "ketoisdin" OR "ketomed" OR "ketomicin" OR "ketomicol" OR "ketona" OR "keto-shampoo" OR "ketozal" OR "ketozol" OR "ketozole" OR "kezon" OR "konaturil" OR "Kuric" OR "kw 1414" OR "lusanoc" OR "micoral" OR "mizole" OR "mizoron" OR "mycofebrin" OR "nastil" OR "nazole" OR "neutrogena t/sal" OR "nisoral" OR "niz creme" OR "niz shampoo" OR "nizoral" OR "oxocanazole" OR "oxoconazole" OR "oxonazol" OR "panfungol" OR "pasalen" OR "picamic" OR "prenalon" OR "pristinex" OR "profungal" OR "r 41, 400" OR "R 41,400" OR "r 41400" OR "R41,400" OR "R41400" OR "sebizole" OR "sporium" OR "sporoxyl" OR "sporozol" OR "termizol" OR "terzolin" OR "triatop lotion" OR "Xolegel" OR "zoralin tabs" OR "zorinax"

**33 results**, 7/13/18

OR

"hydroxy carbamide" OR **"Hydroxyurea"** OR "X6Q56QN5QC" OR "127-07-1" OR "(HYDROXYCARBAMOYL)AMINYL" OR "1-oxidanylurea" OR "aminohydroxamic acid" OR "biosupressin" OR "carbamic acid oxime" OR "carbamide oxide" OR "Carbamohydroxamic acid" OR "Carbamohydroximic acid" OR "Carbamohydroxyamic acid" OR "Carbamoyl oxime" OR "Carbamyl hydroxamate" OR "Carbomohydroxamic acid" OR "Carrbamoyl Oxime" OR "Cytodrox" OR "droxia" OR "Hidrix" OR "Hidroxicarbamida" OR "hydab" OR "hydrea" OR "Hydreia" OR "hydrine" OR "Hydroxicarbamidum" OR "hydroxy urea" OR "hydroxyaminomethanamide" OR "Hydroxycarbamid" OR "hydroxycarbamide" OR "Hydroxycarbamidum" OR "Hydroxycarbamine" OR "hydroxyl urea" OR "Hydroxylurea" OR "Hydura" OR "Hydurea" OR "Idrossicarbamide" OR "Litaler" OR "litalir" OR "mylocel" OR "N-(Aminocarbonyl) Hydroxyamine" OR "N-(Aminocarbonyl)hydroxylamine" OR "N-Carbamoylhydroxylamine" OR "neodrea" OR "nsc 32065" OR "NSC32065" OR "oncocarbide" OR "onco-carbide" OR "oxycarbamide" OR "oxyrea" OR "oxyurea" OR "siklos"

**151 results**, 7/13/18

OR

"Dipropyl Acetate" OR **"Valproic Acid"** OR "614OI1Z5WI" OR "99-66-1" OR "2 propylpentanoate" OR "2 propylpentanoic acid" OR "2 propylvalerate sodium" OR "2 propylvaleric acid" OR "2, 2 dipropyl acetic acid" OR "2-propyl-Pentanoate" OR "2-Propylpentanoic Acid" OR "2-PROPYL-PENTANOIC ACID" OR "2-PropylpentanoicAcid" OR "2-Propylvaleric acid" OR "4-Heptanecarboxylic acid" OR "absenor" OR "Acidum valproicum" OR "alpha propylvalerate" OR "alpha propylvaleric acid" OR "apilepsin" OR "atemperator" OR "Avugane" OR "Baceca" OR "convulex" OR "Convulsofin" OR "delepsine" OR "depacon" OR "depakene" OR "depakin" OR "depakine" OR "Depakote" OR "depalept" OR "deprakine" OR "Deproic" OR "di n propylacetate" OR "di n propylacetic acid" OR "di-n-propyl acetic acid" OR "diplexil" OR "dipropyl acetic acid" OR "dipropylacetate" OR "dipropylacetatic acid" OR "dipropylacetic acid" OR "diprosin" OR "Divalproex" OR "Encorate" OR "Epical" OR "epilam" OR "epilex" OR "epilim" OR "episenta" OR "Epival" OR "ergenyl" OR "espa valept" OR "Eurekene" OR "everiden" OR "goilim" OR "hexaquin" OR "kw 6066 n" OR "labazene" OR "leptilan" OR "leptilanil" OR "micropakine" OR "mylproin" OR "myproic acid" OR "n dipropylacetic acid" OR "orfil" OR "orfiril" OR "orlept" OR "petilin" OR "Propylisopropylacetic Acid" OR "Propylvaleric acid" OR "propymal" OR "Savicol" OR "sodium 2 propylpentanoate" OR "sodium 2 propylvalerate" OR "sodium di n propyl acetate" OR "sodium di n propylacetate" OR "sodium dipropyl acetate" OR "sodium dipropylacetate" OR "sodium n dipropylacetate" OR "stavzor" OR "valberg pr" OR "valcote" OR "Valdisoval" OR "valepil" OR "valeptol" OR "valerin" OR "valhel pr" OR "valoin" OR "valpakine" OR "valparin" OR "valporal" OR "valprax" OR "valpro" OR "valproate" OR "valprodura" OR "valprosid" OR "valprotek" OR "valsup" OR "Vupral"

**357 results**, 7/13/18

OR

**"Tretinoin"** OR "5688UTC01R" OR "302-79-4" OR "1 (8 carboxy 3, 7 dimethyl 1, 3, 5, 7 octatetraen 1 yl) 2, 6, 6 trimethyl 1 cyclohexene" OR "3, 7 dimethyl 9 (2, 6, 6 trimethyl 1 cyclohexen 1 yl) 2, 4, 6, 8 nonatetraenoic acid" OR "3, 7 dimethyl 9 (2, 6, 6 trimethyl 1 cyclohexen 1 yl) nona 2, 4, 6, 8 tetraen 1 oic acid" OR "3,7-Dimethyl-9-(2,6,6-trimethyl-1-cyclohexen-1-yl)-2,4,6,8-nonatetraenoic acid" OR "3,7-Dimethyl-9-(2,6,6-trimethyl-1-cyclohexene-1-yl)-2,4,6,8-nonatetraenoic acid" OR "3,7-dimethyl-9-(2,6,6-trimethyl-1-cyclohexenyl)nona-2,4,6,8-tetraenoic acid" OR "3,7-Dimethyl-9-(2,6,6-trimethylcyclohex-1-enyl)nona-2,4,6,8-all-trans-tetraenoic acid" OR "9-cis-RA" OR "Aberel" OR "Aberela" OR "acid a vit" OR "Acnavit" OR "Airol" OR "Aknefug" OR "Aknoten" OR "all-trans-Vitamin A1 acid" OR "alquingel" OR "alten" OR "altinac" OR "anhydroretinoic acid" OR "ar 623" OR "ar623" OR "atra" OR "atragen" OR "atralin" OR "avita" OR "avitcid" OR "Avitoin" OR "betarretin" OR "dermairol" OR "dermik a" OR "effederm" OR "epi aberel" OR "epiaberel" OR "eudyna" OR "facenol" OR "ilotycin-a" OR "locacid" OR "Nexret" OR "nsc 122758" OR "nsc122758" OR "prosome a cream" OR "reacel-a" OR "Refissa" OR "Renova" OR "Retacnyl" OR "retavit" OR "retiderma" OR "Retin A" OR "Retinoate" OR "Retinoic acid" OR "Retinova" OR "Retionic acid" OR "Retisol-A" OR "retrieve cream" OR "ro 01 5488" OR "ro 1 5488" OR "ro 15488" OR "ro015488" OR "ro15488" OR "stieva a" OR "stievaa" OR "tracne" OR "Trans-Retinoicacid" OR "trentin" OR "Tretin M" OR "Tretinoinum" OR "TRETINON" OR "Vesanoid" OR "Vitamin A acid" OR "vitinoin"

**588 results**, 7/13/18

OR

**"Vitamin A"** OR "11103-57-4" OR "68-26-8" OR "3, 7 dimethyl 9 (2, 6, 6 trimethyl 1 cyclohexen 1 yl) 2, 4, 6, 8 nonatetraen 1 ol" OR "3, 7 dimethyl 9 (2, 6, 6 trimethyl 1 cyclohexenyl) 2, 4, 6, 8 nonatetraen 1 ol" OR "3,7-Dimethyl-9-(2,6,6-trimethyl-1-cyclchexen-1-yl)-2,4,6,8-nonatetraen-1-ol" OR "3,7-Dimethyl-9-(2,6,6-trimethyl-1-cyclohexen-1-yl)-2,4,6,8-nonate-traen-1-ol" OR "3,7-Dimethyl-9-(2,6,6-trimethyl-1-cyclohexen-1-yl)-2,4,6,8-nonatetraen-1-ol, (all-E)-" OR "3,7-Dimethyl-9-(2,6,6-trimethyl-1-cyclohexen-1-yl)-2,4,6,8-nonatetraen-1-ol, all (E)-" OR "3,7-Dimethyl-9-(2,6,6-trimethyl-1-cyclohexenyl)-2,4,6,8-nonatetraen-1-ol" OR "3,7-dimethyl-9-(2,6,6-trimethyl-1-cyclohexenyl)-nona-2,4,6,8-tetraen-1-ol" OR "a mulsin" OR "a vitan" OR "a313" OR "acrisina" OR "acrisine" OR "adatone" OR "Afaxin" OR "afaxine" OR "afilina" OR "afiline" OR "agiolan" OR "Agoncal" OR "alfa monovite" OR "alfaergin" OR "alfaergine" OR "alfamin" OR "alfamine" OR "alfamonovit" OR "alfasir" OR "alfasole" OR "alfasterolo" OR "alfatar" OR "alfavena" OR "alfavene" OR "alfavitina" OR "alfavitine" OR "alfene" OR "alin" OR "Alphalin" OR "alphaline" OR "alphasterol" OR "amulsal" OR "amulsin" OR "amulsine" OR "amulvit" OR "Anatola" OR "anavit" OR "Anti-infective vitamin" OR "Antixerophthalmic vitamin" OR "Aoral" OR "apexol" OR "Apostavit" OR "Aquasol A" OR "Aquasola" OR "Aquasynth" OR "asol" OR "asteril" OR "Atars" OR "aterapion" OR "Avibon" OR "avimin" OR "avimine" OR "avipur" OR "avitabiol" OR "avitadit" OR "avital" OR "avitan" OR "A-Vitan" OR "avitana" OR "avitane" OR "avite" OR "avitil" OR "avitina" OR "Avitol" OR "axerophthol" OR "Axerophtholum" OR "axerophthylium" OR "biosterol" OR "Chocola A" OR "Dohyfral A" OR "envit a" OR "gadol" OR "hydrosol" OR "Lard Factor" OR "Oleovitamin A" OR "ophthalamin" OR "Prepalin" OR "prepaline" OR "retinol" OR "Retinolo" OR "Retinolum" OR "Retinyl A" OR "retinyl alcohol" OR "ro a vit" OR "Sehkraft A" OR "Tegosphere VitA" OR "Testavol" OR "Thalasphere" OR "vaconex" OR "Vaflol" OR "Vafol" OR "Veroftal" OR "viadenin" OR "vialpha" OR "Vi-Alpha" OR "viatate" OR "vidoma" OR "vitadone" OR "vitadral" OR "vitalen a" OR "vitalfa" OR "vitama" OR "Vitamin A1" OR "Vitamine A" OR "vitpex" OR "Vogan" OR "xerophthol" OR "Zinosan N"

**1,132 results**, 7/13/18

OR

**"Aminopterin"** OR "JYB41CTM2Q" OR "54-62-6" OR "4 amino 4 deoxyfolic acid" OR "4 amino 4 desoxyfolic acid" OR "4 amino 9 methylpteroylglutamic acid" OR "4 aminofolic acid" OR "4 aminomethylpteroylglutamic acid" OR "4 aminopteroylglutamic acid" OR "4-Amino-4-deoxypteroylglutamate" OR "4-Aminofolate" OR "4-Aminopteroyl- glutamic acid" OR "4-Aminopteroyl-R glutamic acid" OR "4-Aminopteroylglutamate" OR "4-Aminopteroylglutamic acid" OR "4-Aminopteroyl-glutamic acid" OR "Aminopterine" OR "Aminopterinum" OR "Aminotrexate" OR "nsc 739" OR "NSC739" OR "Pteramina"

**22 results**, 7/13/18

OR

**"Methotrexate"** OR "YL5FZ2Y5U1" OR "59-05-2" OR "4 amino 10 methylfolic acid" OR "4 amino 10 methylpteroylglutamic acid" OR "4 amino n10 methylpteroylglutamic acid" OR "4-Aminomethylpteroylglutamic acid" OR "4-amino-N(10)-methylpteroylglutamic acid" OR "4-Amino-N(sup 10)-methylpteroylglutamic acid" OR "4-Amino-N10-methylpteroyl-L-glutamic acid" OR "Abitrexate" OR "amethopterin" OR "A-Methopterin" OR "amethopterine" OR "ametopterine" OR "Antifolan" OR "Arbitrexate" OR "biotrexate" OR "Brimexate" OR "canceren" OR "CL 14377" OR "cl14377" OR "Emtexate" OR "emthexat" OR "emthexate" OR "emtrexate" OR "enthexate" OR "farmitrexat" OR "farmitrexate" OR "farmotrex" OR "Fauldexato" OR "Folex" OR "ifamet" OR "intradose MTX" OR "jylamvo" OR "Lantarel" OR "ledertrexate" OR "Lumexon" OR "maxtrex" OR "Medsatrexate" OR "Metatrexan" OR "metex" OR "methoblastin" OR "methohexate" OR "Methotextrate" OR "methotrate" OR "Methotrexat" OR "methotrexato" OR "Methotrexatum" OR "methoxtrexate" OR "methrotrexate" OR "Methylaminopterin" OR "methylaminopterine" OR "Methylaminopterinum" OR "meticil" OR "metoject" OR "Metolate" OR "metothrexate" OR "Metotressato" OR "metotrexat" OR "metotrexate" OR "metotrexin" OR "metrex" OR "Metrotex" OR "Mexate" OR "mpi 5004" OR "mpi5004" OR "MTX hydrate" OR "N-Bismethylpteroylglutamic acid" OR "neotrexate" OR "nordimet" OR "novatrex" OR "nsc 740" OR "NSC740" OR "Otrexup" OR "rasuvo" OR "reumatrex" OR "Rheumatrex" OR "Texate" OR "texorate" OR "Tremetex" OR "trexall" OR "Trexeron" OR "Trixilem" OR "xaken" OR "Xatmep" OR "zexate"

**132 results**, 7/13/18

OR

"di-hydan" OR **"Phenytoin"** OR "6158TKW0C5" OR "57-41-0" OR "630-93-3" OR "5, 5 diphenylhydantoin" OR "5, 5' diphenylhydantoin" OR "5, 5 diphenylimidazoline 2, 4 dione" OR "5,5-diphenyl hydantoin" OR "5,5-Diphenylhydantoin" OR "5,5-diphenylimidazolidine-2,4-dione" OR "alepsin" OR "aleviatin" OR "antilepsin" OR "Antisacer" OR "Auranile" OR "cansoin" OR "Causoin" OR "Citrullamon" OR "Citrulliamon" OR "Comital" OR "Comitoina" OR "Convul" OR "cumatil" OR "Danten" OR "Dantinal" OR "dantoin" OR "Dantoinal" OR "Dantoine" OR "denyl" OR "Difenin" OR "difetoin" OR "differenin" OR "difhydan" OR "Dihycon" OR "dihydan" OR "Dihydantoin" OR "Dilabid" OR "Dilantin" OR "Dilantine" OR "Dillantin" OR "dintoin" OR "dintoina" OR "Diphantoin" OR "diphantoine" OR "Diphedal" OR "diphedan" OR "Diphenat" OR "Diphenin" OR "Diphenine" OR "Diphentoin" OR "Diphentyn" OR "diphenyl hydantoin" OR "Diphenylan" OR "diphenyldantoin" OR "Diphenylhydantoin" OR "Diphenylhydatanoin" OR "diphenytoin" OR "Di-Phetine" OR "ditoin" OR "Ditoinate" OR "ditomed" OR "Elepsindon" OR "Enkelfel" OR "Epamin" OR "Epanutin" OR "Epdantoin" OR "Epdantoine simple" OR "Epelin" OR "Epifenyl" OR "Epihydan" OR "Epilan D" OR "Epilantin" OR "epileptin" OR "Epinat" OR "Episar" OR "Epised" OR "Epsolin" OR "Eptal" OR "Eptoin" OR "felantin" OR "fenantoin" OR "Fenidantoin s" OR "Fenigramon" OR "Fenitoin" OR "Fentoin" OR "Fenylepsin" OR "fenytoin" OR "Fenytoine" OR "Hidan" OR "hidanil" OR "Hidantal" OR "Hidantilo" OR "Hidantina" OR "Hidantomin" OR "Hindatal" OR "Hydantal" OR "Hydantin" OR "hydantinal" OR "Hydantoinal" OR "Hydantol" OR "Ictalis simple" OR "Idantoil" OR "Idantoin" OR "Iphenylhydantoin" OR "Kessodanten" OR "Labopal" OR "Lehydan" OR "lepitoin" OR "Lepsin" OR "Minetoin" OR "Neos-Hidantoina" OR "Neosidantoina" OR "Oxylan" OR "Phenatine" OR "Phenhydan" OR "phenhydane" OR "Phenitoin" OR "Phentoin" OR "Phentytoin" OR "phenydantin" OR "Phenytoinum" OR "phenytonium" OR "sanepil" OR "sodanton" OR "Sodium Diphenylhydantoinate" OR "solantyl" OR "Zentropil"

**324 results**, 7/13/18

OR

"glycol ethylene" OR "Glycol alcohol" OR "etylene glycol" OR "Ethylene alcohol" OR "ethylen glycol" OR "ethyl glycol" OR **"Ethylene Glycol"** OR "FC72KVT52F" OR "107-21-1" OR "1, 2 ethanediol" OR "1,2-dihydroxy ethane" OR "1,2-Dihydroxyethane" OR "1,2-Ethandiol" OR "1,2-ethane diol" OR "1,2-ethanediol" OR "1,2-ethyleneglycol" OR "2-hydroxyethanol" OR "ethan-1,2-diol" OR "Ethane-1,2-diol" OR "ethane-1.2-diol" OR "ethanediol" OR "Ethylene dihydrate" OR "ethyleneglycol" OR "Ethylenglycol" OR "Etilenglicol" OR "Hypodicarbonous acid" OR "monoethylene glycol"

**334 results**, 7/13/18

OR

"triethylene thiophosphamide" OR "Tri-1-aziridinylphosphine sulfide" OR "tio tef" OR "Tifosyl" OR "Tiofosfamid" OR "triethylenethiophosphamide" OR "thiotriethylenephosphoramide" OR "Thio-Tep" OR "Thiotepum" OR **"Thiotepa"** OR "905Z5W3GKH" OR "52-24-4" OR "AI3-24916" OR "AI324916" OR "Girostan" OR "Ledertepa" OR "methylenethiophosphoramide" OR "n, n', n'' triethylenethiophosphoramide" OR "NSC 6396" OR "nsc 6996" OR "NSC6396" OR "Oncotepa" OR "Oncothio-tepa" OR "oncotiotepa" OR "Phosphoric tri(ethyleneamide)" OR "Phosphorothioic acid triethylenetriamide" OR "PHOSPHOROTHIOIC TRI(ETHYLENEAMIDE)" OR "Stepa" OR "tepadina" OR "tespa" OR "Tespamin" OR "Tespamine" OR "thio tepa" OR "Thiofozil" OR "Thiophosphamide" OR "Thiophosphamidum" OR "Thioplex" OR "Thiotef" OR "triethylene thiophosphoramide" OR "Triethylenethiophosphoramide"

**15 results**, 7/13/18

OR

"Aktikon" OR "Azoprim" OR "Aatrex" OR "Hungazin" OR "Pitezin" OR "Primatol" OR "Primaze" OR "Radazin" OR "Strazine" OR "Weedex A" OR "Wonuk" OR "Zeazin" OR "Zeazine" OR "Fenamin" OR "Fenamine" OR "Fogard" OR "Gesamprim" OR "Gesaprim" OR "6-Chloro-N2-ethyl-N4-isopropyl-1,3,5-triazine-2,4-diamine" OR "2-chloro-4-ethyl-amino-6-isopropylamino-s-triazine" OR "6 chloro n2 ethyl n4 isopropyl 1, 3, 5 triazine 2, 4 diamine" OR **"Atrazine"** OR "2-Chloro-4-(ethylamino)-6-[(prop-2-yl)amino]-1,3,5-triazine" OR "QJA9M5H4IM" OR "1912-24-9" OR "2 chloro 4 ethylamino 6 isopropylamino 1, 3, 5 triazine" OR "2 chloro 4 ethylamino 6 isopropylamino s triazine" OR "2-Chloro-4-(ethylamino)-6-(isopropylamino)-1,3,5-triazine" OR "2-Chloro-4-(ethylamino)-6-(isopropylamino)-s-triazine" OR "2-Chloro-4-(ethylamino)-6-(isopropylamino)triazine" OR "2-Chloro-4-ethylamino-6-isopropylamino-1,3,5-triazine" OR "2-Chloro-4-ethylamino-6-isopropylamino-s-triazine" OR "Aktinit A" OR "Aktinit PK" OR "Atranex" OR "Atrasine" OR "Atrataf" OR "Atrazin" OR "Atred" OR "Atrex"

**95 results**, 7/14/18

OR

**"dinoseb"** OR "88-85-7" OR "2 (1 methylpropyl) 4, 6 dinitrophenol" OR "2 sec butyl 4, 6 dinitrophenol" OR "2-(1-Methylpropyl)-4,6-dinitrophenol" OR "2-(1-Methylpropyl)-4,6-dinitro-Phenol" OR "2-(sec-Butyl)-4,6-dinitrophenol" OR "2, 4 dinitro 6 sec butylphenol" OR "2,4-Dinitro-6-sec-butylphenol" OR "2,4-dinitro-6-sec-butyl-phenol" OR "2-[1-methylpropyl]-4,6-dinitrophenol" OR "2-sec-Butyl-4,6-dinitrophenol" OR "2-Sec-butyl-4,6-dinitro-Phenol" OR "4, 6 dinitro 2 (1 methylpropyl) phenol" OR "4, 6 dinitro 2 sec butylphenol" OR "4,6-Dinitro-2-(1-methyl-n-propyl)phenol" OR "4,6-Dinitro-2-(1-methyl-propyl)phenol" OR "4,6-Dinitro-2-sec-butylphenol" OR "4,6-Dinitro-o-sec-butylphenol" OR "6 sec butyl 2, 4 dinitrophenol" OR "6-sec-Butyl-2,4-dinitrophenol" OR "Aatox" OR "Aretit" OR "Basanite" OR "Blaartox" OR "Butaphene" OR "Caldon" OR "Chemsect" OR "Desicoil" OR "Dibutox" OR "Dinitrall" OR "Dinitrax" OR "dinitrobutylphenol" OR "Dinitro-ortho-sec-butyl phenol" OR "Dinitro-o-sec-butylphenol" OR "Dynanap" OR "Dytop" OR "Elgetol 318" OR "Gebutox" OR "Hivertox" OR "Ivosit" OR "Kiloseb" OR "Ladob" OR "Laseb" OR "Nitropone C" OR "Phenotan" OR "Premerg" OR "Premerge" OR "Sinox general" OR "Sparic" OR "Spurge" OR "Subitex" OR "Tubotox"

**26 results**, 7/14/18

OR

**"fluazinam"** OR "0P91PCK33Q" OR "79622-59-6" OR "3-Chloro-N-(3-chloro-2,6-dinitro-4-(trifluoromethyl)phenyl)-5-(trifluoromethyl)-2-pyridinamine" OR "3-Chloro-N-(3-chloro-2,6-dinitro-4-(trifluoromethyl)phenyl)-5-(trifluoromethyl)pyridin-2-amine" OR "3-chloro-N-(3-chloro-2,6-dinitro-4-trifluoromethylphenyl)-5-trifluoromethyl-2-pyridinamine" OR "3-Chloro-N-(3-chloro-2,6-dinitro-4-trifluoromethylphenyl)-5-trifluoromethyl-2-pyridylamine" OR "3-chloro-N-[3-chloro-2,6-dinitro-4-(trifluoromethyl)phenyl]-5-(trifluoromethyl)-2-pyridinamine" OR "3-chloro-N-[3-chloro-2,6-dinitro-4-(trifluoromethyl)phenyl]-5-(trifluoromethyl)pyridin-2-amine" OR "Altima" OR "Fluaziname" OR "Mapro" OR "Sekoya" OR "Shirlan"

**2 results**, 7/14/18

OR

**"systhane"** OR "B6T1JTM6KZ" OR "88671-89-0" OR "(R)-2-p-chlorophenyl-2-(1H-1,2,4-triazol-1-ylmethyl)hexanenitrile" OR ".alpha.-Butyl-.alpha.-(4-chlorophenyl)-1H-1,2,4-triazole-1-propanenitrile" OR "2 (4 chlorophenyl) 2 (1h 1, 2, 4 triazol 1 ylmethyl) hexanenitrile" OR "2-(4-chlorophenyl)-2-(1,2,4-triazol-1-ylmethyl)hexanenitrile" OR "2-(4-Chlorophenyl)-2-(1H-1,2,4-triazol-1-ylmethyl)hexanenitrile" OR "2-p-Chlorophenyl-2-(1H-1,2,4-triazol-1-ylmethyl)hexanenitrile" OR "alpha butyl alpha (4 chlorophenyl) 1h 1, 2, 4 triazole 1 propanenitrile" OR "alpha-Butyl-alpha-(4-chlorophenyl)-1H-1,2,4-triazole-1-propanenitrile" OR "alpha-n-butyl-alpha(4-chlorophenyl)-1H-1,2,4-triazole-1-propanenitrile" OR "alpha-n-butyl-alpha-(4-chlorophenyl)-1H-1,2,4-triazole-1-propanenitrile" OR "myclobutanil" OR "Synthane 12E"

**7 results**, 7/14/18

OR

**"ochratoxin A"** OR "1779SX6LUY" OR "303-47-9" OR "ochratoxine a"

**95 results**, 7/14/18

OR

**"spiroxamine"** OR "OUT5YHB7BO" OR "118134-30-8" OR "(8-tert-Butyl-1,4-dioxa-spiro[4.5]dec-2-ylmethyl)-ethyl-propyl-amine" OR "8-tert-butyl-1,4-dioxaspiro(4.5)decan-2-ylmethyl(ethyl)(propyl)amine"

**0 results**, 7/14/18

OR

**"thiacloprid"** OR "DSV3A944A4" OR "111988-49-9" OR "(3-((6-Chloro-3-pyridinyl)methyl)-2-thiazolidinylidene)cyanamide" OR "thiaclopride"

**3 results**, 7/14/18

OR

"tetramethyl thiuram disulfide" OR **"Thiram"** OR "0D771IS0FH" OR "137-26-8" OR "16c tetramethylthiuram disulfide" OR "Aapirol" OR "Aatiram" OR "Accel TMT" OR "Aceto TETD" OR "Akrochem TMTD" OR "Anles" OR "Arasan" OR "Atiram" OR "Aules" OR "Basultra" OR "Betoxin" OR "bis (dimethyl thiocarbamoyl) disulfide" OR "bis (dimethylthiocarbamoyl) disulfide" OR "Bis(dimethyl thiocarbamoyl)disulfide" OR "Bis(dimethylthiocarbamoyl) disulfide" OR "Bis(dimethylthiocarbamoyl) disulphide" OR "Bis(dimethylthiocarbamyl) disulfide" OR "Cunitex" OR "Cyuram DS" OR "Delsan" OR "Ekagom TB" OR "Falitiram" OR "Fermide" OR "Fernacol" OR "Fernasan" OR "Fernide" OR "Formalsol" OR "Granuflo" OR "Hermal" OR "Hexathir" OR "Kregasan" OR "Mercuram" OR "Methyl thiuramdisulfide" OR "Methyl tuads" OR "Methylthiuram disulfide" OR "Metiur" OR "Metiurac" OR "Nobecutan" OR "Nomersan" OR "Normersan" OR "NSC 1771" OR "NSC1771" OR "Panoram 75" OR "Polyram ultra" OR "Pomarsol" OR "Pomasol" OR "puralin" OR "Radothiram" OR "Rezifilm" OR "rhenogran" OR "Robac TMT" OR "Sadoplon" OR "Spotrete" OR "Sranan-sf-X" OR "Teramethylthiuram disulfide" OR "Tersan" OR "Tetramethyl thiuramdisulfide" OR "Tetramethyl thiurane disulfide" OR "Tetramethyl thiurane disulphide" OR "Tetramethyldiurane sulphite" OR "Tetramethylenethiuram disulfide" OR "Tetramethylenethiuram disulphide" OR "Tetramethylthiocarbamoyldisulphide" OR "Tetramethylthioperoxydicarbonic diamide" OR "Tetramethylthioramdisulfide" OR "Tetramethylthiouram disulfide" OR "Tetramethylthiuram" OR "tetramethylthiuramdisulfide" OR "tetramethylthiuramidisulfide" OR "Tetramethylthiuran disulphide" OR "Tetramethylthiurane disulfide" OR "Tetramethylthiurum disulfide" OR "Tetramethylthiurum disulphide" OR "Tetrapom" OR "Tetrasipton" OR "tetrathion" OR "tetrathione" OR "tetrathionine" OR "Tetrathiuram disulfide" OR "Tetrathiuram disulphide" OR "Thianosan" OR "Thillate" OR "Thimar" OR "Thimer" OR "thiosan" OR "Thioscabin" OR "Thiotex" OR "Thiotox" OR "Thiramad" OR "Thirampa" OR "Thiramum" OR "Thirasan" OR "Thiulin" OR "Thiulix" OR "Thiurad" OR "Thiuram" OR "Thiuramin" OR "thiuramyl" OR "Thylate" OR "Tiradin" OR "tiram" OR "tiramo" OR "Tirampa" OR "tiuramyl" OR "TMT Disulfide" OR "TMTD" OR "TMTDS" OR "Trametan" OR "Tridipam" OR "Tripomol" OR "tuad" OR "TUEX" OR "Tulisan" OR "Tutan" OR "Tyradin"

**47 results**, 7/14/18

OR

**"triadimefon"** OR "43121-43-3" OR "1 (4 chlorophenoxy) 3, 3 dimethyl 1 (1, 2, 4 triazol 1 yl) 2 butanone" OR "1-(1,2,4-triazolyl)-1-(4-chlorophenoxy)-3,3-dimethylbutan-2-one" OR "1-(1,2,4-Triazoyl-1)-1-(4-chloro-phenoxy)-3,3-dimethylbutanone" OR "1-(4-chlorophenoxy)-3,3-dimethyl-1-(1,2,4-triazol-1-yl)butan-2-one" OR "1-(4-Chlorophenoxy)-3,3-dimethyl-1-(1,2,4-triazol-1-yl)-butan-2-one" OR "1-(4-Chlorophenoxy)-3,3-dimethyl-1-(1,2,4-triazol-1-yl)butanone" OR "1-(4-chlorophenoxy)-3,3-dimethyl-1-(1H-1,2,4-triazol-1-yl) butan-2-one" OR "1-(4-Chlorophenoxy)-3,3-dimethyl-1-(1H-1,2,4-triazol-1-yl)-2-butanone" OR "1-(4-chlorophenoxy)-3,3-dimethyl-1-(1H-1,2,4-triazol-1-yl)butan-2-one" OR "1-(4-Chlorophenoxy)-3,3-dimethyl-1-(1H-1,2,4-triazole -1-yl)-2-butanone" OR "1-(4-Chloro-phenoxy)-3,3-dimethyl-1-[1,2,4]triazol-1-yl-butan-2-one" OR "Acizol" OR "Adifon" OR "Amiral" OR "Azocene" OR "Bayleton" OR "Diametom B" OR "Fenxiunin" OR "Haleton" OR "Miltek" OR "Nurex" OR "Otria 25" OR "Rofon" OR "Tidifon" OR "Triadimefone" OR "Triadimeform" OR "triadimenol" OR "Tripinacloraz"

**38 results**, 7/14/18

OR

**"flusilazole"** OR "F3WG2VVD87" OR "85509-19-9" OR "Benocarp" OR "DPX 6573" OR "dpx h6573" OR "DPX-H 6573" OR "dpxh6573" OR "DPX-N 6573" OR "DPX-N6573" OR "Flusilazol" OR "Fluzilazol" OR "Nustar" OR "Olymp" OR "PPX-H6573"

**18 results**, 7/14/18

OR

**"hexaconazole"** OR "SX9R3X1FQV" OR "79983-71-4" OR "2 (2, 4 dichlorophenyl) 1 (1h 1, 2, 4 triazol 1 yl) 2 hexanol" OR "2-(2,4-dichlorophenyl)-1-(1,2,4-triazol-1-yl)-2-hexanol" OR "2-(2,4-dichlorophenyl)-1-(1,2,4-triazol-1-yl)hexan-2-ol" OR "2-(2,4-dichlorophenyl)-1-(1H-1,2,4-triazol-1-yl)hexan-2-ol" OR "alpha butyl alpha (2, 4 dichlorophenyl) 1h 1, 2, 4 triazole 1 ethanol" OR "Canvil" OR "Chlortriafol" OR "Clortriafol" OR "Contaf" OR "Flowmax 5SC" OR "Hexaconazol" OR "Ranvil"

**2 results**, 7/14/18

OR

**"propiconazole"** OR "142KW8TBSR" OR "60207-90-1" OR "1-[[2-(2,4-dichlorophenyl)-4-propyl-1,3-dioxolan-2-yl]methyl]-1,2,4-triazole" OR "1-[[2-(2,4-Dichlorophenyl)-4-propyl-1,3-dioxolan-2-yl]methyl]-1H-1,2,4-triazole" OR "1-[2-(2,4-Dichloro-phenyl)-4-propyl-[1,3]dioxolan-2-ylmethyl]-1H-[1,2,4]triazole" OR "1-[2-(2,4-dichlorophenyl)-4-propyl-1,3-dioxolan-2-ylmethyl]-1H-1,2,4-triazole" OR "1-{[2-(2,4-dichlorophenyl)-4-propyl-1,3-dioxolan-2-yl]methyl}-1H-1,2,4-triazole" OR "Bamper" OR "cga 64250" OR "cga64250" OR "Desmel" OR "Proconazole" OR "Propiconazol" OR "Propyconazol" OR "Wocosen" OR "Wocosin 50TK"

**10 results**, 7/14/18

OR

**"Endosulfan"** OR "OKA6A6ZD4K" OR "115-29-7" OR "5, 6 bis (hydroxymethyl) 1, 2, 3, 4, 7, 7 hexachloronorbornene sulfite" OR "5, 6 bis (hydroxymethyl) 1, 2, 3, 4, 7, 7 hexachloronorbornenesulfite" OR "5, 6 bis (hydroxymethyl) hexachloronorcamphene sulfite" OR "alpha endosulfane" OR "benzoepin" OR "beosit" OR "beta endosulfane" OR "chlorothiepine" OR "chlorthiapinum" OR "chlorthiepin" OR "Chlortiepin" OR "Crisulfan" OR "cyclodan" OR "devisulfan" OR "Devisulphan" OR "Endocel" OR "endogan" OR "Endosol" OR "Endosulphan" OR "Endotaf" OR "ensawan" OR "FMC 5462" OR "fmc5462" OR "Hildan" OR "Insectophene" OR "malix" OR "Rasayansulfan" OR "Sialan" OR "thifor" OR "thimul" OR "Thiodan" OR "Thiodon" OR "thiofor" OR "thiomul" OR "Thionate" OR "thionex" OR "Thiosulfan" OR "Thiotox" OR "thyodan" OR "thyonex" OR "tiodan" OR "tionel" OR "Tionex" OR "Tiovel"

**55 results**, 7/14/18

OR

**"pyridaben"** OR "2E4JBA5272" OR "96489-71-3" OR "2-tert-Butyl-5-(4-tert-Butylbenzylthio)-4-chloropyridazin-3(2H)-one" OR "2-tert-Butyl-5-(4-tert-butyl-benzylthio)-4-chloropyridazin-3(2H)-one" OR "2-tert-butyl-5-[(4-tert-butylbenzyl)thio]-4-chloropyridazin-3(2H)-one" OR "2-tert-butyl-5-[(4-tert-butylbenzyl)thio]-4-chloro-pyridazin-3-one" OR "Damanlin" OR "Nexter" OR "Pyramite" OR "Sanmite"

**0 results**, 7/14/18

OR

"wood alcohol" OR "MetOH" OR **"Methanol"** OR "67-56-1" OR "Y4S76JWI15" OR "carbinol" OR "Carbonal" OR "hydroxymethan" OR "Hydroxymethane" OR "MeOH" OR "Methoxy Group" OR "methyl alcohol" OR "Methyl hydrate" OR "Methyl hydroxide" OR "Methylalcohol" OR "Methylic alcohol" OR "Methylol" OR "Monohydroxymethane" OR "monomethylol" OR "wood spirit"

**197 results**, 7/14/18

OR

**"cyproconazole"** OR "94361-06-5" OR "622B9C3E6T" OR "Atemi" OR "Cyproconazol"

**6 results**, 7/14/18

OR

**"triticonazole"** OR "131983-72-7" OR "(1RS)-(E)-5-((4-chlorophenyl)methylene)-2,2-dimethyl-1-(1H-1,2,4-triazol-1-ylmethyl)cyclopentan-1-ol"

**1 result**, 7/14/18

OR

**"diniconazole"** OR "X82HVO1N83" OR "76714-16-4" OR "83657-24-3" OR "1-(2,4-dichlorophenyl)-4,4-dimethyl-2-(1,2,4-triazol-1-yl)-1-penten-3-ol" OR "1-(2,4-Dichlorophenyl)-4,4-dimethyl-2-(1H-1,2,4-triazol-1-yl)pent-1-en-3-ol" OR "Diclopentezol" OR "S 3308" OR "S-3308L"

**0 results**, 7/14/18

OR

"AZT" OR **"Zidovudine"** OR "4B9XT59T7S" OR "30516-87-1" OR "3' azido 2', 3' dideoxyribosylthymine" OR "3' azido 2', 3' dideoxythymidine" OR "3' Azido 2',3' Dideoxythymidine" OR "3' Azido 3' deoxythymidine" OR "3' azido 3' deoxythymidine" OR "3' azidothymidine" OR "3'azido-3'deoxythymidine" OR "3'-azido3'-deoxythymidine" OR "3-Azido-3-deoxythymidine" OR "3'-Azido-3'deoxythymidine" OR "adovi" OR "avirzid" OR "azidodeoxythymidine" OR "azidomine" OR "Azidothymidine" OR "Azitidin" OR "bio zt" OR "BW A509U" OR "BWA509U" OR "BWA-509U" OR "Dendrigen" OR "pranadox" OR "retrocar" OR "Retrovir" OR "Retrovis" OR "ZDV" OR "zidis" OR "zidovir" OR "zidovudin" OR "Zidovudinum" OR "zudovidine" OR "zydowin"

**53 results**, 7/14/18

OR

**"Metoclopramide"** OR "L4YEB44I46" OR "364-62-5" OR "4-amino-5-chloro-N-[2-(diethylamino)-ethyl]-2-methoxybenzamide" OR "4 amino 5 chloro n (2 diethylaminoethyl) 2 methoxybenzamide" OR "4 amino 5 chloro n (2 diethylaminoethyl) o anisamide" OR "4 amino 5 chloro n (2 diethylaminoethyl) ortho anisamide" OR "4 Amino-5-chloro-N-(2-(diethylamino)ethyl)-2-methoxybenzamide" OR "4-Amino-5-chloro-N-(2-(diethylamino)ethyl)-o-anisamide" OR "4-Amino-5-chloro-N-(2-diethylamino-ethyl)-2-methoxy-benzamide" OR "5 chloro 2 methoxyprocainamide" OR "ahr 3070 c" OR "ahr 3070c" OR "ahr3070c" OR "ametic" OR "anausin" OR "apo-metoclop" OR "aputern" OR "betaclopramide" OR "carnotprim primperan" OR "Cerucal" OR "clodilion" OR "clopamon" OR "clopan" OR "Clopra" OR "clopram" OR "Clopromate" OR "Degan" OR "del 1267" OR "del1267" OR "dibertil" OR "Duraclamid" OR "Elieten" OR "emenil" OR "emetal" OR "emetard" OR "Emetid" OR "Emitasol" OR "emperal" OR "encil" OR "enzimar" OR "Eucil" OR "gastro timelets" OR "gastrobi" OR "Gastrobid" OR "Gastromax" OR "Gastronerton" OR "gastrosil" OR "Gastrotablinen" OR "gastrotem" OR "gastrotimelets" OR "gavistal" OR "gensil" OR "Gimoli" OR "hemesis" OR "hyrin" OR "imperan" OR "m 813" OR "m813" OR "maril" OR "Maxeran" OR "maxeron" OR "maxolan" OR "Maxolon" OR "mcp-beta tropfen" OR "meclomid" OR "meclopamide" OR "meclopramide" OR "Meclopran" OR "Megaldrate" OR "meramide" OR "Metaclopramide" OR "Metadrate" OR "metagliz" OR "metamide" OR "Methochlopramide" OR "methoclopramide" OR "methoclopramine" OR "Methoxychlorprocainamide" OR "metlazel" OR "Metochlopramide" OR "Metochloropramide" OR "Metoclol" OR "metoclopamide" OR "metoclopramid" OR "Metoclopramidum" OR "metoclopramine" OR "metoclopranide hydrochloride" OR "metoclor" OR "metoclorpramide" OR "metocobil" OR "metocyl" OR "metodopramide" OR "metolon" OR "metopram" OR "metox" OR "metozolv" OR "metpamid" OR "metram" OR "Metramid" OR "Moriperan" OR "mygdalon" OR "nausil" OR "neopramiel" OR "netaf" OR "nilatika" OR "normastin" OR "Octamide" OR "opram" OR "Parmid" OR "Paspertin" OR "Peraprin" OR "perinorm" OR "pharmyork" OR "Plasil" OR "pramidin" OR "Pramiel" OR "pramin" OR "pramotel" OR "Primperan" OR "primperil" OR "prinparl" OR "prokinyl lp" OR "prowel" OR "pulin" OR "Pylomid" OR "Reclomide" OR "Regla" OR "Reglan" OR "Reliveran" OR "rimetin" OR "sensamide"

**7 results**, 7/14/18

OR

**"Acebutolol"** OR "67P356D8GH" OR "37517-30-9" OR "1 (2 acetyl 4 butyramidophenoxy) 2 hydroxy 3 isopropylaminopropane" OR "1-(2-Acetyl-4-n-butyramidophenoxy)-2-hydroxy-3-isopropylaminopropane" OR "Acebrutololum" OR "Acebutololo" OR "Acebutololum" OR "acecor" OR "ApoAcebutolol" OR "bay c 7705" OR "bay c7705" OR "diasectral" OR "espesil" OR "flebutol" OR "grifobutol" OR "il 17803a" OR "il17803a" OR "M & B 17803A" OR "M and B 17803A" OR "Monitan" OR "Neptal" OR "neptall" OR "NovoAcebutolol" OR "Prent" OR "Rhotral" OR "Sectral" OR "Wesfalin"

**2 results**, 7/14/18

OR

"Isonicotinyl hydrazide" OR "isonicotinic acid hydrazine" OR "Isonicotinic acid hydrazide" OR **"Isoniazid"** OR "isozine" OR "isozone" OR "izoniazid" OR "Isonicotinoylhydrazine" OR "Isozide" OR "Laniazid" OR "V83O1VOZ8L" OR "54-85-3" OR "4 pyridinecarbohydrazide" OR "4 pyridinecarboxylic acid hydrazide" OR "Abdizide" OR "Andrazide" OR "Anidrasona" OR "antimic" OR "Antimicina" OR "antimicine" OR "Antituberkulosum" OR "Armacide" OR "Armazid" OR "Armazide" OR "atcotibin" OR "Atcotibine" OR "Azuren" OR "bacillen" OR "Bacillin" OR "Cedin" OR "Cemidon" OR "Chemiazid" OR "Chemidon" OR "Continazine" OR "Cortinazine" OR "Cotinazin" OR "cotinazine" OR "Cotinizin" OR "cotinizine" OR "curazid forte" OR "Defonin" OR "dianicotyl" OR "diazid" OR "Dibutin" OR "Diforin" OR "Dinacrin" OR "Ditubin" OR "Ebidene" OR "Eralon" OR "eralone" OR "Ertuban" OR "Eutizon" OR "eutizone" OR "Evalon" OR "Fetefu" OR "Fimalene" OR "fimaline" OR "FRS-3" OR "fsr3" OR "Ftivazide" OR "GINK" OR "hain" OR "hiconyl" OR "Hid rasonil" OR "hidraciber" OR "Hidranizil" OR "Hidrasonil" OR "hidrazida" OR "Hidrulta" OR "hidrulte" OR "Hidrun" OR "Hycozid" OR "hycozide" OR "hydrasonil" OR "Hydrazid" OR "Hydrazide" OR "hydrazin" OR "Hyozid" OR "Hyzyd" OR "ido tebin" OR "Idrazil" OR "Inah" OR "inh burgthal" OR "INHd20" OR "Inizid" OR "inizide" OR "Iscotin" OR "iscotine" OR "Isidrina" OR "isidrine" OR "Ismazide" OR "Isobicina" OR "isobicine" OR "Isocid" OR "Isocidene" OR "isoco tin" OR "Isocotin" OR "isocotine" OR "Isohydrazide" OR "Isokin" OR "Isolyn" OR "isolyne" OR "isomazide" OR "isomerina" OR "Isonerit" OR "Isonex" OR "isoniac" OR "Isoniacid" OR "Isoniazide" OR "isoniazidine" OR "Isoniazidum" OR "isoniazone" OR "isonicazid" OR "Isonicazide" OR "Isonicid" OR "isonicide" OR "Isonico" OR "Isonicotan" OR "isonicotane" OR "isonicotic acid hydrazide" OR "Isonicotil" OR "isonicotinate hydrazide" OR "Isonicotinhydrazid" OR "isonicotinic acid hydrazone" OR "Isonicotinic Acid Vanillylidenehydrazide" OR "Isonicotinic hydrazide" OR "isonicotinicacid hydrazide" OR "Isonicotinohydrazide" OR "Isonicotinoyl hydrazide" OR "isonicotinoylhydrazide" OR "Isonicotinyl hydrazine" OR "Isonicotinylhydrazide" OR "Isonicotinylhydrazine" OR "Isonide" OR "Isonidrin" OR "isonidrine" OR "Isonikazid" OR "isonikazide" OR "Isonizide" OR "isopharmide" OR "Isotamine" OR "Isotebe" OR "Isotebezid" OR "isotebezide"

**42 results**, 7/14/18

OR

"pyridine 4 carbohydrazide" OR "pyridine-4-carboxylic acid hydrazide" OR "Neoteben" OR "neotebene" OR "Neo-Tizide" OR "Neoxin" OR "neoxine" OR "neoxon" OR "neoxone" OR "Neumandin" OR "Nevin" OR "Niadrin" OR "nicatibine" OR "nicazid" OR "Nicazide" OR "Nicetal" OR "Nicizina" OR "nicodrin" OR "Niconyl" OR "nicosciorin" OR "nicotibin" OR "Nicotibina" OR "Nicotibine" OR "Nicotisan" OR "nicotubin" OR "nicotubine" OR "nicozid" OR "Nicozide" OR "nicozyd" OR "Nidaton" OR "Nidrazid" OR "nidrazide" OR "Nikozid" OR "nikozide" OR "niosciorine" OR "Niplen" OR "Nitadon" OR "Niteban" OR "Nitebannsc 9659" OR "nortibina" OR "nortibine" OR "Nydrazid" OR "nydrazide" OR "Nyscozid" OR "nyscozide" OR "Pelazid" OR "pelazide" OR "Percin" OR "Phthisen" OR "Phthivazid" OR "Phthivazide" OR "puran" OR "Pycazide" OR "Pyreazid" OR "pyreazide" OR "Pyricidin" OR "pyricidine" OR "Pyridicin" OR "Pyrizidin" OR "pyrizidine" OR "ramnanon" OR "Raumanon" OR "Razide" OR "Retozide" OR "rhymicid" OR "Rifater" OR "Rimicid" OR "rimicide" OR "Rimifon" OR "Rimiphone" OR "Rimitsid" OR "Robiselin" OR "Robisellin" OR "robiselline" OR "Roxifen" OR "roxyfen miquel" OR "RP 5015" OR "Sanohidrazina" OR "sanohydrazina" OR "sanohydrazine" OR "santerazid" OR "santerazide" OR "Sauterazid" OR "Sauterzid" OR "Stanozide" OR "supercidin" OR "tb phlogin" OR "Tebecid" OR "tebecide" OR "tebecin" OR "tebecine" OR "tebemid" OR "Tebenic" OR "tebesium" OR "tebetracin" OR "tebetracine" OR "Tebexin" OR "tebexine" OR "Tebilon" OR "tebilone" OR "Tebos" OR "Teebaconin" OR "teebaconine" OR "Tekazin" OR "tekazine" OR "thiocevit" OR "Tibazide" OR "Tibemid" OR "tibemide" OR "Tibiazide" OR "Tibinide" OR "Tibison" OR "tibisone" OR "tibitan" OR "tibitane" OR "Tibivis" OR "Tibizide" OR "Tibusan" OR "tibusane" OR "Tisin" OR "Tisiodrazida" OR "tisiodrazide" OR "tisiotrazida" OR "Tizide" OR "Tubazid" OR "Tubazide" OR "Tubeco" OR "Tubecotubercid" OR "tubercid" OR "Tuberian" OR "tuberiane" OR "Tubicon" OR "tubicone" OR "Tubilysin" OR "Tubizid" OR "Tubomel" OR "tubonil" OR "tubylisin" OR "tubylisine" OR "tyrid" OR "Tyvid" OR "tyvide" OR "Unicocyde" OR "Unicozyde" OR "valifol" OR "Vazadrine" OR "Vederon" OR "vederone" OR "vitazide" OR "yuhan-zid" OR "Zidafimia" OR "Zinadon" OR "zinadone" OR "Zonazide"

**3 results**, 7/14/18

OR

**"Saccharin"** OR "FST467XS7D" OR "81-07-2" OR "128-44-9" OR "1, 2 benzisothiazol 3 (2h) one 1, 1 dioxide" OR "1, 2 benzisothiazol 3 one 1, 1 dioxide" OR "1, 2 dihydro 2 ketobenzisosulfonazole" OR "1, 2-Benzisothiazol-3(2H)-one, 1,1-dioxide" OR "1,2-Benzisothiazol-3(2H)-one 1,1-dioxide" OR "1,2-Benzisothiazol-3(2H)-one, 1,1-dioxide" OR "1,2-Dihydro-2-ketobenzisosulfonazole" OR "1,2-Dihydro-2-ketobenzisosulphonazole" OR "2, 3 dihydro 3 oxobenzisosulfonazole" OR "2,3-Dihydro-3-oxobenzisosulfonazole" OR "2,3-Dihydro-3-oxo-Benzisosulfonazole" OR "2,3-Dihydro-3-oxobenzisosulphonazole" OR "2-Sulfobenzoic acid imide" OR "2-Sulfobenzoic imide" OR "2-Sulfobenzoicimide" OR "2-Sulphobenzoic imide" OR "Anhydro-o-sulfaminebenzoic acid" OR "Benzo-2-sulfiide" OR "Benzo-2-sulphimide" OR "Benzoic acid sulfimide" OR "Benzoic sulfimide" OR "Benzoic sulphimide" OR "Benzosulfimide" OR "Benzosulfinide" OR "Benzosulphimide" OR "Benzo-sulphinide" OR "Benzoylsulfonic Imide" OR "Cristallose" OR "Crystallose" OR "Garantose" OR "Glucid" OR "glucide" OR "Gluside" OR "Glycophenol" OR "Hermesetas" OR "Kandiset" OR "Kristallose" OR "Madhurin" OR "Natreen" OR "Neosaccharin" OR "o-Benzoic acid sulfimide" OR "o-Benzoic sulfimide" OR "o-Benzoic sulphimide" OR "o-Benzosulfimide" OR "o-Benzosulphimide" OR "o-Benzoyl sulfimide" OR "o-Benzoyl sulphimide" OR "O-Benzoylsulfimide" OR "ortho sulfobenzimide" OR "ortho sulfobenzoic acid imide" OR "Ortho sulphobenzamide" OR "o-Sulfobenzimide" OR "o-Sulfobenzoic acid imide" OR "O-Sulfobenzoic imide" OR "o-Sulfonbenzoic acid imide sodium salt" OR "Sacarina" OR "Saccharimide" OR "Saccharina" OR "Saccharine" OR "Saccharinol" OR "Saccharinose" OR "saccharoid" OR "Saccharol" OR "Saxin" OR "Sodium saccharide" OR "Sucrette" OR "Sucromat" OR "sweet n low" OR "Sweeta" OR "sweetex" OR "sweetnin" OR "Sykose" OR "Syncal" OR "Willosetten" OR "Zaharina"

**132 results**, 7/14/18

OR

**"Penicillin G"** OR "Benzylpenicillin sodium" OR "YS5LY7JF4N" OR "69-57-8" OR "1406-05-9" OR "American penicillin" OR "Benpen" OR "Coliriocilina" OR "Crystapen" OR "Kesso-Pen" OR "Mycofarm" OR "Novocillin" OR "Parcillin" OR "Pekamin" OR "Pengesod" OR "Penibiot" OR "Penicilina G Llorente" OR "Penicillin Grünenthal" OR "penicillin ii sodium" OR "penicillin sodium" OR "penicilline g sodium" OR "PenicillinGsodiumsalt" OR "Penilaryn" OR "Penilevel" OR "Peniroger" OR "Pfizerpen" OR "Sodiopen" OR "Sodipen" OR "Sodium 6-(phenylacetamido)penicillanate" OR "sodium benzyl penicillin" OR "Sodium benzylpenicillin" OR "Sodium benzylpenicillinate" OR "Sodium penicillin" OR "sodium penicilline g" OR "Sugracillin sodium salt" OR "Unicilina" OR "Ursopen" OR "Veticillin"

**21 results**, 7/14/18

OR

**"Thalidomide"** OR "4Z8R6ORS6L" OR "50-35-1" OR ".alpha.-Phthalimidoglutarimide" OR "3-Phthalimidoglutarimide" OR "Algosediv" OR "alpha-Phthalimidoglutarimide" OR "Asidon 3" OR "Asmadion" OR "Asmaval" OR "Bonbrain" OR "Calmore" OR "Calmorex" OR "Celgene" OR "Contergan" OR "Corronarobetin" OR "Distaval" OR "Distaxal" OR "Distoval" OR "Ectiluran" OR "Enterosediv" OR "Gastrinide" OR "Glupan" OR "Glutanon" OR "Grippex" OR "Hippuzon" OR "Imidene" OR "Isomin" OR "Kedavon" OR "Kevadon" OR "n (2, 6 dioxopiperid 3 yl) phthalimide" OR "N-(2,6-dioxo-3-piperidinyl)phthalimide" OR "N-(2,6-Dioxo-3-piperidyl)phthalimide" OR "n-(2,6-dioxopiperidin-3-yl)phthalimide" OR "Neaufatin" OR "Neosedyn" OR "Neosydyn" OR "Nerosedyn" OR "Neufatin" OR "Neurodyn" OR "Neurosedin" OR "Neurosedym" OR "Neurosedyn" OR "neurosedyne" OR "Nevrodyn" OR "Nibrol" OR "Noctosediv" OR "Noxodyn" OR "N-Phthalimidoglutamic acid imide" OR "N-Phthaloylglutamimide" OR "N-Phthalylglutamic acid imide" OR "nsc 66847" OR "NSC66847" OR "Pangul" OR "Pantosediv" OR "Pharmion" OR "Polygripan" OR "Pro-Bam M" OR "Pro-ban M" OR "Profarmil" OR "Quetimid" OR "Quietoplex" OR "Sandormin" OR "Sedalis" OR "Sedimide" OR "Sedin" OR "Sedisperil" OR "Sedoval" OR "shin naito" OR "Shinnibrol" OR "Sleepan" OR "Slipro" OR "Softenil" OR "Softenon" OR "Synovir" OR "Talargan" OR "Talidomide" OR "Talimol" OR "Talinol" OR "Talismol" OR "Talizer" OR "Telagan" OR "Telargan" OR "Telargean" OR "Tensival" OR "thado" OR "Thaled" OR "thalidomid" OR "Thalidomidum" OR "Thalin" OR "Thalinette" OR "thalix" OR "Thalomid" OR "Thalomide" OR "Theophilcholine" OR "Valgis" OR "Valgraine" OR "Yodomin"

**299 results**, 7/14/18

OR

**"Doxylamine"** OR "95QB77JKPL" OR "469-21-6" OR "562-10-7" OR ".alpha.-Dimethylaminoethoxyphenylmethyl-2-picoline" OR "2-Dimethylaminoethoxyphenylmethyl-2-picoline" OR "alsadorm" OR "decapryn" OR "deoxylamine succinate" OR "Diclectin" OR "Dolased" OR "donormyl" OR "dormidina" OR "Dossilamina" OR "doxilamina" OR "doxylaminesuccinate" OR "doxy-sleep-aid" OR "Dozile" OR "Evanorm" OR "gittalun" OR "histadoxylamine" OR "hoggar" OR "mereprine" OR "Mersyndol" OR "Restavit" OR "sedaplus" OR "Somnil" OR "Syndol" OR "Unisom" OR "vicks nyquil"

**9 results**, 7/14/18

OR

**"Diphenhydramine"** OR "8GTS82S83M" OR "58-73-1" OR "147-24-0" OR "beta dimethylaminoethyl benzhydryl ether" OR ".beta.-(Dimethylamino)ethyl benzhydryl ether" OR ".beta.-Dimethylamino-aethyl-benzhydryl-aether" OR "2 (benzhydroloxy) n, n dimethylethylamine" OR "2 (diphenylmethoxy) n, n dimethyl ethylamine" OR "2 (diphenylmethoxy) n, n dimethylethylamine" OR "2 benzhydryloxy n, n dimethylethylamine" OR "2 diphenylmethoxy n, n dimethylethylamine" OR "2-(Benzhydryloxy)-N,N-dimethylethanamine" OR "2-(Benzhydryloxy)-N,N-dimethylethylamine" OR "2-(Benzohydryloxy)-N,N-dimethylethylamine" OR "2-(diphenylmethoxy)-N,N-dimethylethanamine" OR "2-(Diphenylmethoxy)-N,N-dimethylethylamine" OR "2-(diphenylmethyl)oxy-N,N-dimethylethanamine" OR "2-(diphenylmethyl)oxy-N,N-dimethyl-ethanamine" OR "2-[(diphenylmethyl)oxy]-N,N-dimethylethanamine" OR "2-[di(phenyl)methoxy]-N,N-dimethylethanamine" OR "2-benzhydryloxy-N,N-dimethylethanamine" OR "2-benzhydryloxy-N,N-dimethyl-ethanamine" OR "2-diphenylmethoxy-N,N-demthylethanamine" OR "2-Diphenylmethoxy-N,N-dimethylethylamine" OR "Aleryl" OR "Alledryl" OR "Allerdryl" OR "Allergan" OR "Allergeval" OR "Allergical" OR "Allergina" OR "Allergival" OR "Amidryl" OR "Antistominum" OR "Antomin" OR "Automin" OR "Bagodryl" OR "banaril" OR "Banophen" OR "Baramine" OR "Beldin" OR "Belix" OR "Benachlor" OR "benadril" OR "Benadrin" OR "Benadryl" OR "benadyl" OR "Ben-allergin" OR "Benapon" OR "Benhydramin" OR "benocten" OR "Benodin" OR "Benodine" OR "Benylan" OR "Benylin" OR "Benzantine" OR "Benzhydramine" OR "Benzhydraminum" OR "Benzhydroamina" OR "Betramin" OR "caladryl" OR "carphenamine" OR "carphenex" OR "cathejell" OR "Compoz" OR "Dabylen" OR "Debendrin" OR "Dermistina" OR "Dermodrin" OR "Desentol" OR "Diabenyl" OR "Diabylen" OR "Dibendrin" OR "Dibenil" OR "Dibondrin" OR "dibrondrin" OR "Difedryl" OR "Difenhydramin" OR "Difenhydramine" OR "Dihidral" OR "Dimedrol" OR "Dimedryl" OR "Dimehydrinate" OR "Diphantine" OR "diphedryl" OR "Diphen" OR "diphenacen" OR "diphendramine" OR "diphenhydramide" OR "diphenhydramin" OR "Diphenhydraminum" OR "diphenydramine" OR "Diphenylhydramin" OR "Diphenylhydramine" OR "Dobacen" OR "Dormarex 2" OR "Dormin" OR "Hydramine" OR "nytol" OR "sediat" OR "sleepeze" OR "sominex" OR "vertirosan" OR "vicks formula 44"

**42 results**, 7/14/18

OR

**"clopyralid"** OR "10G14M0WDH" OR "1702-17-6" OR "3,6-Dichloropicolinic acid" OR "Benzalox" OR "Cirtoxin" OR "Cliophar" OR "Clopiralid" OR "Clopyralide" OR "Cyronal" OR "dichloropyridine acid" OR "Dowco 290" OR "Huiloralid" OR "Loncid" OR "Lontrel" OR "Matrigon" OR "Transline" OR "Versatill"

**2 results**, 7/14/18

OR

**"Camphor"** OR "76-22-2" OR "21368-68-3" OR "2 bornanone" OR "2 camphanone" OR "2 oxobornane" OR "2-Camphanone" OR "2-Camphonone" OR "Alcanfor" OR "Alphanon" OR "Bornan-2-one" OR "camphora" OR "korodin" OR "Root bark oil" OR "Root bark spirit" OR "sarna"

**27 results**, 7/14/18

OR

**"fipronil"** OR "QGH063955F" OR "120068-37-3" OR "5 amino 1 (2, 6 dichloro alpha, alpha, alpha trifluoro para tolyl) 4 trifluoromethylsulfinylpyrazole 3 carbonitrile" OR "5 amino 1 [2, 6 dichloro 4 (trifluoromethyl) phenyl] 4 [ (trifluoromethyl) sulfinyl] 1h pyrazole 3 carbonitrile" OR "5-amino-1 -(2,6-dichloro-4-trifluoromethylphenyl)-4-trifluoromethylsulfinyl-1 H-pyrazole-3-carbonitrile" OR "5-amino-1-(2,6-dichloro-4-(trifluoromethyl) phenyl)-4-((trifluoromethyl) sulfinyl)-1H-pyrazol-3-carbonitrile" OR "5-amino-1-(2,6-dichloro-4-(trifluoromethyl)phenyl)-4-((trifluoromethyl)sulfinyl)-1h-pyrazole-3-carbonitrile" OR "5-amino-1-(2,6-dichloro-alpha,alpha,alpha-trifluoro-p-tolyl)-4-trifluoromethylsulfinylpyrazole-3-carbonitile" OR "5-Amino-1-[2,6-dichloro-4-(trifluoromethyl)phenyl]-4-(trifluoromethyl)sulfinylpyrazole-3-carbonitrile" OR "5-amino-1-[2,6-dichloro-4-(trifluoromethyl)phenyl]-4-(trifluoromethylsulfinyl)pyrazole-3-carbonitrile" OR "5-amino-1-[2,6-dichloro-4-(trifluoromethyl)phenyl]-4-[(trifluoromethyl)sulfinyl]-1H-pyrazole-3-carbonitrile" OR "5-amino-1-[2,6-dichloro-4-(trifluoromethyl)phenyl]-4-trifluoromethanesulfinyl-1H-pyrazole-3-carbonitrile" OR "fiprex" OR "Fluocyanobenpyrazole" OR "Frontline Spot-on" OR "Frontline Spray" OR "Frontline Top Spot" OR "Goliath gel" OR "Granedo MC" OR "Maxforce FC" OR "Termidor"

**7 results**, 7/14/18

OR

**"Glycerol"** OR "PDC6A3C0OX" OR "56-81-5" OR "107283-02-3" OR "144086-02-2" OR "144086-03-3" OR "25618-55-7" OR "8013-25-0" OR "1, 2, 3 propanetriol" OR "1, 2, 3 trihydroxypropane" OR "1,2,3-Propanetriol" OR "1,2,3-triglycerol" OR "1,2,3-TRIHYDROXYPROPAN-2-YL" OR "1,2,3-trihydroxypropane" OR "1,2,3-trihydroxypropanol" OR "1,3-Propanetriol" OR "1,3-Trihydroxypropane" OR "alditol" OR "Artificial tears" OR "Bulbold" OR "Citifluor AF 2" OR "Cristal" OR "Dagralax" OR "Glicerina" OR "Glyceol" OR "glycerin" OR "Glycerine" OR "Glycerinum" OR "Glyceritol" OR "Glycerolum" OR "Glycyl alcohol" OR "Glyrol" OR "Glysanin" OR "Glyzerin" OR "Grocolene" OR "microglycerin" OR "Monoctanoin Component D" OR "Neutracett" OR "Oelsuess" OR "Olsuss" OR "Ophthalgan" OR "Osmoglyn" OR "Polyglycerin" OR "Polyglycerine" OR "Polyglycerol" OR "Pricerine 9091" OR "PROPANE-1,2,3-TRIOL" OR "Propanetriol" OR "Tegin M" OR "Trihydroxypropane" OR "Tryhydroxypropane" OR "vilardell" OR "Vitrosupos"

**95 results**, 7/14/18

OR

**"hexazinone"** OR "51235-04-2" OR "Y51727MR1Y" OR "Gridball" OR "Hexazinoe" OR "Hexazinon" OR "Velpar"

**2 results**, 7/15/18

OR

**"imazamox"** OR "UG6793ON5F" OR "114311-32-9"

**0 results**, 7/15/18

OR

**"imazapyr"** OR "81334-34-1" OR "2-(4-Isopropyl-4-methyl-5-oxo-2-imidazolin-2-yl)nicotinic acid"

**0 results**, 7/15/18

OR

**"Loratadine"** OR "7AJO3BO7QN" OR "79794-75-5" OR "Aerotina" OR "Alarin" OR "Alavert" OR "alerfast" OR "alernitis" OR "Alerpriv" OR "alertadin" OR "alertrin" OR "allerta" OR "Allertidin" OR "allertyn" OR "allohex" OR "ambrace" OR "analergal" OR "Anhissen" OR "anlos" OR "ardin" OR "Bactimicina allergy" OR "Bedix Loratadina" OR "Biloina" OR "Bonalerg" OR "caradine" OR "carin" OR "Civeran" OR "clalodine" OR "Claratyne" OR "clarid" OR "Clarinase" OR "Claritin" OR "Claritine" OR "Clarityn" OR "Clarityne" OR "Clarium" OR "cronitin" OR "Cronopen" OR "curyken" OR "demazin anti-allergy" OR "Ethyl-4-(8-chloro-5,6-dihydro-11H-benzo [5,6] cyclohepta [1,2-b]pyridin-11-ylidene)-1-piperidinecarboxylate" OR "ezasmin" OR "ezede" OR "finska" OR "Flonidan" OR "frenaler" OR "Fristamin" OR "genadine" OR "halodin" OR "hislorex" OR "histalor" OR "Histaloran" OR "j-tadine" OR "klarihist" OR "Klaritin" OR "klinset" OR "laredine" OR "lergia" OR "Lergy" OR "Lertamine" OR "Lesidas" OR "lindine" OR "Lisino" OR "lisono" OR "lobeta" OR "lodain" OR "Lomilan" OR "lorabasics" OR "Loracert" OR "loraclar" OR "loraderm" OR "Loradex" OR "Loradif" OR "loradin" OR "lorahist" OR "loralerg" OR "lora-lich" OR "lorano" OR "Loranox" OR "Lorantis" OR "lorapaed" OR "Lorastine" OR "lora-tabs" OR "Loratadinum" OR "loratadura" OR "loratan" OR "loratazine" OR "loratidin" OR "Loratidine" OR "loraton" OR "loratrim" OR "Loratyne" OR "Loraver" OR "loraxin" OR "loreen" OR "Lorfast" OR "lorihis" OR "lorita" OR "Loritine" OR "lotadine" OR "lotarin" OR "Lowadina" OR "mosedin" OR "noratin" OR "notamin" OR "Nularef" OR "onemin" OR "Optimin" OR "Polaratyne" OR "proactin" OR "Restamine" OR "Rhinase" OR "ridamin" OR "rihest" OR "rinityn" OR "Rinolan" OR "Rinomex" OR "rityne" OR "Roletra" OR "Sanelor" OR "Sch 29851" OR "Sch29851" OR "Sensibit" OR "Sinhistan Dy" OR "Sohotin" OR "Symphoral" OR "Tadine" OR "Talorat Dy" OR "tidilor" OR "tirlor" OR "toradine" OR "Velodan" OR "Versal" OR "voratadine" OR "Zeos"

**12 results**, 7/15/18

OR

**"novaluron"** OR "Z8H1B3CW0B" OR "116714-46-6" OR "1-(3-chloro-4-(1,1,2-trifluoro-2-trifluoromethoxyethoxy)phenyl)-3-(2,6-difluorobenzoyl)urea" OR "Rimon EC-10"

**0 results**, 7/15/18

OR

**"2-phenylphenol"** OR "2 biphenylol" OR "Orthophenyl phenol" OR "90-43-7" OR "61788-42-9" OR "D343Z75HT8" OR "2 hydroxydiphenyl" OR "2 phenylphenol" OR "2-Biphenylol" OR "2-hydroxy biphenyl" OR "2-Hydroxy-1,1'-biphenyl" OR "2-Hydroxybiphenyl" OR "2-Hydroxydiphenyl" OR "2-Phenyl phenol" OR "Amocid" OR "Anthrapole 73" OR "Biphenyl-2-ol" OR "Biphenylol" OR "Dowicide" OR "Hydroxy-2-phenylbenzene" OR "Hydroxybiphenyl" OR "Lyorthol" OR "o-Biphenylol" OR "o-Diphenylol" OR "o-Hydroxybiphenyl" OR "o-Hydroxydiphenyl" OR "o-Phenyl phenol" OR "o-phenylphenate" OR "o-phenylphenol" OR "ortho hydroxybiphenyl" OR "ortho hydroxydiphenyl" OR "ortho phenylphenol" OR "Orthohydroxydiphenyl" OR "ortho-phenylphenate" OR "Orthophenylphenol" OR "Orthoxenol" OR "o-Xenol" OR "o-Xonal" OR "Preventol 3041" OR "Preventol O extra" OR "Remol TRF" OR "Rotoline" OR "sodium o-phenylphenoate" OR "Stellisept" OR "Tetrosin oe" OR "Torsite" OR "Xenol"

**25 results**, 7/15/18

OR

**"Propylene Glycol"** OR "6DC9Q167V3" OR "57-55-6" OR "123120-98-9" OR "63625-56-9" OR "1, 2 dihydroxypropane" OR "1, 2 propandiol" OR "1, 2 propanediol" OR "1, 2 propylenglycol" OR "1, 2-propanediol" OR "1,2 Propanediol" OR "1,2-(RS)-Propanediol" OR "1,2-dihydroxypropan-2-yl" OR "1,2-dihydroxypropane" OR "1,2-Dihydroxypropanl" OR "1,2-propandiol" OR "1,2-propane diol" OR "1.2-propanediol" OR "2,3-Propanediol" OR "2-Hydroxypropanol" OR "Aliphatic alcohol" OR "apopropanediol" OR "Ilexan P" OR "Isopropylene glycol" OR "methyl ethyl glycol" OR "methyl glycol" OR "Methylethyl glycol" OR "Methylethylene glycol" OR "methylglycol" OR "Monopropylene glycol" OR "Prolugen" OR "propan 1, 2 diol" OR "Propan-1,2-Diol" OR "propane 1, 2 diol" OR "propane-1,2-diol" OR "propanediol" OR "propyleneglycol" OR "propylenglycol" OR "Sirlene" OR "Trimethyl glycol"

**112 results**, 7/15/18

OR

**"triclopyr"** OR "MV06PHJ6I0" OR "55335-06-3" OR "3,5,6-TPA" OR "((3,5,6-trichloro-2-pyridinyl)oxy)-acetic acid" OR "[(3,5,6-trichloro-2-pyridinyl)oxy]-acetic acid" OR "3,5,6-Trichloro-2-pyridinyloxyacetic Acid" OR "((3,5,6-trichloro-2-pyridyl)oxy)-acetic acid" OR "3,5,6-Trichloro-2-pyridyloxyacetic acid" OR "Garlon" OR "Grazon ET" OR "Trichlopyr" OR "Turflon"

**8 results**, 7/15/18

OR

**"triethylene glycol"** OR "112-27-6" OR "103734-98-1" OR "122784-99-0" OR "137800-98-7" OR "145112-98-7" OR "3P5SU53360" OR "2,2'-(Ethylenedioxy)diethanol" OR "2,2-(Ethylenedioxy)diethanol" OR "2,2'-Ethylenedioxybis(ethanol)" OR "2,2'-Ethylenedioxydiethanol" OR "3, 6 dioxaoctane 1, 8 diol" OR "3,6-Dioxa-1,8-octanediol" OR "3,6-Dioxaoctane-1,8-diol" OR "Bis(2-hydroxyethoxyethane)" OR "Di-.beta.-hydroxyethoxyethane" OR "Di-beta-hydroxyethoxyethane" OR "Ethylene glycol dihydroxydiethyl ether" OR "Ethylene glycol-bis-(2-hydroxyethyl ether)" OR "Glycol bis(hydroxyethyl) ether" OR "Tri-ethylene glycol" OR "Triethyleneglycol" OR "triethylenglycol" OR "Trigenos" OR "Triglycol" OR "Trigol"

**36 results**, 7/15/18

OR

**"zoxamide"** OR "156052-68-5" OR "RH 7281" OR "RH7281" OR "Zoxamid" OR "Zoxium"

**0 results**, 7/15/18

OR

**"pyriproxyfen"** OR "3Q9VOR705O" OR "95737-68-1" OR "126040-81-1" OR "2 [1 methyl 2 (4 phenoxyphenoxy) ethoxy] pyridine" OR "2-(1-Methyl-2-(4-phenoxyphenoxy)ethoxy)pyridine" OR "2-[ 1-methyl-2-(4-phenoxyphenoxy)ethoxy]pyridine" OR "2-[1-methyl-2-(4-phenoxyphenoxy)ethoxy] pyridine" OR "2-[1-Methyl-2-(4-phenoxyphenoxy)ethoxy]pyridine" OR "4-Phenoxyphenyl (RS)-2-(2-pyridyloxy)propyl ether" OR "Archer IGR" OR "Cyclio" OR "juvinal" OR "NyGuard IGR" OR "Nylar" OR "Pyriproxifen" OR "S 31183" OR "S31183" OR "Sumilarv"

**4 results**, 7/15/18

OR

"2-methoxyethanol" OR **"methyl cellosolve"** OR "2-methoxy ethanol" OR "2methoxyethanol" OR "109-86-4" OR "9004-74-4" OR "95507-80-5" OR "EK1L6XWI56" OR "Dowanol 7" OR "Dowanol EM" OR "Ektasolve EM" OR "Ethylene glycol methyl ether" OR "Ethylene glycol monomethyl ether" OR "ethylene glycol monomethylether" OR "Ethyleneglycol monomethyl ether" OR "ethyleneglycol monomethylether" OR "ethylglycol monomethyl ether" OR "Glycol ether EM" OR "Glycol monomethyl ether" OR "Glycolmethyl ether" OR "Jeffersol EM" OR "Karl Fischer Reagent" OR "Methyl ethoxol" OR "Methyl icinol" OR "Methyl oxitol" OR "methylcellosolve" OR "methylcello-solve" OR "Monoethylene glycol methyl ether" OR "Monomethyl ether of ethylene glycol" OR "Monomethyl ethylene glycol ether" OR "Monomethyl glycol" OR "O-Methyl Glycol"

**277 results**, 7/15/18

OR

**"Acetaminophen"** OR "362O9ITL9D" OR "103-90-2" OR "4 hydroxyacetanilide" OR "4' hydroxyacetanilide" OR "4-(Acetylamino)phenol" OR "4-(N-Acetylamino)phenol" OR "4-acetamido phenol" OR "4-ACETAMIDOPHENYLOXIDANYL" OR "Abenol" OR "Abensanil" OR "Abrol" OR "Abrolet" OR "Acamol" OR "Acenol" OR "Acephen" OR "Acertol" OR "Acetaco" OR "Acetagesic" OR "Acetalgin" OR "Acetamidophenol" OR "acetamino phenol" OR "Acetaminofen" OR "acetaminophene" OR "acetaminophenol" OR "Acetamol" OR "Acetofen" OR "acetomenophen" OR "Acetominophen" OR "acetominophene" OR "acetylaminophenol" OR "acetyl-p-aminophenol" OR "Actamin" OR "Actimol" OR "adorem" OR "Afebrin" OR "Afebryl" OR "Aferadol" OR "Algesidal" OR "algiafin" OR "Algina" OR "algocit" OR "Algomol" OR "Algotropyl" OR "Alvedon" OR "Amadil" OR "Aminofen" OR "anadin" OR "Anaflon" OR "analgiser" OR "Analter" OR "Andox" OR "Anelix" OR "Anuphen" OR "Apacet" OR "Apadon" OR "Apamid" OR "Apamide" OR "APAP" OR "apirex" OR "Apitrelal" OR "apotel" OR "Arthralgen" OR "Asetam" OR "Asomal" OR "Asplin" OR "atamel" OR "Atasol" OR "Atralidon" OR "Bacetamol" OR "benuron" OR "Ben-u-ron" OR "Biocetamol" OR "biogesic" OR "Bucet" OR "Calapol" OR "Calonal" OR "Calpol" OR "Causalon" OR "Cefalex" OR "cemol" OR "Cetadol" OR "claradol" OR "Claratal" OR "Clixodyne" OR "clocephen" OR "Codabrol" OR "Codalgin" OR "Codapane" OR "Codicet" OR "Codoliprane" OR "Cofamol" OR "Conacetol" OR "cp 500" OR "cp500" OR "Cuponol" OR "Curadon" OR "Dafalgan" OR "Daphalgan" OR "Darocet" OR "Darvocet" OR "Datril" OR "Deminofen" OR "Democyl" OR "Demogripal" OR "depyretin" OR "Desfebre" OR "Dimindol" OR "Dirox" OR "dismifen" OR "Disprol" OR "dolal" OR "Doliprane" OR "dolofen" OR "Dolofugin" OR "dolomol" OR "dolorol" OR "Dolprone" OR "doltem" OR "Dresan" OR "Dularin" OR "Duorol" OR "Duracetamol" OR "Durapan" OR "Dymadon" OR "Ecosetol" OR "efferalgan" OR "efferelgan" OR "Empracet" OR "Endecon" OR "Enelfa" OR "Eneril" OR "eraldor" OR "eu med" OR "exopon" OR "Fanalgic" OR "Farmadol" OR "Febranine" OR "Febrectol" OR "Febrex" OR "Febricet" OR "Febridol" OR "Febrilix" OR "Febrinol" OR "Febrolin" OR "Fepanil" OR "fibrinol" OR "Finimal" OR "fortolin" OR "Gelocatil" OR "Geluprane" OR "Grippostad" OR "Hedex" OR "helporal" OR "Homoolan" OR "Hydroxyacetanilide" OR "Inalgex" OR "kamolas" OR "Kataprin"

**99 results**, 7/15/18

OR

"p-Hydroxyacetanilide" OR "N-Acetyl-p-aminophenol" OR "n acetyl para aminophenol" OR "n acetyl 4 aminophenol" OR "Labamol" OR "Lekadol" OR "Lemsip" OR "letamol" OR "liquiprin" OR "medamol" OR "Mexalen" OR "Midol" OR "Minafen" OR "minopan" OR "Miralgin" OR "Napafen" OR "napamol" OR "NAPAP" OR "naprex" OR "Naprinol" OR "neodalmin" OR "Neodol" OR "nilapur" OR "Noral" OR "Norco" OR "nysacetol" OR "Ofirmev" OR "Oltyl" OR "Oralgan" OR "Ortensan" OR "Oxycocet" OR "Pacemo" OR "Pacet" OR "p-Acetamidophenol" OR "p-Acetaminophenol" OR "p-Acetoaminophen" OR "p-Acetylaminophenol" OR "Pacimol" OR "Paedol" OR "Pamol" OR "Panacete" OR "Panadeine" OR "Panadol" OR "Panamax" OR "Panasorb" OR "Panex" OR "Panodil" OR "Panofen" OR "Pantalgin" OR "para acetamidophenol" OR "para acetylaminophenol" OR "para hydroxyacetanilide" OR "para suppo" OR "Paracemol" OR "Paracenol" OR "Paracet" OR "paracetaminophenol" OR "Paracetamol" OR "Paracetamole" OR "Paracetamolum" OR "Paracetanol" OR "Paracetol" OR "Paracin" OR "Paracod" OR "Paracodol" OR "Parador" OR "parageniol" OR "Parakapton" OR "Paralen" OR "paramax" OR "paramidol" OR "Paramol" OR "Paramolan" OR "Paranox" OR "Parapan" OR "Parasedol" OR "Parasin" OR "Paraspen" OR "Parcetol" OR "Parelan" OR "Parmol" OR "Parogal" OR "Paroma" OR "Pasolind" OR "paximol" OR "Pediapirin" OR "Pediatrix" OR "pedipan" OR "Perfalgan" OR "Phenaphen" OR "Phendon" OR "Phenipirin" OR "Phogoglandin" OR "Phrenilin" OR "p-hydroxyacetoanilide" OR "p-Hydroxyphenolacetamide" OR "Pinex" OR "Piramin" OR "Pirinasol" OR "Plicet" OR "polarfen" OR "Polmofen" OR "Predimol" OR "Prodol" OR "Prontina" OR "Propacet" OR "Pulmofen" OR "Pyrinazine" OR "Pyromed" OR "raperon" OR "relaphen" OR "Reliv" OR "Remedol" OR "Resfenol" OR "Resprin" OR "revanin" OR "Rivalgyl" OR "Rubophen" OR "Salzone" OR "Sanicet" OR "Sanicopyrine" OR "Scanol" OR "Semolacin" OR "Setakop" OR "Setamol" OR "Setol" OR "Sifenol" OR "Sinaspril" OR "Sinedol" OR "Sine-Off" OR "Sinmol" OR "sinpro" OR "Sunetheton" OR "Supadol mono" OR "Supofen" OR "Tabalgin" OR "tachipirin" OR "tachipirina" OR "taganopain" OR "Talacen" OR "Tapanol" OR "Tapar" OR "Tazamol" OR "Tempanal" OR "Tempra" OR "Tencon" OR "Termacet" OR "Termalgin" OR "Termalgine" OR "Termofren" OR "Titralgan" OR "tramil" OR "Treuphadol" OR "Triaprin" OR "Tricoton" OR "turpan" OR "Tycolet" OR "Tylenol" OR "Tylex" OR "Tylox" OR "Tymol" OR "Valadol" OR "Vicodin"

**28 results**, 7/15/18

OR

**"Ascorbic Acid"** OR "PQ6CK8PD0R" OR "50-81-7" OR "53262-66-1" OR "1-Xyloascorbic Acid" OR "Acidum ascorbicum" OR "acidylina" OR "adenex" OR "agrumina" OR "allercorb" OR "allescorb" OR "arcavit c" OR "arcavite c" OR "arkovital c" OR "ascelat" OR "ascofar" OR "Ascoltin" OR "ascomed" OR "asconvita" OR "ascor" OR "ascorbate" OR "ascorbicap" OR "ascorbicin" OR "ascorbico" OR "ascorbin" OR "ascorbina" OR "ascorbinic acid" OR "ascorbit" OR "ascorbite" OR "ascorbitol" OR "ascorbone" OR "ascorbutina" OR "ascorbyl" OR "ascorbyn" OR "ascorgil" OR "ascorin" OR "ascormin" OR "ascorteal" OR "ascorval" OR "ascorvel" OR "ascorvitina" OR "c vit" OR "c vita" OR "cantan" OR "cantaxin" OR "catavin c" OR "ce vi sol" OR "cebetate" OR "cebicure" OR "Cebid" OR "cebion" OR "cebione" OR "cecon" OR "cecorbyl" OR "cecorbyle" OR "cecrisina" OR "cedon" OR "cedone" OR "cedoxon" OR "cedoxone" OR "cegiolan" OR "Ceklin" OR "celaskon" OR "celaskone" OR "celin" OR "Cemagyl" OR "cenetone" OR "cenol" OR "cenolate" OR "cequinyl" OR "cereon" OR "cergona" OR "cescorbat" OR "cetamican" OR "cetamid" OR "cetamine" OR "cetebe" OR "cevalin" OR "cevaline" OR "cevatine" OR "cevex" OR "cevibid" OR "cevibram" OR "cevigal" OR "cevigen" OR "cevigol" OR "cevilat" OR "cevisol" OR "ce-vi-sol" OR "cevit" OR "cevita" OR "Cevital" OR "cevitamic acid" OR "cevitan" OR "cevite" OR "cevitex" OR "cevitil" OR "cevitol" OR "Chromagen" OR "ciergin" OR "cisir" OR "citamino" OR "citoascorbina" OR "citoxyl" OR "citran" OR "citravite" OR "Citrovit" OR "civitin" OR "civitine" OR "concemin" OR "cortalex" OR "Duoscorb" OR "erftamin c" OR "erftamine c" OR "esuron" OR "godabion c" OR "gregovite c" OR "hicee" OR "hybrin" OR "inovitan c" OR "Juvamine" OR "laroscorbine" OR "lemascorb" OR "Magnorbin" OR "myascorbin" OR "nybadol" OR "Parentrovite" OR "parkovit c" OR "pharmascorbine" OR "proscorbin" OR "proscorbine" OR "redoxon" OR "ribena" OR "Rovimix C" OR "scorbacid" OR "scorbacide" OR "scorbex" OR "scorbitol" OR "secorbate" OR "Semidehydroascorbate" OR "sevalin" OR "Sunkist" OR "vicef" OR "vicelat" OR "vicetrin" OR "viciman" OR "vicin" OR "vicitina" OR "vicon" OR "vitace" OR "vitacimine" OR "vitacin" OR "vitacine" OR "vitamin C" OR "Vitamisin" OR "vitapur c" OR "vitascorbin" OR "xyloascorbic acid"

**189 results**, 7/15/18

OR

**"butylparaben"** OR "3QPI1U3FV8" OR "94-26-8" OR "4 hydroxybenzoic acid butyl ester" OR "4-(Butoxycarbonyl)phenol" OR "4-Hydroxybenzoic acid-n-butyl ester" OR "Aseptoform butyl" OR "butoben" OR "butyl 4 hydroxybenzoate" OR "butyl butex" OR "butyl hydroxybenzoic acid" OR "butyl para hydroxybenzoate" OR "Butyl paraben" OR "butyl parabenbutyl parahydroxybenzoatebutyl p-hydroxybenzoate" OR "Butyl parahydroxybenzoate" OR "Butyl p-hydroxybenzoate" OR "butylhydroxybenzoate" OR "Nipabutyl" OR "para hydroxybenzoic acid butyl ester" OR "p-Hydroxy butyl benzoate" OR "p-Hydroxybenzoic acid butyl ester" OR "p-Hydroxybenzoic acid n-butyl ester" OR "p-Hydroxybenzoic butyl ester"

**12 results**, 7/15/18

OR

**"Carbamazepine"** OR "33CM23913M" OR "298-46-4" OR "5H-Dibenz[ b, f]azepine-5-carboxamide" OR "5H-Dibenz[b,f]azepine-5-carboxamide" OR "5H-Dibenzo[b,f]azepine-5-carboxamide" OR "amizepin" OR "amizepine" OR "apo-carbamazepine" OR "atretol" OR "Bipotrol" OR "biston" OR "calepsin" OR "camapine" OR "carbadac" OR "Carbamazepen" OR "carbamazepin" OR "Carbamazepinum" OR "Carbamezepine" OR "carbategral" OR "carbatol" OR "carbatrol" OR "carbazene" OR "carbazep" OR "Carbazepin" OR "Carbazepine" OR "carbazina" OR "Carbelan" OR "carmaz" OR "carnexiv" OR "carpaz" OR "carzepin" OR "carzepine" OR "clostedal" OR "convuline" OR "epileptol" OR "epimax" OR "epitol" OR "equetro" OR "espa-lepsin" OR "finlepsin" OR "foxalepsin" OR "g 32883" OR "g32883" OR "hermolepsin" OR "Iminostilbene-N-carboxamide" OR "karbamazepin" OR "kodapan" OR "lexin" OR "mazepine" OR "mazetol" OR "neugeron" OR "neurotol" OR "neurotop" OR "nordotol" OR "Novo-Carbamaz" OR "panitol" OR "servimazepin" OR "sirtal" OR "spd 417" OR "spd417" OR "Stazepin" OR "Stazepine" OR "tardotol" OR "taver" OR "tegol" OR "tegral" OR "tegretal" OR "tegretol" OR "tegrital" OR "telesmin" OR "temporol" OR "teril" OR "timonil" OR "Trimonil"

**42 results**, 7/15/18

OR

**"dimethyl phthalate"** OR "08X7F5UDJM" OR "131-11-3" OR "avolin" OR "citrola" OR "Dimethyl benzeneorthodicarboxylate" OR "Dimethyl o-phthalate" OR "Dimethyl orthophthalate" OR "dimethylphthalate" OR "dmp 30" OR "dmp30" OR "fermine" OR "Kemester DMP" OR "Kodaflex DMP" OR "methyl phthalate" OR "mipax" OR "mugia" OR "palatinol m" OR "Phthalic acid dimethyl ester" OR "Repeftal" OR "sketofax" OR "Solvanom" OR "Solvarone" OR "Unimoll DM" OR "Uniplex 110"

**23 results**, 7/15/18

OR

"dimethylammonium chloride" OR **"dimethylamine"** OR "124-40-3" OR "6912-12-5" OR "ARQ8157E0Q" OR "dimethlamine" OR "dimethyamine" OR "dimethyl amine" OR "di-methylamine" OR "dimethylammonia" OR "dimethylarnine" OR "dirnethylamine"

**10 results**, 7/15/18

OR
[truncated: 6,553 more chars]
