# Supplementary material for: A Systematic Review to Compare Chemical Hazard Predictions of the Zebrafish Embryotoxicity Test With Mammalian Prenatal Developmental Toxicity
Source: Toxicol Sci. 2021 Jun 9;183(1):14–35. doi: 10.1093/toxsci/kfab072 (PMC8404989; doi:10.1093/toxsci/kfab072)
Supplement: kfab072_Supplementary_Data [file kfab072_supplementary_data.zip › toxsci-21-0054-File005.docx]

**Supplemental Material 3: Tables. Characterization of included ZET datasets and mammalian studies**

**A systematic review to compare chemical hazard predictions of the zebrafish embryotoxicity test with mammalian prenatal developmental toxicity**

Sebastian Hoffmann^1,2*^, Bianca Marigliani^3^, Sevcan Gül Akgün-Ölmez^4^, Danielle Ireland^5^, Rebecca Cruz^6^, Francois Busquet^7^, Burkhard Flick^8^, Manoj Lalu^9^, Elizabeth C. Ghandakly^10^, Rob B.M. de Vries^1,11^, Hilda Witters^12^, Robert A. Wright^13^, Metin Ölmez^14^, Catherine Willett^15^, Thomas Hartung^16^, Martin L. Stephens^1^, Katya Tsaioun^1^

^1^ Evidence-Based Toxicology Collaboration (EBTC), Johns Hopkins Bloomberg School of Public Health, Baltimore, Maryland, 21205 (https://orcid.org/0000-0002-3214-7678)

^2^ seh consulting + services, 33106 Paderborn, Germany

^3^ Department of Science and Technology, Federal University of São Paulo (UNIFESP), São José dos Campos, São Paulo, Brazil (https://orcid.org/0000-0002-0498-4284)

^4^ Department of Pharmaceutical Toxicology, Faculty of Pharmacy, Marmara University, 34722 Istanbul, Turkey

^5^ Swarthmore College, Swarthmore, Pennsylvania 19081 (https://orcid.org/0000-0002-9827-1604)

^6^ Laboratory of Dental Clinical Research, Universidade Federal Fluminense, Niterói, RJ, Brazil

^7^ Altertox, Brussels, Belgium

^8^ Experimental Toxicology and Ecology, BASF SE, 67063 Ludwigshafen am Rhein, Germany

^9^ Department of Anesthesiology and Pain Medicine, Ottawa Hospital Research Institute,

Ottawa, Canada

^10^ Berman Institute of Bioethics, Johns Hopkins University, Baltimore, Maryland, 21205

^11^ Systematic Review Centre for Laboratory Experimentation (SYRCLE), Department for Health Evidence, Radboud Institute for Health Sciences, Radboudumc, Nijmegen, The Netherlands

^12^ VITO NV, 2400 Mol, Belgium (https://orcid.org/0000-0002-2026-3962)

^13^ William H. Welch Medical Library, Johns Hopkins University, Baltimore, Maryland 21205

^14^ Umraniye Family Health Center (No. 44), Turkish Ministry of Health, Istanbul, Turkey

^15^ Humane Society International, Washington, DC

^16^ Center for Alternatives to Animal Testing (CAAT), Johns Hopkins Bloomberg School of Public Health, Baltimore, Maryland 21205

*Corresponding author: [sebastian.hoffmann@seh-cs.com](mailto:sebastian.hoffmann@seh-cs.com)

In addition, the reference databases of the various selection steps will be provided openly accessible on zenodo.org.

| **Study (reference)** | **Chemical name** | **Zebrafish strain** | **Negative, vehicle, positive control (NC, VC, PC)** | **Test concentration range (µM)^1^** | **No. of conc.** | **Exposure (hpf)** | **Dechorio-nation** | **Timepoints of outcome measurement (hpf)** |
| --- | --- | --- | --- | --- | --- | --- | --- | --- |
|  |  |  |  |  |  |  |  |  |
| Selderslaghs et al. (2012) | acetaminophen | n.r. (wild type) | NC: test medium | 210 – 13,230 | ≥ 3 | 4-72‘ | NO | 24; 48; 72 |
| David and Pancharatna (2009) | acetaminophen | n.r. (wild type) | VC: 0.1% ethanol; NC: Distilled water | 6.62 – 662 | 5 | 0-120 | n.r. | 24; 48; 72; 96; 120 |
| Truong et al. (2014) | acetaminophen | Tropical 5D | VC: 0.64% DMSO; PC: TMTC (5 µM) | 0.0064 – 64 | 5 | 6-120 | YES | 24; 120 |
| Truong et al. (2014) | all-trans-retinoic acid | Tropical 5D | VC: 0.64% DMSO; PC: TMTC (5 µM) | 0.0064 – 64 | 5 | 6-120 | YES | 24; 120 |
| Selderslaghs et al. (2009) | all-trans-retinoic acid | n.r. (wild type) | VC: 0.1% DMSO | 0.00000852 – 16.6 | 10 | 2-72‘ | n.r. | 24; 48; 72 |
| Wang et al. (2014) | all-trans-retinoic acid | n.r. (wild type) | VC: 0.1% DMSO | 0.001 – 0.1 | 7 | 3-120 | n.r. | 24; 48; 72; 96; 120 |
| Vandersea et al. (1998) | all-trans-retinoic acid | n.r. | VC: 0.01% DMSO | 0.1 – 10 | 5 | 6-72 | NO | 72 |
| Teixido et al. (2013) | all-trans-retinoic acid | n.r. | n.r. | 0.0005 – 0.01 | 5 | 4-52 | NO | 8; 28; 52 |
| Selderslaghs et al. (2012) | all-trans-retinoic acid | n.r. (wild type) | NC: test medium; VC: 0.1% DMSO | 0.00000852 – 20 | ≥ 3 | 4-72‘ | NO | 24; 48; 72 |
| Piersma et al. (2013) | all-trans-retinoic acid | n.r. | NC: 3,4-DCA (6.2 µM); PC: 3,4-DCA (48.4 µM) | n.r. | 7 | 1-72 | NO | 72 |
| Wiegand et al. (2001) | atrazine | n.r. | VC: REKO | 23.12 – 185 | 5 | 1-48 | n.r. | 12; 24; 36; 48 |
| Weber et al. (2013) | atrazine | AB | NC: untreated | 0.14 – 0.0014 | 3 | 1-72 | n.r. | 72 |
| Ton et al. (2006) | atrazine | n.r. | VC: 0.1% DMSO | 200; 500; n.r. | ≥ 3 | 6-96 | NO | 24; 48; 72; 96 |
| Perez et al. (2013) | atrazine | n.r. | VC: DMSO; NC: distilled water | 92.5 – 185 | 5 | 3-96 | NO | 24; 48; 72; 96 |
| Truong et al. (2014) | atrazine | Tropical 5D | VC: 0.64% DMSO; PC: TMTC (5 µM) | 0.0064 – 64 | 5 | 6-120 | YES | 24; 120 |
| Truong et al. (2014) | butylparaben | Tropical 5D | VC: 0.64% DMSO; PC: TMTC (5 µM) | 0.0064 – 64 | 5 | 6-120 | YES | 24; 120 |
| Zhu et al. (2015) | caffeine | AB | n.r. | 275.5 – 5,150 | ≥ 3 | 6-54 | NO | 30; 54 |
| Chakraborty et al. (2011) | caffeine | n.r. | NC: untreated | 51.5 – 515 | 4 | 4-60 | n.r. | 60; 72; 84: 96 |
| Selderslaghs et al. (2009) | caffeine | n.r. (wild type) | NC: test medium | 50 – 12,900 | 9 | 2-72‘ | n.r. | 24; 48; 72 |
| Teixido et al. (2013) | caffeine | n.r. | n.r. | 100 – 2,500 | 5 | 4-52 | NO | 8; 28; 52 |
| Selderslaghs et al. (2012) | caffeine | n.r. (wild type) | NC: test medium | 50 – 12,870 | ≥ 3 | 4-72‘ | n.r. | 24; 48; 72 |
| Truong et al. (2014) | caffeine | Tropical 5D | VC: 0.64% DMSO; PC: TMTC (5 µM) | 0.0064 – 64 | 5 | 6-120 | YES | 24; 120 |
| Yim et al. (2014) | camphor | n.r. | VC: ethanol | 395 – 6,320 | 5 | 0-96 | n.r. | 24; 48; 72; 96 |
| Selderslaghs et al. (2012) | camphor | n.r. (wild type) | NC: test medium | 16 – 6,570 | ≥ 3 | 4-72‘ | NO | 24; 48; 72 |
| Truong et al. (2014) | clopyralid | Tropical 5D | VC: 0.64% DMSO; PC: TMTC (5 µM) | 0.0064 – 64 | 5 | 6-120 | YES | 24; 120 |
| Truong et al. (2014) | cyproconazole | Tropical 5D | VC: 0.64% DMSO; PC: TMTC (5 µM) | 0.0064 – 64 | 5 | 6-120 | YES | 24; 120 |
| Hermsen et al. (2011) | cyproconazole | n.r. | VC: 0.2% DMSO; NC: 3,4-DCA (6.2 µM); PC: 3,4-DCA (48.4 µM) | 0.1 – 316 | 7 | 1-72 | NO | 72 |
| Truong et al. (2014) | dimethyl phthalate | Tropical 5D | VC: 0.64% DMSO; PC: TMTC (5 µM) | 0.0064 – 64 | 5 | 6-120 | YES | 24; 120 |
| Truong et al. (2014) | ethylene glycol | Tropical 5D | VC: 0.64% DMSO; PC: TMTC (5 µM) | 0.0064 – 64 | 5 | 6-120 | YES | 24; 120 |
| Truong et al. (2014) | fluazinam | Tropical 5D | VC: 0.64% DMSO; PC: TMTC (5 µM) | 0.0064 – 64 | 5 | 6-120 | YES | 24; 120 |
| Ren et al. (2012) | genistein | AB | VC: 0.1% DMSO | 0.01 – 50 | 6 | 6-120 | NO | 24; 48; 72; 96; 120 |
| Truong et al. (2014) | genistein | Tropical 5D | VC: 0.64% DMSO; PC: TMTC (5 µM) | 0.0064 – 64 | 5 | 6-120 | YES | 24; 120 |
| Truong et al. (2014) | hexazinone | Tropical 5D | VC: 0.64% DMSO; PC: TMTC (5 µM) | 0.0064 – 64 | 5 | 6-120 | YES | 24; 120 |
| Gustafson et al. (2012) | lovastatin | n.r. (wild type 1) | VC: DMSO; NC: saccharin; PC: ATRA | 0.1 – 1,000 | 5 | 5-120 | NO | 120 |
| Gustafson et al. (2012) | lovastatin | WIK | VC: DMSO; NC: saccharin; PC: ATRA | 0.1 – 1,000 | 5 | 5-120 | NO | 120 |
| Gustafson et al. (2012) | lovastatin | n.r. | VC: DMSO; NC: saccharin; PC: ATRA | 0.1 – 1,000 | 5 | 5-120 | NO | 120 |
| Gustafson et al. (2012) | lovastatin | n.r. (wild type 2) | VC: DMSO; NC: saccharin; PC: ATRA | 0.0001 – 100 | ≥ 3 | 5-120 | YES | 120 |
| Truong et al. (2014) | lovastatin | Tropical 5D | VC: 0.64% DMSO; PC: TMTC (5 µM) | 0.0064 – 64 | 5 | 6-120 | YES | 24; 120 |
| Teixido et al. (2013) | methoxyacetic acid | n.r. | n.r. | 2,000 – 16,000 | 5 | 4-52 | NO | 8; 28; 52 |
| Hermsen et al. (2011) | methoxyacetic acid | n.r. | VC: water; NC: 3,4-DCA (6.2 µM); PC: 3,4-DCA (48.4 µM) | 10 – 10,000 | 7 | 1-72 | NO | 72 |
| Piersma et al. (2013) | methoxyacetic acid | n.r. | VC: water; NC: 3,4-DCA (6.2 µM); PC: 3,4-DCA (48.4 µM) | n.r. | 7 | 1-72 | NO | 72 |
| Zhang et al. (2013) | n-methylpyrrolidone | n.r. | NC: untreated | 880 – 26,400 | 5 | 5-120 | NO | 24; 48; 72; 96; 120 |
| Truong et al. (2014) | 2-phenylphenol | Tropical 5D | VC: 0.64% DMSO; PC: TMTC (5 µM) | 0.0064 – 64 | 5 | 6-120 | YES | 24; 120 |
| Pinho et al. (2013) | rotenone | n.r. (wild type) | n.r.; PC: Valproic acid | 0.01 – 1 | 5 | 4-80 | n.r. | 8; 32; 56; 80 |
| Melo et al. (2015) | rotenone | n.r. | NC: untreated | 0.0125 – 0.2 | 5 | 1-96 | NO | 24; 48; 72; 96 |
| Truong et al. (2014) | rotenone | Tropical 5D | VC: 0.64% DMSO; PC: TMTC (5 µM) | 0.0064 – 64 | 5 | 6-120 | YES | 24; 120 |
| Yang et al. (2015) | tetrabromobisphenol A | AB | NC: untreated | 0.092 – 1.84 | 4 | 4-96 | NO | 24; 32; 48; 96 |
| Song et al. (2014) | tetrabromobisphenol A | n.r. | VC: DMSO | 0.92 – 2.76 | 3 | (3)-120 | NO | 24; 48; 72; 96; 120 |
| Noyes et al. (2015) | tetrabromobisphenol A | Tropical 5D | VC: DMSO | 0.0064 – 64 | 5 | 6-120 | YES | 24; 120 |
| McCormick et al. (2010) | tetrabromobisphenol A | AB | VC: DMSO | 0.5 – 3 | 5 | 3-120 | NO | 24; 48; 72; 96; 120 |
| Hu et al. (2009) | tetrabromobisphenol A | AB | NC: untreated | 0.00368 – 2.76 | 6 | 2-96 | n.r. | 12; 24; 36; 48; 60 |
| Carlsson and Norrgren (2014) | tetrabromobisphenol A | n.r. | VC: 0.1% DMSO | 0.00184 – 1.84 | 4 | 1-48 | NO | 24; 48 |
| Baumann et al. (2016) | tetrabromobisphenol A | AB | n.r. | 0.185 – 0.74 | 4 | 2-120 | NO | 120 |
| Truong et al. (2014) | tetrabromobisphenol A | Tropical 5D | VC: 0.64% DMSO; PC: TMTC (5 µM) | 0.0061 – 61 | 5 | 6-120 | YES | 24; 120 |
| Gao et al. (2014) | thalidomide | n.r. (wild type) | VC: 0.1% DMSO | 2.76 – 27 | 4 | 2-72 | YES | 24; 48; 72; 96 |
| Selderslaghs et al. (2012) | thalidomide | n.r. (wild type) | NC: test medium; VC: 0.1% DMSO | 4.84 – 150 | ≥ 3 | 4-72‘ | NO | 24; 48; 72 |
| Truong et al. (2014) | thalidomide | Tropical 5D | VC: 0.64% DMSO; PC: TMTC (5 µM) | 0.0064 – 64 | 5 | 6-120 | YES | 24; 120 |
| Gustafson et al. (2012) | thalidomide | n.r. | VC: DMSO; NC: saccharin; PC: ATRA | 0.1 – 1,000 | 5 | 5-120 | NO | 120 |
| Gustafson et al. (2012) | thalidomide | n.r. (wild type 1) | VC: DMSO; NC: saccharin; PC: ATRA | 0.1 – 1,000 | 5 | 5-120 | NO | 120 |
| Gustafson et al. (2012) | thalidomide | n.r. (wild type 2) | VC: DMSO; NC: saccharin; PC: ATRA | 0.1 – 1,000 | 5 | 5-120 | NO | 120 |
| Gustafson et al. (2012) | thalidomide | WIK | VC: DMSO; NC: saccharin; PC: ATRA | 0.1 – 1,000 | 5 | 5-120 | NO | 120 |
| Gustafson et al. (2012) | thalidomide | n.r. (wild type 3) | VC: DMSO; NC: saccharin; PC: ATRA | 0.1 – 100 | 4 | 5-120 | YES | 120 |
| Truong et al. (2014) | triadimefon | Tropical 5D | VC: 0.64% DMSO; PC: TMTC (5 µM) | 0.0064 – 64 | 5 | 6-120 | YES | 24; 120 |
| Hermsen et al. (2011) | triadimefon | n.r. | VC: 0.2% DMSO; NC: 3,4-DCA (6.2 µM); PC: 3,4-DCA (48.4 µM) | 0.1 – 316 | 7 | 1-72 | NO | 72 |
| Truong et al. (2014) | triclopyr | Tropical 5D | VC: 0.64% DMSO; PC: TMTC (5 µM) | 0.0064 – 64 | 5 | 6-120 | YES | 24; 120 |
| Truong et al. (2014) | triethylene glycol | Tropical 5D | VC: 0.64% DMSO; PC: TMTC (5 µM) | 0.0064 – 64 | 5 | 6-120 | YES | 24; 120 |
| Herrmann (1993) | valproic acid | n.r. | NC: untreated | 10 – 10000 | 7 | 6-120 | n.r. | 24; 48; 72; 96; 120 |
| Beker van Woudenberg et al. (2014) | valproic acid | AB | VC: 0.2% DMSO | 60 – 730 | 4 | 0-96 | NO | 24; 48; 72; 96 |
| Selderslaghs et al. (2009) | valproic acid | n.r. (wild type) | NC: test medium | 470 – 12,000 | 9 | 2-72‘ | n.r. | 24; 48; 72 |
| Teixido et al. (2013) | valproic acid | n.r. | n.r. | 50 – 600 | 5 | 4-52 | NO | 8; 28; 52 |
| Selderslaghs et al. (2012) | valproic acid | n.r. (wild type) | NC: test medium | 50 – 13,870 | ≥ 3 | 4-72‘ | NO | 24; 48; 72 |
| Truong et al. (2014) | valproic acid | Tropical 5D | VC: 0.64% DMSO; PC: TMTC (5 µM) | 0.0064 – 64 | 5 | 6-120 | YES | 24; 120 |
| Piersma et al. (2013) | valproic acid # | n.r. | NC: 3,4-DCA (6.2 µM); PC: 3,4-DCA (48.4 µM) | n.r. | 7 | 1-72 | NO | 72 |
| Lee et al. (2013) | valproic acid # | AB | VC: 1% DMSO | 6.25 – 100 | 5 | 5.25-72 | n.r. | 24; 48; 72 |

Table S1: Characterization of included ZET datasets [hpf: hours post fertilization; DMSO: dimethyl sulfoxide; n.r.: not reported; ‘: exposure until 144 hpf; *: data reported differently; #: sodium salt (considered eligible); ^1^: reported units transformed to µM, if required; 3,4-DCA: 3,4-dichloroaniline; TMTC: trimethyltin chloride; ATRA: all-trans-retinoic acid; REKO: artificial egg medium]

| **Study (reference)** | **Chemical name** | **Method of administration** | **Species** | **Strain** | **Negative/vehicle control** | **Doses (mg/kg bw/day)** | **Treatment period (GD)** | **GD of sacrifice** |
| --- | --- | --- | --- | --- | --- | --- | --- | --- |
|  |  |  |  |  |  |  |  |  |
| Burdan et al. (2001) | acetaminophen | gavage | rat | Wistar | water/Tween 80 | 3.5; 35; 350 | 8-14 | 21 |
| Seegmiller et al. (1997) | all-trans-retinoic acid | gavage | rat | Wistar | hydroxypropyl  MC suspension | 1; 2.5; 5; 10 | 6-15 | 20 |
| Infurna et al. (1988) | atrazine | gavage | rat | Crl:CD® BR albino | 3% aqueous corn starch containing 0.5% Tween 80 | 10; 70; 100 | 6-15 | 20 |
| Infurna et al. (1988) | atrazine | gavage | rabbit | New Zealand White | 3% aqueous corn starch containing 0.5% Tween 80 | 1; 5; 75 | 7-19 | 29 |
| Daston (2004) | butylparaben | gavage | rat | Sprague-Dawley | 0.5% CMC | 10; 100; 1,000 | 6-19 | 20 |
| Collins et al. (1981) | caffeine | intubation | rat | Osborne Mendel | distilled water | 6; 12; 40; 80; 125 | 0-19 | 20 |
| Collins et al. (1983) | caffeine | drinking water | rat | Osborne Mendel | distilled water | 10.1; 25.9; 50.8; 100.8; 144; 216; 288 | 0-20 | 20 |
| Collins et al. (1987) | caffeine | drinking water | rat | Osborne Mendel | distilled water | 29.0; 48.8; 70.6 | 0-20 | 20 |
| Navarro et al. (1992a) | camphor | gavage | rat | Crl:CD® BR albino | corn oil | 100; 400; 800 | 6-15 | 20 |
| Navarro et al. (1992b) | camphor | gavage | rabbit | New Zealand White | corn oil | 50; 200; 400 | 6-19 | 30 |
| Leuschner (1997) | camphor | orally | rat | Sprague-Dawley | propylene glycol | 216; 464; 1000 | 6-17 | 20 |
| Hayes et al. (1984) | clopyralid | gavage | rat | Fischer 344 | vegetable oil | 15; 75; 250 | 6-15 | 21 |
| Machera (1995) | cyproconazole | gavage | rat | Wistar | CMC | 20; 50;75 | 6-16 | 21 |
| SDS-Biotech (1997) | cyproconazole | orally | rat | Wistar | distilled water containing 4% CMC sodium salt | 6; 12; 24; 48 | 6-15 | 21 |
| SDS-Biotech (1997) | cyproconazole | orally | rabbit | Chinchilla | distilled water containing 4% CMC sodium salt | 2; 10; 50 | 6-18 | 29 |
| Field et al. (1993) | dimethyl phthalate | diet | rat | Sprague-Dawley | untreated diet | 200; 840; 3,570 | 6-15 | 20 |
| Maronpot et al. (1983) | ethylene glycol | diet | rat | Fischer 344 | same ground diet | 40; 200; 1,000 | 6-15 | 21 |
| **Neeper-Bradley (1990)** | ethylene glycol | gavage | rat | Sprague-Dawley | deionized water | 150; 500; 1,000; 2,500 | 6-15 | 21 |
| **Price et al. (1992)** | ethylene glycol | gavage | rat | Crl:CD® BR albino | distilled water | 1,250; 2,500; 5,000 | 6-15 | 20 |
| Tyl et al. (1991) | ethylene glycol | gavage | rabbit | New Zealand White | distilled deionized water | 100; 500; 1,000; 2,000 | 6-19 | 30 |
| **Tesh et al. (1992)** | fluazinam | gavage | rat | Crl:CD® BR albino | corn oil | 10: 50; 250 | 6-15 | 20 |
| McClain et al. (2007) | genistein | diet | rat | Wistar | diet only | 5; 50; 100; 500 | 5-21 | 21 |
| Kennedy and Kaplan (1984) | hexazinone | diet | rat | Crl:CD® BR albino | diet only | 4.74; 22.5; 107 | 6-15 | 21 |
| Kennedy and Kaplan (1984) | hexazinone | gavage | rabbit | New Zealand White | 0.5% aqueous MC | 20; 50; 125 | 6-19 | 29 |
| Lankas et al. (2004) | lovastatin | orally | rat | Crl:CD® BR albino | 0.5% (w/v) MC in deionized water | 100; 200; 400; 800 | 6-20 | 21 |
| Minsker et al. (1983) | lovastatin | orally | rat | Crl:CD® BR albino | 0.5% aqueous MC | 8; 80; 800 | 6-17 | 20 |
| Carney et al. (2003) | methoxyacetic acid | gavage | rabbit | New Zealand White | distilled water | 2.5; 7.5; 15 | 7-19 | 28 |
| Saillenfait et al. (2002) | n-methylpyrrolidone | gavage | rat | Sprague-Dawley | distilled water | 125; 250; 500; 750 | 6-20 | 21 |
| **Anonymous (1991)** | 2-phenylphenol | gavage | rat | Sprague-Dawley | cottonseed oil | 100; 300; 700 | 6-15 | 21 |
| **Anonymous (1992)** | 2-phenylphenol | gavage | rabbit | New Zealand White | corn oil | 25; 100; 250 | 7-19 | 28 |
| Kaneda et al. (1978) | 2-phenylphenol | intubation | rat | Wistar | distilled water with the aid of gum arabic (5%) | 150; 300; 600; 1,200 | 6-15 | 20 |
| Khera et al. (1982) | rotenone | gavage | rat | Wistar | corn oil | 2.5; 5; 10 | 6-15 | 22 |
| Cope et al. (2015) | tetrabromobisphenol A | gavage | rat | Sprague-Dawley | corn oil | 100; 300; 1,000 | 6-15 | 20 |
| Sterz et al. (1987) | thalidomide | gavage | rabbit | Himalayan (Russian) | 0.5% aqueous solution of MC | 50; 100; 150; 200 | 8-11 | 30 |
| **Machener (1992)** | triadimefon | gavage | rat | Long-Evans | cremophor | 10; 30; 50; 75; 100 | 6-15 | 20 |
| Hanley et al. (1984) | triclopyr | gavage | rat | Sprague-Dawley | 0.5% hydroxy-propyl MC | 50; 100; 200 | 6-15 | 20 |
| Ballantyne and Snellings (2005) | triethylene glycol | gavage | rat | Crl:CD® BR albino | distilled water | 1,126; 5,630; 11,260 | 6-15 | 21 |
| Ong et al. (1983) | valproic acid | intubation | rat | CD | 10% acacia | 50; 150; 600 | 6-15 | 21 |
| Petrere et al. (1986) | valproic acid | gavage | rabbit | Dutch Belted | 10% acacia | 50; 150; 350 | 6-18 | 30 |
| Vorhees (1987) | valproic acid | gavage | rat | Sprague-Dawley | propylene glycol | 200; 300; 400; 600 | 7-18 | 20 |

Table S2: Characterization of included mammalian studies [*: unit is mg/kg bw/day; GD: gestational day; (C)MC: (carboxy)methylcellulose; bold references refer to studies submitted to the Office of Toxic Substances of the US Environmental Protection Agency]
